# Comprehensive analyses of the cancer-associated fibroblast subtypes and their score system for prediction of outcomes and immunosuppressive microenvironment in prostate cancer

**DOI:** 10.1186/s12935-024-03305-5

**Published:** 2024-04-05

**Authors:** Ze Gao, Ning Zhang, Bingzheng An, Dawei Li, Zhiqing Fang, Dawei Xu

**Affiliations:** 1 Department of Urology, Qilu Hospital of Shandong University, Jinan, 250012 China; 2 Institute of Andrology, Shandong University, Jinan, 250012 China; 3 Department of Breast Surgery, Qilu Hospital of Shandong University, Jinan, 250012 China; 4 Department of Medicine, Division of Hematology, Bioclinicum, Karolinska Institute and, Karolinska University Hospital, Solna, Stockholm, SE-17176 Sweden

**Keywords:** Caner associated fibroblasts, Immunotherapy, Prostate cancer, Tumor microenvironment, Tumor progression

## Abstract

**Background:**

Cancer-associated fibroblasts (CAFs) drive cancer progression and treatment failure on one hand, while their tumor-restraining functions are also observed on the other. Recent single cell RNA sequencing (scRNA-seq) analyses demonstrates heterogeneity of CAFs and defines molecular subtypes of CAFs, which help explain their different functions. However, it remains unclear whether these CAF subtypes have the same or different biological/clinical implications in prostate cancer (PCa) or other malignancies.

**Methods:**

PCa cells were incubated with supernatant from normal fibroblasts and CAFs to assess their effects on cell behaviors. Sequencing, genomic, and clinical data were collected from TCGA, MSKCC, CPGEA and GEO databases. CAF molecular subtypes and total CAF scores were constructed and grouped into low and high groups based on CAF-specific gene expression. Progression free interval (PFI), clinicopathological features, telomere length, immune cell infiltration, drug treatment and somatic mutations were compared among CAF molecular subtypes and low/high score groups.

**Results:**

The PCa CAF-derived supernatant promoted PCa cell proliferation and invasion. Based on differentially expressed genes identified by scRNA-seq analyses, we classified CAFs into 6 molecular subtypes in PCa tumors, and each subtype was then categorized into score-high and low groups according to the subtype-specific gene expression level. Such score models in 6 CAF subtypes all predicted PFI. Telomeres were significantly shorter in high-score tumors. The total CAF score from 6 CAF subtypes was also associated with PFI in PCa patients inversely, which was consistent with results from cellular experiments. Immunosuppressive microenvironment occurred more frequently in tumors with a high CAF score, which was characterized by increased CTLA4 expression and indicated better responses to CTLA4 inhibitors. Moreover, this model can also serve as a useful PFI predictor in pan-cancers.

**Conclusion:**

By combining scRNA-seq and bulk RNA-seq data analyses, we develop a CAF subtype score system as a prognostic factor for PCa and other cancer types. This model system also helps distinguish different immune-suppressive mechanisms in PCa, suggesting its implications in predicting response to immunotherapy. Thus, the present findings should contribute to personalized PCa intervention.

**Supplementary Information:**

The online version contains supplementary material available at 10.1186/s12935-024-03305-5.

## Introduction

Prostate cancer (PCa) is the second most common malignancy worldwide, causing about 375,000 deaths in 2020 [1], and the incidence has arose rapidly over the past decades [2]. Most PCa patients have no obvious symptoms in the early stages and are usually diagnosed when an advanced disease has developed [3]. The 5-year survival rate for men with a localized tumor is as high as 99%, while only 28% for those with metastasis [4]. Androgen deprivation therapy (ADT) is the first-line treatment option for advanced PCa, but castration-resistant prostate cancer (CRPC) occurs eventually, which leads to treatment failure and disease progression [5].

PCa progression and CRPC are driven by genetic/epigenetic factors that maintain active androgen-androgen receptor (AR) signaling [6]. The direct *AR* gene alterations, including amplification, mutation, and alternative splicing, have been well characterized to lead to CRPC or advanced PCa, while AR-associated factor dysregulations act as important contributors, too [6]. In addition, aberrant epigenetics interact with genetic alterations or directly regulate AR signaling to promote CRPC development [7]. For instance, histone H2A Lys130-acetylation stimulates androgen production, thereby resulting in CRPC [8].

In addition to intrinsic mechanisms, evidence has accumulated that tumor microenvironment (TME) plays a pivotal role in cancer progression, such as angiogenesis induction, invasion or metastasis and therapeutic resistance [9–13]. The components of TME include cancer-associated fibroblasts (CAFs), endothelial cells and pericytes, various immune and inflammatory cells, bone marrow derived cells, and extracellular matrix (ECM) [14]. CAFs are the predominant stromal cell type in the TME and secrete growth factors, inflammatory ligands and extracellular matrix (ECM) proteins, thereby promoting carcinogenesis [10, 11, 15–17]. On the other hand, CAFs may also exert tumor-restraining effects [17–19]. The development of single-cell RNA sequencing (scRNA-seq) technology has revealed the heterogeneity of not only tumor cells, but also cells in the TME [20]. CAFs have been classified into several subtypes in previous studies [11, 15, 21], and more recently, Luo et al. further showed CAF heterogeneity and diversity across human solid tumors based on scRNA-seq analyses and they molecularly stratified CAFs into the following 6 subtypes: cancer-associated myofibroblasts (CAFmyo), inflammatory CAFs (CAFinfla), adipogenic CAFs (CAFadi), endothelial-to-mesenchymal transition CAF (CAFendMT), peripheralnerve-like CAF (CAFpn), and antigen-presenting CAF (CAFap) [22]. Moreover, these 6 subtypes of CAFs shared similar transcriptomic profiles in all analyzed 10 different solid tumors including PCa. These findings provide insights into the diverse roles of CAFs in cancer biology. However, several issues remain unsolved in PCa. First, whether these CAF subtypes are involved in the PCa pathogenesis differently? Second, whether these CAF subtypes are associated with PCa outcomes differently? Finally, the requirement of fresh samples, time-consuming handling procedure and unfriendly cost significantly limit scRNA-seq application, whereas bulk transcriptome sequencing remains the most frequent approach for RNA expression profiling, especially for analyses of large numbers of tumors or tissues. Thus, it raises an important question of whether scRNA-seq data can be translated into bulk RNA analyses, and if so, it will be easier to make them suitable for future clinical application. The present study is designed to address this issue. We first demonstrated that supernatant derived from primary PCa CAFs strongly promoted PCa cell proliferation and migration. Based on specific biomarkers identified using scRNA-seq analyses, we then applied them to the tumor bulk RNA seq data in public databases to classify molecular subtypes of CAFs and then establish the CAF score in PCa. Our results show that the CAF (subtype and total) score model is a robust predictor for PCa outcomes and immunosuppressive microenvironment. Moreover, we further verified the usefulness of this model system in other solid tumors.

## Patients and methods

### PCa patients, specimens, and isolation of normal fibroblasts (NFs) and CAFs

Three patients were included in the present study, which was approved by the Shandong University Qilu Hospital Ethics Committee (#KYLL-202208-044). The clinical information for 3 patients is listed in Table 1. Tumors and matched non-tumorous prostate tissues (NTs) were obtained from these patients undergoing radical prostatectomy without other therapies. Both tumors and NTs were minced and washed followed by the addition of 2 ml of digestion solution (A430371, Asegene, China) and incubation for 2 h at 37 °C. The tissues were blown vigorously 20 times with a pasteur pipet, then allowed to stand and filtered through a 100 μm strainer (R20B01060005, Biosharp, China). The filtered solutions were incubated with 20% fetal bovine serum (FBS) (ExCell Bio, China) containing RPMI-1640 medium (C11875500BT, Gibco, USA) in 6-well plates for 4 days, and adherent NFs and CAFs were then digested and harvested.

**Table 1:** The clinical information of 3 patients with prostate cancer in the present study

Patient	Age (Years)	PSA	Gleason score	Survival state
#1	68	48.30 ng/ml	4 + 3 = 7	Alive
#2	64	100.00 ng/ml	5 + 4 = 9	Alive
#3	56	65.90 ng/ml	4 + 5 = 9	Alive

### Cell culture, supernatant harvest, and supernatant treatment of PCa-derived cell lines

One million NFs and CAFs were seeded into 10 cm culture dishes and cultured in 10 ml of serum-free medium for 2 days. Supernatant was then collected. PCa cell lines PC-3 and DU145 were purchased from the National Collection of Authenticated Cell Cultures (Shanghai, China). PC-3 cells were cultured in RPMI-1640 medium (Gibco, USA) and DU145 in DMEM medium (C11995500BT, Gibco). Cell culture medium was supplemented with 10% FBS (ExCell Bio, China) and 1% penicillin/streptomycin (C100C5, NCM Biotech, China). All cells were mycoplasma free and cultured at 37 °C in a humidified 5% CO_2_ atmosphere. Supernatant from NF and CAF medium was added into plates where PC-3 and DU145 cells were incubated, and a final supernatant concentration was 50%.

### Western blotting

Cellular proteins were extracted from PC-3 and DU145 cells with NF and CAF supernatant, and protein concentrations were determined using the BCA kit (P0011, Beyotime, China). Western blot was performed as described [23]. The antibodies used in this study include FAP (66,562 S, CST, USA), PDGFRα/β (ab5443, Abcam, UK), α-SMA (A17910, ABclonal, USA) and GAPDH (10494-1-AP, Proteintech, USA).

### Cell viability and proliferation assay

Cell viability and proliferation was measured using a CCK-8 kit (K1018, APExBIO, USA). PC-3 and DU145 cells were seeded in 96-well plates at a density of 1000 cells/well. CCK-8 solution (10 µL) was added, and the cell proliferation curve was plotted based on the assay values within 7 consecutive days. Living cell numbers in the control and experimental groups were detected color-metrically according to the manufacture’s protocol.

### Transwell assay

PC-3 and DU145 cells were diluted with serum-free medium at a density of 50 000/ well and added to Transwell chambers (353,097, Falcon, USA). The medium mixed with NF and CAF supernatants (50% supernatant plus 50% complete medium) were added to 24-well plates, respectively. The plates containing PC-3 and DU145 were then placed in an incubator at 37 °C for 24 h and 12 h, respectively. The chambers were fixed with 4% paraformaldehyde for 15 min, stained with 0.2% crystal violet for 15 min, and allowed to dry before filming.

### Data acquisition and processing

The RNA sequencing data (standardized), somatic mutation data and clinical data of PCa and other solid tumor were obtained from the Cancer Genome Atlas (TCGA) database (https://portal.gdc.cancer.gov/). RNA abundance was expressed as transcripts per million (TPM). In further analyses, bulk RNA-seq data were log2 (TPM + 1) transformed. Differential gene analysis was analyzed by Wilcox T test using limma package.

The scRNA-seq data of CAFs in PCa tissues were obtained from the Gene Expression Omnibus (GEO) (https://www.ncbi.nlm.nih.gov/geo/). Two PCa cohorts (GSE85606 and GSE68164) with scRNA-seq analyses were obtained to evaluate NF and CAF associated gene expression levels in PCa tissues. The external validation datasets were downloaded from the Memorial Sloan-Kettering Cancer Center (MSKCC) (http://cbio.mskcc.org/cancergenomics/prostate/data), Chinese Prostate Cancer Genome and Epigenome Atlas (CPGEA) (http://www.cpgea.com/) and GSE70770 (https://www.ncbi.nlm.nih.gov/geo/). The RNA-seq and somatic mutation data processing were performed using the R software packages limma and maftools.

### Construction of the CAF score

CAFs in PCa tumors were divided into six categories or subtypes according to Luo et al [22]. We performed univariate Cox regression analysis of the top 30 genes expressed in each category and used the coefficient values to establish CAFs models for different subtypes. The total CAF score was calculated based on all CAF-associated genes. The CAF score formula was established as follows:

$$\text{CAF\, score} = \sum _{i}Coefficient \,of\,\left(i\right)\times \,Expression \,of gene\,\left(i\right)$$

### Gene set enrichment

The single-sample gene set enrichment analysis (ssGSEA) was used to quantify the enrichment level of immune characteristics in each sample, including immune cell types, functions and pathways in R language. To identify the regulatory pathways that differed between the two groups, gene set enrichment analysis (GSEA) was performed using the Pi package in R language. In addition, gene set variation analysis (GSVA) was performed using the GSVA package in R for CAF score low and high groups.

### Immune microenvironment analysis

We downloaded the immunophenoscore (IPS) of each PCa patient from the cancer immune group atlas (TCIA) (https://tcia.at/home). Tracking tumor immunotype (TIP) was used to evaluate the anti-tumor immunity of the seven-step immune cycle in PCa tissues (http://biocc.hrbmu.edu.cn/TIP/). TCGA solid tumors were classified as previously described by Thorsson et al. [24]. For PCa tumors, 4 categories were stratified, which include wound healing (C1), IFN-g dominant (C2), inflammatory (C3) and lymphocyte depleted (C4). Immune checkpoint and cytolytic activity (CYT) scores were used to predict the response to immune checkpoint inhibitors (ICIs), through which a potential association between CAF scores and immunotherapy efficacy was assessed.

### Telomere length analysis

Telomere length data in PCa tumors in the TCGA database were obtained from the previous analyses by Barthel et al. [25].

### Statistical analysis

All statistical analyses were performed using R software (version 4.2.1) and Graphpad prism. The Kaplan-Meier analysis of progression free interval (PFI) was performed with use of the survival and survminer package by R language. Wilcox t test was used for comparison between groups including DEGs analyses. *P* < 0.05 was considered statistically significant if not specified.

## Results

### CAF-mediated proliferation and migration of PCa cells

**Fig. 1:**
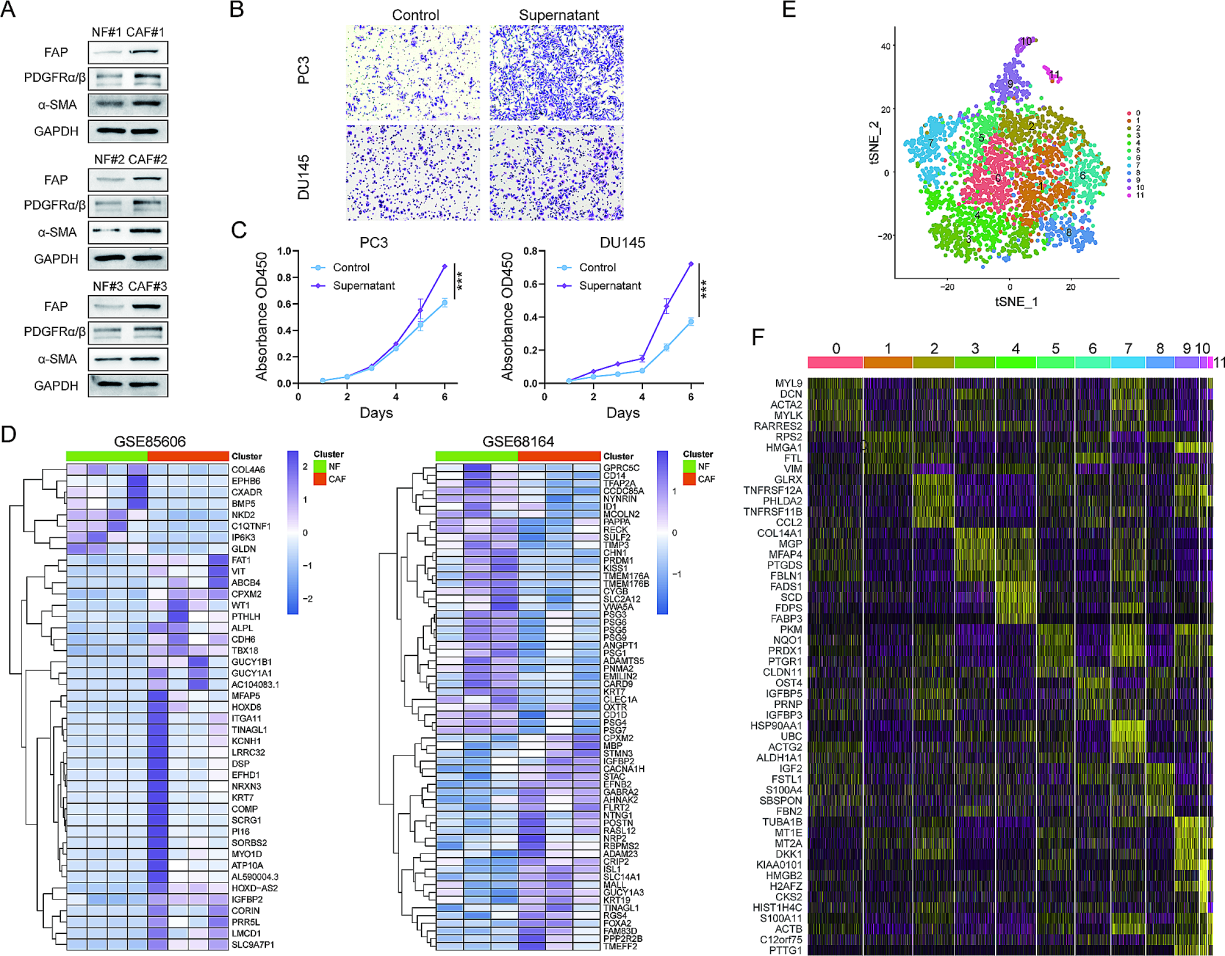
CAF heterogeneity and function in prostate cancer (PCa). (A) Western blot analysis for FAP, PDGFRα/β, α-SMA and GAPDH in the NFs (Normal fibroblasts) and CAFs (cancer-associated fibroblasts). (B) Transwell assay of PC-3 and DU145 cell migration mediated by CAF culture supernatant. (C) CCK-8 assessment of PC-3 and DU145 cell proliferation mediated by CAF culture supernatant. (D) Heatmap of differentially expressed genes (DEGs) in paired NFs and CAFs. (E) T-SNE plot of single cells from CAFs in PCa tissues. (F) Heatmap shows the marker genes of s distributed in the 12 clusters.

Primary NFs and CAFs derived from 3 PCa patients were isolated, and their identity was verified by western blot using their specific biomarkers (α-SMA, PDGFRα/β and FAP) (Fig. 1A). The culture supernatant of CAFs and NFs were collected for cellular experiments. As shown in Fig. 1B and C, the CAF-derived supernatant significantly facilitated PC-3 and DU145 cell migration (Fig. 1B) and proliferation (Fig. 1C).

### CAF heterogeneity in PCa tumors

Given the findings above, we sought to probe potential mechanisms underlying CAF-driven PCa cell migration/proliferation. Towards this end, we first analyzed transcriptomic profiles of NFs and CAFs in GSE85606 and GSE68164 PCa cohorts to identify differentially expressed genes (DEGs) between them. A total of 43 and 63 DEGs (|LogFC| > 1 and *P* < 0.05) were found in GSE85606 and GSE68164 cohorts, respectively (Fig. 1D, and Tables S1 and S2). There were only 4 overlapping DEGs (KRT7, IGFBP2, CPXM2 and TINAGL1) in both cohorts, among which KRT7 expression showed opposite trends. We further analyzed scRNA-seq data of CAFs and observed that CAFs could be divided into 11 clusters (Fig. 1E). Each of these 11 clusters contained unique top DEGs (Fig. 1F and Table S3). The differences in the DEGs among each cluster demonstrate the heterogeneity and plasticity of CAFs (Figure S1). Likely, those identified DEGs render CAFs stimulatory effects on PCa cell prolifreation and migration. Alternatively, the DEGs mark oncogenic CAF subpopulations.

### Establishment of the CAF subtype score to predict patient PFI

**Fig. 2:**
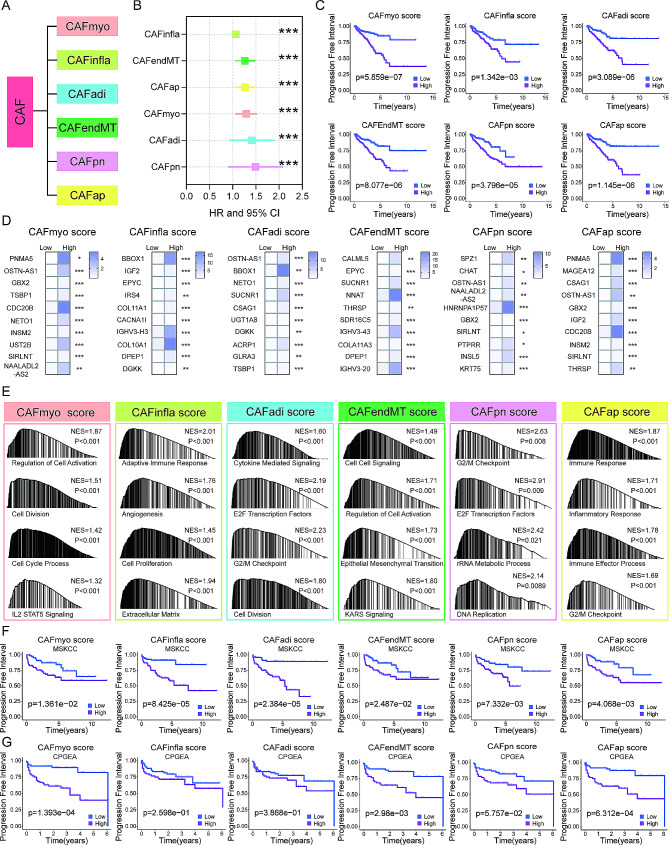
The CAF subtype classification and scores for progression prediction in PCa. (A) The classification of CAF subtypes. (B) Univariate Cox regression analysis of CAF subtype scores and association with progression-free interval (PFI). (C) Kaplan-Meier analysis of the CAF subtype scores and association with PFI. (D) Heatmap of top 10 differentially expressed genes in CAF subtype score low and high groups. (E) GSEA results showing the activated signaling pathways in the CAF score high group. (F-G) The validation of the CAF subtype score model to predict PFI, as determined by Kaplan-Meier analysis in MSKCC and CPGEA PCa cohorts

To gain insights into CAF-driven PCa aggressiveness in more depth and more broadly, we further focused on the molecularly classified CAF subtypes. Based on the molecular heterogeneity of CAF populations obtained from scRNA-seq in solid tumors, CAFs have recently been stratified into the following 6 categories [22]: CAFmyo, CAFinfla, CAFadi, CAFendMT, CAFpn, and CAFap (Fig. 2A). To determine the effect of each CAF subtype on PCa progression and outcomes, we applied this CAF classification system to the bulk RNA-seq profiled PCa tumors (TCGA cohort) by using the top 30 expressed genes in each CAF subtype (Table S4), and CAFs in these PCa tumors were successfully categorized into the identical 6 subtypes, too. Univariate Cox regression analysis was first used to examine the association between patient PFI and the expression levels of top 30 genes in each CAF subtype (Figure S2A), but not all those 30 genes could predict PFI, indicating the role of the subtype rather than gene expression as per. Nevertheless, the CAF scores of each category were constructed according to the expression level of those 30 top genes. Using the median score as the cutoff, the CAF score was associated with PFI in all subtypes (Fig. 2B and C). Among the 6 different CFA categories, the area under the ROC curve (AUC) of the scores in all 6 categories was the largest in 7 years (Figure S2B). We further identified the top 10 DEGs in each subtype (Fig. 2D), and subsequent GSEA analyses showed both different and overlapping pathway enrichments among 6 subtypes with high CAF scores (Fig. 2E). The enriched pathways in the high-score groups mainly include cell proliferation, ECM, EMT, angiogenesis, inflammation and immune responses, which are intimately associated with tumor progression. The co-expression network between CAF typing and CAF-related genes was analyzed by Sankey plot (Figure S3A). The regulatory network of CAF-related genes in different subtypes represents the study of expression correlation and tumor progression in PCa patients (Figure S3B).

We further analyzed PCa cohorts from the MSKCC, CPGEA and GEO datasets to validate the CAF score model as a prognostic factor observed in the TCGA PCa patients. For the MSKCC cohort, similar results were obtained. In the six subtypes of CAF score models, patients in the high score group had more rapid disease progression than those in the low score one (Fig. 2F). In the CPGEA cohort, patients in the high score group had shorter PFI, but statistical significances were reached only for CAFmyo, CAFendMT and CAFap subtypes, while at a board-line for CAFpn (Fig. 2G). The GSE70770 cohort analysis showed that the scores for CAFmyo, CAFendMT and CAFap subtypes were significantly associated with patient PFI (Figure S6A), as observed in the CPGEA cohort.

### The total CAF score model as a predictor for PCa patient PFI and treatment response

**Fig. 3:**
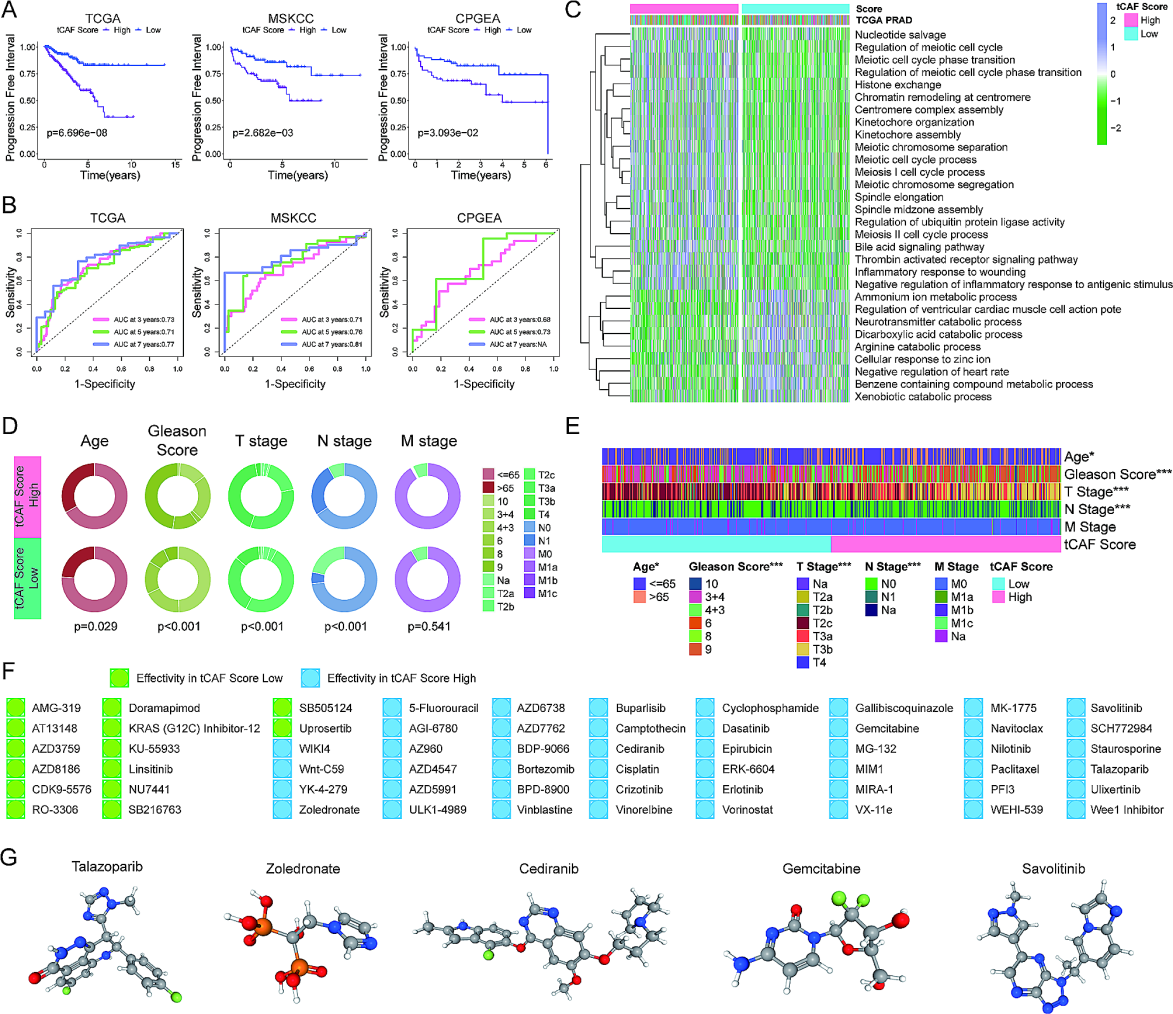
The tCAF score model and association with survival, clinical features and drug sensitivity in PCa. (A) Kaplan-Meier analysis of the tCAF score low and high groups in TCGA, MSKCC and CPGEA PCa cohorts. (B) ROC curves of tCAF scores at 3, 5, and 7 years in the TCGA cohorts. (C) GSVA enrichment analysis showing the activation states of biological pathways in the tCAF score low and high groups. (D-E) Differences in clinical characteristics between the tCAF score low and high groups in the TCGA PCa cohort. (F) The effect of tCAF scores on response to commonly used drugs. (G) The 3D structure tomographs of 5 candidate small-molecule drugs for tCAF score high groups in PCa.

To simplify the CAF subtype score system above for potential clinical application, we integrated six subtype scores and all CAF associated genes to construct a total CAF (tCAF) score model. Taking the median tCAF score as the cutoff, the analysis of TCGA, MSKCC, CPGEA and GSE70770 PCa cohorts showed that tCAF score had high accuracy in predicting PFI. The patient PFI in the tCAF high group was significantly worse than that in the low one (Figs. 3A and S6B). The ROC curves for each group, when the third, fifth, and seventh years were evaluated as the end time points, demonstrated the robust predictive power of the tCAF score model (Figs. 3B and S6C). GSVA pathway analysis unraveled that proliferation-related pathways were highly enriched in the tCAF score high group (Fig. 3C). Further assessments of the TCGA PCa cohort showed that there were significant differences between the tCAF score and age, Gleason score, T and N stages (Fig. 3D and E). The Gleason score, an important indicator in PCa, was significantly higher in the tCAF score high group. Next, we explored whether the tCAF score could be used in the selection of drug therapy (excluding ADT) in PCa patients. The IC50 value of each drug for each patient in the tCAF score low and high groups was calculated using the oncoppredict package. We computationally identified 14 drugs that were more effective in the tCAF score low group and 46 drugs that were more effective in the tCAF score high group (Fig. 3F and Figure S4). The 3D structural tomography of talazoparib, zoledronate, cediranib, gemcitabine, and savolitinib that could potentially be used to treat patients in the tCAF high group was searched in PubChem database (Fig. 3G).

### Correlation of tCAF scores with genomic alterations in PCa tumors

**Fig. 4:**
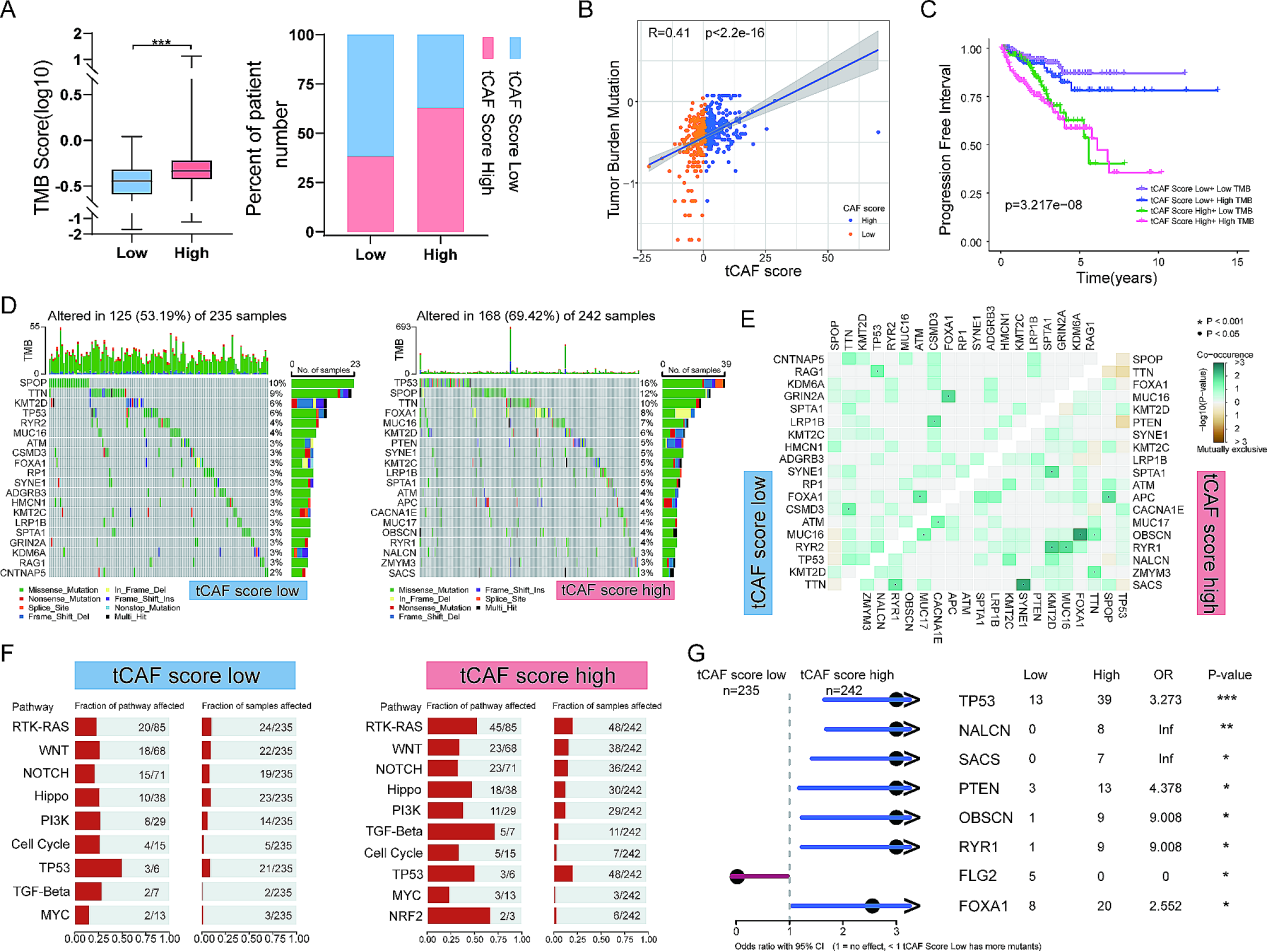
The mutational profiles in tCAF score low and high PCa tumors. (A-B) The relationship between TMB and tCAF scores. (C) Kaplan-Meier analysis of the tCAF score and TMB. (D) The frequencies of mutated genes among the tCAF score low and high groups. (E) Mutual exclusivity and co-occurrence of mutations in tCAF score low and high groups. (F) The mutation frequencies of common oncogenic pathways in two tCAF score subtypes. (G) Differentially mutated genes in two tCAF score subtypes

We calculated the tumor mutation burden (TMB) for each patient in the TCGA PCa cohort. The TMB in the tCAF high score group was significantly higher (*p* < 0.001) (Fig. 4A). There was a highly positive correlation between TMB and tCAF score (Fig. 4B). The overall TMB was 53.19% and 69.42% in the tCAF score low and high groups, respectively (Fig. 4D). The prognosis of patients with both tCAF score and TMB low was much better (Fig. 4C). Figure 4E showed the mutual exclusivity and co-occurrence of mutations in tCAF score groups. We further examined the mutation frequencies of nine major oncogenic pathways in the tCAF score low and high groups. Nine major oncogenic pathways were detected in the tCAF score low group, while 10 major oncogenic pathways were detected in the tCAF score high group, mainly including RTK-RAS, WNT, NOTCH and Hippo pathway (Fig. 4F). Cancers differ from each other in their mutational patterns. We examined these differentially mutated genes by comparing the two cohorts of CAF score. The results showed that besides FLG2, TP53, NALCN, SACS, PTEN, OBSCN, RYR1 and FOXA1 were highly mutated in the CAF score high group (Fig. 4G). CNV alterations (mainly copy number deletions) occurred more frequently in all the CAF score subtype high groups (Figure S5).

### Effect of the tCAF score on immune status in PCa tumors

**Fig. 5:**
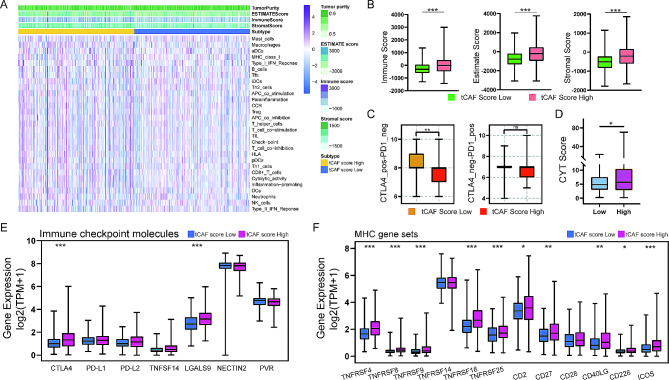
The tCAF score prediction of response to immunotherapy in PCa. (A) Twenty-nine immune-related gene sets were enriched in TCGA PCa cohort. (B) The stromal, immune and ESTIMATE scores between two tCAF score subtypes. (C-D) The IPS and CYT scores between two tCAF score subtypes. (E-F) The expression of immune checkpoint related genes and MHC gene set in two tCAF score groups

The PCa tumors in the TCGA cohort were scored using ssGSEA to quantify the activity, enrichment level and function of immune cells in each sample, and then grouped according to their tCAF scores (Fig. 5A). The immune status was more active in the tCAF high score group. Based on the expression profile, the ESTIMATE algorithm was used to calculate the stromal, immune and ESTIMATE scores of PCa. The results showed that the ESTIMATE, immune and stromal scores in the tCAF score high group were all higher than those in the low one (Fig. 5B). The cytolytic activity (CYT) score, an immunotherapy biomarker characterizing the antitumor immune activity of CD8^+^ cytotoxic T cells and macrophages, was significantly higher in the tCFA score high group (Fig. 5D). These results indicate the active immune status in the high tCAF score tumors. IPS is a quantitative index to evaluate the cancer-immunity cycle (CIC) efficacy. In the case of CTLA4 expression, the tCAF score low group had a better response to immunotherapy, while there was no difference between the two groups in the case of PD-L1 expression (Fig. 5C). Consistently, CTLA4 was significantly expressed in the tCAF score high group, while NECTIN2 was significantly expressed in the low group. The expression of PD-L1, PD-L2 and CCA did not differ significantly between the two groups (Fig. 5E). We further examined the relationship between tCAF scores and major histocompatibility complex (MHC). Except for TNFRSF14 and CD28, the expression level of MHC gene sets tended to be higher in the tCAF high score group (Fig. 5F).

### Association between the tCAF score and immune cell infiltration

**Fig. 6:**
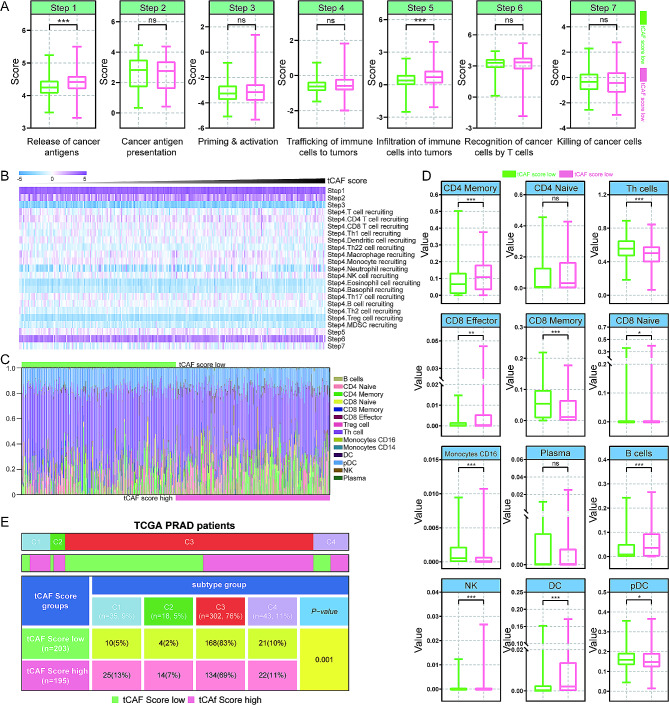
Immune cell infiltration in CAF score low and high PCa tumors. (A) Tumor immune cycle analysis. (B) Heatmap of the seven-step cancer immunity cycle and tCAF score. (C-D) Proportional plot of immune cell infiltration in two tCAF score groups. (E) The immunological classification of solid tumors with tCAF score

Antitumor immunity in tumor tissue can be interpreted as seven sequential processes, including release of cancer antigens (step 1), cancer antigen presentation (step 2), priming and activation (step 3), Tracking of immune cells to tumors (step 4), infiltration of immune cell into tumors (step 5), recognition of cancer cells by T cells (step 6), and killing of cancer cells (step 7). Although only step1 and step5 showed active status in the tCAF score high group, step2, step3, step4, step6 and step7 showed similar active status in both groups (Fig. 6A and B). Further analysis of infiltrated immune cells in tumor tissues showed that CD4 memory, CD8 effector, CD8 naive, B cells, NK cells and DC cells were more abundant in the tCAF score high group than the tCAF score low group (Fig. 6C and D). However, the degree of infiltration of Th cells, CD8 memory, Monocytes CD16, and pDC cells was reversed (Fig. 6D). Based on the immunological classification of solid tumors by Thorsson et al. [24], we further examined distributions of immune subtypes in two tCAF score groups. In the tCAF score low group, C1 (wound healing), C2 (IFN-gamma dominant), C3 (inflammatory), and C4 (lymphocyte depleted) accounted for 5%, 2%, 83%, and 10%, respectively. However, in the tCAF score high group, C1, C2, C3 and C4 accounted for 13%, 7%, 69% and 11%, respectively (Fig. 6E). The proportion of C1 and C2 was significantly higher, while the proportion of C3 was lower in the tCAF score high group.

### Shorter telomeres in the tCAF score high PCa tumors and association with unfavorable PFI

**Fig. 7:**
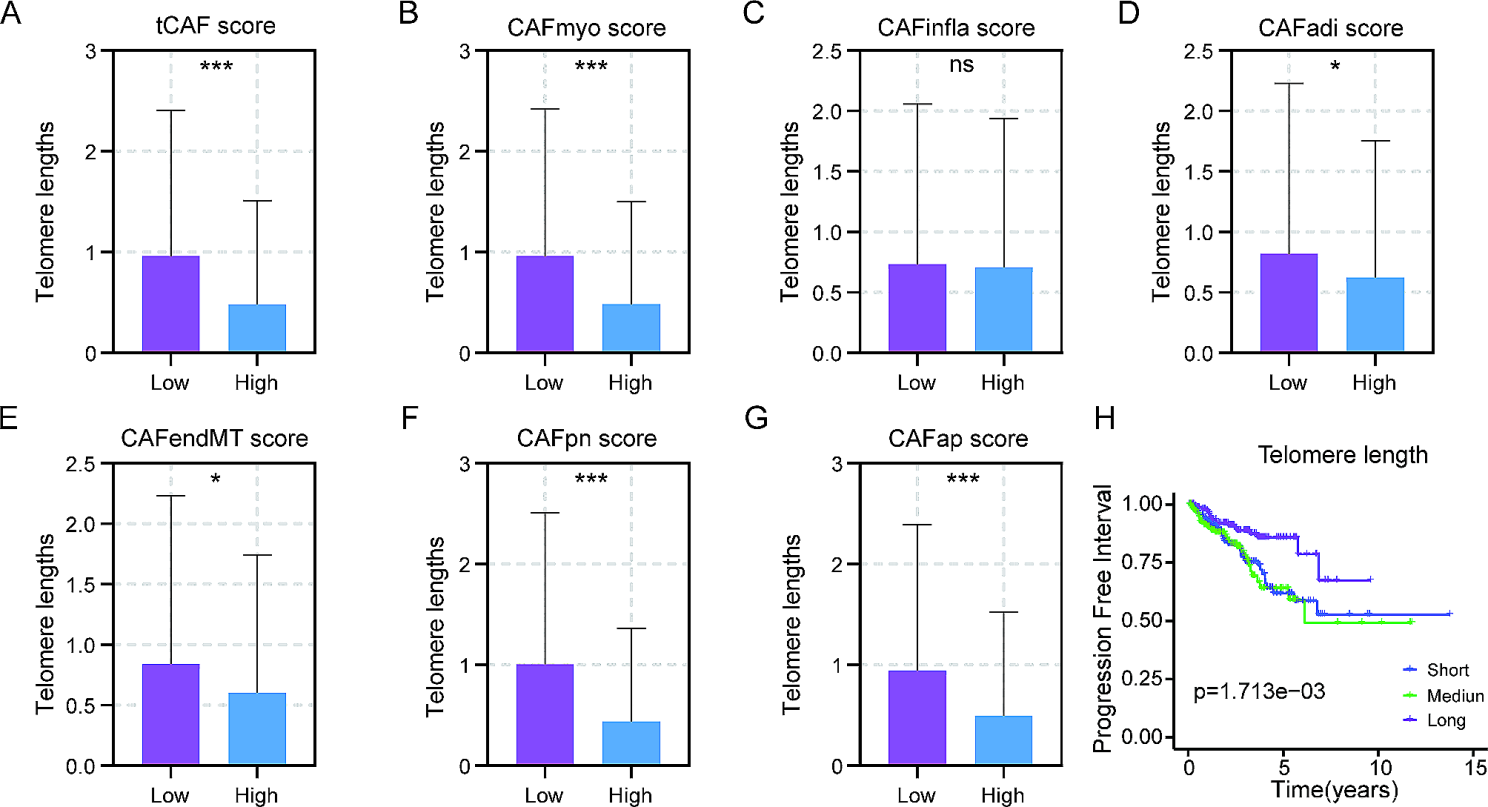
The relationship between telomere length and tCAF scores in PCa tumors. (A-G) The telomeres length in different CAF score subtypes. (H) Kaplan-Meier analysis of the telomere length in the TCGA PCa cohort

Telomere shortening or dysfunction occurs with aging, which drives inflammation [26]. We thus sought to determine whether increased immune activity observed in the tCAF score high tumors above was associated with altered telomere length. To this end, the TCGA PCa cohort was analyzed [25]. As shown in Fig. 7A and G, PCa tumors with high tCAF score had dramatically shorter telomeres compared to low score tumors. Moreover, the worst PFI was observed in patients with shortest telomere-bearing tumors (Fig. 7H).

#### The tCAF score system as a prognostic factor in pan-cancer

**Fig. 8:**
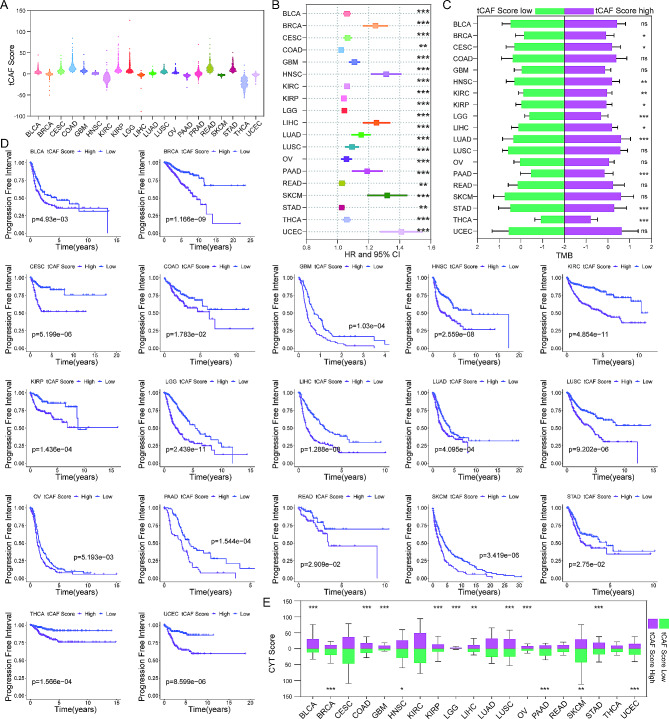
The tCAF score model in pan-cancer. (A) The CAF score in solid tumor. (B) Univariate Cox regression analysis of CAF score in pan-cancer. (C) The TMB of CAF score low and high groups in pan-cancer. (D) Kaplan-Meier analysis of the CAF score low and high groups in pan-cancer. (E) The CYT score of CAF score low and high groups in pan-cancer

Because Luo et al. showed similarity in CAF heterogeneity and transcriptomic profile across cancer types, we determined whether our tCAF score system could predict PFI and immune status in other solid tumors by analyzing the TCGA pan-cancer. We established separate tCAF scoring models for all solid tumors to improve the accuracy of CAF scoring model adaptation. Individual tCAF scores were established by performing univariate Cox regression analyses in each solid tumor (Fig. 8A and B). tCAF scores predicted PFI in pan-cancer (*p* < 0.05) (Fig. 8B). MK plots further showed significantly shorter PFI in those tumors with high tCAF score (Fig. 8D). TMB score and CYT score were analyzed simutaneously. According to the tCAF scores, TMB showed significant differences in BRCA, CESC, HNSC, KIRC, KIRP, LGG, LIHC, LUAD, PAAD, STAD and THCA (Fig. 8C). CYT score was more active in BLCA, COAD, GBM, KIRP, LGG, LIHC, LUSC, OV and STAD with tCAF score high tumors, while in BRCA, HNSC, PAAD, SKCM and UCEC with tCAF score low tumors (Fig. 8E).

## Discussion

The application of the scRNA-seq technology has substantially contributed to the reliable identification of CAF subtypes. CAFs are heterogeneous cell populations in the tumor TME and have been molecularly classified into 6 subtypes including CAFmyo, CAFinfla, CAFadi, CAFendMT, CAFpn and CAFap across solid tumors by Luo et al. [22]. In the present study, we explored the possibility of constructing a CAF-related score model to predict tumor progression and immunosuppressive microenvironment in high-throughput bulk sequencing of tumor tissues mixed with CAFs based on the scRNA-seq analysis of PCa tumors. Our results demonstrated that the scores for each of 6 CAF subtypes and the overall tCAF score were useful for prediction of patient outcomes. This score model can be further extended to other solid tumors, suggesting its broad implications in cancer clinics.

In cancer immunity, activation signals stimulate functional phenotypic transformation and accelerate proliferation of cytotoxic immune cells, thereby enhancing their ability to kill cancer cells. CAFs are the main cellular component of TME [27], however, CAFs in different tumor tissues have different molecular and functional characteristics, and even CAFs isolated from the same tissue may have different biological properties. CAFs interact directly or indirectly with the immune cells in the TME and can affect the active state of the immune cells [10, 16]. For example, CAFs are involved in regulating myeloid-derived suppressor cell (MDSC) infiltration and activation by secreting CXCL12, IL-6, VEGF, and CCL2 [28, 29]. CAFs can induce the polarization of macrophages to M2 type by secreting M-CSF [30]. In addition, CAF can affect T cell differentiation, function, or infiltration through a variety of pathways [10, 16, 31–33]. Thus, it is of great significance to explore the immune status and immune cell infiltration in PCa based on CAF score for screening patients suitable for immunotherapy. Our results showed that the patients with high CAF score had a more active immune state. However, tumor cells also achieve immune escape by expressing inhibitory ligands. In addition, the predicted CYT score was significantly higher in the CAF score high group. This also provides a basis for the effectiveness of immunotherapy. Taken together, patients with high tCAF score-bearing tumors would benefit from anti-CTLA4 immunotherapy.

Interestingly, in a colon cancer mouse model, the immune-suppressive effect of TGF-β1 has been shown to be involved in repression of CXCL9 and CXCL10 expression in CAFs, which subsequently inhibited the recruitment of effector T cell infiltration into tumors [34]. Mechanistically, TGF-β1 induces histone H3 lysine 27 trimethylation (H3K27me3) by recruiting histone demethylase EZH2 to the CXCL9 and 10 promoters, inhibiting their transcription. More recently, Sridaran et al. observed that oncogenic tyrosine kinase Activated CDC42 kinase 1 (ACK1) inhibited CXCL10 expression via the EZH2/H3K27 methylation-dependent manner and consequently reduced CD8 T cell infiltration in PCa tumors [7, 35]. ACK1 also directly constrains T cell activation [7, 35]. These findings raise the question of whether ACK1, like TGF-β1, exerts the same effect in CAFs. If so, ACK1 induces immune suppression via multiple pathways, including tumor-intrinsic and extrinsic mechanisms, and targeting ACK1 is expected to boost anti-tumor immunity.

In recent years, TMB has been considered as a potential indicator for tumor immunotherapy [36, 37]. The detection and recognition of neoantigens by T cells is an important link in predicting the efficacy of immunotherapy [38]. When the number of somatic mutations increases, more neoantigens are produced and more likely to be recognized by T cells [39]. It has been confirmed that high TMB is significantly associated with improved prognosis in cancer patients treated with ICIs [40]. In a pooled analysis of 27 tumors, TMB was associated with response to anti-PD-1 therapy [41]. In PCa, we found a positive correlation between TMB and tCAF, which further indicates that immunotherapy may benefit the tCAF score high PCa. However, the present findings should not be over-interpreted before they are confirmed experimentally and clinically.

It is well established that telomeres become progressively short with cellular proliferation or increased age, and shortened telomeres trigger aging at both cellular and organ levels, inducing chronic inflammation [26, 42, 43]. Interestingly, we observed significantly shorter telomeres in PCa tumors with a high tCAF score. It is currently unclear whether there is a causal relationship between shorter telomeres and CAF property, or whether shorter telomeres promote oncogenic function of CAFs. These issues call for further studies. Nevertheless, the presence of shortest telomeres in PCa tumors predicts the worst PFI, which is consistent with the tCAF high score tumors and has clinical implications.

Our study has limitations. First, we are unable to ascertain whether the relationship of CAF subtypes with aggressive phenotypes or outcomes are causal in PCa. Second, the association between the CAF score and sensitivity to ICIs are only based on the evaluation of transcriptomic data from tumors in PCa patients without receiving ICI therapy. Much more experimental and clinical investigations are required to solve these issues.

In conclusion, we have developed a novel PCa CAF score system based on CAF associated genes. This score model exhibits its value in assessing patient disease progression and tumor immune microenvironment.

### Electronic supplementary material

Below is the link to the electronic supplementary material.

Supplementary Material 1

## Data Availability

All sequencing data and related analyses in this article can be downloaded from the corresponding online database. TCGA: https://portal.gdc.cancer.gov/; GEO (GSE85606, GSE68164 and GSE70770): https://www.ncbi.nlm.nih.gov/geo/; MSKCC: http://cbio.mskcc.org/cancergenomics/prostate/data; CPGEA: http://www.cpgea.com/; TCIA: (https://tcia.at/home); TIP: http://biocc.hrbmu.edu.cn/TIP/.

## Abbreviations

ADT: Androgen deprivation therapy
AR: Androgen receptor
CAF: Cancer-associated fibroblast
CAFadi: Adipogenic CAFs
CAFap: Antigen-presenting CAF
CAFendMT: Endothelial-to-mesenchymal transition CAF
CAFinfla: inflammatory CAFs
CAFmyo: Cancer-associated myofibroblasts
CAFpn: Peripheralnerve-like CAF
CRPC: Castration-resistant prostate cancer
ECM: Extracellular matrix
GSEA: Gene set enrichment analysis
GSVA: Gene set variation analysis
IPS: Immunophenoscore
PCa: Prostate cancer
MDSC: Myeloid-derived suppressor cell
PFI: Progression-free interval
scRNA-seq: Single-cell RNA sequencing
TCIA: Cancer immune group atlas
TIP: Tracking tumor immunotype
TPM: Transcripts per million
TME: Tumor microenvironment
