# Supplementary material for: Comprehensive analyses of the cancer-associated fibroblast subtypes and their score system for prediction of outcomes and immunosuppressive microenvironment in prostate cancer
Source: Cancer Cell Int. 2024 Apr 5;24:127. doi: 10.1186/s12935-024-03305-5 (PMC10996219; doi:10.1186/s12935-024-03305-5)
Supplement: Supplementary file 1 — Supplementary Material 1 [file 12935_2024_3305_MOESM1_ESM.pdf]

Comprehensive analyses of the cancer-associated fibroblast subtypes and their score system for prediction of outcomes and immunosuppressive microenvironment in prostate cancer

Ze Gao[1,2#], Ning Zhang[3#], Bingzheng An[1], Dawei Li[1,2], Zhiqing Fang[1,2*], Dawei Xu[4*]

Supplementary materials include Figure S1- S6 and Table S1 – S4.

Figure S1. Distribution and expression levels of CAF associated genes.

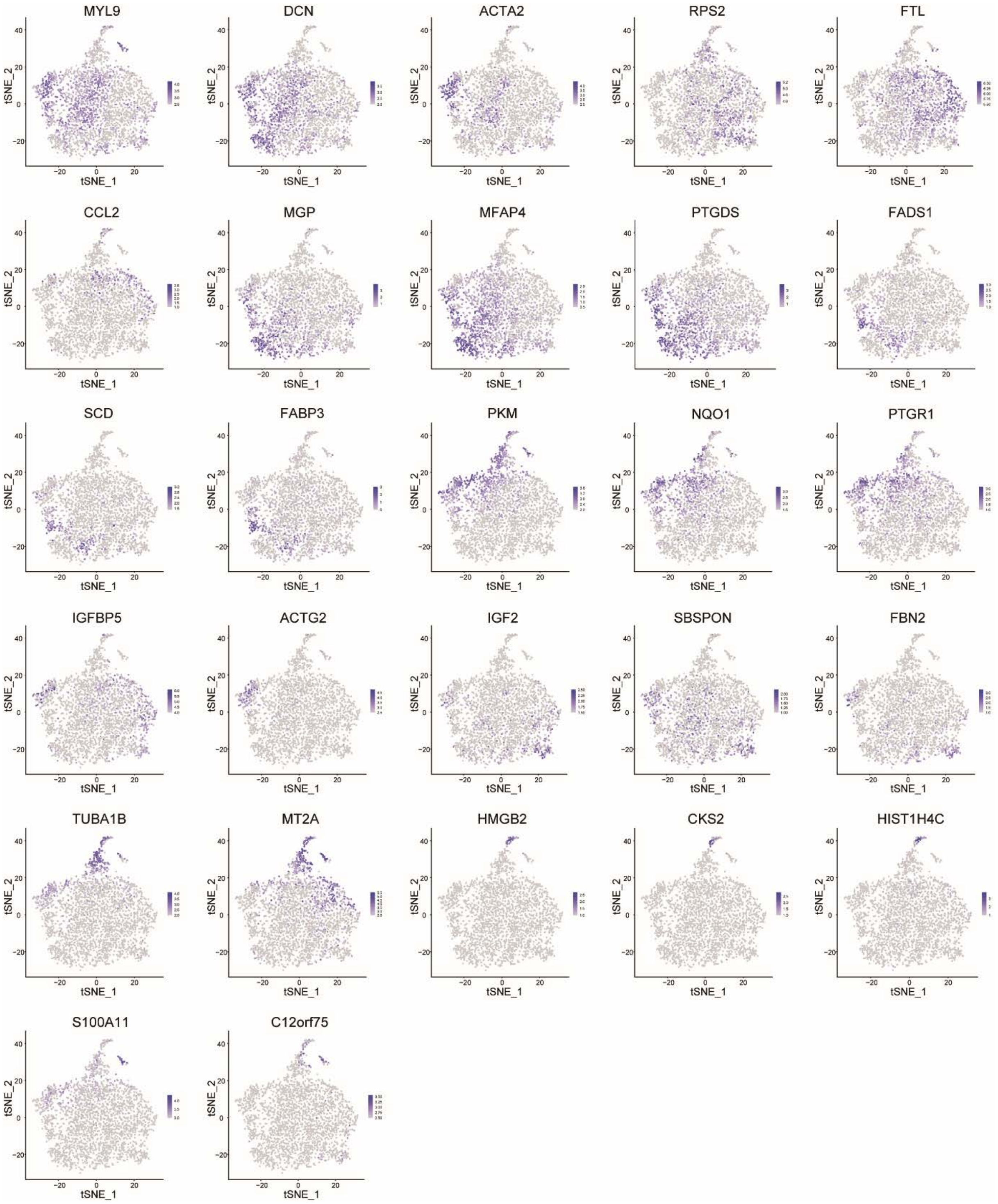

Figure S2. Establishment of CAF subtype score model. (A) Univariate Cox regression analysis of CAF associated genes in different subtypes. (B) ROC curves of CAF subtypes score at 3, 5, and 7 years in the TCGA dataset.

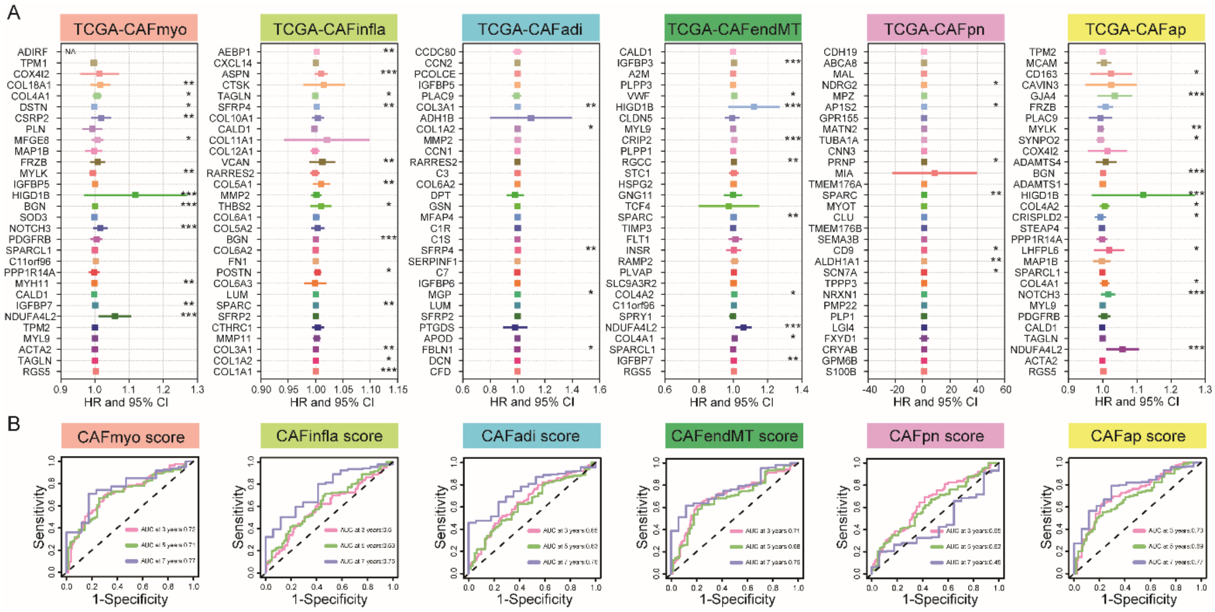

Figure S3. The interactions between genes. (A) The Sankey diagram of the relationship between CAF associated genes and CAF subtype. (B) The interaction between CAF associated genes in PCa tumors.

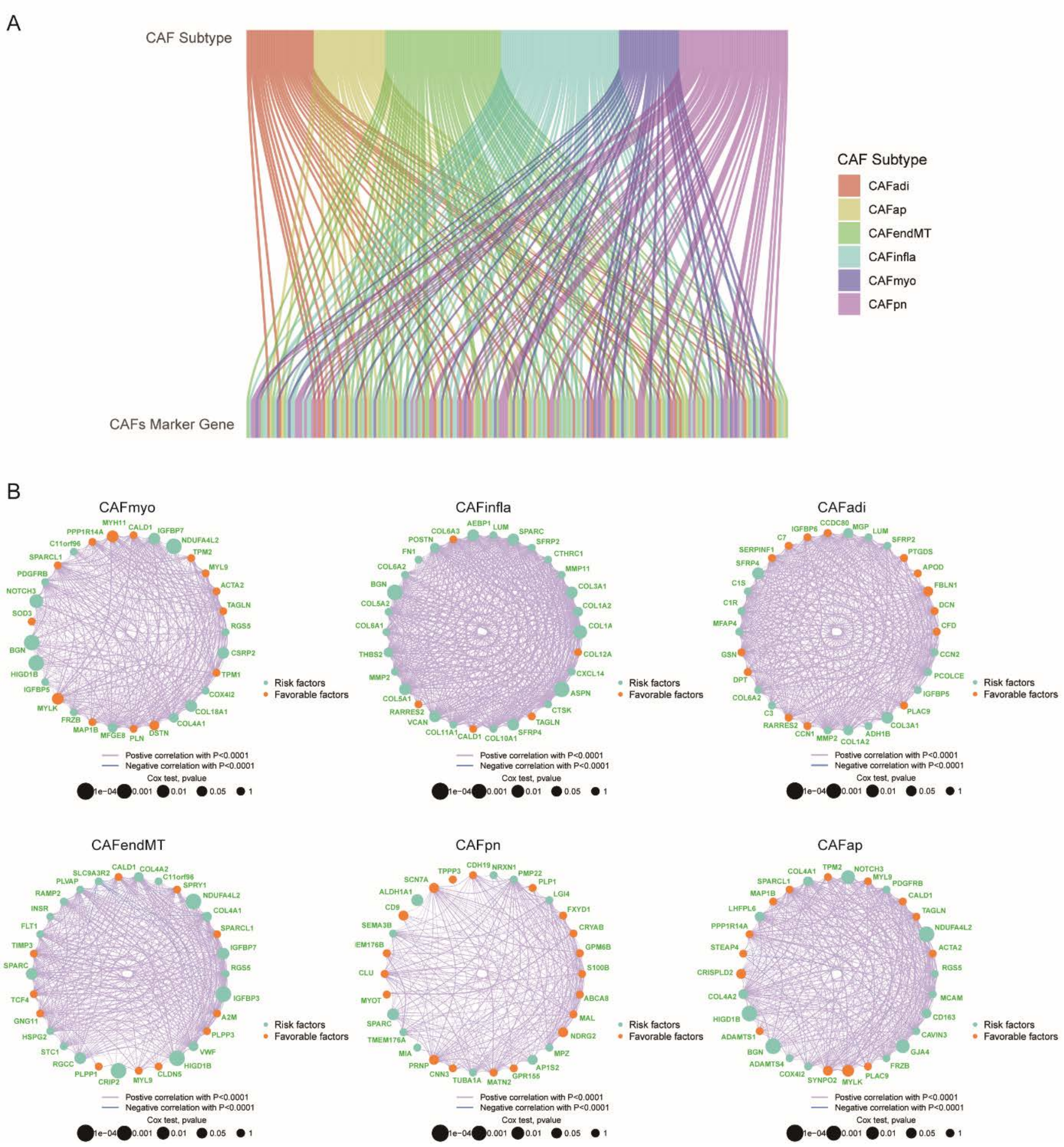

Figure S4. The box plot of treatment sensitivity of drugs between CAF score low and high groups.

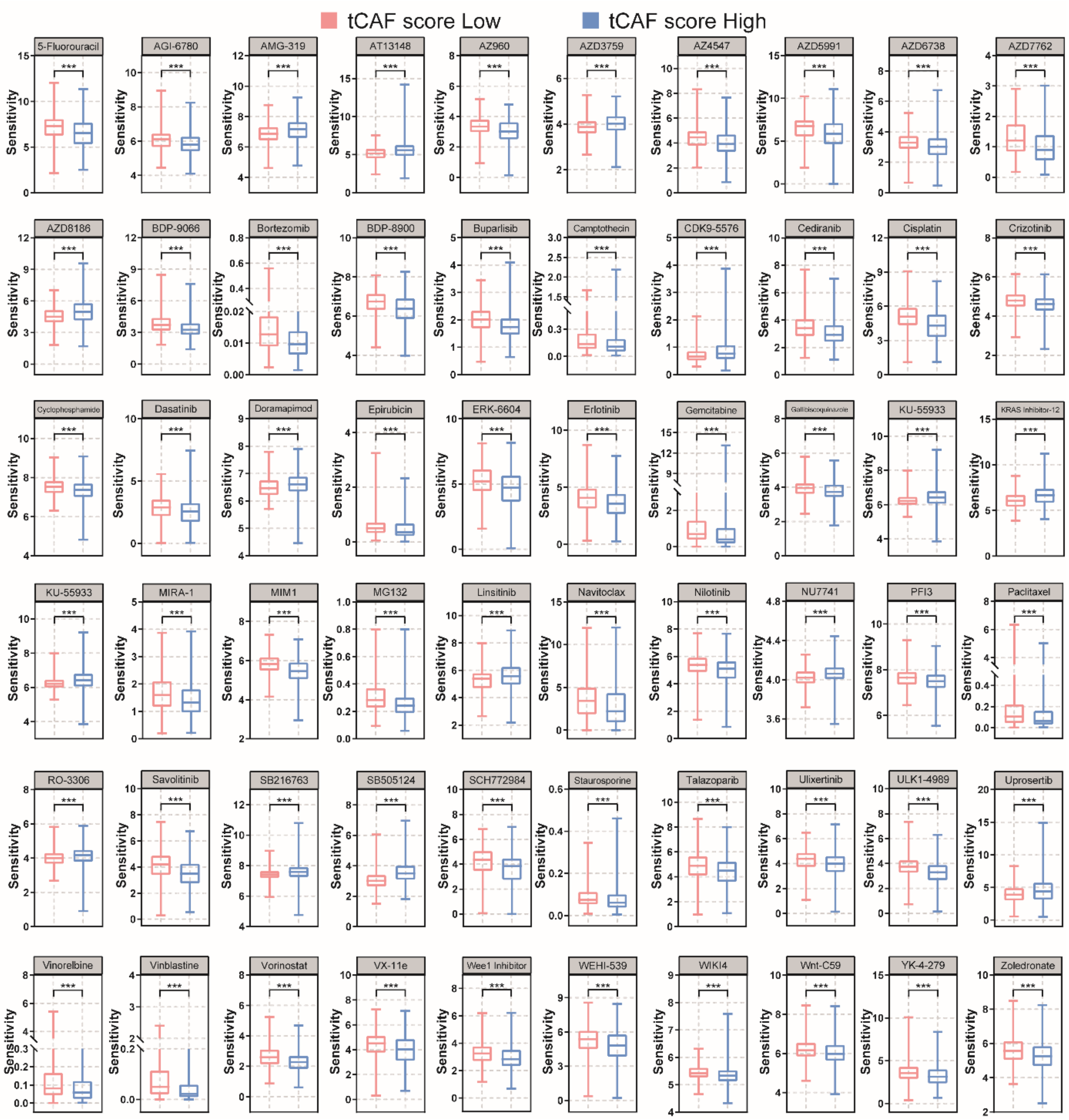

Figure S5 The CNV variation frequency of CAF associated genes in the TCGA PCa cohort.

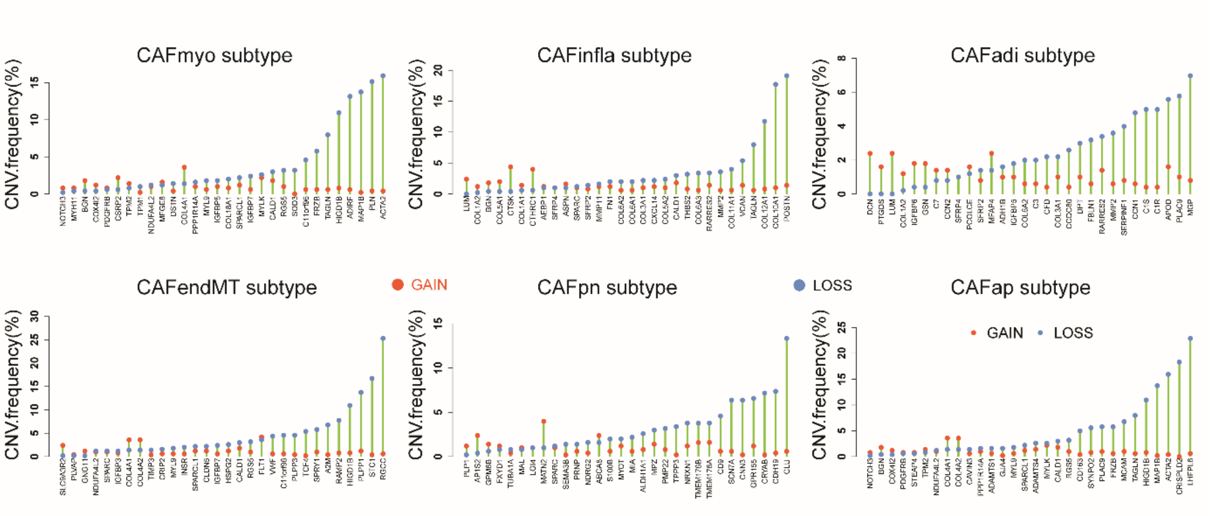

Figure S6. The GSE70770 PCa cohort analysis of the subtype and total CAF scores as prognostic factors. (A) Kaplan-Meier analysis was performed in the GSE70770 cohort based on the CAF subtype score-high and low groups (Median value as a cutoff). (B) Kaplan-Meier analysis of the total CAF score low and high groups (Median value as a cutoff). (C) ROC curves of CAF score at 3, 5, and 7 years in the GSE70770 PCa cohort.

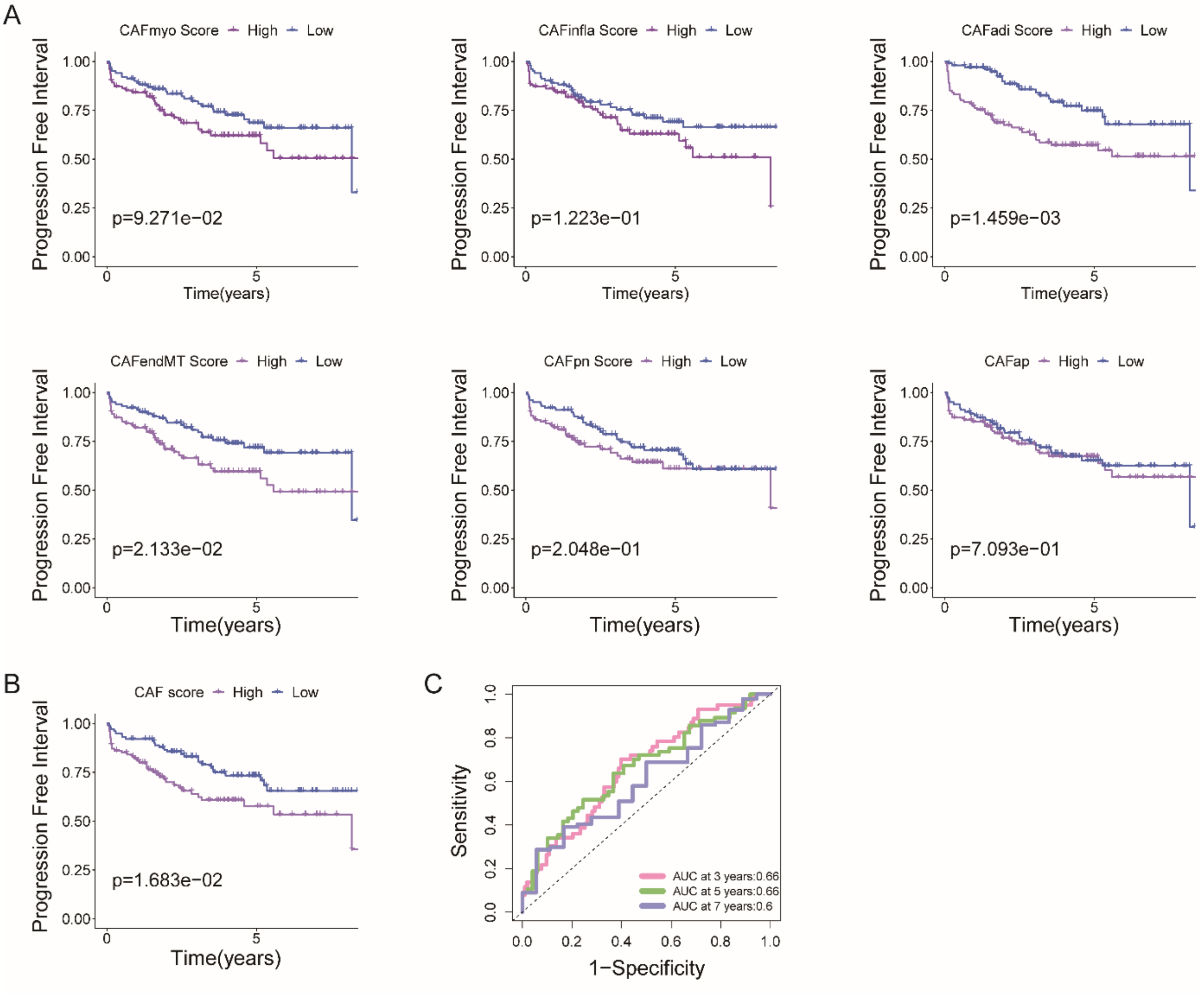

Table S1. Differentially expressed genes between normal and cancer-associated fibroblasts in the GSE85606 cohort of PCa

id	logFC	logCPM	PValue	FDR
GUCY1A1	7,885493	3,753533	5,77E-11	1,23E-06
CXADR	-5,6815	2,47182	3,83E-08	0,000409
DSP	6,454763	5,158253	1,49E-07	0,000843
MFAP5	5,606379	2,256999	1,58E-07	0,000843
CDH6	2,543334	8,409941	2,13E-07	0,000911
GUCY1B1	4,086999	4,715483	3,33E-07	0,001183
HOXD8	5,488266	0,847565	5,67E-07	0,001729
ATP10A	4,370887	3,004412	1,51E-06	0,004025
IP6K3	-7,40106	-0,80844	2,26E-06	0,004882
AC104083.1	7,025483	1,617654	2,29E-06	0,004882
EFHD1	9,502561	1,088124	3,52E-06	0,006832
TINAGL1	5,03803	4,305257	5,26E-06	0,009358
SORBS2	5,371859	2,053699	6,71E-06	0,011024
WT1	7,371468	-0,85531	7,75E-06	0,011817
KCNH1	4,482869	1,772273	1,05E-05	0,014983
ALPL	2,932214	3,858573	1,19E-05	0,015922
NRXN3	5,253206	2,656047	1,27E-05	0,015934
PTHLH	3,806804	4,196686	1,57E-05	0,017788
MYO1D	2,913677	5,238364	1,58E-05	0,017788
COL4A6	-2,60147	6,275737	2,08E-05	0,021255
COMP	6,647635	2,661221	2,09E-05	0,021255
LMCD1	3,884528	3,933095	2,30E-05	0,021386
SCRG1	6,423207	2,017926	2,30E-05	0,021386
FAT1	1,950638	9,602256	2,41E-05	0,021434
ITGA11	3,415483	6,427306	2,63E-05	0,022454
SLC9A7P1	3,342623	0,332459	3,19E-05	0,025964
PI16	5,688627	1,375801	3,28E-05	0,025964
TBX18	2,995038	2,139452	3,71E-05	0,028284
VIT	2,661941	3,351742	4,02E-05	0,029578
HOXD-AS2	6,385873	-1,64017	4,87E-05	0,034674
LRRC32	3,431237	4,230473	5,58E-05	0,036816
IGFBP2	2,426366	6,131182	5,62E-05	0,036816
BMP5	-4,29845	2,251389	5,96E-05	0,036816
CORIN	3,879725	2,66953	6,02E-05	0,036816
EPHB6	-3,75112	3,474429	6,06E-05	0,036816
ABCB4	3,47886	1,554	6,34E-05	0,036816
CPXM2	4,134199	0,996904	6,38E-05	0,036816
AL590004.3	3,968475	0,74743	6,88E-05	0,03868
GLDN	-2,51317	5,804362	8,12E-05	0,044478
NKD2	-3,10426	1,538811	9,09E-05	0,048326
PRR5L	3,031372	2,007677	9,30E-05	0,048326
KRT7	3,755878	3,05417	9,51E-05	0,048326
C1QTNF1	-1,69415	4,968964	0,000101	0,049984

Table S2. Differentially expressed genes between normal and cancer-associated fibroblasts in the GSE 68164 cohort of PCa

ID	adj.P.Val	P.Value	t	B	logFC	GI	Gene.symbol
ILMN_2060086	0,999	0,024616	-2,77414	-3,68	-1,58857	4,99E+08	ADAM23
ILMN_1681886	0,999	0,002087	4,506955	-2,95	1,147516	1E+09	ADAMTS5
ILMN_1657111	0,999	0,011107	-3,30381	-3,41	-1,84974	1,57E+08	AHNAK2
ILMN_2086890	0,999	0,001079	5,028668	-2,8	1,135729	9,31E+08	ANGPT1
ILMN_1671263	0,999	0,00061	-5,50681	-2,69	-1,5708	53832008	CACNA1H
ILMN_1712532	0,999	0,043877	2,398604	-3,89	1,422036	1,96E+08	CARD9
ILMN_1669982	0,999	0,001153	4,974831	-2,81	1,167262	1,23E+08	CCDC85A
ILMN_2396444	0,999	0,012307	3,23436	-3,44	1,101351	2,92E+08	CD14
ILMN_1719433	0,999	0,014871	3,107267	-3,51	1,215376	1,11E+08	CD1D
ILMN_1678493	0,999	0,012119	3,244749	-3,44	1,327039	3,31E+08	CHN1
ILMN_1691339	0,999	0,00378	4,061966	-3,1	1,216423	6,63E+08	CLEC1A
ILMN_1741688	0,999	0,001374	-4,83423	-2,85	-2,85627	2,23E+08	CPXM2
ILMN_1694432	0,999	0,004713	-3,90202	-3,16	-1,82269	3,99E+08	CRIP2
ILMN_1758128	0,999	0,000759	5,320457	-2,73	1,076072	3,74E+08	CYGB
ILMN_1703852	0,16	1,29E-05	-9,63402	-2,18	-2,01317	3,17E+08	EFNB2
ILMN_1697268	0,999	0,041913	2,428219	-3,87	1,071805	60498977	EMILIN2
ILMN_1781943	0,999	0,004076	-4,00692	-3,12	-1,09593	1,16E+08	FAM83D
ILMN_1769615	0,999	0,00254	-4,35746	-3	-1,88479	62241047	FLRT2
ILMN_1668052	0,999	0,017724	-2,99043	-3,56	-1,33731	1,94E+08	FOXA2
ILMN_1659678	0,409	6,6E-05	-7,66712	-2,35	-1,18762	1,67E+08	GABRA2
ILMN_2352090	0,999	0,035249	2,540343	-3,8	1,093972	40217832	GPRC5C
ILMN_1808590	0,999	0,003536	-4,11089	-3,08	-1,06321	3,74E+08	GUCY1A3
ILMN_1664861	0,999	0,039655	2,464056	-3,85	1,015809	3,42E+08	ID1
ILMN_1725193	0,999	0,040019	-2,45813	-3,85	-1,81465	55925575	IGFBP2
ILMN_1763390	0,409	4,96E-05	-7,98398	-2,31	-1,71747	1,15E+08	ISL1
ILMN_1669404	0,999	0,039364	2,468823	-3,85	1,076971	1,17E+08	KISS1
ILMN_1730777	0,999	0,004582	-3,92229	-3,15	-1,94863	1,31E+08	KRT19
ILMN_2163723	0,999	0,021092	2,875547	-3,62	1,259429	67782364	KRT7
ILMN_2063168	0,999	0,037123	-2,50676	-3,82	-1,45714	2,12E+08	MALL
ILMN_1672660	0,999	0,007777	-3,54843	-3,3	-1,63993	68509937	MBP
ILMN_1660462	0,999	0,04312	2,409864	-3,88	1,110164	7,33E+08	MCOLN2
ILMN_1910180	0,999	0,009209	-3,43174	-3,35	-1,00908	10437021	NRP2
ILMN_2189371	0,999	0,034492	-2,55442	-3,8	-1,5677	9,18E+08	NTNG1
ILMN_3236858	0,999	0,000691	5,400602	-2,71	1,211205	1,27E+08	NYNRIN
ILMN_1804929	0,999	0,044668	2,387068	-3,89	1,122145	32307151	OXTR
ILMN_1721770	0,999	0,008131	3,51756	-3,32	1,299356	9,67E+08	PAPPA
ILMN_1790778	0,999	0,000899	5,179119	-2,76	1,607315	1,57E+08	PNMA2
ILMN_2196328	0,999	0,040766	-2,44617	-3,86	-1,22614	2,1E+08	POSTN
ILMN_1660732	0,999	0,007337	-3,58887	-3,28	-2,51055	1,88E+08	PPP2R2B
ILMN_1655077	0,999	0,008394	3,495589	-3,33	1,137711	1,72E+08	PRDM1
ILMN_1798000	0,999	0,007631	3,561541	-3,3	1,039068	21361391	PSG1
ILMN_1685312	0,999	0,036693	2,514311	-3,82	1,68089	1,09E+08	PSG3

ILMN_1693397	0,999	0,003285	4,165237	-3,06	1,106646	4,52E+08	PSG4
ILMN_1728734	0,999	0,042593	2,417809	-3,87	2,14674	1,94E+08	PSG5
ILMN_1718514	0,999	0,027406	2,703995	-3,71	1,494331	3,95E+08	PSG6
ILMN_1772768	0,999	0,000794	5,282746	-2,74	1,283698	1,58E+08	PSG7
ILMN_1801776	0,999	0,049535	2,320237	-3,93	1,404139	6,84E+08	PSG9
ILMN_1806403	0,999	0,012951	-3,19999	-3,46	-1,31238	8,08E+08	RASL12
ILMN_1808238	0,999	0,018297	-2,96937	-3,57	-1,22083	34915989	RBPMS2
ILMN_1688630	0,999	0,001388	4,825984	-2,85	1,033403	2,07E+08	RECK
ILMN_1758067	0,999	0,00679	-3,64305	-3,26	-1,02477	1,65E+08	RGS4
ILMN_1805561	0,107	3,69E-06	-11,4142	-2,1	-2,25699	3,32E+08	SLC14A1
ILMN_1766261	0,999	0,026804	2,718474	-3,71	1,120744	93277101	SLC2A12
ILMN_1774127	0,999	0,001831	-4,6082	-2,92	-1,11177	6,36E+08	STAC
ILMN_3244117	0,999	0,004409	-3,95001	-3,14	-1,26504	4,44E+08	STMN3
ILMN_1667460	0,999	0,013566	3,168784	-3,48	1,003161	2,4E+08	SULF2
ILMN_2374115	0,999	0,004594	3,920358	-3,15	1,177922	1,09E+08	TFAP2A
ILMN_1701461	0,999	0,00368	4,081557	-3,09	1,506383	75905820	TIMP3
ILMN_1807169	0,999	0,031782	-2,60755	-3,77	-1,16845	2,1E+08	TINAGL1
ILMN_1730645	0,999	0,020355	-2,89896	-3,61	-2,06354	7,62E+08	TMEFF2
ILMN_1791511	0,999	0,044484	2,389724	-3,89	2,095423	32484986	TMEM176A
ILMN_2085012	0,999	0,029643	2,652847	-3,74	1,897876	1,56E+08	TMEM176B
ILMN_1764769	0,999	0,025161	2,759809	-3,68	1,119898	47498550	VWA5A

Table S3. Differentially expressed genes in 11 clusters of cAFs identified by single-cell RNA seq in PCa

ID	p_val	avg_log2FC	pct.1	pct.2	p_val_adj	cluster	gene
IFITM3	3,16E-68	0,392606	1	1	6,04E-64	0	IFITM3
MYL9	5,48E-67	0,433935	1	1	1,05E-62	0	MYL9
MYL6	3,17E-52	0,344065	1	1	6,06E-48	0	MYL6
B2M	3,17E-44	0,307425	1	1	6,06E-40	0	B2M
FHL2	2,24E-43	0,352943	1	0,996	4,29E-39	0	FHL2
DCN	1,02E-42	0,49688	1	0,999	1,95E-38	0	DCN
ACTA2	1,23E-40	0,611423	1	0,998	2,36E-36	0	ACTA2
ACTG2	9,29E-40	0,308491	1	1	1,78E-35	0	ACTG2
HSPB1	3,46E-34	0,268679	1	1	6,62E-30	0	HSPB1
MYLK	9,41E-32	0,393896	1	0,999	1,80E-27	0	MYLK
PDLIM1	1,16E-30	0,268265	1	0,998	2,23E-26	0	PDLIM1
MFAP4	4,36E-30	0,274973	0,993	0,906	8,33E-26	0	MFAP4
IFITM2	5,42E-30	0,301119	0,998	0,996	1,04E-25	0	IFITM2
HLA-B	8,58E-30	0,269333	1	0,998	1,64E-25	0	HLA-B
RARRES3	1,22E-28	0,298006	0,995	0,944	2,33E-24	0	RARRES3
COL3A1	6,32E-28	0,291715	1	0,992	1,21E-23	0	COL3A1
FBLN1	2,16E-27	0,260629	1	0,99	4,14E-23	0	FBLN1
CFD	9,17E-26	0,351769	0,979	0,946	1,75E-21	0	CFD
TAGLN	1,73E-22	0,298714	1	1	3,31E-18	0	TAGLN
CSRP1	2,67E-22	0,25575	0,998	0,984	5,11E-18	0	CSRP1
RARRES2	2,11E-21	0,404552	0,993	0,984	4,03E-17	0	RARRES2
RBP1	3,31E-21	0,301176	0,917	0,843	6,33E-17	0	RBP1
FHL1	3,47E-20	0,300647	0,924	0,875	6,64E-16	0	FHL1
CXCL1	5,88E-17	0,39304	0,768	0,647	1,12E-12	0	CXCL1
RPL7A	9,98E-78	0,277903	1	1	1,91E-73	1	RPL7A
RPS3	3,27E-75	0,279544	1	1	6,25E-71	1	RPS3
RPS12	6,84E-72	0,262031	1	1	1,31E-67	1	RPS12
RPS18	3,26E-70	0,273394	1	1	6,23E-66	1	RPS18
RPL3	3,41E-70	0,283834	1	1	6,53E-66	1	RPL3
RPL10A	1,18E-67	0,263645	1	1	2,27E-63	1	RPL10A
RPL12	3,02E-62	0,27153	1	1	5,78E-58	1	RPL12
RPL8	3,22E-62	0,261363	1	1	6,15E-58	1	RPL8
GNB2L1	3,91E-60	0,258433	1	1	7,49E-56	1	GNB2L1
RPSA	3,19E-51	0,282035	1	1	6,10E-47	1	RPSA
RPS2	5,27E-51	0,335246	1	1	1,01E-46	1	RPS2
SLC25A6	3,14E-40	0,251551	1	1	6,00E-36	1	SLC25A6
HMGA1	6,70E-32	0,401292	0,995	0,965	1,28E-27	1	HMGA1
FTL	4,74E-29	0,290601	1	1	9,07E-25	1	FTL
SNHG8	7,22E-24	0,276102	0,997	0,967	1,38E-19	1	SNHG8
CD44	1,59E-22	0,250918	1	0,999	3,03E-18	1	CD44
METRN	1,18E-20	0,2555	0,971	0,884	2,25E-16	1	METRN
VIM	4,03E-19	0,290322	1	1	7,71E-15	1	VIM
GLRX	8,27E-83	0,978023	1	0,993	1,58E-78	2	GLRX
TNFRSF12A	1,92E-75	0,660891	1	0,952	3,67E-71	2	TNFRSF12A
PHLDA2	4,00E-74	0,738809	1	0,963	7,65E-70	2	PHLDA2
COTL1	1,46E-69	0,586709	1	0,977	2,80E-65	2	COTL1
TNFRSF11B	2,70E-68	0,689543	0,997	0,891	5,17E-64	2	TNFRSF11B
SEC61G	6,10E-65	0,440661	1	0,998	1,17E-60	2	SEC61G

CCL2	2,78E-64	1,294465	0,956	0,76	5,31E-60	2	CCL2
STC2	4,32E-61	0,62764	0,99	0,864	8,27E-57	2	STC2
TXNRD1	1,35E-57	0,493255	1	0,997	2,59E-53	2	TXNRD1
LINC00152	9,47E-57	0,498098	1	0,986	1,81E-52	2	LINC00152
PFN1	6,21E-56	0,393451	1	1	1,19E-51	2	PFN1
SRM	3,05E-55	0,441936	1	0,965	5,84E-51	2	SRM
GPRC5A	1,31E-54	0,523351	0,987	0,848	2,50E-50	2	GPRC5A
CALM2	1,25E-51	0,436592	1	1	2,40E-47	2	CALM2
ARPC2	2,54E-51	0,433479	1	0,999	4,85E-47	2	ARPC2
SH3BGRL3	2,25E-49	0,490949	1	0,997	4,30E-45	2	SH3BGRL3
GAS6	1,13E-47	0,636559	1	0,988	2,15E-43	2	GAS6
PDCD5	2,79E-45	0,361709	1	0,988	5,33E-41	2	PDCD5
KRT10	5,93E-45	0,375727	1	0,986	1,13E-40	2	KRT10
IGFBP3	1,14E-44	0,575862	0,965	0,754	2,18E-40	2	IGFBP3
MTDH	5,14E-44	0,320402	1	0,993	9,83E-40	2	MTDH
CYCS	2,56E-43	0,353552	0,997	0,963	4,90E-39	2	CYCS
MPC2	2,85E-42	0,430744	1	0,986	5,45E-38	2	MPC2
ANGPT1	3,14E-42	0,32051	0,863	0,505	6,01E-38	2	ANGPT1
DKK1	5,90E-42	0,502339	0,921	0,667	1,13E-37	2	DKK1
PRELID1	6,23E-42	0,328789	1	0,997	1,19E-37	2	PRELID1
UGCG	2,64E-41	0,36153	0,943	0,741	5,05E-37	2	UGCG
MIR4435-2	5,45E-41	0,387853	1	0,95	1,04E-36	2	MIR4435-2HG
CITED2	7,14E-41	0,472444	0,997	0,935	1,36E-36	2	CITED2
TIMM8B	1,18E-40	0,362908	1	0,974	2,27E-36	2	TIMM8B
ZFAND5	1,64E-40	0,438152	1	0,984	3,14E-36	2	ZFAND5
ASPH	2,12E-40	0,37288	1	0,985	4,05E-36	2	ASPH
NHP2	1,11E-39	0,351334	1	0,964	2,11E-35	2	NHP2
GTF2A2	1,75E-39	0,371394	0,997	0,951	3,36E-35	2	GTF2A2
USMG5	2,06E-39	0,294109	1	0,997	3,95E-35	2	USMG5
SMURF2	7,85E-39	0,40023	0,99	0,822	1,50E-34	2	SMURF2
PPP1R14B	8,10E-39	0,368883	1	0,961	1,55E-34	2	PPP1R14B
KRTAP1-5	1,16E-38	0,380978	0,632	0,304	2,23E-34	2	KRTAP1-5
RND3	2,21E-38	0,410015	1	0,997	4,23E-34	2	RND3
RAB32	8,09E-38	0,348956	1	0,99	1,55E-33	2	RAB32
MEST	1,90E-37	0,55913	0,956	0,818	3,64E-33	2	MEST
ATP5G1	2,04E-37	0,322577	1	0,967	3,90E-33	2	ATP5G1
BID	2,32E-37	0,255639	0,952	0,697	4,44E-33	2	BID
POMP	8,10E-37	0,289495	1	1	1,55E-32	2	POMP
HSBP1L1	8,66E-37	0,335808	0,99	0,96	1,66E-32	2	HSBP1L1
ODC1	2,30E-36	0,303942	0,959	0,75	4,39E-32	2	ODC1
OPN3	2,75E-36	0,439959	0,911	0,663	5,26E-32	2	OPN3
ARPC5	4,37E-36	0,334892	1	0,989	8,36E-32	2	ARPC5
SBF2-AS1	5,03E-36	0,474129	1	0,942	9,62E-32	2	SBF2-AS1
CAV2	6,66E-36	0,345913	0,99	0,912	1,27E-31	2	CAV2
TMEM14B	1,11E-35	0,343453	1	0,97	2,13E-31	2	TMEM14B
GCLM	1,24E-35	0,470739	0,994	0,962	2,36E-31	2	GCLM
TFAP2A	2,21E-35	0,271663	0,873	0,591	4,23E-31	2	TFAP2A
IGFBP5	1,42E-34	0,606282	1	1	2,72E-30	2	IGFBP5
SELM	1,65E-34	0,301328	1	1	3,15E-30	2	SELM
C20orf24	3,60E-34	0,313306	0,994	0,91	6,89E-30	2	C20orf24

DDA1	4,81E-34	0,278768	0,987	0,889	9,20E-30	2	DDA1
HEBP1	1,17E-33	0,311737	0,997	0,988	2,23E-29	2	HEBP1
DYNLL1	8,72E-33	0,346344	1	0,998	1,67E-28	2	DYNLL1
SLC20A2	9,26E-33	0,296216	0,87	0,626	1,77E-28	2	SLC20A2
DAB2	1,56E-32	0,435029	1	0,989	2,99E-28	2	DAB2
COX7A2	2,88E-32	0,259317	1	0,999	5,52E-28	2	COX7A2
FKBP1A	4,24E-32	0,280268	1	0,996	8,10E-28	2	FKBP1A
CYR61	4,78E-32	0,402971	1	0,962	9,15E-28	2	CYR61
F3	8,55E-32	0,386977	0,933	0,751	1,64E-27	2	F3
MLF2	1,10E-31	0,284498	1	0,981	2,10E-27	2	MLF2
UQCR10	1,95E-31	0,25923	1	0,995	3,73E-27	2	UQCR10
SRGN	2,04E-31	0,426426	0,851	0,62	3,90E-27	2	SRGN
CCND1	2,19E-31	0,436216	0,994	0,964	4,18E-27	2	CCND1
NME1	5,40E-31	0,276333	0,987	0,934	1,03E-26	2	NME1
EMC6	7,53E-31	0,259662	0,997	0,933	1,44E-26	2	EMC6
NEDD8	7,69E-31	0,253083	1	0,999	1,47E-26	2	NEDD8
CTNNAL1	9,60E-31	0,283867	0,946	0,731	1,84E-26	2	CTNNAL1
OSTC	1,08E-30	0,270946	1	0,994	2,06E-26	2	OSTC
KRT18	1,25E-30	0,554062	0,937	0,762	2,39E-26	2	KRT18
POLE4	3,48E-30	0,281912	1	0,973	6,65E-26	2	POLE4
SERP1	6,20E-30	0,259254	1	0,993	1,19E-25	2	SERP1
PFN2	7,62E-30	0,274176	0,997	0,971	1,46E-25	2	PFN2
RHEB	1,03E-29	0,267067	1	0,985	1,97E-25	2	RHEB
MYDGF	6,53E-29	0,337141	1	0,997	1,25E-24	2	MYDGF
EIF4EBP1	8,95E-29	0,383618	0,997	0,97	1,71E-24	2	EIF4EBP1
GNG11	1,58E-28	0,326109	1	0,998	3,02E-24	2	GNG11
SNU13	1,77E-28	0,250265	1	0,994	3,39E-24	2	SNU13
RRAS2	1,78E-28	0,260707	0,978	0,852	3,41E-24	2	RRAS2
ZYX	1,90E-28	0,296239	1	0,954	3,64E-24	2	ZYX
CAV1	3,86E-28	0,470401	1	0,999	7,37E-24	2	CAV1
CLTB	5,51E-28	0,295581	1	0,98	1,05E-23	2	CLTB
LHFP	5,95E-28	0,292393	0,99	0,949	1,14E-23	2	LHFP
C7orf73	8,57E-28	0,283989	1	0,98	1,64E-23	2	C7orf73
BDNF	2,13E-27	0,381871	0,952	0,821	4,06E-23	2	BDNF
BOLA3	2,38E-27	0,253215	0,984	0,86	4,56E-23	2	BOLA3
SLC16A3	2,83E-27	0,270053	0,99	0,903	5,41E-23	2	SLC16A3
PLAU	4,82E-27	0,442928	0,914	0,739	9,22E-23	2	PLAU
MKKS	9,59E-27	0,26133	0,99	0,923	1,83E-22	2	MKKS
RGCC	1,13E-26	0,436369	0,562	0,296	2,17E-22	2	RGCC
CFL1	1,34E-26	0,271387	1	1	2,57E-22	2	CFL1
SULF1	2,31E-26	0,361533	0,886	0,712	4,41E-22	2	SULF1
MRPL14	2,36E-26	0,284022	1	0,974	4,51E-22	2	MRPL14
GTF2H5	2,36E-26	0,270217	1	0,985	4,51E-22	2	GTF2H5
FTL1	2,52E-26	0,316463	1	1	4,81E-22	2	FTL
STAT1	5,40E-26	0,286431	0,99	0,946	1,03E-21	2	STAT1
SLC7A11	9,36E-26	0,30288	0,965	0,801	1,79E-21	2	SLC7A11
COX20	1,23E-25	0,265367	0,994	0,938	2,36E-21	2	COX20
ARHGAP29	1,61E-25	0,279883	0,978	0,854	3,08E-21	2	ARHGAP29
CCND3	3,94E-25	0,268895	0,876	0,685	7,54E-21	2	CCND3
RPL22L1	5,04E-25	0,35934	0,997	0,971	9,64E-21	2	RPL22L1

PELO	1,14E-24	0,26052	0,962	0,796	2,18E-20	2	PELO
P3H2	2,00E-24	0,2551	0,902	0,692	3,82E-20	2	P3H2
EXT1	3,64E-23	0,27804	0,997	0,931	6,96E-19	2	EXT1
CDC42EP1	3,77E-23	0,252279	0,956	0,844	7,21E-19	2	CDC42EP1
LMO7	2,69E-22	0,26719	1	0,943	5,14E-18	2	LMO7
GADD45A	3,13E-22	0,266934	0,949	0,802	5,99E-18	2	GADD45A
VASP	4,38E-22	0,261405	0,975	0,886	8,37E-18	2	VASP
POC1B	4,57E-21	0,257794	0,708	0,48	8,73E-17	2	POC1B
PAPSS2	3,66E-20	0,255195	0,994	0,914	7,00E-16	2	PAPSS2
OXTR	5,29E-20	0,266771	0,775	0,526	1,01E-15	2	OXTR
TUBB6	3,82E-19	0,251225	0,994	0,97	7,30E-15	2	TUBB6
CYB5A	3,92E-17	0,271549	1	0,988	7,50E-13	2	CYB5A
BRIX1	8,65E-17	0,293898	0,943	0,803	1,65E-12	2	BRIX1
CEMIP	4,43E-16	0,291396	0,933	0,752	8,48E-12	2	CEMIP
FST	2,06E-15	0,270673	0,829	0,647	3,94E-11	2	FST
HMGA11	7,82E-15	0,270412	0,997	0,966	1,50E-10	2	HMGA1
PDLIM7	8,64E-15	0,254576	1	0,995	1,65E-10	2	PDLIM7
CYP1B1	3,66E-14	0,292342	0,629	0,422	7,00E-10	2	CYP1B1
USP53	4,57E-14	0,279821	0,933	0,787	8,74E-10	2	USP53
MT1E	7,56E-14	0,291447	1	0,959	1,45E-09	2	MT1E
MT2A	3,48E-11	0,559309	1	0,999	6,66E-07	2	MT2A
ADM	9,60E-11	0,257971	0,987	0,945	1,84E-06	2	ADM
RHOBTB3	1,62E-10	0,250686	1	0,996	3,09E-06	2	RHOBTB3
TIMP3	1,63E-10	0,259367	0,854	0,719	3,11E-06	2	TIMP3
COL1A2	1,36E-89	0,678883	1	0,999	2,60E-85	3	COL1A2
COL14A1	4,64E-89	0,83895	0,981	0,824	8,88E-85	3	COL14A1
MGP	7,64E-86	1,808004	0,984	0,816	1,46E-81	3	MGP
MFAP41	8,00E-82	1,133897	0,971	0,912	1,53E-77	3	MFAP4
C1S	1,17E-78	0,807523	1	0,985	2,24E-74	3	C1S
NR2F1	3,05E-70	0,798167	0,994	0,972	5,83E-66	3	NR2F1
COL3A11	5,91E-67	0,7098	1	0,992	1,13E-62	3	COL3A1
PTGDS	4,27E-62	1,417567	0,984	0,891	8,17E-58	3	PTGDS
FBLN11	7,41E-62	0,822699	0,997	0,991	1,42E-57	3	FBLN1
NBL1	2,28E-60	0,646422	0,949	0,873	4,35E-56	3	NBL1
PBX1	7,56E-60	0,560555	0,949	0,836	1,45E-55	3	PBX1
MMP2	4,25E-59	0,643294	1	0,991	8,12E-55	3	MMP2
PNRC1	8,03E-59	0,567956	0,977	0,959	1,53E-54	3	PNRC1
MALAT1	1,71E-54	0,610234	1	1	3,28E-50	3	MALAT1
QSOX1	4,03E-54	0,602345	0,994	0,992	7,70E-50	3	QSOX1
LTBP4	5,46E-52	0,545063	0,952	0,932	1,04E-47	3	LTBP4
TGFBI	5,59E-51	0,614164	0,997	0,99	1,07E-46	3	TGFBI
PSAP	9,57E-51	0,56008	1	0,996	1,83E-46	3	PSAP
GRN	8,35E-50	0,545814	0,997	0,99	1,60E-45	3	GRN
PBX3	2,11E-49	0,52523	0,939	0,831	4,04E-45	3	PBX3
SOD3	2,70E-49	0,657406	0,952	0,872	5,15E-45	3	SOD3
SEPP1	3,52E-48	0,682333	0,897	0,751	6,72E-44	3	SEPP1
LRP1	6,78E-48	0,460415	0,977	0,962	1,30E-43	3	LRP1
COL6A2	1,02E-47	0,470055	1	0,998	1,95E-43	3	COL6A2
CXCL12	1,03E-46	0,484096	0,685	0,397	1,97E-42	3	CXCL12
NEAT1	1,27E-46	0,589768	1	0,999	2,42E-42	3	NEAT1

MLXIP	2,05E-46	0,515349	0,974	0,935	3,93E-42	3	MLXIP
SOX4	8,02E-46	0,572085	0,971	0,921	1,53E-41	3	SOX4
VCAN	2,35E-45	0,324404	0,463	0,168	4,49E-41	3	VCAN
REV3L	2,61E-45	0,478697	0,929	0,842	5,00E-41	3	REV3L
NPC2	3,46E-44	0,466211	1	0,999	6,62E-40	3	NPC2
FN1	3,79E-44	0,493986	1	0,996	7,26E-40	3	FN1
RBP11	1,34E-43	0,604036	0,932	0,845	2,56E-39	3	RBP1
CST3	2,96E-43	0,478197	1	0,999	5,65E-39	3	CST3
CCDC80	9,84E-43	0,722925	1	0,999	1,88E-38	3	CCDC80
SERPING1	2,80E-41	0,541943	0,878	0,761	5,34E-37	3	SERPING1
PLPP1	2,82E-41	0,535329	0,936	0,837	5,39E-37	3	PLPP1
PARM1	4,38E-41	0,33226	0,531	0,238	8,38E-37	3	PARM1
CDH11	3,82E-40	0,401969	0,926	0,861	7,30E-36	3	CDH11
PFDN5	8,34E-40	0,26147	1	1	1,59E-35	3	PFDN5
SMPDL3A	1,71E-39	0,505089	0,968	0,922	3,26E-35	3	SMPDL3A
RPLP1	4,96E-39	0,256246	1	1	9,49E-35	3	RPLP1
DCN1	2,35E-38	0,638747	1	0,999	4,50E-34	3	DCN
CCNI	8,17E-38	0,369096	0,997	0,998	1,56E-33	3	CCNI
N4BP2L2	1,01E-37	0,419697	0,994	0,972	1,93E-33	3	N4BP2L2
C12orf57	2,54E-37	0,384491	0,994	0,992	4,86E-33	3	C12orf57
C1R	4,01E-37	0,577083	0,997	0,974	7,68E-33	3	C1R
CTSK	8,45E-37	0,801252	0,955	0,899	1,62E-32	3	CTSK
MYO6	2,77E-36	0,420638	0,894	0,824	5,30E-32	3	MYO6
MEG3	3,07E-36	0,528272	0,997	0,97	5,87E-32	3	MEG3
PNISR	3,65E-36	0,425231	0,971	0,953	6,98E-32	3	PNISR
ZFP36L1	4,88E-36	0,454497	0,971	0,97	9,33E-32	3	ZFP36L1
FOXN3	6,10E-36	0,365056	0,794	0,645	1,17E-31	3	FOXN3
EIF3E	6,31E-36	0,356992	0,99	1	1,21E-31	3	EIF3E
H3F3A	1,09E-35	0,339565	1	1	2,09E-31	3	H3F3A
FMOD	2,21E-35	0,329802	0,55	0,284	4,22E-31	3	FMOD
COL8A1	2,57E-35	0,466924	0,736	0,541	4,92E-31	3	COL8A1
COL6A3	1,72E-34	0,355928	1	0,993	3,29E-30	3	COL6A3
GLTSCR2	2,77E-34	0,384322	0,997	0,994	5,29E-30	3	GLTSCR2
TIMP2	5,87E-34	0,379723	0,997	0,991	1,12E-29	3	TIMP2
BTG1	2,43E-33	0,487795	0,981	0,968	4,65E-29	3	BTG1
COL6A1	4,77E-33	0,388571	1	0,996	9,12E-29	3	COL6A1
HTRA1	5,09E-33	0,444338	0,839	0,71	9,74E-29	3	HTRA1
GPNMB	5,31E-33	0,521142	0,968	0,93	1,01E-28	3	GPNMB
PCBP2	1,82E-32	0,286489	0,997	0,997	3,48E-28	3	PCBP2
APP	2,65E-32	0,395713	0,981	0,978	5,07E-28	3	APP
SERPINF1	3,28E-32	0,633735	0,887	0,824	6,28E-28	3	SERPINF1
CTSF	1,30E-31	0,385833	0,717	0,528	2,49E-27	3	CTSF
PTN	1,39E-31	0,498563	0,994	0,994	2,65E-27	3	PTN
ANGPTL2	7,51E-31	0,391043	0,913	0,848	1,44E-26	3	ANGPTL2
ZFP36L2	2,92E-30	0,421414	0,981	0,966	5,58E-26	3	ZFP36L2
RGS10	3,02E-30	0,370733	0,971	0,984	5,77E-26	3	RGS10
CAMLG	6,81E-30	0,3598	0,965	0,959	1,30E-25	3	CAMLG
ADH1B	1,77E-29	0,369393	0,36	0,132	3,39E-25	3	ADH1B
HCFC1R1	2,14E-29	0,375383	0,936	0,904	4,09E-25	3	HCFC1R1
PTMA	2,69E-29	0,467047	0,997	1	5,15E-25	3	PTMA

NID1	3,28E-29	0,345812	0,904	0,851	6,26E-25	3	NID1
RAB13	4,49E-29	0,391955	0,997	0,996	8,59E-25	3	RAB13
CFD1	4,89E-29	0,703907	0,958	0,95	9,34E-25	3	CFD
MEIS2	4,96E-29	0,3534	0,965	0,949	9,49E-25	3	MEIS2
ITGB8	5,36E-29	0,250278	0,537	0,299	1,03E-24	3	ITGB8
EEF2	5,79E-29	0,2804	1	1	1,11E-24	3	EEF2
COL4A2	6,68E-29	0,358289	0,961	0,955	1,28E-24	3	COL4A2
SULF2	8,32E-29	0,306815	0,65	0,441	1,59E-24	3	SULF2
SH3BGRL	3,15E-28	0,348387	0,974	0,975	6,02E-24	3	SH3BGRL
RSPO1	4,74E-28	0,268878	0,595	0,361	9,06E-24	3	RSPO1
PAMR1	5,93E-28	0,364351	0,9	0,876	1,13E-23	3	PAMR1
ERP29	6,55E-28	0,319339	0,997	0,993	1,25E-23	3	ERP29
TXNIP	7,31E-28	0,419045	0,932	0,909	1,40E-23	3	TXNIP
C6orf48	8,95E-28	0,30629	0,984	0,996	1,71E-23	3	C6orf48
ITIH5	1,51E-27	0,411091	0,717	0,538	2,89E-23	3	ITIH5
EMILIN1	2,05E-27	0,394869	0,945	0,927	3,92E-23	3	EMILIN1
FOXP1	8,23E-27	0,314465	0,891	0,805	1,57E-22	3	FOXP1
NREP	1,86E-26	0,28903	0,64	0,471	3,56E-22	3	NREP
GBP2	2,04E-26	0,364721	0,9	0,837	3,90E-22	3	GBP2
LXN	2,34E-26	0,521327	0,965	0,936	4,47E-22	3	LXN
RARRES21	6,39E-26	0,545083	0,987	0,985	1,22E-21	3	RARRES2
ZMAT3	9,38E-26	0,383747	0,952	0,928	1,79E-21	3	ZMAT3
C11orf96	1,10E-25	0,269981	0,421	0,193	2,11E-21	3	C11orf96
MT-ND3	3,15E-25	0,261622	1	0,999	6,02E-21	3	MT-ND3
PCYOX1	5,81E-25	0,339238	0,923	0,89	1,11E-20	3	PCYOX1
JUNB	5,82E-25	0,481256	0,939	0,916	1,11E-20	3	JUNB
AKAP12	1,23E-24	0,332111	0,977	0,978	2,36E-20	3	AKAP12
ITGA11	3,01E-24	0,27723	0,662	0,507	5,76E-20	3	ITGA11
C1orf21	4,94E-24	0,30454	0,926	0,901	9,45E-20	3	C1orf21
NENF	4,97E-24	0,257407	0,99	0,993	9,51E-20	3	NENF
NFIA	5,01E-24	0,270973	0,685	0,519	9,59E-20	3	NFIA
CCPG1	6,90E-24	0,301742	0,804	0,684	1,32E-19	3	CCPG1
CYBRD1	7,42E-24	0,282989	0,952	0,945	1,42E-19	3	CYBRD1
BMPR2	9,03E-24	0,257256	0,807	0,727	1,73E-19	3	BMPR2
LINC01082	1,04E-23	0,372096	0,871	0,832	2,00E-19	3	LINC01082
JUN	1,61E-23	0,425777	0,897	0,855	3,07E-19	3	JUN
SNHG7	2,80E-23	0,370134	0,981	0,98	5,35E-19	3	SNHG7
FRMD6-AS:	2,90E-23	0,436518	0,624	0,451	5,54E-19	3	FRMD6-AS2
RBPMS	4,81E-23	0,268314	0,994	0,98	9,20E-19	3	RBPMS
SAT1	9,63E-23	0,400062	0,977	0,976	1,84E-18	3	SAT1
COL1A1	1,04E-22	0,292134	1	0,997	1,99E-18	3	COL1A1
EIF4B	1,51E-22	0,287791	0,994	0,994	2,88E-18	3	EIF4B
NR2F1-AS1	1,81E-22	0,275835	0,862	0,81	3,46E-18	3	NR2F1-AS1
B2M1	6,14E-22	0,25703	1	1	1,17E-17	3	B2M
PAPPA	8,22E-22	0,415087	0,842	0,766	1,57E-17	3	PAPPA
FBN1	8,34E-22	0,332772	0,894	0,877	1,59E-17	3	FBN1
FCGRT	1,17E-21	0,315912	0,971	0,965	2,24E-17	3	FCGRT
DDX17	1,26E-21	0,298422	0,977	0,967	2,40E-17	3	DDX17
FXYD1	1,54E-21	0,289727	0,582	0,413	2,94E-17	3	FXYD1
ZCCHC24	1,95E-21	0,251635	0,772	0,714	3,72E-17	3	ZCCHC24

GPX3	2,00E-21	0,460952	0,733	0,593	3,83E-17	3	GPX3
UBXN1	2,21E-21	0,323648	0,99	0,993	4,23E-17	3	UBXN1
PDGFRB	2,85E-21	0,333115	0,871	0,857	5,45E-17	3	PDGFRB
CIRBP	3,33E-21	0,286083	1	0,996	6,38E-17	3	CIRBP
WSB1	3,40E-21	0,402994	0,955	0,93	6,51E-17	3	WSB1
SPRY1	3,59E-21	0,293924	0,508	0,321	6,87E-17	3	SPRY1
NFIX	5,19E-21	0,286716	0,875	0,859	9,93E-17	3	NFIX
DDAH2	5,51E-21	0,291971	0,965	0,968	1,05E-16	3	DDAH2
COL18A1	1,51E-20	0,289229	0,897	0,855	2,89E-16	3	COL18A1
DDR2	1,94E-20	0,288119	0,955	0,958	3,70E-16	3	DDR2
RB1CC1	4,96E-20	0,25806	0,9	0,893	9,48E-16	3	RB1CC1
MMP14	1,02E-19	0,309606	0,977	0,95	1,96E-15	3	MMP14
RARRES31	2,14E-19	0,377529	0,968	0,95	4,09E-15	3	RARRES3
STAT3	2,20E-19	0,258703	0,939	0,924	4,20E-15	3	STAT3
DDX5	2,90E-19	0,306813	1	0,992	5,55E-15	3	DDX5
ISCU	3,27E-19	0,267769	0,987	0,985	6,24E-15	3	ISCU
MOXD1	4,28E-19	0,264776	0,772	0,69	8,18E-15	3	MOXD1
PDGFRA	7,58E-19	0,291815	0,894	0,893	1,45E-14	3	PDGFRA
THBS1	9,41E-19	0,318051	0,99	0,981	1,80E-14	3	THBS1
EPB41L4A-/	1,15E-18	0,269276	0,92	0,929	2,20E-14	3	EPB41L4A-AS1
SLC25A36	1,17E-18	0,255774	0,955	0,946	2,23E-14	3	SLC25A36
APOE	1,34E-18	0,510327	0,479	0,287	2,56E-14	3	APOE
TRIM56	1,46E-18	0,292118	0,875	0,871	2,79E-14	3	TRIM56
DST	1,71E-18	0,288533	0,99	0,99	3,28E-14	3	DST
OSR2	1,80E-18	0,373846	0,785	0,742	3,44E-14	3	OSR2
PCOLCE	2,14E-18	0,477543	0,99	0,984	4,09E-14	3	PCOLCE
FGF7	2,37E-18	0,345447	0,987	0,992	4,54E-14	3	FGF7
PPIC	3,06E-18	0,358745	0,939	0,945	5,85E-14	3	PPIC
IGBP1	4,89E-18	0,289353	0,958	0,957	9,34E-14	3	IGBP1
DHRS3	8,64E-18	0,299725	0,949	0,962	1,65E-13	3	DHRS3
ZNF503	1,18E-17	0,255658	0,723	0,63	2,27E-13	3	ZNF503
HSPB6	1,43E-17	0,295694	0,881	0,842	2,73E-13	3	HSPB6
PFDN4	2,07E-17	0,277609	0,955	0,966	3,96E-13	3	PFDN4
COL5A1	2,94E-17	0,261725	0,871	0,843	5,63E-13	3	COL5A1
EGR1	6,34E-17	0,301744	0,81	0,774	1,21E-12	3	EGR1
ISOC1	8,15E-17	0,31134	0,859	0,811	1,56E-12	3	ISOC1
RAB23	1,28E-16	0,256689	0,817	0,77	2,45E-12	3	RAB23
SEPW1	3,27E-16	0,253321	0,875	0,856	6,25E-12	3	SEPW1
LAMB1	8,77E-16	0,25429	0,891	0,885	1,68E-11	3	LAMB1
PLD3	9,94E-16	0,283441	0,974	0,98	1,90E-11	3	PLD3
THBS2	1,64E-15	0,2655	0,945	0,927	3,13E-11	3	THBS2
ADD3	2,22E-15	0,265426	0,801	0,785	4,24E-11	3	ADD3
MARCKSL1	2,63E-15	0,293566	0,891	0,883	5,04E-11	3	MARCKSL1
TBX3	3,18E-15	0,291109	0,887	0,889	6,08E-11	3	TBX3
07-sep	3,65E-15	0,281983	0,99	0,996	6,98E-11	3	07-sep
RARRES1	5,14E-15	0,287878	0,836	0,764	9,84E-11	3	RARRES1
LAMA4	1,25E-14	0,261049	0,907	0,924	2,39E-10	3	LAMA4
GABPB1-AS	1,60E-14	0,284615	0,804	0,772	3,07E-10	3	GABPB1-AS1
ITGAV	1,66E-14	0,275841	0,868	0,889	3,18E-10	3	ITGAV
LAMP2	6,73E-14	0,268359	0,945	0,938	1,29E-09	3	LAMP2

MMP23B	9,70E-14	0,26363	0,469	0,329	1,86E-09	3	MMP23B
ZFP36	1,14E-13	0,278517	0,768	0,754	2,18E-09	3	ZFP36
FOS	1,24E-13	0,291776	0,55	0,422	2,37E-09	3	FOS
IER2	3,88E-13	0,30587	0,878	0,874	7,43E-09	3	IER2
TMEM176E	4,16E-13	0,25763	0,997	0,984	7,95E-09	3	TMEM176B
HLA-A	6,44E-13	0,342958	1	0,999	1,23E-08	3	HLA-A
ITM2B	1,84E-12	0,372172	0,99	0,993	3,51E-08	3	ITM2B
COL15A1	2,35E-12	0,313875	0,617	0,531	4,50E-08	3	COL15A1
BST2	4,76E-12	0,342975	0,678	0,581	9,11E-08	3	BST2
CARMN	6,79E-12	0,284659	0,72	0,654	1,30E-07	3	CARMN
LUM	7,01E-12	0,465602	0,987	0,985	1,34E-07	3	LUM
SFRP1	8,95E-12	0,273585	0,743	0,658	1,71E-07	3	SFRP1
MDK	3,44E-11	0,329207	0,977	0,988	6,58E-07	3	MDK
FBN2	3,69E-11	0,331006	0,797	0,765	7,06E-07	3	FBN2
PLAC9	7,08E-11	0,254356	0,743	0,749	1,35E-06	3	PLAC9
GSN	2,40E-10	0,257686	0,936	0,942	4,60E-06	3	GSN
TIMP1	4,48E-10	0,551739	1	1	8,56E-06	3	TIMP1
ID2	5,30E-10	0,281219	0,775	0,739	1,01E-05	3	ID2
LGALS3BP	7,17E-10	0,285359	0,968	0,964	1,37E-05	3	LGALS3BP
A2M	4,47E-06	0,335903	0,392	0,311	0,085465	3	A2M
GREM1	8,90E-06	0,349656	0,804	0,767	0,170281	3	GREM1
PLAT	1,56E-05	0,293745	0,865	0,833	0,29859	3	PLAT
FADS1	1,91E-112	1,221641	0,984	0,93	3,66E-108	4	FADS1
SCD	2,18E-105	1,128236	0,997	0,964	4,16E-101	4	SCD
NPC21	1,46E-102	0,771503	1	0,999	2,79E-98	4	NPC2
EIF1	6,54E-96	0,54928	1	1	1,25E-91	4	EIF1
FDPS	1,04E-92	1,101278	0,994	0,975	1,99E-88	4	FDPS
FABP3	1,57E-91	1,36511	0,728	0,292	3,00E-87	4	FABP3
INSIG1	9,84E-89	0,833326	0,974	0,859	1,88E-84	4	INSIG1
FAM213A	3,79E-80	0,728324	0,984	0,934	7,25E-76	4	FAM213A
GRN1	3,04E-79	0,728916	1	0,99	5,82E-75	4	GRN
ASAH1	1,66E-77	0,690221	0,997	0,94	3,18E-73	4	ASAH1
IDI1	2,62E-76	0,862776	0,961	0,886	5,01E-72	4	IDI1
NEAT11	2,70E-73	0,673897	1	0,999	5,17E-69	4	NEAT1
TKT	1,79E-72	0,756255	1	0,998	3,42E-68	4	TKT
MVD	5,49E-72	0,682437	0,932	0,753	1,05E-67	4	MVD
HMGCS1	1,16E-71	0,73664	0,874	0,611	2,22E-67	4	HMGCS1
LPIN1	2,91E-68	0,554756	0,926	0,779	5,56E-64	4	LPIN1
FDFT1	9,59E-66	0,62918	0,977	0,916	1,83E-61	4	FDFT1
TMEM176A	1,36E-65	0,794708	0,997	0,964	2,61E-61	4	TMEM176A
FBXO32	2,12E-65	0,671356	0,835	0,484	4,05E-61	4	FBXO32
CTSK1	7,14E-63	1,060263	0,981	0,896	1,37E-58	4	CTSK
GPNMB1	3,07E-62	0,692136	0,977	0,929	5,87E-58	4	GPNMB
SQLE	7,59E-62	0,6885	0,984	0,922	1,45E-57	4	SQLE
UBXN11	7,86E-62	0,480618	1	0,992	1,50E-57	4	UBXN1
TMEM97	1,54E-61	0,637211	0,828	0,549	2,94E-57	4	TMEM97
CTSA	5,02E-61	0,57754	1	0,988	9,60E-57	4	CTSA
ERP291	2,68E-60	0,48908	1	0,993	5,12E-56	4	ERP29
PSAP1	4,12E-60	0,510481	1	0,996	7,87E-56	4	PSAP
BTG11	1,69E-59	0,565339	1	0,966	3,22E-55	4	BTG1

TMEM176I	2,38E-59	0,715606	1	0,983	4,56E-55	4	TMEM176B
CTSD	2,78E-56	0,471415	0,994	0,98	5,31E-52	4	CTSD
CCNG1	6,30E-54	0,46315	0,994	0,983	1,21E-49	4	CCNG1
SNHG71	3,13E-52	0,480719	0,997	0,978	5,99E-48	4	SNHG7
MMP21	5,89E-52	0,469896	1	0,991	1,13E-47	4	MMP2
MALAT11	4,79E-50	0,471709	1	1	9,16E-46	4	MALAT1
PTGDS1	6,49E-50	1,120143	0,968	0,892	1,24E-45	4	PTGDS
HLA-E	7,49E-50	0,444982	0,997	0,99	1,43E-45	4	HLA-E
ACAT2	3,01E-49	0,648231	0,945	0,859	5,76E-45	4	ACAT2
FBLN12	4,04E-49	0,697693	1	0,991	7,73E-45	4	FBLN1
TGFBI1	5,99E-49	0,547755	0,994	0,991	1,15E-44	4	TGFBI
FASN	2,08E-48	0,460916	0,874	0,685	3,98E-44	4	FASN
MSMO1	7,63E-47	0,519841	0,922	0,791	1,46E-42	4	MSMO1
PNRC11	1,19E-46	0,455082	0,994	0,957	2,27E-42	4	PNRC1
ST13	1,62E-46	0,370929	1	0,998	3,11E-42	4	ST13
ORAI3	2,60E-46	0,359128	0,883	0,672	4,97E-42	4	ORAI3
EIF3E1	4,11E-46	0,349879	1	0,999	7,87E-42	4	EIF3E
CIRBP1	9,92E-46	0,402135	1	0,996	1,90E-41	4	CIRBP
C1S1	2,71E-45	0,457966	0,997	0,985	5,19E-41	4	C1S
FADS2	1,73E-44	0,450968	0,977	0,927	3,31E-40	4	FADS2
LSS	2,78E-44	0,387259	0,913	0,756	5,31E-40	4	LSS
SEPP11	5,76E-44	0,600749	0,916	0,749	1,10E-39	4	SEPP1
MMP141	3,98E-43	0,445358	0,981	0,95	7,61E-39	4	MMP14
GLMP	4,52E-43	0,385964	0,99	0,908	8,65E-39	4	GLMP
HEXA	5,11E-43	0,41559	0,971	0,9	9,77E-39	4	HEXA
QPRT	8,63E-42	0,522256	0,977	0,871	1,65E-37	4	QPRT
NR2F11	2,27E-41	0,505703	0,997	0,972	4,34E-37	4	NR2F1
PRUNE2	5,37E-41	0,42953	0,748	0,482	1,03E-36	4	PRUNE2
TPGS1	1,64E-40	0,374765	0,961	0,866	3,14E-36	4	TPGS1
CAMLG1	5,22E-40	0,365648	0,987	0,957	9,99E-36	4	CAMLG
ATF4	5,82E-39	0,406737	1	0,996	1,11E-34	4	ATF4
LDLR	7,15E-39	0,397266	0,955	0,861	1,37E-34	4	LDLR
NAP1L1	4,94E-38	0,315328	1	0,999	9,45E-34	4	NAP1L1
IGBP11	8,18E-38	0,351149	0,99	0,953	1,56E-33	4	IGBP1
PTMA1	1,46E-37	0,540993	1	1	2,79E-33	4	PTMA
CCNI1	1,46E-37	0,324129	1	0,998	2,80E-33	4	CCNI
PFDN41	2,37E-37	0,388105	0,987	0,962	4,54E-33	4	PFDN4
APOE1	5,49E-37	0,79089	0,56	0,278	1,05E-32	4	APOE
COX4I1	7,02E-37	0,256686	1	1	1,34E-32	4	COX4I1
SAT11	1,04E-36	0,47704	0,997	0,973	1,99E-32	4	SAT1
PIK3IP1	3,18E-36	0,25094	0,693	0,411	6,09E-32	4	PIK3IP1
HMGCR	3,87E-36	0,316081	0,702	0,438	7,40E-32	4	HMGCR
CYGB	9,08E-36	0,453155	0,945	0,851	1,74E-31	4	CYGB
NEU1	2,40E-35	0,409567	0,932	0,854	4,60E-31	4	NEU1
IRF2BP2	2,82E-35	0,305277	0,848	0,686	5,39E-31	4	IRF2BP2
TPP1	4,27E-35	0,337673	0,964	0,91	8,17E-31	4	TPP1
RAB131	1,54E-34	0,340764	1	0,996	2,94E-30	4	RAB13
MMAB	1,76E-34	0,34824	0,935	0,868	3,37E-30	4	MMAB
B2M2	6,18E-34	0,320992	1	1	1,18E-29	4	B2M
CTSF1	9,03E-34	0,316212	0,754	0,524	1,73E-29	4	CTSF

SCPEP1	1,03E-33	0,376783	0,939	0,855	1,98E-29	4	SCPEP1
PCOLCE1	1,49E-33	0,509873	0,994	0,984	2,84E-29	4	PCOLCE
SMPDL3A1	2,43E-33	0,39886	0,987	0,92	4,64E-29	4	SMPDL3A
NBL11	3,26E-33	0,390208	0,971	0,871	6,23E-29	4	NBL1
MFAP42	3,65E-33	0,512507	0,984	0,91	6,98E-29	4	MFAP4
UQCRB	4,53E-33	0,267363	1	1	8,67E-29	4	UQCRB
GPR137B	6,17E-33	0,331383	0,958	0,859	1,18E-28	4	GPR137B
DHCR7	9,40E-33	0,390547	0,845	0,704	1,80E-28	4	DHCR7
FOXN31	2,39E-32	0,282778	0,828	0,641	4,57E-28	4	FOXN3
C1R1	2,48E-32	0,483101	0,99	0,974	4,74E-28	4	C1R
NUPR1	5,65E-32	0,33831	1	1	1,08E-27	4	NUPR1
VAT1	8,57E-32	0,331172	0,997	0,985	1,64E-27	4	VAT1
VIM1	8,78E-32	0,36432	1	1	1,68E-27	4	VIM
CFD2	9,08E-32	0,608274	0,984	0,947	1,74E-27	4	CFD
SNCA	1,58E-31	0,424655	0,981	0,949	3,02E-27	4	SNCA
CCNB1IP1	2,00E-31	0,347087	0,906	0,821	3,82E-27	4	CCNB1IP1
FN11	2,35E-31	0,35814	1	0,996	4,49E-27	4	FN1
SDCBP	2,61E-31	0,413727	1	0,985	4,99E-27	4	SDCBP
COL14A11	2,62E-31	0,421203	0,955	0,827	5,01E-27	4	COL14A1
RGS101	9,25E-31	0,339511	0,997	0,981	1,77E-26	4	RGS10
HSD17B14	1,20E-30	0,29949	0,796	0,58	2,30E-26	4	HSD17B14
PCBP21	2,60E-30	0,256077	1	0,997	4,96E-26	4	PCBP2
PBX11	2,93E-30	0,328009	0,909	0,84	5,61E-26	4	PBX1
STOM	3,94E-30	0,338306	0,997	0,996	7,54E-26	4	STOM
SYF2	4,01E-30	0,332628	0,994	0,956	7,68E-26	4	SYF2
HLA-B1	4,82E-30	0,427237	1	0,999	9,23E-26	4	HLA-B
MLXIP1	6,02E-30	0,368621	0,977	0,935	1,15E-25	4	MLXIP
LAMP1	1,16E-29	0,279934	0,994	0,988	2,22E-25	4	LAMP1
RARRES22	1,39E-29	0,593036	0,994	0,985	2,66E-25	4	RARRES2
SERPING11	2,26E-29	0,304776	0,906	0,758	4,32E-25	4	SERPING1
PLIN2	2,55E-29	0,45655	0,961	0,91	4,88E-25	4	PLIN2
ANGPTL21	8,77E-29	0,333729	0,935	0,846	1,68E-24	4	ANGPTL2
ELOVL5	1,36E-28	0,328776	0,994	0,961	2,60E-24	4	ELOVL5
HSD17B11	1,46E-28	0,265585	0,841	0,679	2,79E-24	4	HSD17B11
TM7SF2	2,76E-28	0,33739	0,657	0,446	5,27E-24	4	TM7SF2
BAG1	8,59E-28	0,323443	0,932	0,881	1,64E-23	4	BAG1
CYBRD11	1,04E-27	0,327234	0,964	0,943	2,00E-23	4	CYBRD1
PBX31	1,12E-27	0,351109	0,919	0,833	2,15E-23	4	PBX3
EIF4B1	2,10E-27	0,265324	0,997	0,994	4,01E-23	4	EIF4B
PERP	2,32E-27	0,295342	1	0,997	4,43E-23	4	PERP
ZFP36L11	2,96E-27	0,353362	0,994	0,968	5,66E-23	4	ZFP36L1
MGP1	3,07E-27	0,532436	0,929	0,822	5,86E-23	4	MGP
BBC3	5,58E-27	0,26265	0,761	0,543	1,07E-22	4	BBC3
GLUL	1,35E-26	0,28088	0,819	0,718	2,58E-22	4	GLUL
SPON2	2,42E-26	0,463932	1	0,997	4,63E-22	4	SPON2
LAMP21	6,29E-26	0,341775	0,964	0,935	1,20E-21	4	LAMP2
CEBPB	1,08E-25	0,291552	0,997	0,984	2,07E-21	4	CEBPB
TXNIP1	1,46E-25	0,36849	0,958	0,907	2,79E-21	4	TXNIP
ACLY	2,66E-25	0,352717	0,922	0,844	5,08E-21	4	ACLY
UBC	2,74E-25	0,265977	1	1	5,24E-21	4	UBC

HTRA11	6,98E-25	0,277323	0,845	0,71	1,33E-20	4	HTRA1
SERPINF11	7,42E-25	0,378136	0,919	0,821	1,42E-20	4	SERPINF1
ZNF106	7,93E-25	0,320658	0,977	0,952	1,52E-20	4	ZNF106
TRAPPC6A	1,93E-24	0,26281	0,783	0,635	3,68E-20	4	TRAPPC6A
SC5D	1,99E-24	0,27573	0,77	0,645	3,81E-20	4	SC5D
TGM2	3,49E-24	0,470619	1	0,986	6,67E-20	4	TGM2
BNIP3L	4,10E-24	0,282638	0,997	0,978	7,84E-20	4	BNIP3L
HLA-A1	9,10E-24	0,389191	1	0,999	1,74E-19	4	HLA-A
COL6A21	1,01E-23	0,276978	1	0,998	1,92E-19	4	COL6A2
AMDHD2	1,01E-23	0,255795	0,812	0,666	1,94E-19	4	AMDHD2
ZNF581	4,04E-23	0,26068	0,89	0,799	7,73E-19	4	ZNF581
FAM210B	5,50E-23	0,28989	0,916	0,853	1,05E-18	4	FAM210B
CST31	8,82E-23	0,27795	1	0,999	1,69E-18	4	CST3
JUNB1	8,88E-23	0,347716	0,951	0,915	1,70E-18	4	JUNB
H3F3A1	1,04E-22	0,252173	1	1	1,98E-18	4	H3F3A
TIMP21	3,70E-22	0,323838	1	0,99	7,07E-18	4	TIMP2
C6orf481	4,84E-22	0,265998	1	0,995	9,26E-18	4	C6orf48
HLA-C	7,46E-22	0,282653	1	0,999	1,43E-17	4	HLA-C
C10orf10	1,02E-21	0,429542	0,641	0,439	1,95E-17	4	C10orf10
DPP7	1,22E-21	0,253126	0,981	0,974	2,34E-17	4	DPP7
MYO61	1,68E-21	0,264242	0,929	0,82	3,21E-17	4	MYO6
EDNRB	6,16E-21	0,266254	0,715	0,522	1,18E-16	4	EDNRB
HCFC1R11	1,07E-20	0,268511	0,964	0,901	2,04E-16	4	HCFC1R1
SERPINB6	2,29E-20	0,293352	0,968	0,952	4,38E-16	4	SERPINB6
EPB41L4A-.	2,71E-20	0,256024	0,955	0,925	5,17E-16	4	EPB41L4A-AS1
PLPP11	4,60E-20	0,271943	0,916	0,84	8,79E-16	4	PLPP1
ID3	4,88E-20	0,523733	0,977	0,93	9,32E-16	4	ID3
LITAF	5,87E-20	0,271343	0,987	0,975	1,12E-15	4	LITAF
C14orf1	1,35E-19	0,304865	0,948	0,893	2,57E-15	4	C14orf1
SOX41	1,43E-19	0,357462	0,984	0,92	2,73E-15	4	SOX4
WBP2	2,57E-19	0,257779	0,906	0,829	4,92E-15	4	WBP2
ALDH2	4,13E-19	0,269385	0,929	0,874	7,89E-15	4	ALDH2
ADH5	5,64E-19	0,270322	1	0,99	1,08E-14	4	ADH5
FCGRT1	6,07E-19	0,26071	0,984	0,964	1,16E-14	4	FCGRT
SOD2	8,01E-19	0,465692	0,984	0,97	1,53E-14	4	SOD2
CD9	8,10E-19	0,30212	0,984	0,951	1,55E-14	4	CD9
ZFP36L21	1,08E-18	0,332648	0,981	0,966	2,07E-14	4	ZFP36L2
RARRES32	1,19E-18	0,312891	0,981	0,948	2,27E-14	4	RARRES3
TCF21	1,24E-18	0,298651	0,961	0,941	2,37E-14	4	TCF21
ITM2B1	2,31E-18	0,276149	0,997	0,992	4,42E-14	4	ITM2B
CSRP2	2,40E-18	0,354811	0,883	0,816	4,58E-14	4	CSRP2
LUM1	3,64E-18	0,385671	1	0,984	6,95E-14	4	LUM
PTN1	6,22E-18	0,294176	1	0,993	1,19E-13	4	PTN
AKAP121	7,56E-18	0,284431	0,994	0,977	1,45E-13	4	AKAP12
EBP	1,57E-17	0,321478	0,77	0,661	3,00E-13	4	EBP
CDKN1A	2,63E-17	0,378086	0,994	0,968	5,03E-13	4	CDKN1A
MAP1B	3,00E-17	0,275679	0,997	0,991	5,73E-13	4	MAP1B
NAMPT	1,94E-16	0,254827	0,9	0,864	3,71E-12	4	NAMPT
JUND	3,51E-16	0,250492	0,841	0,776	6,72E-12	4	JUND
HSD17B12	1,07E-15	0,266504	0,896	0,86	2,05E-11	4	HSD17B12

DDIT4	1,16E-15	0,314796	0,903	0,825	2,22E-11	4	DDIT4
LGALS3	1,87E-15	0,278	1	0,999	3,57E-11	4	LGALS3
RBP12	2,47E-15	0,327122	0,89	0,849	4,72E-11	4	RBP1
CD63	1,56E-14	0,258917	1	1	2,99E-10	4	CD63
GPX31	1,97E-14	0,314629	0,738	0,592	3,76E-10	4	GPX3
BDKRB2	2,20E-14	0,26994	0,951	0,903	4,21E-10	4	BDKRB2
PRRX1	1,24E-12	0,262493	0,777	0,682	2,36E-08	4	PRRX1
PDPN	3,64E-12	0,251958	0,893	0,856	6,96E-08	4	PDPN
PKM	1,29E-72	0,694247	1	0,999	2,46E-68	5	PKM
NQO1	3,06E-59	0,69316	1	0,989	5,84E-55	5	NQO1
PRDX1	2,72E-57	0,542381	1	1	5,20E-53	5	PRDX1
PTGR1	8,00E-57	0,673065	0,993	0,971	1,53E-52	5	PTGR1
GAPDH	4,22E-54	0,358612	1	1	8,07E-50	5	GAPDH
ANXA2	6,80E-48	0,400166	1	1	1,30E-43	5	ANXA2
ALDOA	1,00E-46	0,349611	1	1	1,92E-42	5	ALDOA
G6PD	9,86E-46	0,420984	0,997	0,985	1,89E-41	5	G6PD
LMNA	2,69E-45	0,41888	1	0,998	5,15E-41	5	LMNA
ENO1	1,57E-44	0,435061	1	0,998	3,00E-40	5	ENO1
CALR	2,35E-42	0,460124	1	0,998	4,49E-38	5	CALR
PSMB1	6,16E-40	0,388958	0,997	0,995	1,18E-35	5	PSMB1
PPIB	9,27E-40	0,383356	1	1	1,77E-35	5	PPIB
GSTP1	3,93E-39	0,356701	1	0,999	7,52E-35	5	GSTP1
TPI1	1,04E-37	0,345518	1	0,995	1,99E-33	5	TPI1
MGST1	1,26E-37	0,324059	1	1	2,42E-33	5	MGST1
PDIA6	1,43E-37	0,379119	0,993	0,974	2,73E-33	5	PDIA6
HNRNPA1	3,62E-37	0,390037	1	0,999	6,92E-33	5	HNRNPA1
KIAA0101	1,21E-36	0,280006	0,759	0,387	2,31E-32	5	KIAA0101
CD99	1,61E-36	0,291462	1	0,998	3,09E-32	5	CD99
S100A11	7,77E-36	0,308985	1	1	1,49E-31	5	S100A11
CD631	6,18E-35	0,371061	1	1	1,18E-30	5	CD63
COPS6	1,76E-34	0,367296	0,993	0,972	3,37E-30	5	COPS6
EIF3I	5,36E-34	0,322091	0,997	0,991	1,02E-29	5	EIF3I
ATP5G3	1,24E-33	0,331447	1	0,997	2,37E-29	5	ATP5G3
SKP1	2,79E-33	0,314195	0,997	0,999	5,34E-29	5	SKP1
PRDX4	1,66E-32	0,340941	0,986	0,947	3,18E-28	5	PRDX4
NNMT	4,13E-31	0,395779	1	0,995	7,89E-27	5	NNMT
NPM1	1,04E-30	0,314304	1	1	1,98E-26	5	NPM1
LDHA	2,44E-30	0,349128	1	0,992	4,66E-26	5	LDHA
HSPA5	3,32E-30	0,35711	1	0,974	6,35E-26	5	HSPA5
ANXA1	5,82E-30	0,338063	1	0,992	1,11E-25	5	ANXA1
NDUFB8	1,74E-29	0,299829	0,993	0,987	3,32E-25	5	NDUFB8
ETFB	1,11E-28	0,298491	0,979	0,951	2,12E-24	5	ETFB
AKR1B1	1,20E-28	0,372228	0,997	0,976	2,30E-24	5	AKR1B1
AP2M1	3,02E-28	0,271794	1	0,998	5,78E-24	5	AP2M1
COPE	3,34E-28	0,278023	1	0,995	6,39E-24	5	COPE
ARPC3	5,88E-28	0,275216	1	0,996	1,12E-23	5	ARPC3
PSMD8	5,90E-28	0,299135	1	0,993	1,13E-23	5	PSMD8
PSMB6	8,76E-28	0,309112	0,993	0,982	1,68E-23	5	PSMB6
PRDX2	1,34E-27	0,293237	0,997	0,984	2,57E-23	5	PRDX2
PLIN3	1,58E-27	0,294792	0,972	0,925	3,02E-23	5	PLIN3

IFITM21	1,62E-27	0,340647	1	0,996	3,09E-23	5	IFITM2
RPS27L	9,56E-27	0,275691	1	1	1,83E-22	5	RPS27L
TMBIM6	1,47E-26	0,251203	0,997	0,992	2,82E-22	5	TMBIM6
CLIC1	1,60E-26	0,278617	1	0,999	3,06E-22	5	CLIC1
ARPC1B	2,36E-26	0,300933	0,997	0,996	4,52E-22	5	ARPC1B
SQSTM1	2,48E-26	0,284586	1	1	4,74E-22	5	SQSTM1
DDOST	2,71E-26	0,302188	0,99	0,946	5,19E-22	5	DDOST
NDUFV2	8,25E-26	0,271953	0,99	0,988	1,58E-21	5	NDUFV2
ECM1	1,03E-25	0,293521	0,917	0,767	1,97E-21	5	ECM1
PSMA7	1,11E-25	0,277497	1	0,999	2,11E-21	5	PSMA7
MRPL28	2,34E-25	0,27111	0,972	0,905	4,48E-21	5	MRPL28
CHMP2A	7,80E-25	0,268448	0,997	0,985	1,49E-20	5	CHMP2A
PDIA3	9,05E-25	0,253228	0,993	0,972	1,73E-20	5	PDIA3
TMED9	2,26E-24	0,267329	0,997	0,987	4,31E-20	5	TMED9
UQCRC1	4,54E-24	0,259859	0,983	0,949	8,67E-20	5	UQCRC1
ANXA11	4,74E-24	0,274793	0,997	0,992	9,07E-20	5	ANXA11
SDHC	6,10E-24	0,25068	0,993	0,983	1,17E-19	5	SDHC
SCAND1	6,36E-24	0,255702	0,997	0,991	1,22E-19	5	SCAND1
LMAN2	8,62E-24	0,266076	0,99	0,972	1,65E-19	5	LMAN2
PRKCDBP	2,43E-23	0,308206	1	0,994	4,65E-19	5	PRKCDBP
TKT1	2,45E-23	0,343391	0,997	0,998	4,69E-19	5	TKT
EBPL	2,68E-23	0,285355	0,969	0,931	5,12E-19	5	EBPL
MDH2	3,64E-23	0,255362	0,997	0,985	6,96E-19	5	MDH2
NANS	7,90E-23	0,252867	0,931	0,842	1,51E-18	5	NANS
TALDO1	1,23E-22	0,288865	1	0,997	2,36E-18	5	TALDO1
SRSF3	1,89E-22	0,295207	0,997	0,975	3,62E-18	5	SRSF3
UCHL1	2,63E-22	0,258082	1	0,999	5,03E-18	5	UCHL1
HLA-C1	3,03E-22	0,287319	1	0,999	5,80E-18	5	HLA-C
TECR	6,40E-22	0,274249	0,928	0,867	1,22E-17	5	TECR
MGST3	7,04E-22	0,256611	1	0,996	1,35E-17	5	MGST3
MXRA8	7,33E-22	0,251502	0,993	0,972	1,40E-17	5	MXRA8
ATP5C1	1,47E-21	0,256274	0,983	0,984	2,81E-17	5	ATP5C1
MYDGF1	2,93E-21	0,274964	0,997	0,997	5,60E-17	5	MYDGF
RPL22L11	3,43E-21	0,340372	0,986	0,972	6,55E-17	5	RPL22L1
FHL21	3,63E-21	0,290909	1	0,996	6,94E-17	5	FHL2
MT1E1	3,89E-21	0,274769	0,976	0,962	7,44E-17	5	MT1E
PHGDH	5,37E-21	0,272629	0,966	0,9	1,03E-16	5	PHGDH
YWHAE	1,17E-20	0,259732	1	0,995	2,23E-16	5	YWHAE
SRSF2	1,23E-20	0,262307	0,979	0,952	2,35E-16	5	SRSF2
REXO2	3,55E-20	0,257288	0,993	0,995	6,78E-16	5	REXO2
APOBEC3C	6,56E-20	0,308422	0,917	0,827	1,25E-15	5	APOBEC3C
BASP1	2,05E-19	0,302007	0,983	0,99	3,91E-15	5	BASP1
KRT181	3,29E-19	0,428037	0,876	0,77	6,29E-15	5	KRT18
SERPINH1	9,40E-19	0,252007	0,997	0,985	1,80E-14	5	SERPINH1
S100A10	2,09E-18	0,2522	0,997	0,997	4,00E-14	5	S100A10
PITX1	1,46E-17	0,417322	0,986	0,987	2,78E-13	5	PITX1
ALDH1A1	2,28E-17	0,446544	0,993	0,991	4,37E-13	5	ALDH1A1
EIF5A	6,05E-17	0,333575	0,952	0,893	1,16E-12	5	EIF5A
GLRX1	1,59E-16	0,307622	1	0,993	3,03E-12	5	GLRX
IGFBP6	2,47E-16	0,319108	0,997	0,993	4,73E-12	5	IGFBP6

LXN1	4,65E-15	0,355096	0,979	0,935	8,89E-11	5	LXN
VIM2	1,58E-14	0,263641	1	1	3,02E-10	5	VIM
CLDN11	6,93E-14	0,557472	1	0,999	1,32E-09	5	CLDN11
AXL	6,17E-13	0,364912	0,962	0,925	1,18E-08	5	AXL
CTSC	7,04E-13	0,28135	1	0,999	1,35E-08	5	CTSC
TCF211	7,36E-13	0,260259	0,969	0,94	1,41E-08	5	TCF21
CDC42	2,19E-12	0,269573	0,99	0,985	4,19E-08	5	CDC42
GDF15	2,40E-12	0,452249	0,907	0,842	4,59E-08	5	GDF15
CEBPD	4,30E-12	0,255927	0,997	0,98	8,23E-08	5	CEBPD
SPON21	1,91E-11	0,266632	1	0,997	3,66E-07	5	SPON2
LRRC75A	1,20E-10	0,44621	0,697	0,607	2,29E-06	5	LRRC75A
CITED21	5,62E-10	0,298962	0,966	0,938	1,07E-05	5	CITED2
SDC4	6,50E-09	0,359358	0,921	0,888	0,000124	5	SDC4
ADIRF	7,24E-07	0,264095	0,941	0,921	0,013844	5	ADIRF
CTNNB1	8,09E-06	0,256582	0,793	0,771	0,154732	5	CTNNB1
ATP5E	8,26E-71	0,432805	1	1	1,58E-66	6	ATP5E
OST4	5,93E-68	0,516243	1	1	1,13E-63	6	OST4
SERF2	5,06E-64	0,384527	1	1	9,67E-60	6	SERF2
RPS28	4,94E-56	0,287637	1	1	9,45E-52	6	RPS28
FTH1	3,10E-55	0,492292	1	1	5,93E-51	6	FTH1
FTL2	3,11E-55	0,55747	1	1	5,94E-51	6	FTL
CRIM1	1,09E-54	0,480625	0,996	0,864	2,08E-50	6	CRIM1
MT-ND4	1,45E-54	0,411644	1	1	2,77E-50	6	MT-ND4
POLR2L	8,83E-53	0,503948	1	0,999	1,69E-48	6	POLR2L
RPL39	1,59E-52	0,28788	1	1	3,03E-48	6	RPL39
LINC00152	7,95E-50	0,509318	1	0,986	1,52E-45	6	LINC00152
TMSB10	3,21E-48	0,34953	1	1	6,14E-44	6	TMSB10
EXT11	6,89E-47	0,467774	0,993	0,933	1,32E-42	6	EXT1
RPS29	7,17E-45	0,262038	1	1	1,37E-40	6	RPS29
TMSB4X	8,86E-41	0,425407	1	1	1,69E-36	6	TMSB4X
RPL34	1,18E-40	0,257838	1	1	2,27E-36	6	RPL34
ITGAE	3,61E-40	0,415352	0,993	0,958	6,90E-36	6	ITGAE
CAMK2N1	1,55E-39	0,424139	1	0,991	2,97E-35	6	CAMK2N1
MIR4435-2	2,31E-39	0,409329	0,993	0,951	4,42E-35	6	MIR4435-2HG
OPN31	1,76E-38	0,461323	0,914	0,666	3,37E-34	6	OPN3
TOMM7	2,29E-38	0,285152	1	0,999	4,38E-34	6	TOMM7
MT-CO2	3,68E-38	0,339895	1	1	7,04E-34	6	MT-CO2
PPDPF	4,24E-38	0,339422	1	0,999	8,11E-34	6	PPDPF
MGAT5	4,49E-38	0,281482	0,721	0,367	8,59E-34	6	MGAT5
ZFAND51	4,65E-38	0,479898	0,996	0,984	8,89E-34	6	ZFAND5
USMG51	6,18E-38	0,360555	0,996	0,997	1,18E-33	6	USMG5
MPC21	1,26E-37	0,401923	0,996	0,987	2,41E-33	6	MPC2
TMEM47	1,27E-37	0,462867	0,955	0,827	2,44E-33	6	TMEM47
PAPSS21	8,80E-37	0,422316	0,989	0,916	1,68E-32	6	PAPSS2
MT-CO1	5,89E-36	0,27904	1	1	1,13E-31	6	MT-CO1
NABP1	2,92E-35	0,48963	0,996	0,971	5,59E-31	6	NABP1
WNT5A	3,07E-35	0,424672	0,955	0,805	5,87E-31	6	WNT5A
TMA7	1,84E-34	0,292733	1	0,999	3,52E-30	6	TMA7
IGFBP51	9,05E-34	0,759728	1	1	1,73E-29	6	IGFBP5
GNG12	1,95E-33	0,341082	1	0,973	3,72E-29	6	GNG12

COMMD6	9,00E-33	0,306053	1	0,996	1,72E-28	6	COMMD6
HEG1	1,12E-32	0,402092	0,989	0,933	2,13E-28	6	HEG1
ATP13A3	1,71E-32	0,311579	0,929	0,756	3,26E-28	6	ATP13A3
MYEOV2	1,43E-30	0,28939	1	0,992	2,74E-26	6	MYEOV2
SULF11	1,49E-30	0,477349	0,892	0,714	2,86E-26	6	SULF1
PXDN	2,42E-30	0,325488	0,981	0,945	4,62E-26	6	PXDN
ADAMTS5	3,58E-30	0,419251	0,926	0,716	6,85E-26	6	ADAMTS5
TMTC1	4,72E-30	0,34027	0,929	0,803	9,02E-26	6	TMTC1
STAT11	5,05E-30	0,340269	0,993	0,946	9,65E-26	6	STAT1
MT-ND1	5,63E-30	0,300097	1	0,999	1,08E-25	6	MT-ND1
NEDD81	6,74E-30	0,298314	1	0,999	1,29E-25	6	NEDD8
C17orf89	9,94E-30	0,358532	0,989	0,986	1,90E-25	6	C17orf89
PRNP	2,42E-29	0,50981	0,989	0,98	4,63E-25	6	PRNP
GUK1	3,73E-29	0,317005	1	0,998	7,13E-25	6	GUK1
PAPPA1	8,42E-29	0,492426	0,941	0,758	1,61E-24	6	PAPPA
MT-ND5	1,07E-28	0,298065	1	0,998	2,05E-24	6	MT-ND5
YWHAG	3,11E-28	0,297857	0,978	0,882	5,95E-24	6	YWHAG
LINC01420	5,17E-28	0,292152	0,993	0,956	9,88E-24	6	LINC01420
FSTL1	5,56E-28	0,390232	1	0,994	1,06E-23	6	FSTL1
GPRC5A1	3,00E-27	0,402712	0,955	0,853	5,74E-23	6	GPRC5A
DDAH1	3,31E-27	0,315596	0,9	0,709	6,34E-23	6	DDAH1
SMURF21	3,43E-27	0,416174	0,967	0,827	6,55E-23	6	SMURF2
TNS3	6,06E-27	0,322135	0,929	0,803	1,16E-22	6	TNS3
ATOX1	6,43E-27	0,331137	0,996	0,996	1,23E-22	6	ATOX1
NME4	7,39E-27	0,296522	0,993	0,992	1,41E-22	6	NME4
ELL2	1,03E-26	0,280335	0,952	0,799	1,96E-22	6	ELL2
USP531	1,63E-26	0,413857	0,941	0,789	3,11E-22	6	USP53
MMP24-AS	2,14E-26	0,28754	0,993	0,99	4,10E-22	6	MMP24-AS1
C7orf731	2,27E-26	0,28186	0,996	0,98	4,34E-22	6	C7orf73
GJA1	3,40E-26	0,330922	0,97	0,83	6,49E-22	6	GJA1
LAMC1	5,98E-26	0,292508	0,981	0,955	1,14E-21	6	LAMC1
TLK1	8,31E-26	0,292896	0,963	0,83	1,59E-21	6	TLK1
IGFBP31	1,61E-25	0,565534	0,952	0,759	3,08E-21	6	IGFBP3
TXNRD11	1,64E-25	0,33801	1	0,997	3,15E-21	6	TXNRD1
GABARAPL	6,43E-25	0,385873	0,963	0,944	1,23E-20	6	GABARAPL1
SLC7A111	7,05E-25	0,333367	0,948	0,806	1,35E-20	6	SLC7A11
RP11-356J!	1,26E-24	0,256498	0,937	0,775	2,41E-20	6	RP11-356J5.12
SLC40A1	1,38E-24	0,429277	0,825	0,591	2,64E-20	6	SLC40A1
RND31	1,48E-24	0,342111	1	0,997	2,83E-20	6	RND3
CEMIP1	2,58E-24	0,503781	0,926	0,756	4,94E-20	6	CEMIP
ATP6V1A	2,62E-24	0,280686	0,948	0,816	5,00E-20	6	ATP6V1A
LASP1	2,64E-24	0,323594	0,97	0,934	5,06E-20	6	LASP1
C9orf16	2,97E-24	0,268998	1	0,988	5,68E-20	6	C9orf16
SBF2-AS11	4,47E-24	0,43272	0,993	0,944	8,56E-20	6	SBF2-AS1
LMO71	6,13E-24	0,288596	0,985	0,945	1,17E-19	6	LMO7
COX14	1,50E-23	0,272576	0,996	0,98	2,88E-19	6	COX14
NGFRAP1	2,76E-23	0,311341	1	0,999	5,29E-19	6	NGFRAP1
IGF2	3,68E-23	0,357947	1	0,977	7,03E-19	6	IGF2
LMO7-AS1	5,75E-23	0,281603	0,907	0,786	1,10E-18	6	LMO7-AS1
UBL5	1,48E-22	0,274703	1	0,999	2,84E-18	6	UBL5

VGLL3	4,16E-22	0,257157	0,929	0,788	7,95E-18	6	VGLL3
COX17	4,23E-22	0,280619	0,948	0,887	8,08E-18	6	COX17
ZFAS1	1,22E-21	0,252048	1	0,998	2,32E-17	6	ZFAS1
PDE5A	2,29E-21	0,328023	0,996	0,954	4,37E-17	6	PDE5A
RDH10	4,27E-21	0,493479	0,978	0,944	8,16E-17	6	RDH10
TRAM1	1,25E-20	0,266481	1	0,998	2,39E-16	6	TRAM1
CCND11	4,04E-20	0,362655	0,993	0,964	7,73E-16	6	CCND1
SLC38A2	5,54E-20	0,251494	1	0,971	1,06E-15	6	SLC38A2
RHOBTB31	8,06E-20	0,419355	1	0,996	1,54E-15	6	RHOBTB3
CCND2	9,47E-20	0,343465	0,784	0,612	1,81E-15	6	CCND2
EPAS1	1,70E-19	0,273068	1	0,994	3,25E-15	6	EPAS1
COL5A2	2,18E-19	0,250161	0,996	0,974	4,17E-15	6	COL5A2
GAS61	5,58E-19	0,344453	0,996	0,988	1,07E-14	6	GAS6
CTTN	1,08E-18	0,382821	0,996	0,974	2,07E-14	6	CTTN
TP53I11	2,80E-18	0,283097	0,959	0,902	5,35E-14	6	TP53I11
MT-ND2	4,55E-18	0,262085	1	1	8,70E-14	6	MT-ND2
DAB21	2,36E-17	0,2992	0,996	0,99	4,50E-13	6	DAB2
LBH	5,33E-17	0,329519	0,993	0,945	1,02E-12	6	LBH
SLC20A21	7,17E-17	0,263463	0,81	0,636	1,37E-12	6	SLC20A2
SDC41	9,95E-17	0,298797	0,989	0,882	1,90E-12	6	SDC4
CTGF	1,31E-16	0,322377	0,981	0,899	2,50E-12	6	CTGF
CCL21	3,40E-15	0,471245	0,896	0,769	6,49E-11	6	CCL2
AXL1	5,25E-10	0,284078	0,989	0,923	1,00E-05	6	AXL
B4GALT1	2,20E-08	0,258665	0,74	0,604	0,000421	6	B4GALT1
CYP1B11	1,60E-07	0,258252	0,584	0,43	0,00306	6	CYP1B1
CTNNB11	3,32E-07	0,330981	0,885	0,762	0,006349	6	CTNNB1
LRRC75A1	1,14E-06	0,489372	0,732	0,604	0,021738	6	LRRC75A
CLDN111	3,58E-06	0,400372	1	0,999	0,068515	6	CLDN11
HSP90AA1	1,46E-138	1,51227	1	0,999	2,80E-134	7	HSP90AA1
UBC1	1,37E-135	1,322205	1	1	2,61E-131	7	UBC
CD632	2,10E-132	1,215976	1	1	4,02E-128	7	CD63
LAPTM4A	2,52E-131	1,173381	1	0,997	4,82E-127	7	LAPTM4A
ANXA5	4,15E-129	1,03301	1	1	7,94E-125	7	ANXA5
ITM2B2	2,59E-121	1,163117	1	0,992	4,95E-117	7	ITM2B
HLA-C2	1,06E-114	1,050031	1	0,999	2,02E-110	7	HLA-C
PSMA71	6,12E-113	0,87559	1	0,999	1,17E-108	7	PSMA7
PDIA31	1,57E-112	0,948063	1	0,972	3,00E-108	7	PDIA3
HLA-A2	4,01E-112	1,211232	1	0,999	7,66E-108	7	HLA-A
RPN2	1,42E-110	0,940499	0,996	0,953	2,71E-106	7	RPN2
TALDO11	4,26E-109	1,03683	1	0,997	8,14E-105	7	TALDO1
HSP90AB1	1,80E-107	1,062237	1	0,999	3,44E-103	7	HSP90AB1
sep-71	3,11E-107	0,967632	1	0,995	5,95E-103	7	07-sep
SSB	3,69E-107	0,952947	1	0,963	7,05E-103	7	SSB
TMBIM61	1,44E-106	0,941626	1	0,992	2,75E-102	7	TMBIM6
HSP90B1	4,37E-106	1,122417	1	0,964	8,36E-102	7	HSP90B1
UBXN4	9,01E-105	0,816313	0,996	0,96	1,72E-100	7	UBXN4
RTN4	4,91E-104	0,814572	1	1	9,40E-100	7	RTN4
ANXA21	9,96E-104	1,110721	1	1	1,90E-99	7	ANXA2
PGK1	1,14E-103	0,910451	0,996	0,973	2,18E-99	7	PGK1
TMEM59	1,46E-103	0,891677	1	0,992	2,79E-99	7	TMEM59

HSPA51	5,16E-103	1,130213	0,996	0,974	9,86E-99	7	HSPA5
ATP6AP2	7,43E-103	0,854809	0,996	0,94	1,42E-98	7	ATP6AP2
MVP	1,29E-100	0,862836	0,996	0,945	2,46E-96	7	MVP
ATP5B	3,24E-100	0,756597	0,996	0,994	6,19E-96	7	ATP5B
MAGED2	3,77E-100	0,890743	0,996	0,911	7,20E-96	7	MAGED2
CTSL	4,38E-100	0,940618	0,992	0,94	8,37E-96	7	CTSL
IGFBP7	1,64E-99	1,031663	1	1	3,14E-95	7	IGFBP7
PRDX11	2,46E-99	1,028698	1	1	4,70E-95	7	PRDX1
UBB	1,31E-98	0,831221	1	0,999	2,50E-94	7	UBB
PSMA4	1,43E-98	0,854705	1	0,988	2,74E-94	7	PSMA4
ATP1B3	2,06E-97	0,788761	0,996	0,952	3,93E-93	7	ATP1B3
REEP5	1,70E-96	0,755135	0,996	0,965	3,25E-92	7	REEP5
LGALS3BP1	9,80E-96	0,917556	0,989	0,962	1,87E-91	7	LGALS3BP
MYL12B	1,14E-94	0,799367	1	0,999	2,19E-90	7	MYL12B
TPM2	2,90E-93	0,841434	1	1	5,55E-89	7	TPM2
DNAJA1	6,67E-93	0,743583	0,996	0,954	1,27E-88	7	DNAJA1
EMC7	2,94E-91	0,658198	0,989	0,891	5,63E-87	7	EMC7
PSMA3	7,55E-91	0,778198	0,977	0,961	1,44E-86	7	PSMA3
ADH51	1,35E-89	0,77084	0,992	0,991	2,58E-85	7	ADH5
SPCS2	2,44E-89	0,700712	0,992	0,951	4,66E-85	7	SPCS2
ANXA12	1,06E-88	0,974148	1	0,992	2,02E-84	7	ANXA1
COPB2	1,34E-88	0,728224	0,977	0,848	2,56E-84	7	COPB2
PRDX41	2,99E-88	0,765774	0,996	0,946	5,72E-84	7	PRDX4
TUBA1A	1,49E-87	1,308098	1	0,997	2,85E-83	7	TUBA1A
LMNA1	5,65E-87	0,800296	1	0,998	1,08E-82	7	LMNA
SEC62	8,95E-87	0,720436	1	0,983	1,71E-82	7	SEC62
UQCRC11	9,69E-87	0,692748	0,996	0,948	1,85E-82	7	UQCRC1
HLA-B2	1,99E-86	1,024019	1	0,999	3,80E-82	7	HLA-B
CLU	3,09E-86	1,040373	0,996	0,957	5,92E-82	7	CLU
CCT5	1,63E-85	0,730842	0,977	0,87	3,13E-81	7	CCT5
APLP2	2,42E-85	0,797275	1	0,978	4,64E-81	7	APLP2
GSTP11	6,42E-85	0,656778	1	0,999	1,23E-80	7	GSTP1
CD91	2,29E-84	0,845199	1	0,95	4,38E-80	7	CD9
PPIB1	1,17E-83	0,696577	1	1	2,24E-79	7	PPIB
RNH1	1,19E-83	0,653168	1	0,982	2,28E-79	7	RNH1
C1R2	8,32E-83	1,169237	1	0,974	1,59E-78	7	C1R
ANXA6	8,44E-83	0,796361	1	0,978	1,61E-78	7	ANXA6
NUCB2	3,56E-82	0,741624	0,989	0,909	6,80E-78	7	NUCB2
MYL12A	7,10E-82	0,858901	1	1	1,36E-77	7	MYL12A
CALD1	8,08E-82	1,045412	1	1	1,54E-77	7	CALD1
HACD3	8,20E-82	0,595244	0,936	0,65	1,57E-77	7	HACD3
UCHL11	1,89E-81	1,005907	1	0,999	3,62E-77	7	UCHL1
PCOLCE2	4,15E-81	1,187702	1	0,983	7,93E-77	7	PCOLCE
ACTB	5,48E-81	0,696871	1	1	1,05E-76	7	ACTB
XRCC6	1,40E-80	0,685825	0,996	0,928	2,68E-76	7	XRCC6
ERP44	4,79E-80	0,644706	0,955	0,745	9,16E-76	7	ERP44
MGST11	2,14E-77	0,763731	1	1	4,10E-73	7	MGST1
EPHX1	5,18E-77	0,712751	0,962	0,781	9,91E-73	7	EPHX1
MDH1	1,32E-76	0,629535	0,973	0,917	2,52E-72	7	MDH1
COPS61	2,82E-76	0,659909	0,992	0,973	5,39E-72	7	COPS6

LAMP11	3,35E-76	0,664712	1	0,988	6,40E-72	7	LAMP1
ENO11	4,47E-76	0,818165	1	0,998	8,54E-72	7	ENO1
PSMC2	7,61E-76	0,596131	0,947	0,788	1,46E-71	7	PSMC2
PSMD7	3,49E-75	0,612701	0,973	0,926	6,68E-71	7	PSMD7
XRCC5	4,90E-75	0,661643	0,989	0,933	9,37E-71	7	XRCC5
ALDOA1	9,17E-75	0,562902	1	1	1,75E-70	7	ALDOA
PSMB11	1,86E-74	0,62716	1	0,995	3,56E-70	7	PSMB1
MDH21	1,50E-73	0,5576	0,996	0,985	2,87E-69	7	MDH2
ACTR10	4,02E-73	0,577109	0,966	0,777	7,69E-69	7	ACTR10
LAMP22	8,22E-73	0,647615	0,989	0,934	1,57E-68	7	LAMP2
CCT2	2,09E-72	0,593625	0,981	0,897	4,00E-68	7	CCT2
PKM1	4,76E-72	0,86271	1	0,999	9,10E-68	7	PKM
FUCA2	5,05E-72	0,548176	0,977	0,841	9,65E-68	7	FUCA2
HEXB	1,62E-71	0,687114	0,973	0,831	3,10E-67	7	HEXB
C1S2	3,93E-71	0,87712	1	0,985	7,51E-67	7	C1S
PSMD1	7,97E-71	0,614509	0,97	0,834	1,52E-66	7	PSMD1
ACTG21	1,94E-70	1,454601	1	1	3,70E-66	7	ACTG2
EPRS	4,10E-70	0,711239	0,977	0,849	7,84E-66	7	EPRS
TIMP11	5,30E-70	0,900418	1	1	1,01E-65	7	TIMP1
PSMC3	2,43E-69	0,574506	0,992	0,919	4,64E-65	7	PSMC3
TPM1	3,60E-69	0,879253	1	1	6,89E-65	7	TPM1
NDUFS2	2,08E-68	0,550049	0,966	0,845	3,98E-64	7	NDUFS2
ARPC31	7,28E-68	0,526101	0,989	0,997	1,39E-63	7	ARPC3
PDIA61	1,85E-67	0,716349	0,989	0,974	3,54E-63	7	PDIA6
REXO21	6,02E-67	0,62188	0,992	0,995	1,15E-62	7	REXO2
PSMD6	9,89E-67	0,53674	0,943	0,768	1,89E-62	7	PSMD6
NQO11	2,39E-66	0,90378	0,996	0,99	4,57E-62	7	NQO1
RARS	3,30E-66	0,645016	0,985	0,866	6,32E-62	7	RARS
CYBA	3,67E-66	0,848661	0,992	0,98	7,03E-62	7	CYBA
SKP11	4,15E-66	0,546871	1	0,999	7,94E-62	7	SKP1
CTSC1	6,55E-66	0,848668	1	0,999	1,25E-61	7	CTSC
CD47	7,37E-66	0,634308	0,992	0,926	1,41E-61	7	CD47
ARL6IP5	1,40E-65	0,505285	0,992	0,98	2,67E-61	7	ARL6IP5
GSN1	1,58E-65	0,642616	0,996	0,936	3,02E-61	7	GSN
GSTO1	2,52E-65	0,660046	0,996	0,994	4,82E-61	7	GSTO1
ARF4	3,61E-65	0,578387	0,996	0,993	6,90E-61	7	ARF4
RABAC1	1,27E-64	0,497618	1	1	2,43E-60	7	RABAC1
DHRS7	3,77E-64	0,5412	0,955	0,789	7,21E-60	7	DHRS7
VCP	9,59E-64	0,613249	0,992	0,934	1,83E-59	7	VCP
LGALS31	1,11E-63	0,722751	1	0,999	2,13E-59	7	LGALS3
COPE1	1,84E-63	0,501377	0,996	0,995	3,52E-59	7	COPE
NUDC	2,24E-63	0,526737	0,985	0,919	4,28E-59	7	NUDC
PPP1R7	8,54E-63	0,561487	0,973	0,887	1,63E-58	7	PPP1R7
NDUFA9	4,11E-62	0,484153	0,947	0,774	7,86E-58	7	NDUFA9
HEXA1	4,31E-62	0,60722	0,973	0,901	8,24E-58	7	HEXA
CTSA1	9,43E-62	0,711389	1	0,988	1,80E-57	7	CTSA
CDC123	1,17E-61	0,475546	0,955	0,77	2,24E-57	7	CDC123
PSMD81	1,26E-61	0,55708	0,996	0,994	2,41E-57	7	PSMD8
PSMC4	1,59E-61	0,503488	0,943	0,757	3,04E-57	7	PSMC4
SLC3A2	2,50E-61	0,683432	0,985	0,912	4,77E-57	7	SLC3A2

DEGS1	2,71E-61	0,575613	0,977	0,847	5,19E-57	7	DEGS1
EIF4A3	2,73E-61	0,472815	0,917	0,654	5,21E-57	7	EIF4A3
LDHB	3,11E-61	0,443943	1	1	5,94E-57	7	LDHB
G6PD1	6,13E-61	0,637961	1	0,985	1,17E-56	7	G6PD
LUM2	6,16E-61	0,944071	0,996	0,985	1,18E-56	7	LUM
BLVRA	4,19E-60	0,425796	0,932	0,665	8,02E-56	7	BLVRA
PDHB	4,46E-60	0,499472	0,939	0,793	8,52E-56	7	PDHB
GSTM3	4,80E-60	0,661467	0,97	0,965	9,19E-56	7	GSTM3
CCT7	5,76E-60	0,514841	0,981	0,915	1,10E-55	7	CCT7
AP2M11	1,16E-59	0,51993	1	0,998	2,22E-55	7	AP2M1
EMC3	3,76E-59	0,4742	0,962	0,857	7,18E-55	7	EMC3
PHGDH1	4,17E-59	0,76786	0,985	0,899	7,98E-55	7	PHGDH
CAPZB	7,06E-59	0,488411	1	0,993	1,35E-54	7	CAPZB
FDPS1	1,99E-58	0,828963	0,996	0,975	3,81E-54	7	FDPS
PSME2	2,46E-58	0,640579	0,962	0,952	4,71E-54	7	PSME2
GARS	3,00E-58	0,707658	0,989	0,969	5,74E-54	7	GARS
DDX1	4,13E-58	0,509404	0,947	0,825	7,90E-54	7	DDX1
SON	4,89E-58	0,681554	0,996	0,967	9,34E-54	7	SON
SIL1	5,13E-58	0,510437	0,936	0,758	9,81E-54	7	SIL1
RAC1	9,84E-58	0,456883	1	0,999	1,88E-53	7	RAC1
AIMP1	1,10E-57	0,506928	0,97	0,84	2,10E-53	7	AIMP1
PSMB3	4,34E-57	0,568315	0,981	0,977	8,31E-53	7	PSMB3
KIF5B	4,36E-57	0,682219	0,992	0,971	8,35E-53	7	KIF5B
TUBB4B	4,75E-57	0,847953	0,977	0,914	9,08E-53	7	TUBB4B
DDOST1	9,48E-57	0,535595	0,985	0,947	1,81E-52	7	DDOST
ILK	2,24E-56	0,602813	0,996	0,971	4,28E-52	7	ILK
SF3B2	2,86E-56	0,502163	0,996	0,938	5,48E-52	7	SF3B2
LMAN1	3,07E-56	0,523006	0,992	0,929	5,87E-52	7	LMAN1
ACAT1	3,42E-56	0,486188	0,973	0,851	6,54E-52	7	ACAT1
C1GALT1C1	5,57E-56	0,43348	0,822	0,447	1,06E-51	7	C1GALT1C1
MAGED1	1,85E-55	0,613351	0,97	0,935	3,53E-51	7	MAGED1
PSMC1	2,08E-55	0,49358	0,966	0,889	3,98E-51	7	PSMC1
HADHB	2,82E-55	0,473311	0,966	0,894	5,39E-51	7	HADHB
CALM21	3,03E-55	0,694751	1	1	5,80E-51	7	CALM2
CALR1	3,94E-55	0,618958	1	0,998	7,54E-51	7	CALR
HDAC2	2,27E-54	0,488826	0,955	0,834	4,35E-50	7	HDAC2
PRDX21	1,01E-53	0,494864	0,989	0,985	1,93E-49	7	PRDX2
P4HA2	1,16E-53	0,456095	0,939	0,741	2,21E-49	7	P4HA2
GHITM	1,43E-53	0,452061	0,996	0,979	2,74E-49	7	GHITM
TAX1BP1	1,79E-53	0,531457	0,992	0,977	3,43E-49	7	TAX1BP1
PRMT1	2,09E-53	0,49867	0,966	0,882	4,00E-49	7	PRMT1
OCIAD1	2,47E-53	0,459193	0,977	0,934	4,73E-49	7	OCIAD1
ATP6V0D1	3,68E-53	0,462087	0,977	0,919	7,04E-49	7	ATP6V0D1
PSAP2	4,09E-53	0,638615	1	0,996	7,82E-49	7	PSAP
NNMT1	4,35E-53	0,741062	1	0,995	8,32E-49	7	NNMT
LRPAP1	5,32E-53	0,509203	0,985	0,927	1,02E-48	7	LRPAP1
RRAGA	7,51E-53	0,445713	0,947	0,861	1,44E-48	7	RRAGA
SCPEP11	8,01E-53	0,50243	0,973	0,853	1,53E-48	7	SCPEP1
SOD1	8,19E-53	0,443803	0,989	0,999	1,57E-48	7	SOD1
ATP5C11	1,60E-52	0,467233	0,989	0,983	3,05E-48	7	ATP5C1

ATP5A1	1,62E-52	0,447722	1	0,996	3,10E-48	7	ATP5A1
PTGR11	3,52E-52	0,869818	0,992	0,972	6,73E-48	7	PTGR1
DDX24	3,82E-52	0,523911	0,996	0,943	7,31E-48	7	DDX24
FAM50A	4,01E-52	0,456319	0,989	0,944	7,67E-48	7	FAM50A
BCAP31	4,10E-52	0,493224	0,985	0,929	7,85E-48	7	BCAP31
XRN2	4,87E-52	0,515909	0,989	0,931	9,31E-48	7	XRN2
CNN3	7,24E-52	0,485139	0,996	0,994	1,38E-47	7	CNN3
MYL61	8,76E-52	0,538474	1	1	1,68E-47	7	MYL6
VPS35	1,34E-51	0,456163	0,955	0,869	2,57E-47	7	VPS35
ATXN10	1,64E-51	0,490053	0,97	0,908	3,15E-47	7	ATXN10
SDHA	1,96E-51	0,455067	0,955	0,752	3,75E-47	7	SDHA
PERP1	2,64E-51	0,482636	1	0,997	5,04E-47	7	PERP
SNRPB2	3,50E-51	0,483439	0,966	0,959	6,70E-47	7	SNRPB2
NANS1	3,75E-51	0,580566	0,97	0,839	7,17E-47	7	NANS
EMP3	4,09E-51	0,512723	0,996	0,999	7,83E-47	7	EMP3
FERMT2	4,68E-51	0,639262	0,985	0,918	8,95E-47	7	FERMT2
ARL1	5,08E-51	0,427751	0,958	0,861	9,71E-47	7	ARL1
DYNC1I2	9,79E-51	0,539461	0,989	0,93	1,87E-46	7	DYNC1I2
SERPINF12	1,19E-50	0,645441	0,977	0,817	2,28E-46	7	SERPINF1
TUFM	1,26E-50	0,461277	0,996	0,977	2,41E-46	7	TUFM
TMEM50A	1,39E-50	0,465987	0,97	0,974	2,66E-46	7	TMEM50A
DPM1	1,63E-50	0,450467	0,924	0,809	3,11E-46	7	DPM1
CCT4	2,10E-50	0,500682	0,985	0,943	4,02E-46	7	CCT4
SERPINH11	3,81E-50	0,620206	0,996	0,985	7,29E-46	7	SERPINH1
PUF60	4,36E-50	0,43084	0,958	0,797	8,34E-46	7	PUF60
ECH1	4,49E-50	0,478824	0,966	0,839	8,58E-46	7	ECH1
HAT1	4,72E-50	0,315146	0,807	0,438	9,02E-46	7	HAT1
ETFB1	5,20E-50	0,526329	0,981	0,951	9,94E-46	7	ETFB
ASNS	1,65E-49	0,64733	0,837	0,6	3,15E-45	7	ASNS
EMC2	2,33E-49	0,404633	0,864	0,607	4,46E-45	7	EMC2
HSPA9	2,93E-49	0,478435	0,958	0,879	5,60E-45	7	HSPA9
HNRNPH2	4,18E-49	0,413074	0,924	0,759	7,99E-45	7	HNRNPH2
IK	4,45E-49	0,474068	0,936	0,814	8,50E-45	7	IK
NCL	5,89E-49	0,562388	1	0,988	1,13E-44	7	NCL
CHMP2A1	9,30E-49	0,436804	0,981	0,987	1,78E-44	7	CHMP2A
UNC50	2,09E-48	0,357034	0,909	0,64	3,99E-44	7	UNC50
UQCRC2	2,13E-48	0,42987	0,989	0,954	4,07E-44	7	UQCRC2
OAT	2,33E-48	0,468359	0,992	0,911	4,45E-44	7	OAT
TPR	3,21E-48	0,504053	0,966	0,858	6,13E-44	7	TPR
CLTA	5,33E-48	0,391827	1	1	1,02E-43	7	CLTA
ACTR3	6,18E-48	0,540481	0,996	0,98	1,18E-43	7	ACTR3
CPNE3	7,59E-48	0,395644	0,958	0,768	1,45E-43	7	CPNE3
RAB2A	9,01E-48	0,414048	0,992	0,985	1,72E-43	7	RAB2A
NDUFC2	1,07E-47	0,491442	0,989	0,985	2,04E-43	7	NDUFC2
PCMT1	1,15E-47	0,422488	0,955	0,83	2,20E-43	7	PCMT1
VPS29	1,24E-47	0,431892	0,97	0,959	2,36E-43	7	VPS29
ALDH21	2,31E-47	0,556522	0,97	0,871	4,42E-43	7	ALDH2
SGCE	2,51E-47	0,398169	0,818	0,494	4,80E-43	7	SGCE
ATP6V1E1	3,72E-47	0,43647	0,989	0,976	7,12E-43	7	ATP6V1E1
GRN2	4,56E-47	0,596909	1	0,99	8,72E-43	7	GRN

PSMD2	5,20E-47	0,426958	0,977	0,886	9,95E-43	7	PSMD2
PSMD4	7,18E-47	0,394418	0,996	0,952	1,37E-42	7	PSMD4
SYPL1	9,58E-47	0,454556	0,977	0,901	1,83E-42	7	SYPL1
UFL1	1,00E-46	0,373827	0,848	0,569	1,92E-42	7	UFL1
TFPI	1,59E-46	0,535681	0,996	0,975	3,05E-42	7	TFPI
DECR1	2,05E-46	0,397324	0,955	0,921	3,93E-42	7	DECR1
FH	2,13E-46	0,387265	0,875	0,622	4,07E-42	7	FH
MGST31	2,95E-46	0,478127	0,996	0,996	5,64E-42	7	MGST3
GLB1	4,27E-46	0,433158	0,871	0,633	8,17E-42	7	GLB1
ALDH1A11	8,05E-46	1,398335	1	0,99	1,54E-41	7	ALDH1A1
RCN2	1,12E-45	0,430404	0,989	0,947	2,14E-41	7	RCN2
TRAPPC4	1,58E-45	0,396779	0,928	0,808	3,03E-41	7	TRAPPC4
SERPINE2	1,82E-45	0,74316	0,989	0,91	3,47E-41	7	SERPINE2
ANXA7	6,02E-45	0,386921	0,958	0,873	1,15E-40	7	ANXA7
ECM11	1,04E-44	0,481593	0,917	0,769	2,00E-40	7	ECM1
MORF4L1	2,05E-44	0,421662	1	0,997	3,92E-40	7	MORF4L1
CCPG11	2,07E-44	0,396435	0,924	0,675	3,96E-40	7	CCPG1
TXNL1	2,12E-44	0,440798	0,97	0,952	4,06E-40	7	TXNL1
CD151	2,45E-44	0,530367	1	0,995	4,68E-40	7	CD151
ACAT21	2,54E-44	0,608713	0,973	0,858	4,86E-40	7	ACAT2
SDCBP1	3,64E-44	0,440423	0,992	0,986	6,95E-40	7	SDCBP
ZNF622	5,29E-44	0,375533	0,909	0,712	1,01E-39	7	ZNF622
ADRM1	5,90E-44	0,426199	0,992	0,962	1,13E-39	7	ADRM1
SUCLG1	6,06E-44	0,432713	0,943	0,894	1,16E-39	7	SUCLG1
YWHAB	6,70E-44	0,393149	1	0,994	1,28E-39	7	YWHAB
FKBP3	8,18E-44	0,422529	0,939	0,876	1,56E-39	7	FKBP3
VCAM1	8,49E-44	0,782498	0,932	0,801	1,62E-39	7	VCAM1
ACTN1	1,06E-43	0,61439	1	0,996	2,03E-39	7	ACTN1
HSPD1	1,51E-43	0,474884	0,981	0,92	2,88E-39	7	HSPD1
MFAP1	1,53E-43	0,347082	0,83	0,55	2,92E-39	7	MFAP1
HNRNPK	2,53E-43	0,435193	1	0,995	4,84E-39	7	HNRNPK
LEPROT	2,65E-43	0,441658	0,985	0,984	5,07E-39	7	LEPROT
DNAJC8	6,34E-43	0,424892	0,989	0,955	1,21E-38	7	DNAJC8
MYL91	8,23E-43	0,542949	1	1	1,57E-38	7	MYL9
POLR2G	1,04E-42	0,394662	0,939	0,869	1,98E-38	7	POLR2G
ETFA	1,18E-42	0,381875	0,955	0,907	2,26E-38	7	ETFA
SMC3	1,24E-42	0,440865	0,924	0,761	2,37E-38	7	SMC3
SRP54	1,38E-42	0,376448	0,89	0,652	2,65E-38	7	SRP54
ATIC	1,39E-42	0,367984	0,864	0,614	2,65E-38	7	ATIC
RPN1	1,44E-42	0,490441	0,981	0,892	2,76E-38	7	RPN1
GPX8	2,93E-42	0,399139	0,985	0,976	5,61E-38	7	GPX8
MICU2	3,96E-42	0,291093	0,811	0,495	7,57E-38	7	MICU2
ANXA4	4,20E-42	0,431456	0,939	0,879	8,02E-38	7	ANXA4
TUBB2A	4,75E-42	0,457771	0,864	0,66	9,08E-38	7	TUBB2A
PEPD	5,44E-42	0,446337	0,955	0,867	1,04E-37	7	PEPD
COPS4	9,64E-42	0,374723	0,932	0,806	1,84E-37	7	COPS4
PSMA5	1,02E-41	0,474896	0,955	0,97	1,96E-37	7	PSMA5
SRI	1,18E-41	0,423629	0,981	0,971	2,26E-37	7	SRI
AKR1B11	1,22E-41	0,639303	0,981	0,978	2,33E-37	7	AKR1B1
CALU	1,34E-41	0,561337	1	0,997	2,57E-37	7	CALU

PSMD14	1,63E-41	0,372976	0,924	0,837	3,12E-37	7	PSMD14
SUPT16H	2,11E-41	0,409674	0,924	0,715	4,04E-37	7	SUPT16H
RER1	2,64E-41	0,390311	0,989	0,98	5,04E-37	7	RER1
KPNA2	2,76E-41	0,382925	0,875	0,643	5,28E-37	7	KPNA2
GGNBP2	2,94E-41	0,409786	0,962	0,851	5,62E-37	7	GGNBP2
DNAJB11	3,33E-41	0,370588	0,92	0,753	6,38E-37	7	DNAJB11
SEC13	3,98E-41	0,401096	0,958	0,924	7,61E-37	7	SEC13
PSMB61	4,05E-41	0,455192	0,985	0,982	7,74E-37	7	PSMB6
EIF4A1	5,14E-41	0,497341	0,989	0,973	9,84E-37	7	EIF4A1
WDR61	5,43E-41	0,337501	0,886	0,709	1,04E-36	7	WDR61
CCT3	8,83E-41	0,40267	0,985	0,973	1,69E-36	7	CCT3
NDUFB81	9,38E-41	0,424677	0,985	0,987	1,79E-36	7	NDUFB8
ORMDL2	9,96E-41	0,344525	0,856	0,637	1,90E-36	7	ORMDL2
ITGB1	1,05E-40	0,697871	1	1	2,01E-36	7	ITGB1
LDHA1	1,65E-40	0,478538	0,996	0,993	3,16E-36	7	LDHA
PLRG1	2,13E-40	0,357446	0,845	0,611	4,08E-36	7	PLRG1
EID1	2,13E-40	0,405173	0,992	0,999	4,08E-36	7	EID1
DPP3	2,25E-40	0,277108	0,773	0,447	4,30E-36	7	DPP3
OS9	3,69E-40	0,434317	0,962	0,869	7,06E-36	7	OS9
FBLN13	3,92E-40	0,87603	1	0,991	7,50E-36	7	FBLN1
MTX1	4,08E-40	0,338308	0,902	0,695	7,79E-36	7	MTX1
AIFM2	4,48E-40	0,360834	0,886	0,693	8,56E-36	7	AIFM2
HNRNPA2B	6,38E-40	0,387715	1	0,992	1,22E-35	7	HNRNPA2B1
COPB1	7,07E-40	0,508995	0,97	0,864	1,35E-35	7	COPB1
GBE1	8,07E-40	0,489684	0,928	0,785	1,54E-35	7	GBE1
CAPZA2	8,22E-40	0,402562	0,989	0,967	1,57E-35	7	CAPZA2
CNIH4	9,74E-40	0,363786	0,947	0,914	1,86E-35	7	CNIH4
F10	1,02E-39	0,319468	0,742	0,423	1,95E-35	7	F10
HERPUD1	1,12E-39	0,422767	0,985	0,953	2,15E-35	7	HERPUD1
CTNNA1	1,41E-39	0,479116	0,981	0,919	2,69E-35	7	CTNNA1
BLVRB	1,60E-39	0,435536	0,985	0,97	3,06E-35	7	BLVRB
PLD31	1,77E-39	0,482751	0,992	0,978	3,38E-35	7	PLD3
AGA	1,89E-39	0,369294	0,856	0,606	3,62E-35	7	AGA
TRPV2	2,34E-39	0,439077	0,83	0,644	4,48E-35	7	TRPV2
BSG	3,36E-39	0,403157	1	0,998	6,42E-35	7	BSG
CORO1B	4,07E-39	0,387065	0,989	0,945	7,78E-35	7	CORO1B
SYNCRIP	4,39E-39	0,408228	0,955	0,897	8,40E-35	7	SYNCRIP
SQSTM11	4,93E-39	0,542348	1	1	9,42E-35	7	SQSTM1
ACTA21	6,92E-39	1,61246	0,996	0,999	1,32E-34	7	ACTA2
AKR1C1	7,87E-39	0,535959	0,883	0,709	1,50E-34	7	AKR1C1
RBM42	9,46E-39	0,341921	0,92	0,768	1,81E-34	7	RBM42
NDFIP1	1,09E-38	0,377204	0,958	0,933	2,08E-34	7	NDFIP1
ATP5F1	1,32E-38	0,368838	0,996	0,987	2,53E-34	7	ATP5F1
SMPDL3A2	1,40E-38	0,534863	0,981	0,921	2,68E-34	7	SMPDL3A
VIMP	1,59E-38	0,426888	0,973	0,942	3,04E-34	7	VIMP
HSPA8	2,09E-38	0,467503	1	1	3,99E-34	7	HSPA8
TMED91	3,53E-38	0,463058	0,992	0,988	6,75E-34	7	TMED9
HNRNPU	3,92E-38	0,425374	0,981	0,959	7,49E-34	7	HNRNPU
PFKP	4,11E-38	0,457001	0,909	0,781	7,86E-34	7	PFKP
CALM1	4,54E-38	0,408403	0,985	0,992	8,67E-34	7	CALM1

IFNGR1	5,35E-38	0,334804	0,818	0,544	1,02E-33	7	IFNGR1
TM9SF2	6,42E-38	0,395341	0,936	0,778	1,23E-33	7	TM9SF2
TRAM11	6,85E-38	0,517537	1	0,998	1,31E-33	7	TRAM1
PSMB2	6,92E-38	0,375087	0,989	0,973	1,32E-33	7	PSMB2
NFE2L2	7,71E-38	0,426943	1	0,991	1,47E-33	7	NFE2L2
CBR1	1,03E-37	0,406789	0,97	0,907	1,96E-33	7	CBR1
TSPAN4	1,08E-37	0,398538	0,996	0,984	2,06E-33	7	TSPAN4
DCAF13	1,96E-37	0,349345	0,83	0,617	3,75E-33	7	DCAF13
NEMF	2,72E-37	0,384468	0,898	0,717	5,21E-33	7	NEMF
CCT6A	2,92E-37	0,366899	0,966	0,919	5,58E-33	7	CCT6A
HACD1	3,15E-37	0,41601	0,943	0,878	6,01E-33	7	HACD1
MESDC2	3,15E-37	0,360113	0,947	0,829	6,02E-33	7	MESDC2
PTN2	3,41E-37	0,564518	0,996	0,994	6,52E-33	7	PTN
CARS	3,53E-37	0,351575	0,928	0,754	6,74E-33	7	CARS
AHSA1	4,67E-37	0,323805	0,909	0,728	8,93E-33	7	AHSA1
HSPA1A	8,14E-37	0,28679	0,758	0,451	1,56E-32	7	HSPA1A
VTN	1,84E-36	0,432237	0,61	0,305	3,52E-32	7	VTN
CCT8	1,87E-36	0,384543	1	0,993	3,58E-32	7	CCT8
QSOX11	2,15E-36	0,664721	1	0,991	4,11E-32	7	QSOX1
UROD	2,23E-36	0,364193	0,917	0,846	4,26E-32	7	UROD
GLRX3	2,35E-36	0,358624	0,955	0,896	4,50E-32	7	GLRX3
PDHA1	2,59E-36	0,297234	0,856	0,617	4,94E-32	7	PDHA1
SF3B1	4,36E-36	0,398989	0,977	0,888	8,34E-32	7	SF3B1
PSMG1	4,42E-36	0,338585	0,909	0,769	8,45E-32	7	PSMG1
FEZ2	4,84E-36	0,374066	0,932	0,814	9,25E-32	7	FEZ2
LACTB2	6,35E-36	0,407664	0,902	0,766	1,21E-31	7	LACTB2
PSMC6	6,75E-36	0,37533	0,924	0,795	1,29E-31	7	PSMC6
HM13	6,85E-36	0,344644	0,951	0,915	1,31E-31	7	HM13
MGMT	7,49E-36	0,389564	0,977	0,981	1,43E-31	7	MGMT
PRCP	9,52E-36	0,39515	0,936	0,792	1,82E-31	7	PRCP
FAP	9,63E-36	0,500835	0,894	0,743	1,84E-31	7	FAP
SRSF11	1,02E-35	0,384099	0,985	0,959	1,95E-31	7	SRSF11
DDX51	1,17E-35	0,50656	1	0,992	2,24E-31	7	DDX5
CFH	1,36E-35	0,466448	0,803	0,562	2,60E-31	7	CFH
PSMD13	1,71E-35	0,354103	0,898	0,784	3,27E-31	7	PSMD13
POMP1	1,76E-35	0,410628	1	1	3,36E-31	7	POMP
NAE1	1,83E-35	0,352854	0,89	0,75	3,49E-31	7	NAE1
SLC1A5	2,11E-35	0,495917	0,989	0,939	4,03E-31	7	SLC1A5
PSMB5	2,37E-35	0,356516	0,992	0,995	4,53E-31	7	PSMB5
HNRNPM	2,57E-35	0,409017	0,973	0,871	4,92E-31	7	HNRNPM
ATP5H	2,70E-35	0,34147	0,989	0,995	5,16E-31	7	ATP5H
TMEM165	3,52E-35	0,402253	1	0,973	6,74E-31	7	TMEM165
MT-ND6	3,78E-35	0,575578	0,973	0,873	7,24E-31	7	MT-ND6
EIF6	9,79E-35	0,383128	0,973	0,964	1,87E-30	7	EIF6
PPP1R12A	9,80E-35	0,373523	0,917	0,788	1,87E-30	7	PPP1R12A
TCP1	1,09E-34	0,365565	0,981	0,932	2,08E-30	7	TCP1
PON2	1,39E-34	0,327188	0,939	0,797	2,66E-30	7	PON2
CTSD1	1,60E-34	0,469014	1	0,98	3,06E-30	7	CTSD
PPP2R1A	1,68E-34	0,329534	0,981	0,948	3,22E-30	7	PPP2R1A
NUCB1	2,57E-34	0,375543	0,973	0,858	4,92E-30	7	NUCB1

COL6A22	3,06E-34	0,554192	1	0,998	5,84E-30	7	COL6A2
SGCB	3,98E-34	0,34776	0,902	0,767	7,61E-30	7	SGCB
MYO62	4,34E-34	0,593775	0,951	0,82	8,30E-30	7	MYO6
NEXN	6,27E-34	0,552515	0,992	0,972	1,20E-29	7	NEXN
RBM8A	6,67E-34	0,359555	0,928	0,922	1,27E-29	7	RBM8A
RDX	8,67E-34	0,390829	0,977	0,943	1,66E-29	7	RDX
AKAP122	9,73E-34	0,84536	0,992	0,977	1,86E-29	7	AKAP12
TAGLN1	1,11E-33	0,919214	1	1	2,12E-29	7	TAGLN
TMEM176I	1,35E-33	0,789751	0,985	0,985	2,59E-29	7	TMEM176B
NOP56	1,50E-33	0,332192	0,883	0,691	2,86E-29	7	NOP56
SSBP1	1,75E-33	0,36411	0,981	0,985	3,35E-29	7	SSBP1
PSMD12	2,16E-33	0,325942	0,856	0,672	4,12E-29	7	PSMD12
C4orf27	2,17E-33	0,311775	0,807	0,562	4,15E-29	7	C4orf27
WLS	2,37E-33	0,40269	0,966	0,855	4,54E-29	7	WLS
PAMR11	3,52E-33	0,448866	0,955	0,871	6,72E-29	7	PAMR1
MTCH2	4,56E-33	0,357132	0,977	0,945	8,71E-29	7	MTCH2
NIT2	4,59E-33	0,320755	0,905	0,779	8,78E-29	7	NIT2
GBP1	4,96E-33	0,494803	0,92	0,795	9,48E-29	7	GBP1
SCFD1	5,48E-33	0,349279	0,89	0,723	1,05E-28	7	SCFD1
IDH3G	6,67E-33	0,317437	0,917	0,828	1,27E-28	7	IDH3G
SQRDL	6,88E-33	0,448856	0,879	0,746	1,32E-28	7	SQRDL
PRPF40A	7,13E-33	0,386885	0,947	0,885	1,36E-28	7	PRPF40A
TUBA1B	7,30E-33	0,49745	0,996	0,995	1,40E-28	7	TUBA1B
PSME1	7,98E-33	0,354881	0,992	0,986	1,53E-28	7	PSME1
HSPA4	9,40E-33	0,392984	0,89	0,738	1,80E-28	7	HSPA4
TSG101	1,00E-32	0,287581	0,909	0,764	1,92E-28	7	TSG101
TERF2IP	1,19E-32	0,343538	0,909	0,819	2,28E-28	7	TERF2IP
NDUFS3	1,24E-32	0,329465	0,936	0,874	2,36E-28	7	NDUFS3
CLN5	1,29E-32	0,287374	0,803	0,554	2,47E-28	7	CLN5
LACTB	1,65E-32	0,473164	0,917	0,83	3,16E-28	7	LACTB
ACSL3	1,74E-32	0,409165	0,981	0,927	3,32E-28	7	ACSL3
P4HB	1,96E-32	0,42171	1	0,995	3,75E-28	7	P4HB
11-sep	2,01E-32	0,440872	0,996	0,984	3,83E-28	7	11-sep
SLC16A31	3,10E-32	0,488241	0,955	0,908	5,92E-28	7	SLC16A3
ASPH1	4,38E-32	0,500742	1	0,985	8,37E-28	7	ASPH
RAB11A	4,48E-32	0,331773	0,973	0,959	8,57E-28	7	RAB11A
MRPS22	5,22E-32	0,290221	0,86	0,688	9,99E-28	7	MRPS22
KDELR2	5,84E-32	0,321398	0,989	0,996	1,12E-27	7	KDELR2
NUDT5	6,10E-32	0,360453	0,936	0,921	1,17E-27	7	NUDT5
FDFT11	6,98E-32	0,404176	0,966	0,918	1,34E-27	7	FDFT1
PRDX5	7,01E-32	0,286393	0,992	0,999	1,34E-27	7	PRDX5
PSMA1	7,15E-32	0,316753	0,989	0,989	1,37E-27	7	PSMA1
DDRGK1	7,94E-32	0,319613	0,924	0,802	1,52E-27	7	DDRGK1
VDAC3	1,09E-31	0,311429	0,966	0,915	2,09E-27	7	VDAC3
NAA20	1,37E-31	0,300641	0,951	0,885	2,62E-27	7	NAA20
NOL7	1,47E-31	0,319681	0,966	0,902	2,82E-27	7	NOL7
VPS26A	1,81E-31	0,302678	0,905	0,773	3,47E-27	7	VPS26A
KARS	2,15E-31	0,359573	0,958	0,892	4,12E-27	7	KARS
RAB7A	4,50E-31	0,326341	0,981	0,989	8,60E-27	7	RAB7A
PRDX6	6,34E-31	0,399877	0,992	0,997	1,21E-26	7	PRDX6

NAGK	6,46E-31	0,320409	0,909	0,765	1,24E-26	7	NAGK
RBBP7	1,26E-30	0,313817	0,936	0,841	2,41E-26	7	RBBP7
ILF2	1,54E-30	0,298311	0,939	0,855	2,95E-26	7	ILF2
MRPL47	1,99E-30	0,328926	0,932	0,877	3,81E-26	7	MRPL47
PPHLN1	2,04E-30	0,30038	0,928	0,763	3,89E-26	7	PPHLN1
CLIC11	2,05E-30	0,32605	1	0,999	3,92E-26	7	CLIC1
CD55	2,33E-30	0,383013	0,848	0,644	4,45E-26	7	CD55
HSPA2	2,90E-30	0,467392	0,777	0,581	5,54E-26	7	HSPA2
CCDC47	2,92E-30	0,268105	0,822	0,608	5,59E-26	7	CCDC47
DCTN3	2,93E-30	0,309721	0,981	0,977	5,61E-26	7	DCTN3
PGM3	3,55E-30	0,287977	0,814	0,608	6,78E-26	7	PGM3
SMARCA1	3,59E-30	0,343132	0,879	0,68	6,86E-26	7	SMARCA1
PSMD11	4,48E-30	0,33066	0,902	0,804	8,57E-26	7	PSMD11
HSD17B4	5,16E-30	0,266789	0,739	0,452	9,87E-26	7	HSD17B4
P3H1	6,37E-30	0,31172	0,833	0,619	1,22E-25	7	P3H1
ATP1A1	6,59E-30	0,333712	0,92	0,792	1,26E-25	7	ATP1A1
SPPL2A	6,72E-30	0,356	0,902	0,751	1,28E-25	7	SPPL2A
SAP18	7,42E-30	0,3185	0,989	0,994	1,42E-25	7	SAP18
TMBIM4	1,14E-29	0,292098	0,928	0,84	2,18E-25	7	TMBIM4
ITFG1	1,32E-29	0,31015	0,883	0,697	2,53E-25	7	ITFG1
TXNDC9	1,33E-29	0,316183	0,875	0,801	2,55E-25	7	TXNDC9
PLPP12	1,51E-29	0,456178	0,947	0,838	2,89E-25	7	PLPP1
MORF4L2	1,78E-29	0,375178	0,996	0,994	3,41E-25	7	MORF4L2
SERPINB61	1,93E-29	0,340409	0,962	0,953	3,69E-25	7	SERPINB6
CTSB	2,05E-29	0,387351	1	0,996	3,92E-25	7	CTSB
LEPROTL1	2,05E-29	0,28876	0,909	0,83	3,93E-25	7	LEPROTL1
RAN	2,85E-29	0,301779	0,992	0,997	5,46E-25	7	RAN
NASP	3,54E-29	0,258741	0,818	0,596	6,77E-25	7	NASP
DHX36	3,71E-29	0,376208	0,932	0,831	7,08E-25	7	DHX36
PIGK	4,16E-29	0,254698	0,735	0,455	7,96E-25	7	PIGK
PBDC1	4,24E-29	0,28109	0,803	0,588	8,11E-25	7	PBDC1
S100A111	4,47E-29	0,331551	1	1	8,56E-25	7	S100A11
PSMB7	4,90E-29	0,298454	1	0,993	9,37E-25	7	PSMB7
NARS	5,05E-29	0,387928	0,966	0,911	9,66E-25	7	NARS
ATP6V1H	5,40E-29	0,323435	0,902	0,757	1,03E-24	7	ATP6V1H
PCK2	1,23E-28	0,336077	0,777	0,539	2,36E-24	7	PCK2
WARS	1,25E-28	0,405897	0,985	0,97	2,38E-24	7	WARS
DNAJC1	2,08E-28	0,35886	0,939	0,867	3,98E-24	7	DNAJC1
SCCPDH	2,27E-28	0,28295	0,833	0,655	4,33E-24	7	SCCPDH
ACOT9	2,37E-28	0,257671	0,807	0,601	4,54E-24	7	ACOT9
BZW1	3,17E-28	0,293243	0,977	0,964	6,06E-24	7	BZW1
HSPB11	3,32E-28	0,31635	0,939	0,896	6,35E-24	7	HSPB11
NDUFV1	3,51E-28	0,304104	0,962	0,921	6,72E-24	7	NDUFV1
VDAC1	4,27E-28	0,292561	1	0,987	8,17E-24	7	VDAC1
CASP1	4,46E-28	0,387861	0,924	0,816	8,53E-24	7	CASP1
CACYBP	5,36E-28	0,274055	0,905	0,806	1,03E-23	7	CACYBP
PPA1	8,71E-28	0,312277	0,989	0,995	1,67E-23	7	PPA1
YWHAQ	9,93E-28	0,267985	0,996	0,992	1,90E-23	7	YWHAQ
CYC1	1,08E-27	0,323921	0,989	0,96	2,06E-23	7	CYC1
ANXA111	1,10E-27	0,292858	0,996	0,992	2,11E-23	7	ANXA11

SPARC	1,23E-27	0,485157	1	0,999	2,36E-23	7	SPARC
WDR1	1,40E-27	0,426127	0,992	0,989	2,68E-23	7	WDR1
ARL3	1,48E-27	0,298164	0,92	0,879	2,84E-23	7	ARL3
DCN2	1,76E-27	0,585527	1	0,999	3,36E-23	7	DCN
RCN1	2,02E-27	0,358947	0,996	0,989	3,87E-23	7	RCN1
CHMP5	2,08E-27	0,298516	0,97	0,945	3,99E-23	7	CHMP5
PARK7	2,47E-27	0,292547	1	0,999	4,72E-23	7	PARK7
ALG5	2,53E-27	0,266137	0,917	0,803	4,85E-23	7	ALG5
SARAF	2,70E-27	0,310344	1	0,983	5,16E-23	7	SARAF
HSD17B12	2,85E-27	0,34162	0,924	0,858	5,45E-23	7	HSD17B12
SRP72	3,64E-27	0,292962	0,936	0,881	6,97E-23	7	SRP72
SCARB2	4,47E-27	0,353533	0,977	0,946	8,54E-23	7	SCARB2
COMT	4,47E-27	0,292504	0,985	0,979	8,54E-23	7	COMT
EFEMP2	4,49E-27	0,346533	0,996	0,995	8,59E-23	7	EFEMP2
DCTN6	4,87E-27	0,288876	0,905	0,809	9,30E-23	7	DCTN6
COLEC10	4,90E-27	0,386841	0,924	0,823	9,37E-23	7	COLEC10
RCN3	4,94E-27	0,399474	0,833	0,657	9,45E-23	7	RCN3
PVRL2	5,76E-27	0,354474	0,947	0,866	1,10E-22	7	PVRL2
LXN2	5,93E-27	0,588121	0,966	0,936	1,13E-22	7	LXN
PRKDC	6,29E-27	0,387784	0,932	0,826	1,20E-22	7	PRKDC
VWA5A	1,06E-26	0,328395	0,856	0,713	2,03E-22	7	VWA5A
CST32	1,08E-26	0,312339	1	0,999	2,07E-22	7	CST3
CAPG	1,10E-26	0,322113	0,856	0,676	2,11E-22	7	CAPG
DAD1	1,14E-26	0,311329	0,981	0,992	2,18E-22	7	DAD1
SPCS1	1,16E-26	0,284107	0,981	0,991	2,23E-22	7	SPCS1
SUCLA2	1,91E-26	0,257966	0,75	0,515	3,65E-22	7	SUCLA2
PPIL4	2,68E-26	0,265738	0,788	0,577	5,13E-22	7	PPIL4
ALDH7A1	2,71E-26	0,253486	0,731	0,467	5,18E-22	7	ALDH7A1
IKBIP	2,94E-26	0,282633	0,92	0,811	5,62E-22	7	IKBIP
TFPI2	2,95E-26	1,012955	0,943	0,937	5,64E-22	7	TFPI2
LAP3	2,98E-26	0,288841	0,841	0,672	5,70E-22	7	LAP3
ERGIC2	3,09E-26	0,306338	0,947	0,907	5,92E-22	7	ERGIC2
SRRM1	4,55E-26	0,330845	0,97	0,921	8,70E-22	7	SRRM1
CDC37	5,13E-26	0,304445	0,977	0,967	9,81E-22	7	CDC37
SMARCA5	5,38E-26	0,356599	0,939	0,845	1,03E-21	7	SMARCA5
SNX2	5,93E-26	0,260997	0,864	0,714	1,13E-21	7	SNX2
PDIA4	7,23E-26	0,327886	0,943	0,783	1,38E-21	7	PDIA4
TFG	7,30E-26	0,286618	0,97	0,918	1,40E-21	7	TFG
TWF2	8,67E-26	0,302763	0,905	0,827	1,66E-21	7	TWF2
EEA1	8,69E-26	0,399138	0,973	0,913	1,66E-21	7	EEA1
TCEAL4	9,77E-26	0,384591	0,992	0,987	1,87E-21	7	TCEAL4
IFI16	1,03E-25	0,363133	0,981	0,897	1,98E-21	7	IFI16
NDUFB4	1,04E-25	0,292837	0,985	0,998	2,00E-21	7	NDUFB4
SP100	1,16E-25	0,283992	0,909	0,779	2,22E-21	7	SP100
TECR1	1,16E-25	0,296397	0,928	0,868	2,23E-21	7	TECR
PIR	1,44E-25	0,298164	0,86	0,715	2,76E-21	7	PIR
DLD	1,67E-25	0,25635	0,833	0,65	3,18E-21	7	DLD
LARP7	1,78E-25	0,272263	0,864	0,724	3,41E-21	7	LARP7
MFGE8	2,02E-25	0,473257	1	0,965	3,86E-21	7	MFGE8
APMAP	2,86E-25	0,297163	0,879	0,774	5,46E-21	7	APMAP

HSD17B10	3,40E-25	0,290456	0,947	0,916	6,51E-21	7	HSD17B10
UBE2L3	3,41E-25	0,302804	0,989	0,989	6,53E-21	7	UBE2L3
TTC14	4,49E-25	0,312832	0,826	0,662	8,58E-21	7	TTC14
COPG1	4,96E-25	0,271306	0,894	0,762	9,49E-21	7	COPG1
PPIG	5,08E-25	0,282679	0,936	0,894	9,72E-21	7	PPIG
GNAI3	5,12E-25	0,296612	0,936	0,861	9,79E-21	7	GNAI3
ESD	5,14E-25	0,273959	0,992	0,996	9,83E-21	7	ESD
TEX264	6,31E-25	0,254795	0,928	0,855	1,21E-20	7	TEX264
ERP292	6,57E-25	0,324206	0,985	0,994	1,26E-20	7	ERP29
FBLN5	7,01E-25	0,471927	0,966	0,937	1,34E-20	7	FBLN5
ASAH11	8,24E-25	0,374653	0,966	0,944	1,58E-20	7	ASAH1
DERL2	9,25E-25	0,274099	0,852	0,709	1,77E-20	7	DERL2
PA2G4	9,52E-25	0,293047	0,951	0,926	1,82E-20	7	PA2G4
ECHDC1	9,53E-25	0,266837	0,924	0,885	1,82E-20	7	ECHDC1
GBP21	1,03E-24	0,3885	0,955	0,833	1,98E-20	7	GBP2
TMED10	1,41E-24	0,264557	0,981	0,966	2,69E-20	7	TMED10
ATP5O	1,54E-24	0,277463	0,981	0,996	2,95E-20	7	ATP5O
PLS3	1,54E-24	0,331184	0,932	0,804	2,95E-20	7	PLS3
COPS5	1,59E-24	0,254934	0,867	0,709	3,04E-20	7	COPS5
MALSU1	1,62E-24	0,263308	0,86	0,759	3,09E-20	7	MALSU1
PGRMC2	1,71E-24	0,329274	0,97	0,944	3,27E-20	7	PGRMC2
NDUFA8	1,73E-24	0,27885	0,943	0,934	3,30E-20	7	NDUFA8
C14orf166	1,88E-24	0,260852	0,996	0,996	3,60E-20	7	C14orf166
UFD1L	1,91E-24	0,290109	0,92	0,851	3,65E-20	7	UFD1L
PSAT1	2,01E-24	0,41745	0,932	0,841	3,84E-20	7	PSAT1
RSU1	2,14E-24	0,313139	0,947	0,9	4,09E-20	7	RSU1
SNW1	2,58E-24	0,27078	0,917	0,781	4,93E-20	7	SNW1
SNX17	2,70E-24	0,27134	0,973	0,922	5,16E-20	7	SNX17
TBCB	3,48E-24	0,283042	0,955	0,956	6,66E-20	7	TBCB
TXN	4,64E-24	0,394627	1	1	8,88E-20	7	TXN
PGAM1	5,65E-24	0,283015	1	0,994	1,08E-19	7	PGAM1
CD46	5,82E-24	0,307422	0,936	0,847	1,11E-19	7	CD46
IDH3B	6,75E-24	0,266892	0,852	0,732	1,29E-19	7	IDH3B
TRIP6	6,87E-24	0,317754	0,996	0,968	1,31E-19	7	TRIP6
PSMB9	7,61E-24	0,328106	0,909	0,835	1,46E-19	7	PSMB9
JKAMP	8,05E-24	0,286659	0,86	0,72	1,54E-19	7	JKAMP
RRBP1	9,71E-24	0,33558	1	0,988	1,86E-19	7	RRBP1
PPIC1	1,03E-23	0,298301	0,966	0,942	1,96E-19	7	PPIC
MRPS5	1,24E-23	0,26815	0,883	0,779	2,37E-19	7	MRPS5
MRPL13	1,35E-23	0,285796	0,917	0,881	2,59E-19	7	MRPL13
PMPCB	1,48E-23	0,279363	0,936	0,848	2,82E-19	7	PMPCB
SDHB	1,64E-23	0,2789	0,902	0,879	3,13E-19	7	SDHB
RAB1A	1,67E-23	0,26954	0,966	0,981	3,20E-19	7	RAB1A
RTCB	1,68E-23	0,297295	0,902	0,837	3,21E-19	7	RTCB
STIP1	1,69E-23	0,263296	0,837	0,649	3,24E-19	7	STIP1
PPA2	1,99E-23	0,277595	0,83	0,716	3,80E-19	7	PPA2
RBM25	2,42E-23	0,362956	0,966	0,911	4,63E-19	7	RBM25
SFT2D1	2,47E-23	0,270448	0,913	0,867	4,73E-19	7	SFT2D1
ACADVL	3,30E-23	0,299437	0,867	0,726	6,31E-19	7	ACADVL
YIPF3	5,28E-23	0,285558	0,955	0,947	1,01E-18	7	YIPF3

CLPTM1	5,31E-23	0,251286	0,871	0,718	1,02E-18	7	CLPTM1
TUBB	5,33E-23	0,481173	1	0,999	1,02E-18	7	TUBB
BRIX11	5,54E-23	0,427104	0,905	0,809	1,06E-18	7	BRIX1
APP1	6,81E-23	0,469655	0,996	0,976	1,30E-18	7	APP
PWP1	6,96E-23	0,26579	0,905	0,798	1,33E-18	7	PWP1
IDI11	8,21E-23	0,454273	0,955	0,888	1,57E-18	7	IDI1
DNAJA2	1,04E-22	0,2594	0,894	0,806	2,00E-18	7	DNAJA2
TXNDC15	1,15E-22	0,255035	0,852	0,715	2,20E-18	7	TXNDC15
EIF2S2	1,45E-22	0,274266	1	0,995	2,77E-18	7	EIF2S2
SAR1A	1,48E-22	0,295577	0,955	0,93	2,84E-18	7	SAR1A
TPI11	1,53E-22	0,266757	0,992	0,996	2,93E-18	7	TPI1
SEC63	1,66E-22	0,275471	0,962	0,936	3,18E-18	7	SEC63
GPNMB2	1,83E-22	0,526451	0,973	0,93	3,51E-18	7	GPNMB
NDUFA4	2,28E-22	0,311597	0,996	1	4,35E-18	7	NDUFA4
TUBB61	2,78E-22	0,45705	0,989	0,971	5,32E-18	7	TUBB6
ISG15	3,32E-22	0,302897	0,962	0,941	6,35E-18	7	ISG15
GCLM1	3,57E-22	0,506231	0,977	0,964	6,82E-18	7	GCLM
TUBA1C	3,66E-22	0,414438	0,966	0,959	7,00E-18	7	TUBA1C
PSG4	5,00E-22	0,462302	0,985	0,954	9,57E-18	7	PSG4
SPG20	5,70E-22	0,292521	0,939	0,857	1,09E-17	7	SPG20
PLIN21	5,75E-22	0,365785	0,955	0,911	1,10E-17	7	PLIN2
LMAN21	6,50E-22	0,267272	0,985	0,972	1,24E-17	7	LMAN2
FHL22	6,81E-22	0,365548	1	0,996	1,30E-17	7	FHL2
EZR	8,35E-22	0,281668	0,837	0,661	1,60E-17	7	EZR
PSMC5	1,05E-21	0,257077	0,962	0,941	2,01E-17	7	PSMC5
DYNC1H1	1,13E-21	0,387743	0,958	0,926	2,16E-17	7	DYNC1H1
SBDS	1,21E-21	0,291133	0,985	0,967	2,31E-17	7	SBDS
LARS	1,26E-21	0,289295	0,898	0,776	2,41E-17	7	LARS
BCLAF1	1,33E-21	0,30498	0,928	0,87	2,54E-17	7	BCLAF1
MFSD10	1,48E-21	0,261075	0,928	0,85	2,83E-17	7	MFSD10
AARS	1,51E-21	0,25746	0,848	0,691	2,89E-17	7	AARS
SARS	1,57E-21	0,276642	0,985	0,979	3,01E-17	7	SARS
NDUFA12	1,68E-21	0,268237	0,917	0,929	3,20E-17	7	NDUFA12
ACO1	3,86E-21	0,296079	0,939	0,872	7,38E-17	7	ACO1
UCHL3	5,91E-21	0,251353	0,867	0,779	1,13E-16	7	UCHL3
CPQ	6,42E-21	0,253979	0,727	0,514	1,23E-16	7	CPQ
KIAA1033	6,43E-21	0,324459	0,803	0,668	1,23E-16	7	KIAA1033
DARS	6,82E-21	0,262317	0,913	0,791	1,30E-16	7	DARS
THRAP3	7,15E-21	0,251497	0,955	0,912	1,37E-16	7	THRAP3
HNRNPD	7,28E-21	0,283691	0,955	0,923	1,39E-16	7	HNRNPD
SNRNP70	8,43E-21	0,291479	0,943	0,877	1,61E-16	7	SNRNP70
USP14	1,62E-20	0,269612	0,871	0,772	3,10E-16	7	USP14
TXNRD12	1,85E-20	0,412471	1	0,997	3,54E-16	7	TXNRD1
WBSCR22	2,14E-20	0,25065	0,902	0,812	4,10E-16	7	WBSCR22
UGP2	2,20E-20	0,281789	0,92	0,865	4,20E-16	7	UGP2
PTS	3,40E-20	0,284381	0,924	0,902	6,49E-16	7	PTS
GLA	7,17E-20	0,255628	0,739	0,551	1,37E-15	7	GLA
TOP1	7,97E-20	0,285305	0,932	0,813	1,52E-15	7	TOP1
NCSTN	1,01E-19	0,254951	0,803	0,658	1,94E-15	7	NCSTN
CYB5R3	1,72E-19	0,265055	1	0,991	3,28E-15	7	CYB5R3

NRBP1	2,14E-19	0,272097	0,905	0,841	4,10E-15	7	NRBP1
TRAPPC2L	2,17E-19	0,280315	0,939	0,977	4,14E-15	7	TRAPPC2L
SEC31A	2,26E-19	0,340263	0,981	0,949	4,32E-15	7	SEC31A
ME1	2,42E-19	0,322742	0,883	0,829	4,63E-15	7	ME1
UBA1	2,56E-19	0,263546	0,89	0,789	4,90E-15	7	UBA1
ITM2C	4,29E-19	0,277851	0,955	0,922	8,21E-15	7	ITM2C
CFD3	4,31E-19	0,64858	0,97	0,949	8,24E-15	7	CFD
HNRNPF	4,47E-19	0,252096	0,977	0,953	8,55E-15	7	HNRNPF
TUBA4A	7,68E-19	0,257043	0,723	0,545	1,47E-14	7	TUBA4A
RARRES33	9,27E-19	0,382532	0,973	0,949	1,77E-14	7	RARRES3
LAMC11	1,18E-18	0,5987	0,989	0,955	2,25E-14	7	LAMC1
MANF	1,20E-18	0,267809	0,958	0,873	2,29E-14	7	MANF
EIF3A	1,30E-18	0,396743	0,989	0,983	2,48E-14	7	EIF3A
NDN	1,35E-18	0,365352	0,799	0,765	2,57E-14	7	NDN
KDELR3	1,46E-18	0,265767	0,902	0,865	2,79E-14	7	KDELR3
AKR7A2	1,48E-18	0,260091	0,951	0,887	2,83E-14	7	AKR7A2
ATP5G31	1,63E-18	0,276845	0,981	0,999	3,12E-14	7	ATP5G3
ATPIF1	2,32E-18	0,250241	0,985	0,991	4,44E-14	7	ATPIF1
DNAJC7	2,74E-18	0,258078	0,939	0,898	5,24E-14	7	DNAJC7
DHCR71	2,81E-18	0,282146	0,852	0,705	5,38E-14	7	DHCR7
MYH10	3,06E-18	0,251818	0,78	0,632	5,85E-14	7	MYH10
WBP5	3,09E-18	0,256641	0,989	0,996	5,91E-14	7	WBP5
CAPN1	3,21E-18	0,257918	0,902	0,846	6,15E-14	7	CAPN1
NEU11	4,75E-18	0,268067	0,913	0,857	9,09E-14	7	NEU1
IDH1	5,81E-18	0,285719	0,981	0,937	1,11E-13	7	IDH1
MSMO11	8,61E-18	0,329342	0,871	0,798	1,65E-13	7	MSMO1
FILIP1L	1,12E-17	0,398786	0,973	0,95	2,15E-13	7	FILIP1L
CERCAM	1,14E-17	0,277309	0,92	0,846	2,17E-13	7	CERCAM
ADAM9	1,54E-17	0,387603	0,981	0,967	2,95E-13	7	ADAM9
CAP1	1,55E-17	0,310314	0,996	0,992	2,96E-13	7	CAP1
MAP1B1	1,76E-17	0,548982	0,992	0,991	3,37E-13	7	MAP1B
EDNRB1	1,87E-17	0,262219	0,712	0,526	3,57E-13	7	EDNRB
02-sep	2,17E-17	0,267547	0,977	0,98	4,16E-13	7	02-sep
XBP1	2,74E-17	0,258158	0,966	0,921	5,24E-13	7	XBP1
PDCD6IP	3,92E-17	0,286196	0,879	0,795	7,49E-13	7	PDCD6IP
COL1A21	5,00E-17	0,530356	1	0,999	9,55E-13	7	COL1A2
SNAPC1	6,50E-17	0,330564	0,723	0,577	1,24E-12	7	SNAPC1
MAP1A	7,31E-17	0,327307	0,985	0,949	1,40E-12	7	MAP1A
EBP1	8,05E-17	0,268562	0,811	0,659	1,54E-12	7	EBP
GSPT1	1,63E-16	0,255422	0,902	0,861	3,12E-12	7	GSPT1
BST1	1,73E-16	0,280355	0,833	0,718	3,31E-12	7	BST1
STOM1	2,73E-16	0,251825	0,996	0,996	5,23E-12	7	STOM
GFPT1	2,79E-16	0,263538	0,807	0,71	5,33E-12	7	GFPT1
THOC2	3,73E-16	0,258965	0,845	0,719	7,13E-12	7	THOC2
HNRNPA3	4,94E-16	0,272654	0,992	0,969	9,44E-12	7	HNRNPA3
CAPN2	5,62E-16	0,289712	0,989	0,984	1,07E-11	7	CAPN2
CANX	5,72E-16	0,372316	1	0,983	1,09E-11	7	CANX
PARVA	6,28E-16	0,264305	0,977	0,952	1,20E-11	7	PARVA
OLFML3	7,60E-16	0,284724	0,879	0,767	1,45E-11	7	OLFML3
DPP4	8,26E-16	0,264293	0,833	0,711	1,58E-11	7	DPP4

PMP22	8,51E-16	0,297342	0,989	0,979	1,63E-11	7	PMP22
LAMB11	8,90E-16	0,354769	0,928	0,881	1,70E-11	7	LAMB1
PDLIM71	8,98E-16	0,291945	1	0,995	1,72E-11	7	PDLIM7
CAND1	9,72E-16	0,373218	0,947	0,885	1,86E-11	7	CAND1
PRKCSH	1,33E-15	0,285596	0,981	0,944	2,55E-11	7	PRKCSH
CSRP21	1,89E-15	0,346762	0,89	0,816	3,62E-11	7	CSRP2
ATRX	2,08E-15	0,383324	0,955	0,919	3,97E-11	7	ATRX
LOX	3,53E-15	0,396411	0,966	0,932	6,76E-11	7	LOX
CADM1	4,33E-15	0,250851	0,723	0,558	8,28E-11	7	CADM1
NME11	4,49E-15	0,254527	0,951	0,939	8,59E-11	7	NME1
PDPN1	4,50E-15	0,293853	0,894	0,857	8,61E-11	7	PDPN
NPTN	5,03E-15	0,300136	0,894	0,817	9,62E-11	7	NPTN
TNFRSF11B	5,44E-15	0,707045	0,932	0,9	1,04E-10	7	TNFRSF11B
TGFBI2	7,49E-15	0,327027	0,996	0,99	1,43E-10	7	TGFBI
PALLD	8,87E-15	0,38838	1	0,996	1,70E-10	7	PALLD
TKT2	1,60E-14	0,268694	1	0,998	3,06E-10	7	TKT
ITGA1	1,96E-14	0,473719	0,917	0,853	3,76E-10	7	ITGA1
PRSS23	2,16E-14	0,299509	0,917	0,871	4,12E-10	7	PRSS23
NID2	2,47E-14	0,331767	0,852	0,8	4,72E-10	7	NID2
GOLGB1	2,60E-14	0,311236	0,917	0,84	4,98E-10	7	GOLGB1
USP47	2,62E-14	0,269108	0,811	0,703	5,01E-10	7	USP47
NAMPT1	2,89E-14	0,259481	0,928	0,862	5,52E-10	7	NAMPT
PGD	4,85E-14	0,274848	0,985	0,97	9,27E-10	7	PGD
HIF1A	5,34E-14	0,348674	0,966	0,918	1,02E-09	7	HIF1A
FSTL11	5,94E-14	0,493044	1	0,994	1,14E-09	7	FSTL1
KTN1	1,20E-13	0,32158	1	0,993	2,30E-09	7	KTN1
LMO72	1,51E-13	0,430533	0,977	0,946	2,89E-09	7	LMO7
GJA11	1,55E-13	0,333127	0,917	0,835	2,96E-09	7	GJA1
ATP2B1	2,12E-13	0,340596	0,996	0,985	4,05E-09	7	ATP2B1
LIMCH1	4,39E-13	0,288354	0,977	0,941	8,40E-09	7	LIMCH1
IL32	1,00E-12	0,378136	0,792	0,754	1,92E-08	7	IL32
DDB1	1,21E-12	0,274893	0,845	0,783	2,32E-08	7	DDB1
LOXL2	1,60E-12	0,424912	0,856	0,782	3,07E-08	7	LOXL2
F31	1,75E-12	0,549336	0,871	0,76	3,35E-08	7	F3
SEC23A	2,45E-12	0,285295	0,947	0,874	4,68E-08	7	SEC23A
CKAP4	4,14E-12	0,309671	0,989	0,986	7,92E-08	7	CKAP4
KLC1	6,16E-12	0,259923	0,845	0,777	1,18E-07	7	KLC1
IGFBP2	6,53E-12	0,562721	0,962	0,952	1,25E-07	7	IGFBP2
VMP1	8,20E-12	0,307843	0,958	0,915	1,57E-07	7	VMP1
EIF5B	1,02E-11	0,31558	0,973	0,972	1,95E-07	7	EIF5B
STC21	1,81E-11	0,406122	0,92	0,873	3,46E-07	7	STC2
MAP4	2,69E-11	0,264081	0,981	0,977	5,14E-07	7	MAP4
S100A101	2,85E-11	0,265209	1	0,997	5,45E-07	7	S100A10
GDF151	4,70E-11	0,479532	0,898	0,844	8,99E-07	7	GDF15
TPBG	7,04E-11	0,262578	0,928	0,853	1,35E-06	7	TPBG
ARHGAP29	9,61E-11	0,277194	0,92	0,862	1,84E-06	7	ARHGAP29
LAMA41	1,01E-10	0,329672	0,966	0,919	1,94E-06	7	LAMA4
MMP142	1,37E-10	0,267528	0,973	0,951	2,63E-06	7	MMP14
ACTR2	1,82E-10	0,258856	0,973	0,945	3,47E-06	7	ACTR2
MYADM	1,89E-10	0,26795	0,977	0,941	3,62E-06	7	MYADM

CYR611	2,00E-10	0,501226	0,985	0,964	3,83E-06	7	CYR61
SQLE1	2,17E-10	0,293671	0,973	0,924	4,14E-06	7	SQLE
SLC2A1	2,38E-10	0,250471	0,564	0,41	4,54E-06	7	SLC2A1
IQGAP1	2,99E-10	0,277522	0,913	0,873	5,72E-06	7	IQGAP1
COL1A11	3,14E-10	0,406127	1	0,997	6,00E-06	7	COL1A1
PAPSS22	3,56E-10	0,369849	0,951	0,919	6,80E-06	7	PAPSS2
SCAF11	6,81E-10	0,271264	0,951	0,923	1,30E-05	7	SCAF11
ARID5B	1,96E-09	0,270382	1	0,992	3,75E-05	7	ARID5B
P3H21	2,80E-09	0,293791	0,795	0,706	5,36E-05	7	P3H2
CTGF1	3,44E-09	0,514248	0,92	0,905	6,58E-05	7	CTGF
TNPO1	5,59E-09	0,309139	0,958	0,956	0,000107	7	TNPO1
SVEP1	6,75E-09	0,343506	0,883	0,835	0,000129	7	SVEP1
PCYOX11	9,03E-09	0,261189	0,947	0,888	0,000173	7	PCYOX1
IARS	1,21E-08	0,266913	0,932	0,899	0,000231	7	IARS
GLS	1,56E-08	0,344894	0,864	0,804	0,000297	7	GLS
TMEM471	1,75E-08	0,355166	0,905	0,832	0,000334	7	TMEM47
STAT12	1,87E-08	0,261874	0,97	0,948	0,000358	7	STAT1
RARRES23	1,92E-08	0,374014	0,996	0,985	0,000368	7	RARRES2
OSMR	2,01E-08	0,298074	0,845	0,775	0,000385	7	OSMR
MMP22	2,02E-08	0,257199	1	0,991	0,000386	7	MMP2
FLNA	2,64E-08	0,633547	0,992	0,994	0,000504	7	FLNA
ITGAV1	2,72E-08	0,373734	0,936	0,882	0,00052	7	ITGAV
SMTN	6,43E-08	0,267909	0,742	0,643	0,00123	7	SMTN
SBSPON	1,35E-07	0,285005	0,913	0,879	0,002575	7	SBSPON
TFRC	1,36E-07	0,37965	0,947	0,911	0,002592	7	TFRC
MYLK1	1,39E-07	0,341343	1	0,999	0,002667	7	MYLK
PTGDS2	1,79E-07	0,549015	0,902	0,9	0,003414	7	PTGDS
SPON22	2,35E-07	0,320908	0,992	0,997	0,004498	7	SPON2
TMEM176/	2,81E-07	0,402043	0,962	0,967	0,00538	7	TMEM176A
IGFBP32	3,01E-07	0,308563	0,837	0,77	0,00575	7	IGFBP3
COL3A12	3,21E-07	0,368693	1	0,992	0,006144	7	COL3A1
SLC38A1	5,02E-07	0,289564	0,973	0,952	0,009606	7	SLC38A1
WNT5A1	5,22E-07	0,325332	0,852	0,815	0,00999	7	WNT5A
PRNP1	9,49E-07	0,358602	0,989	0,98	0,018147	7	PRNP
CES1	1,04E-06	0,28628	0,265	0,158	0,019938	7	CES1
DST1	1,81E-06	0,350062	1	0,989	0,034694	7	DST
TRPA1	4,95E-06	0,318182	0,867	0,827	0,094718	7	TRPA1
MACF1	5,32E-06	0,351524	0,955	0,93	0,101762	7	MACF1
RDH101	6,48E-06	0,302679	0,962	0,945	0,12395	7	RDH10
COL6A11	9,59E-06	0,335603	1	0,996	0,183454	7	COL6A1
ADAMTS1	9,98E-06	0,286317	0,883	0,839	0,190831	7	ADAMTS1
MGP2	1,40E-05	0,33269	0,867	0,83	0,268126	7	MGP
THBS11	1,61E-05	0,535245	0,992	0,981	0,307027	7	THBS1
THBS21	1,87E-05	0,501583	0,939	0,928	0,357375	7	THBS2
SLC7A112	2,09E-05	0,279058	0,848	0,815	0,400474	7	SLC7A11
VCL	2,49E-05	0,354986	0,989	0,968	0,475699	7	VCL
CTSK2	5,81E-05	0,318236	0,932	0,902	1	7	CTSK
FLNB	7,09E-05	0,262367	0,826	0,773	1	7	FLNB
CLTC	7,20E-05	0,324727	0,958	0,957	1	7	CLTC
FN12	0,000122	0,494728	1	0,996	1	7	FN1

N4BP2L21	0,000144	0,251509	0,992	0,972	1	7	N4BP2L2
KRT182	0,00016	0,487556	0,799	0,778	1	7	KRT18
DKK11	0,000234	0,260181	0,761	0,687	1	7	DKK1
CCND12	0,000275	0,251676	0,955	0,968	1	7	CCND1
CRIM11	0,000281	0,349094	0,902	0,873	1	7	CRIM1
FBN11	0,000299	0,29759	0,902	0,876	1	7	FBN1
COL12A1	0,000327	0,280683	0,958	0,943	1	7	COL12A1
COL5A21	0,000669	0,355102	0,989	0,975	1	7	COL5A2
SULF12	0,000992	0,269715	0,754	0,727	1	7	SULF1
A2M1	0,001699	0,337902	0,386	0,313	1	7	A2M
SLC38A21	0,0067	0,285901	0,981	0,973	1	7	SLC38A2
IGF21	3,48E-56	0,74684	0,995	0,977	6,66E-52	8	IGF2
FLNA1	7,62E-55	0,587664	1	0,994	1,46E-50	8	FLNA
RPLP11	1,30E-48	0,321457	1	1	2,48E-44	8	RPLP1
FSTL12	1,20E-46	0,591353	0,995	0,994	2,30E-42	8	FSTL1
CAMK2N11	2,51E-42	0,51076	0,995	0,992	4,80E-38	8	CAMK2N1
RALGPS2	2,28E-39	0,42223	0,977	0,869	4,35E-35	8	RALGPS2
PRRX2	9,60E-38	0,431543	0,875	0,605	1,84E-33	8	PRRX2
GTF2I	1,08E-37	0,44555	0,995	0,985	2,06E-33	8	GTF2I
NR2F2	3,91E-37	0,470369	0,991	0,947	7,48E-33	8	NR2F2
S100A4	4,61E-37	0,608074	0,963	0,772	8,82E-33	8	S100A4
VCL1	6,96E-35	0,405723	1	0,967	1,33E-30	8	VCL
TIMP22	7,29E-35	0,454774	0,995	0,991	1,39E-30	8	TIMP2
SBSPON1	2,36E-34	0,623948	0,968	0,875	4,51E-30	8	SBSPON
IMPA2	2,89E-34	0,327423	0,722	0,377	5,53E-30	8	IMPA2
CARD16	9,42E-34	0,47393	0,912	0,66	1,80E-29	8	CARD16
S1PR3	3,22E-33	0,382902	0,921	0,783	6,15E-29	8	S1PR3
BRI3	3,80E-33	0,423546	0,995	0,993	7,26E-29	8	BRI3
PRRX11	4,94E-33	0,391852	0,94	0,673	9,44E-29	8	PRRX1
IGFBP21	3,59E-32	0,550142	0,986	0,95	6,87E-28	8	IGFBP2
C12orf75	3,78E-32	0,552427	1	0,996	7,23E-28	8	C12orf75
EPHB6	4,88E-32	0,284965	0,708	0,344	9,34E-28	8	EPHB6
IGFBP4	1,47E-31	0,405636	1	0,998	2,82E-27	8	IGFBP4
TFRC1	1,50E-31	0,461648	0,968	0,91	2,87E-27	8	TFRC
MYADM1	2,58E-31	0,404369	0,981	0,941	4,94E-27	8	MYADM
NFASC	3,33E-31	0,360372	0,935	0,721	6,36E-27	8	NFASC
ITGAV2	8,89E-31	0,438504	0,981	0,88	1,70E-26	8	ITGAV
ADGRD1	3,06E-30	0,258428	0,833	0,507	5,84E-26	8	ADGRD1
RPS21	3,41E-30	0,320779	1	1	6,52E-26	8	RPS2
AHNAK	4,78E-30	0,352533	1	0,989	9,13E-26	8	AHNAK
MYH9	1,58E-29	0,344283	0,995	0,992	3,02E-25	8	MYH9
TP53I111	2,45E-29	0,416278	0,963	0,902	4,68E-25	8	TP53I11
ANTXR1	1,19E-28	0,388625	0,981	0,952	2,28E-24	8	ANTXR1
GNG121	1,61E-28	0,368195	0,981	0,975	3,08E-24	8	GNG12
LPP	1,07E-27	0,350743	0,995	0,962	2,04E-23	8	LPP
COL6A12	4,44E-27	0,354362	1	0,996	8,48E-23	8	COL6A1
FHL11	6,02E-27	0,379083	0,977	0,874	1,15E-22	8	FHL1
THBS22	7,64E-27	0,373347	0,972	0,926	1,46E-22	8	THBS2
FBN21	1,40E-26	0,8759	0,866	0,761	2,68E-22	8	FBN2
OST41	4,56E-26	0,288102	1	1	8,72E-22	8	OST4

ACTN4	8,75E-25	0,361307	0,986	0,949	1,67E-20	8	ACTN4
FAM20C	1,39E-24	0,326689	0,949	0,885	2,66E-20	8	FAM20C
MIR4458H(	3,49E-24	0,25438	0,866	0,592	6,67E-20	8	MIR4458HG
TPM4	5,80E-24	0,280341	1	1	1,11E-19	8	TPM4
FAT1	8,52E-23	0,30166	0,787	0,556	1,63E-18	8	FAT1
FKBP9	1,41E-22	0,29215	0,949	0,814	2,70E-18	8	FKBP9
WFDC1	2,84E-22	0,477006	0,968	0,889	5,43E-18	8	WFDC1
LASP11	3,64E-22	0,329804	0,981	0,934	6,97E-18	8	LASP1
CD81	4,33E-22	0,306443	0,981	0,967	8,28E-18	8	CD81
RECK	5,87E-22	0,308467	0,921	0,841	1,12E-17	8	RECK
SNTB2	2,15E-21	0,294331	0,963	0,872	4,11E-17	8	SNTB2
KLF6	3,05E-21	0,298422	0,977	0,877	5,82E-17	8	KLF6
PTRF	4,62E-21	0,260107	1	1	8,83E-17	8	PTRF
APOL6	9,43E-21	0,272633	0,963	0,816	1,80E-16	8	APOL6
COL5A11	1,65E-20	0,26247	0,944	0,838	3,16E-16	8	COL5A1
NFIX1	4,10E-20	0,257206	0,963	0,853	7,84E-16	8	NFIX
PLAC91	7,83E-20	0,28934	0,903	0,737	1,50E-15	8	PLAC9
CBX6	1,44E-19	0,317599	0,981	0,903	2,75E-15	8	CBX6
GNAQ	2,16E-19	0,250138	0,907	0,751	4,12E-15	8	GNAQ
VAT11	4,02E-19	0,298927	0,991	0,986	7,69E-15	8	VAT1
FAM63B	4,21E-19	0,268368	0,921	0,767	8,05E-15	8	FAM63B
CRIM12	6,72E-19	0,310542	0,954	0,869	1,29E-14	8	CRIM1
GLIPR2	8,22E-19	0,307343	0,94	0,866	1,57E-14	8	GLIPR2
MEG31	1,26E-18	0,285866	0,981	0,972	2,41E-14	8	MEG3
ZFP36L22	5,61E-18	0,316041	0,995	0,965	1,07E-13	8	ZFP36L2
SLC38A22	9,00E-18	0,282129	0,986	0,972	1,72E-13	8	SLC38A2
CLMP	1,18E-17	0,254119	0,995	0,978	2,25E-13	8	CLMP
LMO7-AS1:	1,70E-17	0,265396	0,903	0,788	3,24E-13	8	LMO7-AS1
NABP11	4,09E-17	0,317217	0,991	0,972	7,83E-13	8	NABP1
RHOB	4,91E-17	0,259963	0,819	0,628	9,40E-13	8	RHOB
USP532	9,15E-17	0,295079	0,921	0,793	1,75E-12	8	USP53
SVEP11	1,05E-16	0,269045	0,935	0,831	2,01E-12	8	SVEP1
MLXIP2	1,10E-16	0,257775	0,991	0,935	2,10E-12	8	MLXIP
AKAP123	1,11E-16	0,26396	1	0,977	2,12E-12	8	AKAP12
CRYAB	1,34E-16	0,268354	0,829	0,601	2,56E-12	8	CRYAB
HSPB61	2,28E-16	0,302078	0,917	0,841	4,37E-12	8	HSPB6
CTTN1	2,33E-16	0,370535	0,991	0,975	4,45E-12	8	CTTN
MYLK2	2,61E-16	0,399358	0,995	1	5,00E-12	8	MYLK
GREM11	1,75E-14	0,326006	0,884	0,762	3,35E-10	8	GREM1
B4GALT11	3,90E-14	0,285228	0,819	0,601	7,45E-10	8	B4GALT1
PCDH7	5,22E-14	0,383991	0,657	0,474	9,99E-10	8	PCDH7
ATP2B11	2,14E-13	0,267414	1	0,984	4,10E-09	8	ATP2B1
LBH1	7,52E-13	0,300085	0,981	0,947	1,44E-08	8	LBH
SBF2-AS12	2,01E-11	0,324081	0,972	0,946	3,84E-07	8	SBF2-AS1
OSR21	3,20E-10	0,323258	0,861	0,737	6,12E-06	8	OSR2
ADAMTS11	4,68E-10	0,255318	0,931	0,836	8,95E-06	8	ADAMTS1
ADIRF1	7,86E-08	0,287879	0,949	0,921	0,001504	8	ADIRF
LRRC75A2	0,000116	0,289413	0,731	0,606	1	8	LRRC75A
CTNNB12	0,000364	0,270953	0,833	0,768	1	8	CTNNB1
TROAP	1,49E-138	0,327434	0,596	0,058	2,85E-134	9	TROAP

TK1	7,54E-132	1,005025	0,967	0,264	1,44E-127	9	TK1
BIRC5	1,38E-123	0,597668	0,727	0,11	2,65E-119	9	BIRC5
KIAA01011	2,39E-97	1,220432	0,973	0,387	4,57E-93	9	KIAA0101
RRM2	1,41E-95	0,277146	0,536	0,069	2,69E-91	9	RRM2
HMGA12	4,99E-86	1,637804	1	0,967	9,55E-82	9	HMGA1
TUBA1B1	8,24E-83	1,89006	1	0,995	1,58E-78	9	TUBA1B
PPP1R14B1	4,92E-81	1,130311	1	0,963	9,40E-77	9	PPP1R14B
LDHA2	4,24E-80	1,135739	1	0,993	8,10E-76	9	LDHA
MT1E2	3,76E-76	1,58775	1	0,961	7,20E-72	9	MT1E
PTTG1	6,82E-75	1,308637	0,891	0,39	1,30E-70	9	PTTG1
TPI12	1,44E-74	0,915493	1	0,995	2,76E-70	9	TPI1
CDKN3	3,61E-74	0,80548	0,847	0,312	6,91E-70	9	CDKN3
HMGN2	1,15E-73	1,13819	1	0,993	2,20E-69	9	HMGN2
DRAP1	7,17E-73	0,788801	1	0,997	1,37E-68	9	DRAP1
MT2A1	1,21E-71	1,940431	1	0,999	2,32E-67	9	MT2A
RPL22L12	1,38E-70	1,146365	1	0,972	2,63E-66	9	RPL22L1
NME12	6,98E-70	0,914858	1	0,936	1,33E-65	9	NME1
HNRNPA11	4,98E-69	0,746963	1	0,999	9,53E-65	9	HNRNPA1
CENPM	6,18E-66	0,319512	0,612	0,145	1,18E-61	9	CENPM
ENO12	2,49E-65	1,141313	1	0,998	4,75E-61	9	ENO1
YBX1	3,61E-65	0,465286	1	1	6,90E-61	9	YBX1
ATP5G11	6,97E-65	0,71624	0,995	0,969	1,33E-60	9	ATP5G1
MYDGF2	1,60E-63	0,706112	1	0,997	3,06E-59	9	MYDGF
H2AFV	2,04E-63	0,731799	0,995	0,931	3,90E-59	9	H2AFV
NHP21	2,77E-63	0,663419	1	0,965	5,31E-59	9	NHP2
HMGB1	4,00E-63	1,056837	1	0,997	7,65E-59	9	HMGB1
PLP2	3,79E-60	0,738059	0,995	0,934	7,24E-56	9	PLP2
DKK12	5,91E-60	1,367778	0,951	0,677	1,13E-55	9	DKK1
PGAM11	9,00E-60	0,638387	1	0,994	1,72E-55	9	PGAM1
RPA3	3,87E-59	0,539519	0,962	0,615	7,41E-55	9	RPA3
NUDT1	1,01E-58	0,517995	0,951	0,619	1,93E-54	9	NUDT1
PKM2	6,05E-58	0,906751	1	0,999	1,16E-53	9	PKM
C1QBP	6,64E-58	0,756889	0,989	0,961	1,27E-53	9	C1QBP
HNRNPA2B	1,61E-57	0,794091	1	0,993	3,09E-53	9	HNRNPA2B1
HN1	2,41E-57	0,829079	1	0,954	4,62E-53	9	HN1
CENPW	1,06E-56	0,690023	0,803	0,348	2,03E-52	9	CENPW
CFL11	1,17E-56	0,588357	1	1	2,25E-52	9	CFL1
RAN1	2,28E-56	0,766682	1	0,996	4,36E-52	9	RAN
HNRNPC	3,29E-56	0,608588	1	0,984	6,28E-52	9	HNRNPC
TYMS	3,67E-56	0,501772	0,792	0,298	7,01E-52	9	TYMS
RANBP1	4,72E-56	0,730043	1	0,943	9,02E-52	9	RANBP1
COX8A	5,74E-56	0,499593	1	0,997	1,10E-51	9	COX8A
HSPD11	1,44E-55	0,741077	0,984	0,922	2,76E-51	9	HSPD1
LMNA2	1,77E-55	0,746661	1	0,998	3,39E-51	9	LMNA
SH3BGRL31	1,94E-55	0,704691	1	0,997	3,71E-51	9	SH3BGRL3
SRM1	1,29E-54	0,685667	1	0,966	2,46E-50	9	SRM
TNFRSF12A	1,79E-54	0,807275	1	0,955	3,42E-50	9	TNFRSF12A
HERC4	2,89E-54	0,763595	0,978	0,8	5,52E-50	9	HERC4
GGH	7,93E-54	0,426881	0,918	0,44	1,52E-49	9	GGH
ANXA22	1,06E-53	0,835588	1	1	2,02E-49	9	ANXA2

CDC20	1,20E-52	0,32257	0,377	0,063	2,30E-48	9	CDC20
IL7R	3,48E-52	0,283588	0,661	0,201	6,65E-48	9	IL7R
PRELID11	1,54E-51	0,558391	1	0,997	2,95E-47	9	PRELID1
PFN11	2,20E-51	0,49332	1	1	4,21E-47	9	PFN1
H2AFZ	2,97E-50	1,063013	1	0,997	5,69E-46	9	H2AFZ
EBNA1BP2	9,16E-50	0,707333	0,984	0,817	1,75E-45	9	EBNA1BP2
SLC25A5	2,87E-49	0,606956	0,995	0,988	5,48E-45	9	SLC25A5
AURKAIP1	5,71E-49	0,470972	1	0,993	1,09E-44	9	AURKAIP1
SNRPB	1,49E-48	0,717239	1	0,98	2,84E-44	9	SNRPB
SERBP1	2,99E-48	0,543431	1	0,991	5,71E-44	9	SERBP1
ATP5G32	3,42E-48	0,525152	1	0,997	6,54E-44	9	ATP5G3
LSM4	6,19E-48	0,556567	0,995	0,963	1,18E-43	9	LSM4
HMGN1	6,71E-48	0,481864	1	0,999	1,28E-43	9	HMGN1
NDUFS6	8,18E-48	0,481168	1	0,997	1,56E-43	9	NDUFS6
CENPN	1,78E-47	0,351563	0,787	0,355	3,40E-43	9	CENPN
CLIC12	2,69E-47	0,568845	1	0,999	5,14E-43	9	CLIC1
UACA	1,56E-46	0,677763	0,984	0,865	2,98E-42	9	UACA
NT5E	1,87E-46	0,564771	0,896	0,515	3,58E-42	9	NT5E
EIF5A1	4,23E-46	0,571534	0,989	0,893	8,09E-42	9	EIF5A
STMN1	1,12E-45	0,886927	0,984	0,794	2,14E-41	9	STMN1
GTF3A	1,48E-45	0,500696	0,989	0,957	2,82E-41	9	GTF3A
CHCHD2	2,82E-45	0,349682	1	0,999	5,40E-41	9	CHCHD2
SNRPE	4,20E-45	0,476218	0,995	0,993	8,04E-41	9	SNRPE
SRSF31	8,38E-45	0,671143	1	0,976	1,60E-40	9	SRSF3
NPM11	1,95E-44	0,580171	1	1	3,74E-40	9	NPM1
RPS26	3,18E-44	0,364192	1	1	6,07E-40	9	RPS26
MRPL51	7,67E-44	0,442675	1	0,993	1,47E-39	9	MRPL51
HNRNPH3	1,91E-43	0,550772	0,989	0,835	3,65E-39	9	HNRNPH3
GAPDH1	2,31E-43	0,422518	1	1	4,42E-39	9	GAPDH
HNRNPM1	4,18E-43	0,542357	0,973	0,874	8,00E-39	9	HNRNPM
PDIA62	4,72E-43	0,604148	1	0,974	9,03E-39	9	PDIA6
ARPC1A	5,10E-43	0,445149	1	0,993	9,76E-39	9	ARPC1A
TMEM14B1	8,10E-43	0,521787	0,995	0,972	1,55E-38	9	TMEM14B
MRPL52	1,89E-42	0,459391	1	0,97	3,62E-38	9	MRPL52
GTF3C6	1,35E-41	0,455662	0,995	0,94	2,59E-37	9	GTF3C6
PRKCDBP1	1,76E-41	0,528839	0,995	0,995	3,36E-37	9	PRKCDBP
STOML2	1,90E-41	0,482884	0,995	0,969	3,63E-37	9	STOML2
GADD45GII	2,66E-41	0,416467	1	0,993	5,10E-37	9	GADD45GIP1
TUBA1C1	4,29E-41	0,70942	1	0,957	8,19E-37	9	TUBA1C
SLIRP	4,64E-41	0,468582	1	0,987	8,88E-37	9	SLIRP
AP2S1	5,47E-41	0,411154	1	1	1,05E-36	9	AP2S1
PHB	7,79E-41	0,463081	1	0,965	1,49E-36	9	PHB
ALYREF	7,84E-41	0,375524	0,852	0,49	1,50E-36	9	ALYREF
LGALS1	1,66E-40	0,344636	1	1	3,18E-36	9	LGALS1
EIF3I1	1,71E-40	0,488215	1	0,991	3,28E-36	9	EIF3I
PDCD51	2,14E-40	0,481701	1	0,989	4,10E-36	9	PDCD5
DTYMK	3,34E-40	0,540839	0,929	0,65	6,39E-36	9	DTYMK
CYC11	7,02E-40	0,475633	1	0,96	1,34E-35	9	CYC1
PSMC51	8,16E-40	0,461944	1	0,939	1,56E-35	9	PSMC5
COX201	1,45E-39	0,51858	0,989	0,941	2,78E-35	9	COX20

PA2G41	3,36E-39	0,534117	0,989	0,925	6,43E-35	9	PA2G4
ODC11	7,74E-39	0,615161	0,951	0,76	1,48E-34	9	ODC1
CAV11	8,19E-39	0,650914	1	0,999	1,57E-34	9	CAV1
PLIN31	1,12E-38	0,465311	0,995	0,925	2,13E-34	9	PLIN3
FABP5	1,48E-38	0,343228	0,787	0,4	2,82E-34	9	FABP5
SMS	1,95E-38	0,551636	0,989	0,94	3,73E-34	9	SMS
EMC4	2,20E-38	0,412227	1	0,93	4,21E-34	9	EMC4
BCL7C	5,35E-38	0,46209	0,984	0,915	1,02E-33	9	BCL7C
PRMT11	1,12E-37	0,552559	0,995	0,882	2,14E-33	9	PRMT1
FKBP2	1,44E-37	0,424756	1	0,986	2,76E-33	9	FKBP2
HSP90B11	1,88E-37	0,577773	1	0,965	3,60E-33	9	HSP90B1
PSMB71	2,18E-37	0,433156	0,989	0,994	4,17E-33	9	PSMB7
PSMB62	3,02E-37	0,506526	1	0,982	5,78E-33	9	PSMB6
SRSF9	3,35E-37	0,430441	1	0,978	6,40E-33	9	SRSF9
SRP9	3,71E-37	0,388192	1	0,976	7,09E-33	9	SRP9
UBE2L31	4,10E-37	0,449271	1	0,988	7,83E-33	9	UBE2L3
CALR2	1,72E-36	0,557479	1	0,998	3,29E-32	9	CALR
PSMD21	1,94E-36	0,472021	0,978	0,888	3,71E-32	9	PSMD2
MT1X	2,13E-36	0,61091	0,956	0,781	4,08E-32	9	MT1X
EMP31	2,41E-36	0,501691	1	0,999	4,61E-32	9	EMP3
CD441	4,57E-36	0,504052	1	0,999	8,74E-32	9	CD44
SNU131	1,00E-35	0,427234	1	0,994	1,91E-31	9	SNU13
PSMA72	2,18E-35	0,523951	1	0,999	4,17E-31	9	PSMA7
ARPC1B1	2,96E-35	0,508909	1	0,995	5,66E-31	9	ARPC1B
STRA13	3,62E-35	0,466037	0,978	0,82	6,92E-31	9	STRA13
ARPC21	5,08E-35	0,467267	1	0,999	9,71E-31	9	ARPC2
TXNDC17	1,75E-34	0,444336	0,995	0,967	3,35E-30	9	TXNDC17
BOLA31	1,88E-34	0,4502	0,973	0,867	3,59E-30	9	BOLA3
PSMB31	2,15E-34	0,492545	0,995	0,976	4,12E-30	9	PSMB3
RPL81	2,25E-34	0,26696	1	1	4,30E-30	9	RPL8
S100A112	3,99E-34	0,399558	1	1	7,63E-30	9	S100A11
PSMB21	5,47E-34	0,412059	1	0,973	1,05E-29	9	PSMB2
PARK71	7,24E-34	0,384534	1	0,999	1,38E-29	9	PARK7
COTL11	8,30E-34	0,477737	1	0,978	1,59E-29	9	COTL1
MRPL12	9,36E-34	0,44185	0,989	0,921	1,79E-29	9	MRPL12
TOMM22	1,15E-33	0,40566	1	0,971	2,21E-29	9	TOMM22
HSPE1	1,33E-33	0,494346	0,995	0,988	2,54E-29	9	HSPE1
FST1	1,71E-33	0,853181	0,907	0,65	3,27E-29	9	FST
ANXA13	1,91E-33	0,520535	1	0,992	3,66E-29	9	ANXA1
MRPL36	2,61E-33	0,445768	0,995	0,948	4,99E-29	9	MRPL36
FXYD5	3,71E-33	0,411467	0,995	0,982	7,09E-29	9	FXYD5
SFRP11	1,11E-32	0,725193	0,88	0,653	2,12E-28	9	SFRP1
BANF1	1,11E-32	0,410659	1	0,964	2,12E-28	9	BANF1
HSP90AB1:	1,58E-32	0,542532	1	0,999	3,01E-28	9	HSP90AB1
ADRM11	2,16E-32	0,426953	1	0,962	4,13E-28	9	ADRM1
ARF1	2,80E-32	0,44146	1	0,99	5,34E-28	9	ARF1
CD59	2,96E-32	0,412081	1	0,997	5,65E-28	9	CD59
PPIA	3,18E-32	0,307329	1	1	6,07E-28	9	PPIA
PDAP1	3,99E-32	0,382744	0,995	0,97	7,62E-28	9	PDAP1
TMED92	6,54E-32	0,4394	1	0,987	1,25E-27	9	TMED9

DIO2	8,26E-32	0,38833	0,552	0,202	1,58E-27	9	DIO2
DDX39A	8,42E-32	0,340424	0,792	0,407	1,61E-27	9	DDX39A
TMEM167/	8,86E-32	0,348645	0,995	0,959	1,69E-27	9	TMEM167A
NDUFC21	1,00E-31	0,417057	1	0,984	1,91E-27	9	NDUFC2
SSRP1	1,08E-31	0,381178	0,945	0,767	2,06E-27	9	SSRP1
CTNNAL11	1,24E-31	0,471941	0,94	0,742	2,38E-27	9	CTNNAL1
NOL71	1,81E-31	0,395066	0,989	0,902	3,47E-27	9	NOL7
HNRNPDL	1,86E-31	0,502192	0,989	0,963	3,55E-27	9	HNRNPDL
ATPIF11	5,22E-31	0,398315	1	0,99	9,99E-27	9	ATPIF1
PTMS	5,75E-31	0,384198	1	0,998	1,10E-26	9	PTMS
DTD1	6,82E-31	0,386794	0,956	0,795	1,30E-26	9	DTD1
CCT51	6,96E-31	0,474638	0,989	0,872	1,33E-26	9	CCT5
C12orf751	7,82E-31	0,662614	1	0,996	1,50E-26	9	C12orf75
CALM3	8,43E-31	0,403654	0,989	0,971	1,61E-26	9	CALM3
TPM3	8,76E-31	0,403059	1	0,981	1,68E-26	9	TPM3
SNRPA	9,80E-31	0,404574	0,945	0,698	1,87E-26	9	SNRPA
DHFR	1,12E-30	0,25656	0,727	0,372	2,14E-26	9	DHFR
ATP5J2	1,36E-30	0,326523	1	0,996	2,61E-26	9	ATP5J2
RUVBL2	1,40E-30	0,349089	0,929	0,658	2,67E-26	9	RUVBL2
TUBB1	1,69E-30	0,525972	1	0,999	3,23E-26	9	TUBB
HNRNPD1	2,15E-30	0,423687	0,984	0,922	4,11E-26	9	HNRNPD
MRPL3	2,42E-30	0,381268	0,978	0,845	4,62E-26	9	MRPL3
RGS20	2,63E-30	0,26554	0,699	0,351	5,03E-26	9	RGS20
CUTA	2,81E-30	0,365443	1	0,999	5,38E-26	9	CUTA
LINC00475	3,06E-30	0,498396	0,858	0,6	5,85E-26	9	LINC00475
NDUFV21	3,26E-30	0,367687	0,995	0,988	6,23E-26	9	NDUFV2
SPCS11	3,52E-30	0,368231	1	0,99	6,74E-26	9	SPCS1
SERPINE1	4,68E-30	0,350884	0,699	0,339	8,94E-26	9	SERPINE1
PAICS	5,79E-30	0,378086	0,869	0,627	1,11E-25	9	PAICS
CCDC85B	7,74E-30	0,489658	0,989	0,995	1,48E-25	9	CCDC85B
EIF4A11	9,42E-30	0,449415	1	0,973	1,80E-25	9	EIF4A1
CYCS1	9,50E-30	0,484691	1	0,965	1,82E-25	9	CYCS
TUBB4B1	1,11E-29	0,687268	1	0,914	2,13E-25	9	TUBB4B
SNRPG	1,20E-29	0,445679	1	0,987	2,30E-25	9	SNRPG
PFDN6	1,23E-29	0,332867	0,945	0,745	2,36E-25	9	PFDN6
H3F3B	1,40E-29	0,392134	1	0,997	2,67E-25	9	H3F3B
SNRPC	1,56E-29	0,415665	0,995	0,934	2,99E-25	9	SNRPC
LSM3	1,59E-29	0,393225	1	0,979	3,05E-25	9	LSM3
SNRPA1	1,98E-29	0,370773	0,852	0,578	3,78E-25	9	SNRPA1
EIF61	2,48E-29	0,410789	0,995	0,963	4,74E-25	9	EIF6
RAB34	2,52E-29	0,359974	0,995	0,983	4,83E-25	9	RAB34
WBSCR221	2,55E-29	0,366562	0,967	0,81	4,87E-25	9	WBSCR22
SLC16A32	2,55E-29	0,51008	0,989	0,907	4,88E-25	9	SLC16A3
CLTB1	4,69E-29	0,381135	1	0,981	8,98E-25	9	CLTB
MRPL141	5,28E-29	0,402953	1	0,975	1,01E-24	9	MRPL14
CCT6A1	6,41E-29	0,394712	1	0,918	1,23E-24	9	CCT6A
PSMD82	8,01E-29	0,43309	1	0,994	1,53E-24	9	PSMD8
MZT2B	8,25E-29	0,324128	1	0,995	1,58E-24	9	MZT2B
UBE2M	8,83E-29	0,371126	0,945	0,801	1,69E-24	9	UBE2M
PSMB12	1,05E-28	0,462831	1	0,995	2,00E-24	9	PSMB1

C20orf27	1,30E-28	0,375698	0,967	0,789	2,48E-24	9	C20orf27
MBOAT7	1,47E-28	0,374473	0,945	0,776	2,82E-24	9	MBOAT7
DUT	2,40E-28	0,419611	0,984	0,923	4,58E-24	9	DUT
MRPS7	5,92E-28	0,355039	0,978	0,868	1,13E-23	9	MRPS7
NCL1	6,25E-28	0,596564	1	0,988	1,19E-23	9	NCL
CCDC71L	6,48E-28	0,508594	0,967	0,924	1,24E-23	9	CCDC71L
SDF2L1	9,55E-28	0,420303	0,956	0,833	1,83E-23	9	SDF2L1
CNPY2	9,66E-28	0,330586	0,995	0,956	1,85E-23	9	CNPY2
LRRC59	9,96E-28	0,404524	0,989	0,926	1,90E-23	9	LRRC59
SNRPD1	1,14E-27	0,427264	0,989	0,968	2,18E-23	9	SNRPD1
POLR2E	1,46E-27	0,354431	1	0,972	2,79E-23	9	POLR2E
TIMM17A	1,50E-27	0,387575	0,984	0,912	2,87E-23	9	TIMM17A
SET	1,59E-27	0,377515	0,995	0,996	3,05E-23	9	SET
NDUFB3	1,76E-27	0,326925	0,984	0,976	3,37E-23	9	NDUFB3
ERH	2,17E-27	0,382824	1	0,994	4,16E-23	9	ERH
OGFRL1	3,10E-27	0,363404	0,896	0,655	5,93E-23	9	OGFRL1
COX7A21	4,72E-27	0,271083	1	0,999	9,03E-23	9	COX7A2
PSME21	5,47E-27	0,424616	0,995	0,95	1,05E-22	9	PSME2
HNRNPA31	7,23E-27	0,410309	0,995	0,969	1,38E-22	9	HNRNPA3
MRPL281	7,60E-27	0,349484	0,984	0,907	1,45E-22	9	MRPL28
CALM11	7,84E-27	0,378733	1	0,991	1,50E-22	9	CALM1
NDUFAB1	1,03E-26	0,362802	0,989	0,932	1,96E-22	9	NDUFAB1
SRSF21	1,32E-26	0,426578	1	0,952	2,53E-22	9	SRSF2
RPL7L1	1,58E-26	0,31532	0,984	0,877	3,03E-22	9	RPL7L1
HMGB3	1,92E-26	0,293749	0,803	0,483	3,67E-22	9	HMGB3
NDUFA121	2,17E-26	0,368009	0,989	0,924	4,15E-22	9	NDUFA12
UBE2S	3,34E-26	0,754345	0,934	0,732	6,39E-22	9	UBE2S
SRGN1	6,06E-26	0,550455	0,896	0,628	1,16E-21	9	SRGN
CD320	7,52E-26	0,319613	0,885	0,656	1,44E-21	9	CD320
SURF4	1,02E-25	0,353338	0,995	0,926	1,95E-21	9	SURF4
SNF8	1,07E-25	0,324292	0,995	0,98	2,04E-21	9	SNF8
HDGF	1,50E-25	0,338799	0,984	0,858	2,86E-21	9	HDGF
MLF21	1,56E-25	0,37614	1	0,982	2,98E-21	9	MLF2
COPS62	1,64E-25	0,386839	1	0,973	3,13E-21	9	COPS6
ECHS1	2,36E-25	0,351484	0,989	0,937	4,51E-21	9	ECHS1
RHOC	3,44E-25	0,317634	0,995	0,996	6,58E-21	9	RHOC
POLD2	4,75E-25	0,325756	0,973	0,876	9,09E-21	9	POLD2
PDIA41	5,70E-25	0,328335	0,967	0,786	1,09E-20	9	PDIA4
ARHGAP18	1,01E-24	0,28544	0,825	0,521	1,94E-20	9	ARHGAP18
SLIT2	1,58E-24	0,275062	0,705	0,383	3,02E-20	9	SLIT2
MRPS12	1,77E-24	0,331945	1	0,935	3,38E-20	9	MRPS12
EMG1	1,87E-24	0,314096	0,836	0,577	3,58E-20	9	EMG1
ALDOA2	2,08E-24	0,332559	1	1	3,97E-20	9	ALDOA
PHF19	2,16E-24	0,422053	0,77	0,518	4,13E-20	9	PHF19
TXNL4A	2,80E-24	0,315157	0,984	0,948	5,36E-20	9	TXNL4A
PSMC31	3,08E-24	0,39024	0,995	0,921	5,88E-20	9	PSMC3
ATP5B1	3,41E-24	0,371351	1	0,994	6,52E-20	9	ATP5B
TECR2	4,69E-24	0,334431	0,967	0,867	8,96E-20	9	TECR
MRPL24	6,57E-24	0,303933	0,913	0,697	1,26E-19	9	MRPL24
BAX	6,84E-24	0,33523	1	0,987	1,31E-19	9	BAX

SF3B6	9,84E-24	0,334336	1	0,99	1,88E-19	9	SF3B6
YWHAE1	1,01E-23	0,322072	1	0,995	1,93E-19	9	YWHAE
RUVBL1	1,03E-23	0,290534	0,814	0,496	1,97E-19	9	RUVBL1
AP1S1	1,32E-23	0,307645	0,989	0,953	2,53E-19	9	AP1S1
HPRT1	1,33E-23	0,250621	0,852	0,558	2,55E-19	9	HPRT1
EWSR1	1,52E-23	0,311132	0,967	0,829	2,91E-19	9	EWSR1
UBE2I	1,67E-23	0,32488	1	0,982	3,20E-19	9	UBE2I
NTMT1	1,81E-23	0,374997	0,989	0,93	3,47E-19	9	NTMT1
GSPT11	1,92E-23	0,31834	0,967	0,858	3,67E-19	9	GSPT1
SRSF7	1,94E-23	0,457316	0,973	0,847	3,71E-19	9	SRSF7
UQCR101	2,27E-23	0,318449	0,995	0,995	4,34E-19	9	UQCR10
C17orf891	2,74E-23	0,394135	1	0,986	5,24E-19	9	C17orf89
MRPL57	3,23E-23	0,317598	0,995	0,976	6,18E-19	9	MRPL57
COA4	3,54E-23	0,302891	0,989	0,895	6,77E-19	9	COA4
NDUFAF3	5,34E-23	0,320478	0,995	0,98	1,02E-18	9	NDUFAF3
VDAC11	6,87E-23	0,308099	1	0,987	1,31E-18	9	VDAC1
GSTO11	7,16E-23	0,394364	1	0,994	1,37E-18	9	GSTO1
CAPN21	9,34E-23	0,333883	0,995	0,984	1,79E-18	9	CAPN2
NUTF2	9,95E-23	0,304561	0,989	0,977	1,90E-18	9	NUTF2
MANF1	1,01E-22	0,410198	0,978	0,874	1,93E-18	9	MANF
FKBP1A1	1,03E-22	0,294854	1	0,996	1,97E-18	9	FKBP1A
MRPL20	1,16E-22	0,320829	0,989	0,974	2,21E-18	9	MRPL20
SNRPF	1,49E-22	0,322687	1	0,981	2,85E-18	9	SNRPF
AXL2	1,55E-22	0,621255	0,984	0,925	2,97E-18	9	AXL
DAZAP1	1,65E-22	0,26885	0,913	0,711	3,15E-18	9	DAZAP1
ANP32E	1,72E-22	0,302095	0,88	0,592	3,29E-18	9	ANP32E
SEC61G1	1,79E-22	0,269443	1	0,998	3,43E-18	9	SEC61G
PLAUR	1,82E-22	0,335273	0,902	0,687	3,49E-18	9	PLAUR
CCT71	2,01E-22	0,323084	0,989	0,916	3,84E-18	9	CCT7
SSBP11	2,52E-22	0,327152	0,995	0,984	4,83E-18	9	SSBP1
PPP1CA	2,64E-22	0,335493	1	0,967	5,06E-18	9	PPP1CA
GPATCH4	2,88E-22	0,322267	0,858	0,635	5,50E-18	9	GPATCH4
HYI	2,95E-22	0,307179	0,896	0,65	5,63E-18	9	HYI
SIGMAR1	2,95E-22	0,283122	0,929	0,696	5,64E-18	9	SIGMAR1
TOMM40	3,06E-22	0,37519	0,869	0,678	5,86E-18	9	TOMM40
RPS19BP1	3,25E-22	0,31801	1	0,973	6,21E-18	9	RPS19BP1
S100A102	3,26E-22	0,36578	1	0,997	6,24E-18	9	S100A10
NANS2	4,06E-22	0,350505	0,984	0,842	7,76E-18	9	NANS
LARP6	4,29E-22	0,356006	1	0,986	8,20E-18	9	LARP6
BASP11	4,76E-22	0,341095	1	0,989	9,11E-18	9	BASP1
RER11	5,15E-22	0,303345	1	0,98	9,84E-18	9	RER1
TRMT112	5,83E-22	0,299731	1	0,993	1,11E-17	9	TRMT112
MRPS18C	6,25E-22	0,32396	0,995	0,878	1,19E-17	9	MRPS18C
MRPL43	7,64E-22	0,276082	1	0,931	1,46E-17	9	MRPL43
GLRX2	8,04E-22	0,576725	1	0,993	1,54E-17	9	GLRX
TAGLN2	1,21E-21	0,341928	0,995	0,988	2,31E-17	9	TAGLN2
HSPA52	1,22E-21	0,507534	1	0,975	2,32E-17	9	HSPA5
LSM7	1,30E-21	0,321069	1	0,984	2,49E-17	9	LSM7
IMPDH2	1,63E-21	0,379424	0,984	0,925	3,12E-17	9	IMPDH2
MRPL4	1,70E-21	0,294946	0,984	0,876	3,25E-17	9	MRPL4

PTMA2	1,85E-21	0,386783	1	1	3,54E-17	9	PTMA
FUS	1,96E-21	0,354883	0,989	0,928	3,74E-17	9	FUS
UFD1L1	2,03E-21	0,284694	0,978	0,85	3,89E-17	9	UFD1L
NMT2	2,59E-21	0,288438	0,945	0,748	4,95E-17	9	NMT2
PTGES3	3,41E-21	0,344248	1	0,984	6,52E-17	9	PTGES3
GNAI2	4,83E-21	0,292435	0,989	0,968	9,24E-17	9	GNAI2
YWHAQ1	5,03E-21	0,308537	1	0,992	9,61E-17	9	YWHAQ
SSR3	5,17E-21	0,328344	1	0,992	9,88E-17	9	SSR3
LACTB1	8,00E-21	0,338554	0,945	0,831	1,53E-16	9	LACTB
PSMD131	8,46E-21	0,291419	0,951	0,784	1,62E-16	9	PSMD13
CCT21	8,92E-21	0,349675	0,973	0,9	1,71E-16	9	CCT2
PRSS3	9,12E-21	0,267398	0,552	0,269	1,74E-16	9	PRSS3
NPM3	9,96E-21	0,306668	0,984	0,885	1,90E-16	9	NPM3
HNRNPK1	1,17E-20	0,330829	1	0,995	2,24E-16	9	HNRNPK
XRCC61	1,38E-20	0,346947	0,995	0,93	2,64E-16	9	XRCC6
MINOS1	1,42E-20	0,279919	1	0,99	2,71E-16	9	MINOS1
KRT183	1,48E-20	0,924873	0,896	0,772	2,83E-16	9	KRT18
RBX1	1,64E-20	0,268054	1	0,991	3,13E-16	9	RBX1
THRAP31	2,01E-20	0,311947	0,995	0,911	3,84E-16	9	THRAP3
ILF21	2,19E-20	0,389789	0,989	0,854	4,18E-16	9	ILF2
NT5DC2	2,46E-20	0,271959	0,852	0,624	4,70E-16	9	NT5DC2
HNRNPR	2,58E-20	0,35244	0,989	0,895	4,94E-16	9	HNRNPR
FBL	2,69E-20	0,28047	0,978	0,927	5,14E-16	9	FBL
NDUFB9	3,01E-20	0,271611	1	0,99	5,76E-16	9	NDUFB9
SFPQ	3,19E-20	0,313194	1	0,939	6,10E-16	9	SFPQ
PDCD6	3,20E-20	0,285062	0,989	0,963	6,12E-16	9	PDCD6
CCDC124	3,23E-20	0,305928	0,978	0,898	6,18E-16	9	CCDC124
XRCC51	3,51E-20	0,352087	0,989	0,935	6,72E-16	9	XRCC5
SLC35B1	3,89E-20	0,273267	0,94	0,784	7,43E-16	9	SLC35B1
CACYBP1	4,00E-20	0,323105	0,962	0,805	7,66E-16	9	CACYBP
SKA2	4,20E-20	0,294724	0,984	0,836	8,03E-16	9	SKA2
ARHGDIA	4,36E-20	0,303907	0,995	0,975	8,34E-16	9	ARHGDIA
MRPL17	4,98E-20	0,333215	0,973	0,871	9,53E-16	9	MRPL17
UBB1	5,51E-20	0,403706	1	0,999	1,05E-15	9	UBB
NUDC1	5,81E-20	0,332172	0,978	0,921	1,11E-15	9	NUDC
STRAP	5,86E-20	0,311705	1	0,98	1,12E-15	9	STRAP
EIF1AX	6,99E-20	0,316125	1	0,966	1,34E-15	9	EIF1AX
GNL3	7,03E-20	0,307871	0,902	0,68	1,34E-15	9	GNL3
PSMB51	8,16E-20	0,274725	0,995	0,994	1,56E-15	9	PSMB5
NDUFC1	8,37E-20	0,295331	1	0,964	1,60E-15	9	NDUFC1
CISD3	1,04E-19	0,278949	0,978	0,841	1,99E-15	9	CISD3
MRPS34	1,40E-19	0,298783	0,995	0,952	2,67E-15	9	MRPS34
MRPL23	1,50E-19	0,272256	0,989	0,961	2,88E-15	9	MRPL23
CRELD2	1,60E-19	0,263956	0,945	0,791	3,07E-15	9	CRELD2
LSM5	1,67E-19	0,393738	1	0,94	3,20E-15	9	LSM5
MLEC	1,71E-19	0,272856	0,989	0,927	3,27E-15	9	MLEC
PHLDA21	1,73E-19	0,487965	0,995	0,965	3,30E-15	9	PHLDA2
SUMO3	1,82E-19	0,262691	0,989	0,952	3,48E-15	9	SUMO3
ENY2	1,92E-19	0,292051	1	0,984	3,66E-15	9	ENY2
MAP2K3	2,26E-19	0,27587	0,852	0,631	4,31E-15	9	MAP2K3

ADIRF2	2,50E-19	0,674714	0,978	0,919	4,79E-15	9	ADIRF
ASPH2	2,72E-19	0,30523	0,995	0,986	5,20E-15	9	ASPH
POMP2	3,46E-19	0,283623	1	1	6,61E-15	9	POMP
TMEM158	3,55E-19	0,501808	0,792	0,58	6,78E-15	9	TMEM158
CLEC11A	4,33E-19	0,28705	0,956	0,794	8,28E-15	9	CLEC11A
RPL26L1	4,74E-19	0,264124	0,984	0,941	9,06E-15	9	RPL26L1
ANAPC15	5,22E-19	0,283864	0,984	0,887	9,97E-15	9	ANAPC15
ITGA2	5,33E-19	0,388035	0,705	0,468	1,02E-14	9	ITGA2
BRMS1	5,44E-19	0,277716	0,94	0,768	1,04E-14	9	BRMS1
TSR3	5,49E-19	0,266909	0,951	0,874	1,05E-14	9	TSR3
SEC131	5,84E-19	0,297465	0,984	0,923	1,12E-14	9	SEC13
VPS25	6,03E-19	0,2552	0,967	0,824	1,15E-14	9	VPS25
CKAP41	6,56E-19	0,287642	1	0,986	1,25E-14	9	CKAP4
UBE2N	6,77E-19	0,302494	0,978	0,923	1,29E-14	9	UBE2N
TMEM106(	8,19E-19	0,299528	0,962	0,791	1,57E-14	9	TMEM106C
DDX46	8,61E-19	0,274487	0,984	0,944	1,65E-14	9	DDX46
NDUFB6	8,71E-19	0,28364	0,973	0,926	1,67E-14	9	NDUFB6
EEF1E1	9,28E-19	0,264328	0,842	0,627	1,78E-14	9	EEF1E1
RAC11	9,50E-19	0,288597	1	0,999	1,82E-14	9	RAC1
NDUFA81	1,18E-18	0,273427	0,995	0,931	2,26E-14	9	NDUFA8
COMMD4	1,39E-18	0,278764	0,973	0,912	2,66E-14	9	COMMD4
SRA1	1,47E-18	0,277527	0,978	0,946	2,82E-14	9	SRA1
CCT41	1,64E-18	0,273113	0,989	0,944	3,13E-14	9	CCT4
MEST1	1,97E-18	0,512153	0,94	0,825	3,76E-14	9	MEST
ISOC2	2,14E-18	0,274104	0,989	0,92	4,09E-14	9	ISOC2
EID11	2,70E-18	0,273527	1	0,999	5,15E-14	9	EID1
ZDHHC12	2,72E-18	0,275241	0,973	0,938	5,20E-14	9	ZDHHC12
ILF3	3,01E-18	0,265293	0,923	0,797	5,75E-14	9	ILF3
MT-ATP6	3,91E-18	0,258808	1	1	7,47E-14	9	MT-ATP6
NDUFB10	4,30E-18	0,26529	0,995	0,994	8,23E-14	9	NDUFB10
MRPS16	4,49E-18	0,264009	0,995	0,955	8,59E-14	9	MRPS16
HGF	4,78E-18	0,451216	0,907	0,8	9,15E-14	9	HGF
YWHAB1	6,65E-18	0,26546	1	0,994	1,27E-13	9	YWHAB
STUB1	7,39E-18	0,269761	0,989	0,984	1,41E-13	9	STUB1
TM4SF1	8,11E-18	0,397855	0,678	0,398	1,55E-13	9	TM4SF1
AP2M12	1,20E-17	0,266334	1	0,998	2,30E-13	9	AP2M1
NDUFS8	1,29E-17	0,287431	1	0,987	2,46E-13	9	NDUFS8
UBE2J2	1,29E-17	0,254996	0,951	0,827	2,47E-13	9	UBE2J2
PSMA31	1,99E-17	0,319683	0,995	0,961	3,81E-13	9	PSMA3
EBPL1	2,12E-17	0,276755	0,973	0,932	4,05E-13	9	EBPL
MRPL131	2,61E-17	0,265524	0,978	0,878	4,98E-13	9	MRPL13
MPG	2,81E-17	0,279946	1	0,992	5,37E-13	9	MPG
SNRNP701	3,40E-17	0,265367	0,962	0,878	6,49E-13	9	SNRNP70
MRPL21	4,68E-17	0,268489	0,978	0,891	8,96E-13	9	MRPL21
PSMC41	4,98E-17	0,270706	0,918	0,764	9,52E-13	9	PSMC4
C16orf13	5,08E-17	0,250364	0,989	0,954	9,71E-13	9	C16orf13
DBI	5,26E-17	0,284567	1	0,999	1,01E-12	9	DBI
CISD1	5,30E-17	0,280354	0,978	0,971	1,01E-12	9	CISD1
TCP11	5,73E-17	0,264484	1	0,932	1,10E-12	9	TCP1
PGK11	6,64E-17	0,299061	0,995	0,974	1,27E-12	9	PGK1

KDELR21	6,79E-17	0,26336	1	0,995	1,30E-12	9	KDELR2
NFIC	8,61E-17	0,264837	1	0,974	1,65E-12	9	NFIC
CTDNEP1	8,87E-17	0,254442	0,978	0,887	1,70E-12	9	CTDNEP1
PIN1	8,88E-17	0,259042	0,995	0,963	1,70E-12	9	PIN1
PPP4C	1,01E-16	0,276574	0,989	0,967	1,93E-12	9	PPP4C
NOP16	1,08E-16	0,293914	0,71	0,471	2,06E-12	9	NOP16
PRDX61	1,37E-16	0,347098	1	0,997	2,61E-12	9	PRDX6
MAP1A1	1,50E-16	0,31678	0,978	0,951	2,87E-12	9	MAP1A
CBR11	1,60E-16	0,283563	0,995	0,907	3,06E-12	9	CBR1
TIMM10	2,10E-16	0,265596	0,945	0,831	4,01E-12	9	TIMM10
NELFE	2,49E-16	0,252928	0,967	0,855	4,76E-12	9	NELFE
CDC371	2,53E-16	0,269015	1	0,966	4,84E-12	9	CDC37
GNB2	2,59E-16	0,265999	1	0,984	4,95E-12	9	GNB2
HNRNPU1	3,22E-16	0,271832	1	0,959	6,15E-12	9	HNRNPU
GAS62	3,25E-16	0,379316	1	0,988	6,22E-12	9	GAS6
PRR13	3,90E-16	0,266394	1	0,978	7,46E-12	9	PRR13
MRPL40	4,54E-16	0,255895	0,973	0,894	8,68E-12	9	MRPL40
SYNCRIP1	4,61E-16	0,282883	0,989	0,896	8,82E-12	9	SYNCRIP
ATG101	4,85E-16	0,25907	0,956	0,859	9,28E-12	9	ATG101
VIM3	5,39E-16	0,37043	1	1	1,03E-11	9	VIM
BDKRB1	5,45E-16	0,261093	0,885	0,656	1,04E-11	9	BDKRB1
VCP1	6,22E-16	0,259221	0,989	0,935	1,19E-11	9	VCP
VDAC31	6,48E-16	0,262829	0,989	0,915	1,24E-11	9	VDAC3
MCTS1	8,91E-16	0,26095	0,995	0,906	1,70E-11	9	MCTS1
PSMD41	9,05E-16	0,285403	0,995	0,954	1,73E-11	9	PSMD4
BNIP3	9,34E-16	0,262397	0,984	0,97	1,79E-11	9	BNIP3
MTCH21	9,73E-16	0,260809	0,989	0,945	1,86E-11	9	MTCH2
UGDH	9,80E-16	0,251917	0,945	0,753	1,87E-11	9	UGDH
PSMC11	1,26E-15	0,255901	0,978	0,891	2,41E-11	9	PSMC1
PRDX12	1,27E-15	0,352846	1	1	2,43E-11	9	PRDX1
TAF10	1,54E-15	0,267017	0,962	0,885	2,95E-11	9	TAF10
RSL1D1	1,84E-15	0,266147	1	0,957	3,51E-11	9	RSL1D1
MDH22	1,89E-15	0,274096	1	0,985	3,62E-11	9	MDH2
UQCC2	2,59E-15	0,273789	0,995	0,947	4,96E-11	9	UQCC2
SNRPB21	3,40E-15	0,266789	1	0,957	6,51E-11	9	SNRPB2
PFDN2	4,57E-15	0,336702	0,995	0,942	8,75E-11	9	PFDN2
MRPL22	5,72E-15	0,253964	0,978	0,871	1,09E-10	9	MRPL22
KRT101	6,04E-15	0,250818	0,995	0,987	1,15E-10	9	KRT10
CCT31	6,20E-15	0,269817	0,984	0,973	1,19E-10	9	CCT3
PRSS231	7,72E-15	0,261587	0,956	0,87	1,48E-10	9	PRSS23
PSMA51	8,03E-15	0,276503	0,995	0,967	1,53E-10	9	PSMA5
TUFM1	8,18E-15	0,262799	1	0,977	1,56E-10	9	TUFM
DEK	1,18E-14	0,304664	0,995	0,948	2,25E-10	9	DEK
DCBLD2	1,51E-14	0,383685	0,858	0,698	2,89E-10	9	DCBLD2
DCUN1D5	1,59E-14	0,250521	0,945	0,811	3,03E-10	9	DCUN1D5
UBE2E3	2,15E-14	0,25291	0,995	0,942	4,11E-10	9	UBE2E3
PLAT1	2,99E-14	0,613736	0,885	0,833	5,71E-10	9	PLAT
SKP12	3,15E-14	0,252164	1	0,999	6,02E-10	9	SKP1
TAF9	3,33E-14	0,252687	0,995	0,936	6,36E-10	9	TAF9
ATP5C12	5,18E-14	0,255437	1	0,983	9,90E-10	9	ATP5C1

POLR2F	6,45E-14	0,257167	0,995	0,971	1,23E-09	9	POLR2F
CD471	7,10E-14	0,278959	0,984	0,928	1,36E-09	9	CD47
TOP11	7,19E-14	0,252195	0,934	0,816	1,38E-09	9	TOP1
TNFRSF11B	7,82E-14	0,387338	0,962	0,899	1,49E-09	9	TNFRSF11B
ADI1	7,98E-14	0,251316	0,984	0,927	1,53E-09	9	ADI1
MESDC21	1,82E-13	0,255025	0,94	0,833	3,48E-09	9	MESDC2
PRDX22	2,41E-13	0,26597	0,995	0,985	4,61E-09	9	PRDX2
RPL39L	2,79E-13	0,257908	0,716	0,521	5,34E-09	9	RPL39L
S100A16	3,59E-13	0,432977	0,956	0,916	6,87E-09	9	S100A16
RBMX	4,26E-13	0,250167	0,984	0,915	8,14E-09	9	RBMX
PLAU1	6,49E-13	0,520719	0,88	0,75	1,24E-08	9	PLAU
PPIB2	1,40E-12	0,291429	1	1	2,68E-08	9	PPIB
BZW11	2,54E-12	0,264509	0,995	0,963	4,86E-08	9	BZW1
METRN1	4,32E-12	0,262243	0,978	0,89	8,25E-08	9	METRN
NOP561	9,85E-11	0,250981	0,836	0,699	1,88E-06	9	NOP56
CITED22	1,60E-10	0,324326	0,989	0,938	3,07E-06	9	CITED2
TUBB62	1,75E-10	0,268607	0,995	0,971	3,35E-06	9	TUBB6
BRIX12	2,25E-10	0,261878	0,929	0,81	4,30E-06	9	BRIX1
CKS1B	2,61E-10	0,419802	0,694	0,524	4,99E-06	9	CKS1B
ID1	3,27E-10	0,377615	0,858	0,753	6,25E-06	9	ID1
ATP2B12	4,57E-10	0,26145	1	0,985	8,74E-06	9	ATP2B1
FGF5	2,06E-09	0,263316	0,464	0,297	3,94E-05	9	FGF5
PTGR12	2,61E-09	0,302151	0,995	0,972	5,00E-05	9	PTGR1
CCND13	3,68E-09	0,458337	0,973	0,967	7,04E-05	9	CCND1
NLRP1	4,40E-09	0,296661	0,902	0,812	8,42E-05	9	NLRP1
NQO12	2,13E-08	0,40996	1	0,99	0,000407	9	NQO1
LXN3	1,66E-07	0,285615	0,984	0,936	0,003176	9	LXN
CEBPD1	4,44E-07	0,286146	0,989	0,982	0,008496	9	CEBPD
ADM1	6,00E-07	0,265009	0,962	0,948	0,011477	9	ADM
DDX21	6,88E-06	0,265998	0,913	0,807	0,131507	9	DDX21
PITX11	9,16E-06	0,315843	0,989	0,986	0,175149	9	PITX1
PHLDA1	9,21E-06	0,327148	0,858	0,803	0,176071	9	PHLDA1
SPC25	0	0,622526	0,8	0,009	0	10	SPC25
ESCO2	0	0,378113	0,727	0,003	0	10	ESCO2
KIF2C	1,91E-290	0,515218	0,727	0,009	3,66E-286	10	KIF2C
NUF2	6,61E-281	0,480799	0,709	0,009	1,26E-276	10	NUF2
BUB1B	5,31E-257	0,3391	0,636	0,007	1,02E-252	10	BUB1B
DEPDC1	1,13E-256	0,775842	0,873	0,02	2,16E-252	10	DEPDC1
ASF1B	2,86E-255	0,371667	0,709	0,011	5,47E-251	10	ASF1B
CDCA8	8,26E-252	0,601514	0,818	0,017	1,58E-247	10	CDCA8
CKAP2L	1,51E-249	0,339991	0,691	0,01	2,89E-245	10	CKAP2L
CASC5	3,21E-245	0,499087	0,818	0,018	6,14E-241	10	CASC5
CEP55	5,01E-241	1,013809	0,964	0,03	9,58E-237	10	CEP55
DTL	8,71E-238	0,309089	0,618	0,008	1,67E-233	10	DTL
NCAPH	1,26E-235	0,293778	0,636	0,009	2,41E-231	10	NCAPH
KIFC1	4,16E-235	0,466687	0,727	0,014	7,96E-231	10	KIFC1
SGOL1	1,52E-232	0,476461	0,745	0,015	2,90E-228	10	SGOL1
PKMYT1	1,53E-228	0,449035	0,745	0,016	2,92E-224	10	PKMYT1
CDC25C	1,48E-223	0,290969	0,6	0,008	2,83E-219	10	CDC25C
GTSE1	4,13E-222	0,902401	0,909	0,029	7,89E-218	10	GTSE1

NEK2	5,54E-222	0,313803	0,527	0,005	1,06E-217	10	NEK2
TTK	3,23E-217	0,275066	0,582	0,008	6,18E-213	10	TTK
MKI67	2,12E-215	1,481472	0,982	0,038	4,05E-211	10	MKI67
SKA1	3,48E-213	0,381819	0,818	0,023	6,65E-209	10	SKA1
HMMR	5,87E-210	0,695682	0,764	0,02	1,12E-205	10	HMMR
FAM111B	3,65E-207	0,314799	0,455	0,004	6,98E-203	10	FAM111B
KIF14	8,95E-206	0,368384	0,618	0,011	1,71E-201	10	KIF14
CENPU	6,32E-193	0,844034	0,945	0,041	1,21E-188	10	CENPU
HIST1H1B	2,89E-192	0,411165	0,473	0,005	5,52E-188	10	HIST1H1B
PBK	3,82E-192	0,92205	0,873	0,032	7,31E-188	10	PBK
KIF4A	3,95E-185	0,365574	0,6	0,012	7,55E-181	10	KIF4A
BUB1	8,67E-182	0,35687	0,673	0,017	1,66E-177	10	BUB1
NCAPG	6,27E-176	0,370436	0,727	0,022	1,20E-171	10	NCAPG
SHCBP1	9,84E-175	0,544807	0,745	0,025	1,88E-170	10	SHCBP1
CDCA2	7,46E-174	0,52073	0,764	0,027	1,43E-169	10	CDCA2
RAD51AP1	7,08E-172	0,54368	0,836	0,035	1,35E-167	10	RAD51AP1
DLGAP5	7,13E-164	0,670164	0,745	0,027	1,36E-159	10	DLGAP5
HJURP	2,43E-161	0,423409	0,691	0,023	4,64E-157	10	HJURP
SKA3	2,12E-160	0,3603	0,745	0,027	4,04E-156	10	SKA3
MYBL2	2,66E-159	0,566764	0,764	0,031	5,09E-155	10	MYBL2
CLSPN	3,85E-156	0,764324	0,818	0,037	7,36E-152	10	CLSPN
FAM64A	2,98E-146	0,557288	0,673	0,024	5,71E-142	10	FAM64A
CENPA	1,76E-144	0,862046	0,873	0,049	3,37E-140	10	CENPA
TACC3	5,38E-143	0,73652	0,873	0,05	1,03E-138	10	TACC3
E2F1	5,85E-143	0,465862	0,727	0,031	1,12E-138	10	E2F1
CDCA5	5,41E-142	0,420018	0,709	0,03	1,04E-137	10	CDCA5
UBE2C	7,83E-142	1,389487	0,836	0,046	1,50E-137	10	UBE2C
CENPF	4,39E-141	1,624554	0,945	0,063	8,40E-137	10	CENPF
LMNB1	4,45E-141	0,42098	0,745	0,034	8,51E-137	10	LMNB1
CDC6	2,40E-137	0,523476	0,618	0,022	4,60E-133	10	CDC6
MYBL1	1,42E-132	0,347158	0,673	0,028	2,72E-128	10	MYBL1
TRIP13	1,11E-128	0,434429	0,764	0,04	2,12E-124	10	TRIP13
ORC6	1,76E-128	0,511922	0,873	0,056	3,36E-124	10	ORC6
CDCA3	1,08E-127	0,79824	0,782	0,044	2,06E-123	10	CDCA3
RRM21	5,66E-121	1,256485	0,964	0,081	1,08E-116	10	RRM2
CCNA2	1,72E-118	1,035128	0,909	0,073	3,30E-114	10	CCNA2
AURKB	1,79E-115	0,90961	0,909	0,075	3,43E-111	10	AURKB
MND1	4,20E-115	0,259862	0,6	0,026	8,02E-111	10	MND1
FAM83D	5,34E-114	0,379981	0,582	0,025	1,02E-109	10	FAM83D
ANLN	3,55E-113	0,97807	0,927	0,08	6,79E-109	10	ANLN
CDC201	7,85E-108	1,414407	0,855	0,067	1,50E-103	10	CDC20
GINS2	1,03E-107	0,543389	0,818	0,06	1,97E-103	10	GINS2
MAD2L1	4,01E-107	0,752853	0,945	0,086	7,67E-103	10	MAD2L1
PRC1	4,06E-100	1,053359	0,909	0,09	7,77E-96	10	PRC1
TCF19	4,67E-100	0,450778	0,8	0,063	8,92E-96	10	TCF19
KIF11	5,86E-96	0,348005	0,709	0,049	1,12E-91	10	KIF11
CENPK	2,01E-94	0,778033	0,927	0,1	3,84E-90	10	CENPK
RACGAP1	5,60E-91	0,546387	0,836	0,079	1,07E-86	10	RACGAP1
ZWINT	1,19E-87	0,944988	1	0,135	2,27E-83	10	ZWINT
BIRC51	1,07E-85	1,396344	0,982	0,132	2,04E-81	10	BIRC5

AURKA	1,37E-84	1,190072	0,836	0,088	2,62E-80	10	AURKA
TOP2A	2,67E-84	1,610576	0,909	0,114	5,11E-80	10	TOP2A
SPC24	1,03E-83	0,436987	0,745	0,064	1,97E-79	10	SPC24
NUSAP1	3,12E-83	1,111317	0,909	0,11	5,96E-79	10	NUSAP1
ASPM	6,91E-83	0,990033	0,927	0,116	1,32E-78	10	ASPM
PLK1	7,04E-83	0,634925	0,636	0,046	1,35E-78	10	PLK1
CENPE	4,00E-82	0,649404	0,764	0,072	7,66E-78	10	CENPE
ATAD5	1,05E-81	0,389266	0,673	0,052	2,00E-77	10	ATAD5
CDT1	2,42E-81	0,640419	0,782	0,079	4,62E-77	10	CDT1
PSMC3IP	1,07E-80	0,498151	0,764	0,072	2,05E-76	10	PSMC3IP
KIF20B	1,77E-80	0,821551	0,927	0,122	3,39E-76	10	KIF20B
CDK1	1,12E-78	0,930482	0,8	0,087	2,14E-74	10	CDK1
MCM5	1,51E-77	0,505405	0,855	0,096	2,89E-73	10	MCM5
POC1A	9,39E-77	0,36974	0,8	0,082	1,79E-72	10	POC1A
BRCA2	5,18E-76	0,489566	0,673	0,057	9,90E-72	10	BRCA2
NDC80	3,95E-72	0,443508	0,782	0,086	7,55E-68	10	NDC80
RFC3	3,51E-70	0,335289	0,709	0,07	6,71E-66	10	RFC3
CDC45	2,64E-68	0,353896	0,527	0,037	5,04E-64	10	CDC45
NCAPG2	3,04E-68	0,3284	0,727	0,075	5,81E-64	10	NCAPG2
RAD51	2,85E-67	0,253929	0,582	0,047	5,45E-63	10	RAD51
CENPM1	4,86E-67	0,717903	0,945	0,159	9,30E-63	10	CENPM
FANCI	1,03E-66	0,331864	0,764	0,086	1,97E-62	10	FANCI
BRCA1	1,29E-65	0,36321	0,709	0,076	2,47E-61	10	BRCA1
UHRF1	1,41E-65	0,423958	0,709	0,077	2,70E-61	10	UHRF1
HIST1H1D	1,02E-64	0,438285	0,4	0,022	1,95E-60	10	HIST1H1D
SGOL2	2,95E-64	0,773905	0,909	0,151	5,64E-60	10	SGOL2
KIF23	7,76E-64	0,855107	0,836	0,124	1,48E-59	10	KIF23
CHAF1A	1,28E-59	0,515576	0,891	0,147	2,45E-55	10	CHAF1A
TPX2	1,59E-59	1,23109	0,909	0,172	3,05E-55	10	TPX2
ARHGAP11	3,23E-59	0,572188	0,8	0,12	6,17E-55	10	ARHGAP11A
FOXM1	7,88E-59	0,450055	0,8	0,115	1,51E-54	10	FOXM1
ATAD2	1,10E-58	0,727196	0,855	0,145	2,10E-54	10	ATAD2
MELK	5,56E-56	0,479284	0,873	0,154	1,06E-51	10	MELK
FEN1	2,12E-55	0,740472	0,818	0,139	4,05E-51	10	FEN1
FBXO5	2,28E-55	0,479993	0,745	0,106	4,36E-51	10	FBXO5
CCNB2	3,93E-54	0,637894	0,764	0,112	7,52E-50	10	CCNB2
MCM7	3,79E-53	0,954259	0,909	0,185	7,25E-49	10	MCM7
CCNB1	1,03E-52	1,268388	0,782	0,126	1,96E-48	10	CCNB1
CHAF1B	1,75E-51	0,25055	0,545	0,055	3,35E-47	10	CHAF1B
PRR11	9,50E-51	0,472553	0,655	0,087	1,82E-46	10	PRR11
KIF18A	1,03E-50	0,271167	0,618	0,073	1,97E-46	10	KIF18A
KIAA1524	2,43E-50	0,307456	0,618	0,074	4,64E-46	10	KIAA1524
TROAP1	1,61E-49	0,43238	0,636	0,08	3,08E-45	10	TROAP
WDR76	4,55E-49	0,31945	0,618	0,077	8,71E-45	10	WDR76
ZWILCH	1,76E-48	0,388764	0,836	0,153	3,37E-44	10	ZWILCH
C21orf58	7,73E-48	0,293457	0,636	0,083	1,48E-43	10	C21orf58
SPDL1	2,16E-46	0,532756	0,782	0,147	4,13E-42	10	SPDL1
UBE2T	3,84E-45	1,007148	0,982	0,315	7,34E-41	10	UBE2T
PSRC1	5,76E-45	0,459395	0,6	0,081	1,10E-40	10	PSRC1
RNASEH2A	7,90E-45	0,617512	0,855	0,187	1,51E-40	10	RNASEH2A

MXD3	7,95E-44	0,343364	0,691	0,108	1,52E-39	10	MXD3
HAUS8	2,52E-43	0,299202	0,618	0,086	4,81E-39	10	HAUS8
VRK1	3,63E-43	0,445308	0,8	0,153	6,95E-39	10	VRK1
TK11	5,13E-43	1,358	0,964	0,294	9,81E-39	10	TK1
DSN1	1,84E-42	0,28886	0,709	0,115	3,52E-38	10	DSN1
SMC4	4,18E-42	1,365787	1	0,421	7,99E-38	10	SMC4
TMPO	1,05E-41	0,954805	0,945	0,308	2,00E-37	10	TMPO
TYMS1	1,29E-41	1,04114	0,982	0,316	2,47E-37	10	TYMS
ECT2	2,03E-41	0,45115	0,873	0,207	3,89E-37	10	ECT2
KIAA01012	3,81E-41	2,078821	0,982	0,412	7,28E-37	10	KIAA0101
MCM4	1,47E-40	0,374722	0,764	0,147	2,81E-36	10	MCM4
WHSC1	2,97E-40	0,593362	0,964	0,317	5,69E-36	10	WHSC1
BARD1	1,29E-38	0,464768	0,836	0,201	2,47E-34	10	BARD1
CDCA7	2,54E-38	0,26417	0,527	0,069	4,86E-34	10	CDCA7
FOSL1	2,99E-38	0,907303	0,964	0,366	5,71E-34	10	FOSL1
CENPW1	8,48E-38	1,065625	0,964	0,364	1,62E-33	10	CENPW
GAS2L3	1,76E-37	0,33965	0,727	0,145	3,37E-33	10	GAS2L3
H2AFX	1,88E-37	1,165058	0,982	0,49	3,59E-33	10	H2AFX
CDCA4	4,73E-37	0,571978	0,782	0,193	9,05E-33	10	CDCA4
HMGB2	6,64E-37	2,189285	1	0,73	1,27E-32	10	HMGB2
DBF4	8,70E-37	0,515068	0,909	0,257	1,66E-32	10	DBF4
RFC2	1,16E-36	0,380409	0,8	0,178	2,22E-32	10	RFC2
NRGN	1,25E-36	0,282734	0,673	0,117	2,39E-32	10	NRGN
DEK1	2,71E-36	1,436642	1	0,95	5,18E-32	10	DEK
DNAJC9	2,90E-36	0,720166	0,982	0,38	5,54E-32	10	DNAJC9
NR2C2AP	8,73E-36	0,303725	0,709	0,132	1,67E-31	10	NR2C2AP
CKS1B1	7,98E-35	1,731534	0,982	0,526	1,53E-30	10	CKS1B
SMC2	1,11E-34	0,684988	0,873	0,269	2,13E-30	10	SMC2
H2AFZ1	1,43E-34	1,994153	1	0,997	2,74E-30	10	H2AFZ
KNSTRN	1,58E-34	0,5375	0,764	0,184	3,03E-30	10	KNSTRN
DHFR1	4,15E-34	0,756089	0,964	0,383	7,94E-30	10	DHFR
CKAP2	1,07E-33	0,9214	0,909	0,312	2,04E-29	10	CKAP2
MZT1	2,29E-33	0,768893	1	0,482	4,37E-29	10	MZT1
EZH2	2,30E-33	0,322401	0,636	0,123	4,40E-29	10	EZH2
RFWD3	5,86E-33	0,297689	0,727	0,162	1,12E-28	10	RFWD3
LIG1	1,41E-32	0,252225	0,6	0,103	2,69E-28	10	LIG1
UBE2S1	1,43E-32	1,95388	0,982	0,74	2,74E-28	10	UBE2S
CKS2	1,77E-32	2,147023	0,982	0,771	3,38E-28	10	CKS2
DIAPH3	3,51E-32	0,585956	0,964	0,367	6,71E-28	10	DIAPH3
GMNN	5,05E-32	0,667835	0,945	0,39	9,65E-28	10	GMNN
TEX30	8,17E-32	0,472389	0,891	0,279	1,56E-27	10	TEX30
STRA131	1,41E-31	1,10957	1	0,826	2,71E-27	10	STRA13
CENPN1	1,92E-31	0,613997	0,945	0,37	3,67E-27	10	CENPN
LRR1	1,12E-30	0,484409	0,909	0,326	2,14E-26	10	LRR1
DTYMK1	1,23E-30	1,14766	1	0,66	2,34E-26	10	DTYMK
RANBP11	1,32E-30	1,296818	1	0,946	2,52E-26	10	RANBP1
SYNE2	1,66E-30	0,255381	0,618	0,111	3,17E-26	10	SYNE2
DUT1	1,68E-29	0,950413	1	0,925	3,22E-25	10	DUT
CDKN31	2,14E-29	1,168215	0,873	0,334	4,09E-25	10	CDKN3
DNMT1	2,16E-29	0,918975	0,982	0,601	4,13E-25	10	DNMT1

LMNB2	3,23E-29	0,508181	0,927	0,337	6,18E-25	10	LMNB2
SNRPD11	3,50E-29	1,037567	1	0,968	6,70E-25	10	SNRPD1
TNFRSF12A	4,17E-29	1,26361	1	0,957	7,98E-25	10	TNFRSF12A
STMN11	4,26E-29	1,691616	0,982	0,802	8,14E-25	10	STMN1
ARHGAP11	4,76E-29	0,347914	0,636	0,136	9,09E-25	10	ARHGAP11B
YWHAH	5,10E-29	0,979618	1	0,959	9,76E-25	10	YWHAH
NCAPD3	8,66E-29	0,270842	0,655	0,142	1,66E-24	10	NCAPD3
HMGB11	1,14E-28	1,407444	1	0,997	2,19E-24	10	HMGB1
RRM1	1,42E-28	0,67176	0,964	0,429	2,72E-24	10	RRM1
HIST1H1A	1,79E-28	0,368343	0,364	0,044	3,42E-24	10	HIST1H1A
RPL39L1	1,98E-28	0,921672	0,927	0,526	3,78E-24	10	RPL39L
LSM41	2,43E-28	0,932525	1	0,964	4,65E-24	10	LSM4
C4orf46	3,35E-28	0,275395	0,745	0,193	6,41E-24	10	C4orf46
HELLS	3,95E-28	0,708995	0,782	0,257	7,55E-24	10	HELLS
SRSF71	9,30E-28	1,008205	1	0,852	1,78E-23	10	SRSF7
RFC4	1,86E-27	0,335793	0,709	0,177	3,55E-23	10	RFC4
LSM51	2,02E-27	0,85647	1	0,943	3,87E-23	10	LSM5
CCDC34	2,16E-27	0,556942	0,836	0,296	4,13E-23	10	CCDC34
PCNA	2,21E-27	0,929079	0,927	0,495	4,23E-23	10	PCNA
CYCS2	3,05E-27	0,982126	1	0,966	5,82E-23	10	CYCS
HNRNPAB	3,85E-27	0,927859	0,982	0,902	7,35E-23	10	HNRNPAB
MIS18A	4,23E-27	0,304712	0,673	0,161	8,09E-23	10	MIS18A
SNRPB1	4,56E-27	1,085214	1	0,981	8,72E-23	10	SNRPB
C9orf40	6,48E-27	0,327351	0,691	0,172	1,24E-22	10	C9orf40
USP1	9,50E-27	0,828374	0,927	0,531	1,82E-22	10	USP1
PGP	1,22E-26	0,760526	0,982	0,714	2,34E-22	10	PGP
MCM3	1,63E-26	0,449486	0,709	0,195	3,12E-22	10	MCM3
PTTG11	1,72E-26	1,691547	0,873	0,412	3,30E-22	10	PTTG1
SRSF22	1,82E-26	0,833545	1	0,954	3,48E-22	10	SRSF2
TUBA1B2	1,96E-26	1,744855	1	0,995	3,74E-22	10	TUBA1B
BCL2L12	2,01E-26	0,605074	0,909	0,427	3,85E-22	10	BCL2L12
HMGN21	2,62E-26	1,32056	1	0,994	5,02E-22	10	HMGN2
PA2G42	4,29E-26	0,888389	1	0,927	8,20E-22	10	PA2G4
TMEM106C	9,88E-26	0,791667	0,982	0,798	1,89E-21	10	TMEM106C
POLE3	2,11E-25	0,370936	0,891	0,333	4,03E-21	10	POLE3
POLA2	2,13E-25	0,279939	0,564	0,117	4,07E-21	10	POLA2
NUCKS1	4,19E-25	0,746641	1	0,994	8,01E-21	10	NUCKS1
NDC1	5,25E-25	0,326874	0,709	0,189	1,00E-20	10	NDC1
HN11	6,32E-25	1,126228	0,982	0,956	1,21E-20	10	HN1
PHF191	6,97E-25	0,85582	0,927	0,526	1,33E-20	10	PHF19
RAN2	7,16E-25	1,145285	1	0,996	1,37E-20	10	RAN
RMI2	1,01E-24	0,260221	0,564	0,121	1,94E-20	10	RMI2
WDR34	1,08E-24	0,535023	0,964	0,51	2,06E-20	10	WDR34
AURKAIP11	1,17E-24	0,654492	1	0,993	2,23E-20	10	AURKAIP1
ODC12	1,68E-24	0,940353	1	0,767	3,22E-20	10	ODC1
CENPH	1,72E-24	0,353707	0,727	0,215	3,29E-20	10	CENPH
HIST1H4C	1,92E-24	2,513346	0,964	0,89	3,67E-20	10	HIST1H4C
PRELID12	5,54E-24	0,739125	1	0,997	1,06E-19	10	PRELID1
NUDT15	6,13E-24	0,37356	0,909	0,351	1,17E-19	10	NUDT15
ANP32E1	8,02E-24	0,773841	0,945	0,603	1,53E-19	10	ANP32E

SIVA1	8,63E-24	0,730342	1	0,981	1,65E-19	10 SIVA1
IL7R1	8,85E-24	0,482581	0,745	0,219	1,69E-19	10 IL7R
HYLS1	1,23E-23	0,286635	0,709	0,199	2,36E-19	10 HYLS1
PLAU2	1,92E-23	1,557593	0,982	0,753	3,66E-19	10 PLAU
PAICS1	1,99E-23	0,58432	0,982	0,635	3,80E-19	10 PAICS
CDKN2C	2,20E-23	0,364769	0,764	0,237	4,22E-19	10 CDKN2C
TOMM401	2,24E-23	0,726802	1	0,684	4,29E-19	10 TOMM40
TUBB4B2	2,33E-23	1,565314	1	0,918	4,45E-19	10 TUBB4B
SNRPG1	2,83E-23	0,720026	1	0,988	5,42E-19	10 SNRPG
HIST1H1E	3,71E-23	0,370899	0,582	0,137	7,09E-19	10 HIST1H1E
C19orf48	4,13E-23	0,674826	0,964	0,575	7,90E-19	10 C19orf48
FABP51	4,75E-23	0,554864	0,927	0,414	9,08E-19	10 FABP5
TUBB63	5,41E-23	0,95895	1	0,972	1,03E-18	10 TUBB6
CEP78	6,39E-23	0,324574	0,764	0,242	1,22E-18	10 CEP78
HNRNPA2B	7,28E-23	0,878542	1	0,993	1,39E-18	10 HNRNPA2B1
PARP1	7,55E-23	0,742303	0,982	0,834	1,44E-18	10 PARP1
CTNNAL12	8,35E-23	0,883295	0,945	0,75	1,60E-18	10 CTNNAL1
ALYREF1	9,90E-23	0,575311	0,945	0,503	1,89E-18	10 ALYREF
TPM31	1,32E-22	0,67049	1	0,982	2,53E-18	10 TPM3
CBX5	1,66E-22	0,675839	0,982	0,777	3,17E-18	10 CBX5
HN1L	1,94E-22	0,356147	0,891	0,365	3,71E-18	10 HN1L
MT2A2	3,13E-22	1,858709	1	0,999	5,99E-18	10 MT2A
RHEB1	3,22E-22	0,66069	1	0,986	6,16E-18	10 RHEB
RPA31	3,76E-22	0,756902	0,964	0,63	7,20E-18	10 RPA3
LSM31	3,80E-22	0,642732	0,982	0,98	7,26E-18	10 LSM3
CSE1L	4,37E-22	0,455305	0,873	0,389	8,36E-18	10 CSE1L
ATP5G12	5,74E-22	0,761039	0,982	0,97	1,10E-17	10 ATP5G1
POP7	6,72E-22	0,620792	0,982	0,824	1,28E-17	10 POP7
PPP1R14B2	1,14E-21	1,159076	0,982	0,965	2,18E-17	10 PPP1R14B
NCAPD2	1,24E-21	0,278126	0,618	0,161	2,38E-17	10 NCAPD2
SSRP11	1,45E-21	0,656575	0,964	0,774	2,78E-17	10 SSRP1
SAC3D1	1,75E-21	0,508734	0,909	0,463	3,35E-17	10 SAC3D1
CD3EAP	1,88E-21	0,297647	0,673	0,192	3,60E-17	10 CD3EAP
CBX1	1,93E-21	0,658617	1	0,799	3,69E-17	10 CBX1
CDKN2D	2,30E-21	0,45751	0,764	0,268	4,39E-17	10 CDKN2D
KPNB1	3,17E-21	0,664254	1	0,972	6,06E-17	10 KPNB1
DRAP11	3,63E-21	0,779995	1	0,997	6,95E-17	10 DRAP1
NAP1L4	4,16E-21	0,528394	1	0,823	7,96E-17	10 NAP1L4
GPSM2	4,23E-21	0,269631	0,545	0,133	8,09E-17	10 GPSM2
TFAM	4,30E-21	0,447428	0,927	0,481	8,22E-17	10 TFAM
FUS1	6,18E-21	0,751192	1	0,93	1,18E-16	10 FUS
HNRNPR1	6,75E-21	0,629939	1	0,899	1,29E-16	10 HNRNPR
MIS18BP1	8,00E-21	0,34514	0,8	0,296	1,53E-16	10 MIS18BP1
TRIM59	8,23E-21	0,305112	0,618	0,169	1,57E-16	10 TRIM59
DKK13	9,57E-21	1,338553	1	0,688	1,83E-16	10 DKK1
RAD21	1,07E-20	0,633235	1	0,787	2,04E-16	10 RAD21
EBNA1BP2	1,16E-20	0,82206	0,982	0,824	2,22E-16	10 EBNA1BP2
ABHD11	1,33E-20	0,266511	0,727	0,237	2,54E-16	10 ABHD11
PHLDA22	1,46E-20	0,9395	1	0,966	2,80E-16	10 PHLDA2
UBE2I1	1,51E-20	0,598299	1	0,983	2,89E-16	10 UBE2I

EIF1AX1	1,57E-20	0,576411	1	0,968	3,01E-16	10 EIF1AX
IL11	1,67E-20	0,458441	0,564	0,141	3,20E-16	10 IL11
BUB3	1,88E-20	0,686991	1	0,731	3,59E-16	10 BUB3
SRSF32	2,30E-20	0,888	1	0,977	4,40E-16	10 SRSF3
HIRIP3	2,82E-20	0,263524	0,6	0,165	5,39E-16	10 HIRIP3
ANP32B	3,32E-20	0,685379	1	0,986	6,36E-16	10 ANP32B
PFN12	4,70E-20	0,588027	1	1	8,99E-16	10 PFN1
SRM2	5,44E-20	0,823968	1	0,968	1,04E-15	10 SRM
TFDP1	6,77E-20	0,555191	0,964	0,769	1,29E-15	10 TFDP1
HSPE11	7,94E-20	0,749687	1	0,988	1,52E-15	10 HSPE1
FAM111A	8,77E-20	0,57494	0,836	0,44	1,68E-15	10 FAM111A
NCL2	9,62E-20	0,898781	1	0,989	1,84E-15	10 NCL
EZR1	1,00E-19	0,503178	0,945	0,671	1,92E-15	10 EZR
SMS1	1,04E-19	0,817513	0,964	0,942	1,98E-15	10 SMS
SDF2L11	1,04E-19	0,702077	0,964	0,838	2,00E-15	10 SDF2L1
HNRNPD2	1,30E-19	0,682726	1	0,924	2,49E-15	10 HNRNPD
FST2	1,98E-19	1,435349	0,945	0,66	3,79E-15	10 FST
TUBB2	2,18E-19	0,946874	1	0,999	4,17E-15	10 TUBB
UBE2M1	4,63E-19	0,550559	0,982	0,806	8,85E-15	10 UBE2M
ARHGDIA1	4,89E-19	0,560425	1	0,976	9,35E-15	10 ARHGDIA
PSIP1	5,48E-19	0,566385	0,964	0,742	1,05E-14	10 PSIP1
COTL12	5,53E-19	0,670743	1	0,979	1,06E-14	10 COTL1
RTKN2	6,22E-19	0,266816	0,727	0,253	1,19E-14	10 RTKN2
NUP62	6,28E-19	0,433642	0,891	0,448	1,20E-14	10 NUP62
SMC1A	7,06E-19	0,382554	0,909	0,478	1,35E-14	10 SMC1A
CCP110	8,05E-19	0,251709	0,691	0,218	1,54E-14	10 CCP110
DDX211	9,15E-19	0,686521	0,982	0,81	1,75E-14	10 DDX21
NASP1	1,04E-18	0,669922	0,927	0,609	1,99E-14	10 NASP
C12orf752	1,34E-18	0,919529	1	0,996	2,56E-14	10 C12orf75
HERC41	1,54E-18	0,726287	0,982	0,807	2,95E-14	10 HERC4
HMGB31	1,65E-18	0,69958	0,873	0,495	3,15E-14	10 HMGB3
PDCD52	1,69E-18	0,540459	1	0,989	3,22E-14	10 PDCD5
ARPC22	2,37E-18	0,596294	1	0,999	4,54E-14	10 ARPC2
NUDT11	3,60E-18	0,511789	0,964	0,633	6,89E-14	10 NUDT1
LYAR	3,88E-18	0,461651	0,836	0,433	7,41E-14	10 LYAR
TUBA1C2	4,77E-18	1,105143	1	0,959	9,12E-14	10 TUBA1C
H3F3B1	5,06E-18	0,68142	1	0,997	9,68E-14	10 H3F3B
CALM22	6,99E-18	0,779045	1	1	1,34E-13	10 CALM2
PTGES31	7,23E-18	0,66482	0,982	0,985	1,38E-13	10 PTGES3
NXT1	7,92E-18	0,483301	0,891	0,518	1,51E-13	10 NXT1
HNRNPA32	9,28E-18	0,56568	1	0,97	1,77E-13	10 HNRNPA3
EIF5	9,74E-18	0,589335	1	0,972	1,86E-13	10 EIF5
NTMT11	1,01E-17	0,579038	1	0,932	1,93E-13	10 NTMT1
CBFB	1,12E-17	0,320882	0,891	0,411	2,14E-13	10 CBFB
NME13	1,12E-17	0,751111	1	0,939	2,15E-13	10 NME1
TPI13	1,23E-17	0,879777	1	0,996	2,36E-13	10 TPI1
ATAD3A	1,48E-17	0,374795	0,836	0,387	2,83E-13	10 ATAD3A
LRRC591	1,64E-17	0,65543	0,982	0,929	3,14E-13	10 LRRC59
POLD3	1,98E-17	0,256053	0,655	0,211	3,78E-13	10 POLD3
STIP11	2,14E-17	0,47263	0,982	0,659	4,09E-13	10 STIP1

RPA2	3,01E-17	0,345983	0,8	0,343	5,76E-13	10	RPA2
DDX39A1	3,08E-17	0,464068	0,855	0,422	5,88E-13	10	DDX39A
EMP32	3,31E-17	0,658497	1	0,999	6,32E-13	10	EMP3
PDAP11	3,38E-17	0,549851	1	0,971	6,46E-13	10	PDAP1
SLBP	3,65E-17	0,650954	0,982	0,747	6,97E-13	10	SLBP
H2AFV1	3,70E-17	0,704472	0,982	0,934	7,08E-13	10	H2AFV
NOLC1	5,57E-17	0,447402	0,855	0,418	1,06E-12	10	NOLC1
PHLDA11	5,68E-17	1,112943	0,982	0,803	1,09E-12	10	PHLDA1
PLP21	6,22E-17	0,711213	1	0,936	1,19E-12	10	PLP2
NCAPH2	6,83E-17	0,334048	0,764	0,312	1,31E-12	10	NCAPH2
TOPBP1	7,18E-17	0,260244	0,8	0,303	1,37E-12	10	TOPBP1
REEP4	7,85E-17	0,357208	0,691	0,282	1,50E-12	10	REEP4
CFL12	8,02E-17	0,574776	1	1	1,53E-12	10	CFL1
HSPD12	8,60E-17	0,759323	1	0,924	1,64E-12	10	HSPD1
NOP161	9,03E-17	0,482406	0,891	0,478	1,73E-12	10	NOP16
ATP2B13	9,16E-17	0,702866	1	0,985	1,75E-12	10	ATP2B1
CKLF	1,19E-16	0,590056	0,964	0,793	2,28E-12	10	CKLF
COX8A1	1,29E-16	0,485628	1	0,997	2,46E-12	10	COX8A
LSM2	1,42E-16	0,56272	0,964	0,888	2,71E-12	10	LSM2
EMP2	1,90E-16	0,352749	0,873	0,423	3,63E-12	10	EMP2
HMGA13	2,00E-16	1,230183	0,982	0,969	3,83E-12	10	HMGA1
GMPPB	2,12E-16	0,390588	0,891	0,483	4,06E-12	10	GMPPB
SVIP	2,21E-16	0,457413	0,909	0,634	4,23E-12	10	SVIP
BCL7C1	2,26E-16	0,575817	0,982	0,918	4,31E-12	10	BCL7C
UACA1	2,33E-16	0,646074	0,982	0,87	4,46E-12	10	UACA
ITGB1BP1	2,41E-16	0,532077	0,982	0,955	4,61E-12	10	ITGB1BP1
KPNA21	2,84E-16	1,091599	0,873	0,66	5,43E-12	10	KPNA2
TCEB1	2,89E-16	0,546591	0,982	0,986	5,52E-12	10	TCEB1
MAD2L2	3,10E-16	0,532986	0,927	0,65	5,93E-12	10	MAD2L2
SRRT	3,17E-16	0,305201	0,855	0,384	6,06E-12	10	SRRT
NHP22	3,32E-16	0,579508	0,982	0,967	6,35E-12	10	NHP2
EXOSC8	3,88E-16	0,463327	0,909	0,502	7,42E-12	10	EXOSC8
RNPS1	4,26E-16	0,530041	0,964	0,891	8,14E-12	10	RNPS1
SERPINE11	4,39E-16	0,380775	0,8	0,353	8,39E-12	10	SERPINE1
HAUS6	4,80E-16	0,298698	0,745	0,296	9,18E-12	10	HAUS6
TIMM101	4,87E-16	0,542638	0,964	0,835	9,31E-12	10	TIMM10
TIMM17A1	5,75E-16	0,481587	1	0,915	1,10E-11	10	TIMM17A
MYDGF3	7,32E-16	0,577407	1	0,997	1,40E-11	10	MYDGF
MRPL171	8,21E-16	0,546278	0,982	0,875	1,57E-11	10	MRPL17
ENO13	8,46E-16	1,032635	1	0,998	1,62E-11	10	ENO1
HMGN11	8,63E-16	0,471492	1	0,999	1,65E-11	10	HMGN1
SAP30	9,39E-16	0,350299	0,818	0,366	1,80E-11	10	SAP30
CLN6	9,70E-16	0,28043	0,818	0,381	1,85E-11	10	CLN6
TCOF1	1,13E-15	0,282968	0,8	0,405	2,16E-11	10	TCOF1
ERH1	1,24E-15	0,501267	1	0,994	2,37E-11	10	ERH
PRPS1	1,28E-15	0,480587	0,873	0,482	2,45E-11	10	PRPS1
MT1E3	1,29E-15	1,422284	1	0,962	2,46E-11	10	MT1E
MKKS1	1,63E-15	0,524455	0,982	0,929	3,11E-11	10	MKKS
SET1	1,76E-15	0,525479	1	0,996	3,37E-11	10	SET
DCBLD21	1,86E-15	0,561968	0,945	0,703	3,55E-11	10	DCBLD2

RPA1	1,90E-15	0,251794	0,818	0,336	3,62E-11	10	RPA1
NOP562	1,91E-15	0,604289	0,964	0,703	3,65E-11	10	NOP56
SNRPA11	1,92E-15	0,401216	0,964	0,587	3,67E-11	10	SNRPA1
CAV12	1,96E-15	0,628926	1	0,999	3,75E-11	10	CAV1
C20orf271	2,20E-15	0,474061	0,964	0,797	4,20E-11	10	C20orf27
PHB1	2,50E-15	0,494952	1	0,966	4,77E-11	10	PHB
RAD23A	2,84E-15	0,50364	1	0,972	5,43E-11	10	RAD23A
CARHSP1	2,89E-15	0,519648	1	0,991	5,53E-11	10	CARHSP1
CMC2	3,09E-15	0,468741	0,909	0,663	5,91E-11	10	CMC2
TPRKB	3,15E-15	0,396109	0,945	0,731	6,02E-11	10	TPRKB
SRRM11	3,34E-15	0,53907	1	0,924	6,38E-11	10	SRRM1
PGAM12	3,39E-15	0,617215	1	0,994	6,48E-11	10	PGAM1
PPIF	4,08E-15	0,393181	0,927	0,596	7,81E-11	10	PPIF
RBBP8	4,34E-15	0,299075	0,745	0,313	8,29E-11	10	RBBP8
TMEM1581	4,46E-15	0,653515	0,891	0,587	8,52E-11	10	TMEM158
MRPS121	4,77E-15	0,445089	0,982	0,938	9,12E-11	10	MRPS12
BOLA32	5,39E-15	0,493127	1	0,871	1,03E-10	10	BOLA3
BTG3	5,76E-15	0,45796	0,945	0,623	1,10E-10	10	BTG3
ARL6IP1	7,31E-15	1,076822	0,891	0,707	1,40E-10	10	ARL6IP1
TIMM8A	7,32E-15	0,311241	0,782	0,345	1,40E-10	10	TIMM8A
RBMX1	7,53E-15	0,514707	1	0,917	1,44E-10	10	RBMX
SMC31	7,73E-15	0,481266	1	0,771	1,48E-10	10	SMC3
SERBP11	7,73E-15	0,567989	1	0,991	1,48E-10	10	SERBP1
ANAPC11	1,06E-14	0,424128	1	0,997	2,03E-10	10	ANAPC11
MRPL121	1,06E-14	0,500571	1	0,924	2,03E-10	10	MRPL12
MTDH1	1,08E-14	0,446902	1	0,993	2,06E-10	10	MTDH
ARPC5L	1,09E-14	0,500395	0,945	0,825	2,09E-10	10	ARPC5L
GRK6	1,13E-14	0,332809	0,818	0,437	2,16E-10	10	GRK6
PEG10	1,30E-14	0,263102	0,545	0,164	2,48E-10	10	PEG10
CENPV	1,37E-14	0,426452	0,909	0,617	2,61E-10	10	CENPV
HMGA2	1,41E-14	0,357991	0,618	0,226	2,70E-10	10	HMGA2
NT5E1	1,55E-14	0,466667	0,909	0,531	2,97E-10	10	NT5E
GAS63	1,58E-14	0,76362	1	0,989	3,02E-10	10	GAS6
DCTPP1	1,69E-14	0,338319	0,818	0,423	3,24E-10	10	DCTPP1
CBX3	1,77E-14	0,410551	1	0,983	3,39E-10	10	CBX3
G3BP1	1,99E-14	0,366617	0,982	0,797	3,81E-10	10	G3BP1
COX202	2,27E-14	0,518413	0,982	0,943	4,34E-10	10	COX20
CACYBP2	2,29E-14	0,527435	0,982	0,812	4,38E-10	10	CACYBP
RBM8A1	2,85E-14	0,616241	0,982	0,921	5,45E-10	10	RBM8A
PIN11	3,24E-14	0,418375	1	0,964	6,19E-10	10	PIN1
ARF6	3,35E-14	0,477611	1	0,92	6,41E-10	10	ARF6
SPCS3	3,60E-14	0,424947	1	0,863	6,88E-10	10	SPCS3
MANF2	3,69E-14	0,590063	0,982	0,879	7,06E-10	10	MANF
DKC1	4,09E-14	0,392109	0,873	0,484	7,81E-10	10	DKC1
MRPL142	4,95E-14	0,47761	1	0,976	9,47E-10	10	MRPL14
KIF22	5,04E-14	0,454269	0,927	0,684	9,63E-10	10	KIF22
GPATCH41	5,07E-14	0,465052	0,927	0,644	9,70E-10	10	GPATCH4
CMSS1	5,54E-14	0,304817	0,745	0,343	1,06E-09	10	CMSS1
STUB11	5,71E-14	0,482182	1	0,984	1,09E-09	10	STUB1
TMEM14B2	6,18E-14	0,509639	0,982	0,973	1,18E-09	10	TMEM14B

SMAGP	6,40E-14	0,293199	0,836	0,407	1,22E-09	10	SMAGP
HNRNPDL1	8,26E-14	0,560975	0,982	0,964	1,58E-09	10	HNRNPDL
CALM31	1,15E-13	0,516526	1	0,971	2,19E-09	10	CALM3
DDX461	1,16E-13	0,444043	1	0,946	2,21E-09	10	DDX46
RAB3B	1,28E-13	0,41629	0,964	0,678	2,44E-09	10	RAB3B
POLD21	1,36E-13	0,47594	0,982	0,88	2,60E-09	10	POLD2
MRPL511	1,50E-13	0,430799	1	0,993	2,87E-09	10	MRPL51
HAT11	1,53E-13	0,373981	0,909	0,462	2,93E-09	10	HAT1
FGF51	1,70E-13	0,627364	0,655	0,3	3,26E-09	10	FGF5
RRP7A	1,83E-13	0,396946	0,873	0,549	3,50E-09	10	RRP7A
GPN3	1,89E-13	0,264702	0,764	0,357	3,62E-09	10	GPN3
MEA1	1,96E-13	0,382391	0,982	0,918	3,75E-09	10	MEA1
C11orf24	2,01E-13	0,366277	0,982	0,722	3,84E-09	10	C11orf24
GTF3A1	2,03E-13	0,522077	0,982	0,958	3,88E-09	10	GTF3A
SNRPE1	2,03E-13	0,433727	0,964	0,993	3,89E-09	10	SNRPE
HIST2H2AC	2,05E-13	0,392613	0,582	0,223	3,93E-09	10	HIST2H2AC
EMC9	2,11E-13	0,355171	0,873	0,486	4,04E-09	10	EMC9
KIF5B1	2,17E-13	0,553442	1	0,972	4,15E-09	10	KIF5B
MRPL201	2,24E-13	0,439701	0,982	0,975	4,29E-09	10	MRPL20
UAP1	2,29E-13	0,463898	0,891	0,511	4,38E-09	10	UAP1
RGMB	2,30E-13	0,36577	0,8	0,395	4,39E-09	10	RGMB
RER12	2,34E-13	0,412983	1	0,981	4,47E-09	10	RER1
FAM101B	2,35E-13	0,517545	0,891	0,758	4,49E-09	10	FAM101B
GGCT	2,49E-13	0,403946	0,927	0,712	4,77E-09	10	GGCT
PTP4A2	2,55E-13	0,452378	0,982	0,985	4,88E-09	10	PTP4A2
CFAP20	2,62E-13	0,32415	0,873	0,526	5,00E-09	10	CFAP20
EIF5A3	2,99E-13	0,577519	0,964	0,898	5,72E-09	10	EIF5A
RBM17	3,10E-13	0,452047	1	0,913	5,93E-09	10	RBM17
SRSF91	3,13E-13	0,470603	1	0,979	5,99E-09	10	SRSF9
IFRD2	3,54E-13	0,386203	0,945	0,634	6,78E-09	10	IFRD2
TUBG1	3,65E-13	0,444504	0,891	0,551	6,97E-09	10	TUBG1
SLC2A4RG	3,65E-13	0,404919	0,927	0,731	6,97E-09	10	SLC2A4RG
CORO1C	3,81E-13	0,419147	1	0,917	7,29E-09	10	CORO1C
CALM12	4,04E-13	0,460046	1	0,991	7,72E-09	10	CALM1
SUPT16H1	4,23E-13	0,42765	0,982	0,728	8,09E-09	10	SUPT16H
SLC20A1	4,25E-13	0,390399	0,855	0,457	8,12E-09	10	SLC20A1
LDHA3	4,41E-13	0,824427	1	0,993	8,43E-09	10	LDHA
SKA21	4,55E-13	0,452522	0,964	0,843	8,70E-09	10	SKA2
ICMT	4,66E-13	0,361587	0,945	0,713	8,91E-09	10	ICMT
MRTO4	5,18E-13	0,409697	0,855	0,544	9,91E-09	10	MRTO4
EMG11	5,26E-13	0,397811	0,855	0,588	1,01E-08	10	EMG1
POLR2F1	5,44E-13	0,40698	0,982	0,973	1,04E-08	10	POLR2F
EIF4E	5,61E-13	0,399521	0,964	0,785	1,07E-08	10	EIF4E
PPM1G	5,76E-13	0,517801	0,964	0,883	1,10E-08	10	PPM1G
PRKDC1	5,92E-13	0,506442	0,964	0,833	1,13E-08	10	PRKDC
SUZ12	6,08E-13	0,337237	0,891	0,599	1,16E-08	10	SUZ12
RDX1	6,38E-13	0,44875	1	0,945	1,22E-08	10	RDX
RASSF1	6,62E-13	0,413984	0,909	0,597	1,27E-08	10	RASSF1
SIGMAR11	7,06E-13	0,377106	0,909	0,707	1,35E-08	10	SIGMAR1
RNF126	7,14E-13	0,414377	0,891	0,61	1,37E-08	10	RNF126

NEDD82	7,57E-13	0,344671	1	0,999	1,45E-08	10	NEDD8
OGFRL11	9,12E-13	0,474181	0,927	0,665	1,74E-08	10	OGFRL1
ITGA21	9,50E-13	0,474823	0,8	0,477	1,82E-08	10	ITGA2
THOP1	1,13E-12	0,339602	0,8	0,427	2,16E-08	10	THOP1
WBP11	1,25E-12	0,39734	0,945	0,749	2,39E-08	10	WBP11
MRPL571	1,32E-12	0,402706	1	0,977	2,52E-08	10	MRPL57
ARHGAP18	1,36E-12	0,324498	0,927	0,532	2,61E-08	10	ARHGAP18
SNAPC11	1,50E-12	0,376303	0,909	0,583	2,86E-08	10	SNAPC1
UBE2N1	1,50E-12	0,494607	1	0,925	2,87E-08	10	UBE2N
SRP91	1,56E-12	0,438624	1	0,977	2,97E-08	10	SRP9
ARF11	1,56E-12	0,399278	1	0,99	2,98E-08	10	ARF1
SNRNP40	1,62E-12	0,410668	0,891	0,674	3,10E-08	10	SNRNP40
EXOSC9	1,64E-12	0,304975	0,636	0,269	3,14E-08	10	EXOSC9
UQCR102	1,74E-12	0,416743	1	0,995	3,34E-08	10	UQCR10
RPL22L13	1,91E-12	0,642824	1	0,973	3,66E-08	10	RPL22L1
WDR5	2,08E-12	0,264104	0,727	0,344	3,98E-08	10	WDR5
HDGF1	2,45E-12	0,48303	0,964	0,863	4,68E-08	10	HDGF
WDR54	2,61E-12	0,273076	0,873	0,484	4,99E-08	10	WDR54
SFXN1	2,63E-12	0,300253	0,891	0,497	5,04E-08	10	SFXN1
SNRPF1	2,67E-12	0,411906	0,982	0,982	5,11E-08	10	SNRPF
SNU132	2,72E-12	0,444415	1	0,995	5,19E-08	10	SNU13
SUMO31	3,20E-12	0,480861	1	0,954	6,12E-08	10	SUMO3
MSH6	3,39E-12	0,270948	0,673	0,282	6,49E-08	10	MSH6
HNRNPM2	3,77E-12	0,572034	1	0,878	7,22E-08	10	HNRNPM
RNF145	3,98E-12	0,418665	1	0,818	7,60E-08	10	RNF145
PSMD22	4,00E-12	0,614594	1	0,892	7,65E-08	10	PSMD2
HNRNPUL1	4,10E-12	0,352735	0,982	0,851	7,83E-08	10	HNRNPUL1
CLIC13	4,28E-12	0,557116	1	0,999	8,18E-08	10	CLIC1
SRSF1	4,52E-12	0,377551	0,945	0,8	8,65E-08	10	SRSF1
CHCHD21	4,74E-12	0,316292	1	0,999	9,06E-08	10	CHCHD2
ARPC1A1	4,78E-12	0,424121	1	0,993	9,14E-08	10	ARPC1A
MRPL42	5,00E-12	0,384904	0,982	0,797	9,57E-08	10	MRPL42
POLR2D	5,03E-12	0,275417	0,8	0,393	9,63E-08	10	POLR2D
SFPQ1	5,15E-12	0,548979	0,982	0,942	9,84E-08	10	SFPQ
BAZ1A	5,29E-12	0,336834	0,945	0,681	1,01E-07	10	BAZ1A
YBX11	5,32E-12	0,339178	1	1	1,02E-07	10	YBX1
RNASEH1	5,34E-12	0,34893	0,945	0,805	1,02E-07	10	RNASEH1
RPS19BP11	5,59E-12	0,380351	1	0,974	1,07E-07	10	RPS19BP1
FTSJ2	5,84E-12	0,256088	0,782	0,394	1,12E-07	10	FTSJ2
CDKN2AIPI	5,86E-12	0,33494	0,836	0,527	1,12E-07	10	CDKN2AIPNL
POLR3K	6,00E-12	0,332342	0,855	0,528	1,15E-07	10	POLR3K
AP2S11	6,64E-12	0,380258	1	1	1,27E-07	10	AP2S1
TMED93	8,34E-12	0,523923	1	0,988	1,59E-07	10	TMED9
IKBIP1	9,23E-12	0,437581	1	0,817	1,76E-07	10	IKBIP
GGH1	9,30E-12	0,380792	0,836	0,462	1,78E-07	10	GGH
MLF22	9,41E-12	0,427442	1	0,983	1,80E-07	10	MLF2
EIF2S1	9,59E-12	0,40165	0,945	0,713	1,83E-07	10	EIF2S1
PSMC52	9,82E-12	0,434981	1	0,942	1,88E-07	10	PSMC5
ASPH3	9,88E-12	0,441164	1	0,986	1,89E-07	10	ASPH
NT5DC21	1,03E-11	0,330949	0,945	0,632	1,97E-07	10	NT5DC2

UQCC21	1,03E-11	0,415235	1	0,949	1,97E-07	10	UQCC2
CCDC14	1,06E-11	0,395637	0,945	0,745	2,03E-07	10	CCDC14
RGS201	1,16E-11	0,257384	0,782	0,364	2,22E-07	10	RGS20
SLC16A33	1,21E-11	0,576618	1	0,911	2,31E-07	10	SLC16A3
SMCHD1	1,26E-11	0,315066	0,982	0,721	2,40E-07	10	SMCHD1
EIF62	1,32E-11	0,459427	1	0,964	2,53E-07	10	EIF6
NF2	1,40E-11	0,350317	0,927	0,687	2,67E-07	10	NF2
WDR43	1,41E-11	0,326126	0,873	0,549	2,69E-07	10	WDR43
ANAPC5	1,52E-11	0,327109	1	0,84	2,90E-07	10	ANAPC5
PCBP1	1,56E-11	0,380764	1	0,981	2,98E-07	10	PCBP1
HNRNPH31	1,73E-11	0,47417	0,964	0,842	3,30E-07	10	HNRNPH3
TRA2B	1,76E-11	0,416355	0,964	0,83	3,37E-07	10	TRA2B
ADRM12	1,90E-11	0,468022	1	0,964	3,63E-07	10	ADRM1
ILF22	2,10E-11	0,480039	1	0,859	4,02E-07	10	ILF2
PHF5A	2,15E-11	0,37756	0,945	0,754	4,11E-07	10	PHF5A
PPP2CA	2,25E-11	0,375282	0,945	0,799	4,31E-07	10	PPP2CA
MRPL11	2,27E-11	0,339632	1	0,956	4,34E-07	10	MRPL11
CCDC137	2,28E-11	0,40955	0,855	0,576	4,36E-07	10	CCDC137
MAPRE1	2,29E-11	0,421099	0,964	0,818	4,38E-07	10	MAPRE1
CISD31	2,31E-11	0,360549	0,964	0,847	4,42E-07	10	CISD3
CALR3	2,40E-11	0,530842	1	0,998	4,59E-07	10	CALR
VPS4A	2,50E-11	0,323607	0,909	0,645	4,77E-07	10	VPS4A
COX171	2,52E-11	0,445404	1	0,89	4,82E-07	10	COX17
CHAMP1	2,59E-11	0,296253	0,964	0,636	4,96E-07	10	CHAMP1
GTF3C61	2,70E-11	0,401298	0,982	0,942	5,15E-07	10	GTF3C6
PFDN21	2,74E-11	0,45745	1	0,945	5,24E-07	10	PFDN2
PITHD1	2,74E-11	0,311047	0,855	0,552	5,24E-07	10	PITHD1
PPIH	2,87E-11	0,292941	0,782	0,43	5,49E-07	10	PPIH
PTBP1	2,97E-11	0,383818	0,964	0,78	5,68E-07	10	PTBP1
PKM3	3,17E-11	0,687748	1	0,999	6,06E-07	10	PKM
FAM136A	3,38E-11	0,381216	0,927	0,754	6,47E-07	10	FAM136A
IMP4	3,64E-11	0,378308	0,945	0,764	6,96E-07	10	IMP4
TMEM160	3,78E-11	0,376229	1	0,925	7,24E-07	10	TMEM160
MORF4L21	4,18E-11	0,494191	1	0,994	7,99E-07	10	MORF4L2
CLTB2	4,39E-11	0,455435	1	0,982	8,40E-07	10	CLTB
LSM71	4,75E-11	0,427359	0,964	0,985	9,08E-07	10	LSM7
PRADC1	5,30E-11	0,317128	0,891	0,621	1,01E-06	10	PRADC1
BRIX13	5,35E-11	0,795948	0,964	0,815	1,02E-06	10	BRIX1
SRGN2	5,37E-11	0,576933	0,891	0,64	1,03E-06	10	SRGN
RHNO1	5,73E-11	0,298953	0,836	0,559	1,10E-06	10	RHNO1
SYNCRIP2	6,15E-11	0,43634	1	0,9	1,18E-06	10	SYNCRIP
DDX18	6,46E-11	0,335247	0,964	0,783	1,24E-06	10	DDX18
STOML21	6,78E-11	0,409005	0,964	0,971	1,30E-06	10	STOML2
C3orf14	7,48E-11	0,39148	0,873	0,712	1,43E-06	10	C3orf14
HNRNPU2	9,34E-11	0,37004	1	0,96	1,79E-06	10	HNRNPU
SMTN1	9,52E-11	0,424169	0,927	0,646	1,82E-06	10	SMTN
EMC61	9,57E-11	0,402497	0,982	0,939	1,83E-06	10	EMC6
SH3BGRL32	9,72E-11	0,500906	1	0,997	1,86E-06	10	SH3BGRL3
MAGOHB	1,21E-10	0,298384	0,836	0,58	2,31E-06	10	MAGOHB
RPL7L11	1,21E-10	0,409218	0,982	0,881	2,32E-06	10	RPL7L1

CCM2	1,40E-10	0,363769	0,927	0,768	2,67E-06	10	CCM2
DAZAP11	1,41E-10	0,341462	0,927	0,719	2,70E-06	10	DAZAP1
BANF11	1,41E-10	0,448745	1	0,966	2,70E-06	10	BANF1
PMAIP1	1,43E-10	0,345688	0,836	0,547	2,73E-06	10	PMAIP1
CAV21	1,52E-10	0,412528	0,982	0,919	2,91E-06	10	CAV2
NUTF21	1,56E-10	0,335968	0,964	0,978	2,99E-06	10	NUTF2
ILF31	1,58E-10	0,346201	0,982	0,801	3,02E-06	10	ILF3
AXL3	1,61E-10	0,676997	1	0,928	3,07E-06	10	AXL
CCND14	1,69E-10	0,673266	1	0,966	3,23E-06	10	CCND1
C1orf35	1,70E-10	0,272088	0,836	0,546	3,25E-06	10	C1orf35
SPATS2L	1,81E-10	0,379254	1	0,988	3,45E-06	10	SPATS2L
CDK4	2,05E-10	0,356161	0,982	0,978	3,92E-06	10	CDK4
GTF2A21	2,23E-10	0,425547	0,982	0,956	4,26E-06	10	GTF2A2
XPO1	2,27E-10	0,408223	0,982	0,713	4,35E-06	10	XPO1
PDIA63	2,31E-10	0,49741	1	0,975	4,42E-06	10	PDIA6
CENPT	2,34E-10	0,251459	0,782	0,423	4,48E-06	10	CENPT
GAR1	2,35E-10	0,292978	0,945	0,577	4,49E-06	10	GAR1
WBP4	2,38E-10	0,334492	0,964	0,722	4,55E-06	10	WBP4
SRRM2	2,52E-10	0,359282	1	0,964	4,81E-06	10	SRRM2
ZPR1	2,52E-10	0,287095	0,891	0,551	4,83E-06	10	ZPR1
DBI1	2,59E-10	0,470182	1	0,999	4,95E-06	10	DBI
ARL4A	2,90E-10	0,332107	0,855	0,541	5,54E-06	10	ARL4A
UBALD2	2,99E-10	0,311384	0,764	0,484	5,72E-06	10	UBALD2
HSPB111	2,99E-10	0,353477	1	0,898	5,72E-06	10	HSPB11
UPP1	3,12E-10	0,314583	0,909	0,695	5,97E-06	10	UPP1
C1QBP1	3,13E-10	0,517342	1	0,962	5,98E-06	10	C1QBP
RECQL	3,68E-10	0,289691	0,855	0,571	7,04E-06	10	RECQL
SLIRP1	3,73E-10	0,371858	0,982	0,988	7,13E-06	10	SLIRP
SELK	3,76E-10	0,342836	0,982	0,927	7,20E-06	10	SELK
H1FX	3,88E-10	0,486367	0,927	0,687	7,41E-06	10	H1FX
ARHGAP22	4,31E-10	0,255291	0,618	0,3	8,24E-06	10	ARHGAP22
SRSF10	4,31E-10	0,402236	0,982	0,86	8,24E-06	10	SRSF10
LOXL21	4,69E-10	0,376912	0,982	0,785	8,97E-06	10	LOXL2
CKAP42	5,08E-10	0,392815	1	0,986	9,71E-06	10	CKAP4
SAE1	5,26E-10	0,336282	0,909	0,729	1,01E-05	10	SAE1
EEF1E11	5,33E-10	0,346431	0,891	0,635	1,02E-05	10	EEF1E1
SF3B21	5,41E-10	0,409612	1	0,942	1,03E-05	10	SF3B2
MRPS341	5,52E-10	0,387859	1	0,954	1,06E-05	10	MRPS34
TOMM221	5,64E-10	0,399159	1	0,972	1,08E-05	10	TOMM22
FUBP1	5,92E-10	0,330045	0,855	0,547	1,13E-05	10	FUBP1
RAB321	6,07E-10	0,391435	0,982	0,991	1,16E-05	10	RAB32
ARL6IP6	6,26E-10	0,290386	0,745	0,406	1,20E-05	10	ARL6IP6
ID11	6,36E-10	0,655689	0,945	0,756	1,22E-05	10	ID1
MRPL18	6,70E-10	0,368517	1	0,916	1,28E-05	10	MRPL18
DERA	6,86E-10	0,285363	0,945	0,649	1,31E-05	10	DERA
CHCHD3	7,50E-10	0,293413	0,982	0,848	1,44E-05	10	CHCHD3
NDUFS61	7,63E-10	0,33037	1	0,997	1,46E-05	10	NDUFS6
DPM2	8,60E-10	0,317582	0,927	0,745	1,65E-05	10	DPM2
RALBP1	8,99E-10	0,349844	1	0,947	1,72E-05	10	RALBP1
NIPA2	9,06E-10	0,323702	0,945	0,803	1,73E-05	10	NIPA2

HSP90AA1:	9,45E-10	0,68118	1	0,999	1,81E-05	10	HSP90AA1
VDAC32	9,73E-10	0,384594	0,982	0,919	1,86E-05	10	VDAC3
CKAP5	9,78E-10	0,276345	0,782	0,473	1,87E-05	10	CKAP5
ARPP19	1,03E-09	0,330821	0,945	0,798	1,96E-05	10	ARPP19
RPL26L11	1,12E-09	0,36001	1	0,943	2,13E-05	10	RPL26L1
LBR	1,13E-09	0,262565	0,818	0,493	2,17E-05	10	LBR
C20orf241	1,20E-09	0,360545	1	0,918	2,30E-05	10	C20orf24
RRS1	1,22E-09	0,26607	0,691	0,374	2,33E-05	10	RRS1
EXOSC3	1,22E-09	0,323466	0,855	0,59	2,33E-05	10	EXOSC3
WSB2	1,25E-09	0,341383	0,945	0,766	2,39E-05	10	WSB2
ATP5G33	1,27E-09	0,358603	1	0,997	2,43E-05	10	ATP5G3
MCMBP	1,29E-09	0,255346	0,818	0,456	2,46E-05	10	MCMBP
APIP	1,32E-09	0,283051	0,927	0,618	2,53E-05	10	APIP
HYI1	1,36E-09	0,272399	0,945	0,659	2,59E-05	10	HYI
POLR2H	1,42E-09	0,27468	0,909	0,713	2,72E-05	10	POLR2H
FARSA	1,45E-09	0,285364	0,836	0,511	2,77E-05	10	FARSA
MYC	1,57E-09	0,269804	0,727	0,408	3,00E-05	10	MYC
SLC35B2	1,64E-09	0,346754	0,982	0,838	3,14E-05	10	SLC35B2
PLAUR1	1,69E-09	0,554211	0,909	0,696	3,24E-05	10	PLAUR
MRPL27	1,78E-09	0,352866	1	0,958	3,41E-05	10	MRPL27
MGLL	1,79E-09	0,397323	0,964	0,933	3,42E-05	10	MGLL
C9orf142	1,82E-09	0,343615	0,927	0,763	3,48E-05	10	C9orf142
RPS261	1,89E-09	0,271134	1	1	3,61E-05	10	RPS26
PPP1CA1	1,92E-09	0,375022	1	0,968	3,67E-05	10	PPP1CA
ABCE1	1,92E-09	0,342294	0,855	0,614	3,68E-05	10	ABCE1
ACTN41	1,93E-09	0,329056	1	0,951	3,70E-05	10	ACTN4
LYPD1	2,02E-09	0,27101	0,636	0,296	3,87E-05	10	LYPD1
CYC12	2,36E-09	0,467369	0,982	0,962	4,50E-05	10	CYC1
GADD45GII	2,53E-09	0,332686	1	0,993	4,84E-05	10	GADD45GIP1
TOMM34	2,53E-09	0,268284	0,764	0,424	4,84E-05	10	TOMM34
PTMS1	2,61E-09	0,368146	1	0,998	4,98E-05	10	PTMS
SNRNP25	2,65E-09	0,281831	0,982	0,893	5,07E-05	10	SNRNP25
ARPC51	2,65E-09	0,394505	1	0,99	5,08E-05	10	ARPC5
PTP4A1	2,67E-09	0,321263	0,982	0,895	5,11E-05	10	PTP4A1
MRPL55	2,71E-09	0,307486	0,982	0,914	5,18E-05	10	MRPL55
XRCC52	2,83E-09	0,569809	0,964	0,938	5,40E-05	10	XRCC5
TIMM50	3,14E-09	0,277312	0,945	0,7	6,00E-05	10	TIMM50
HGF1	3,18E-09	0,491625	1	0,802	6,08E-05	10	HGF
MRPS11	3,22E-09	0,360783	0,964	0,907	6,15E-05	10	MRPS11
GNB21	3,62E-09	0,314825	0,982	0,985	6,91E-05	10	GNB2
FAM207A	3,94E-09	0,307659	0,927	0,757	7,54E-05	10	FAM207A
PSMG3	4,27E-09	0,299636	0,891	0,683	8,17E-05	10	PSMG3
RIC8A	4,33E-09	0,264574	0,945	0,714	8,28E-05	10	RIC8A
KRT102	4,36E-09	0,398956	1	0,988	8,33E-05	10	KRT10
BRD7	4,48E-09	0,275975	0,982	0,79	8,56E-05	10	BRD7
RBBP71	4,57E-09	0,353294	0,964	0,847	8,75E-05	10	RBBP7
SMARCA4	4,71E-09	0,311278	0,927	0,773	9,01E-05	10	SMARCA4
NAA50	4,93E-09	0,371899	0,964	0,866	9,42E-05	10	NAA50
NUDC2	5,03E-09	0,45468	0,982	0,923	9,62E-05	10	NUDC
TRIM28	5,61E-09	0,340309	0,964	0,768	0,000107	10	TRIM28

SCML1	6,05E-09	0,329922	0,836	0,595	0,000116	10	SCML1
RNF26	6,29E-09	0,298601	0,891	0,655	0,00012	10	RNF26
ACTB1	6,62E-09	0,338205	1	1	0,000127	10	ACTB
LSM6	7,20E-09	0,311522	0,927	0,831	0,000138	10	LSM6
CISD2	7,62E-09	0,326938	0,945	0,812	0,000146	10	CISD2
MRPL521	7,87E-09	0,389295	0,982	0,972	0,00015	10	MRPL52
COA1	8,24E-09	0,312546	0,964	0,796	0,000158	10	COA1
NUCB21	8,34E-09	0,335074	1	0,915	0,00016	10	NUCB2
SNRNP702	8,84E-09	0,425393	0,982	0,881	0,000169	10	SNRNP70
EIF3J	9,63E-09	0,302789	1	0,894	0,000184	10	EIF3J
EWSR11	9,98E-09	0,303803	0,964	0,835	0,000191	10	EWSR1
CCT52	1,03E-08	0,514364	0,964	0,878	0,000197	10	CCT5
TAF91	1,04E-08	0,304016	0,982	0,939	0,000198	10	TAF9
CD3201	1,08E-08	0,296028	0,909	0,666	0,000206	10	CD320
WNT5B	1,10E-08	0,285061	0,873	0,57	0,000209	10	WNT5B
COMMD41	1,11E-08	0,35295	0,982	0,915	0,000213	10	COMMD4
CCT6A2	1,13E-08	0,390235	0,964	0,923	0,000217	10	CCT6A
MT-ATP61	1,16E-08	0,357736	1	1	0,000221	10	MT-ATP6
MT-ND61	1,22E-08	0,41211	0,964	0,881	0,000233	10	MT-ND6
CDC372	1,24E-08	0,397277	0,964	0,968	0,000237	10	CDC37
PRDX3	1,26E-08	0,341202	0,982	0,924	0,00024	10	PRDX3
RALA	1,30E-08	0,327402	0,945	0,851	0,000248	10	RALA
HNRNPH1	1,31E-08	0,381339	0,945	0,845	0,000251	10	HNRNPH1
YWHAB2	1,36E-08	0,298681	0,982	0,995	0,000261	10	YWHAB
ANXA23	1,39E-08	0,6118	1	1	0,000266	10	ANXA2
RPP30	1,51E-08	0,286499	0,873	0,661	0,000289	10	RPP30
EIF5A2	1,54E-08	0,31226	0,873	0,614	0,000294	10	EIF5A2
TAGLN21	1,59E-08	0,376367	1	0,989	0,000304	10	TAGLN2
COA41	1,59E-08	0,322895	0,964	0,9	0,000305	10	COA4
DYNLL11	1,60E-08	0,407279	1	0,998	0,000305	10	DYNLL1
IMP3	1,66E-08	0,339446	0,964	0,805	0,000317	10	IMP3
POLR2E1	1,78E-08	0,302032	1	0,974	0,00034	10	POLR2E
UBA2	1,89E-08	0,273436	0,927	0,703	0,000362	10	UBA2
VASP1	1,99E-08	0,322559	0,982	0,894	0,000381	10	VASP
FKBP21	2,03E-08	0,312734	1	0,986	0,000388	10	FKBP2
PSME22	2,16E-08	0,472707	0,964	0,953	0,000412	10	PSME2
KIFC3	2,17E-08	0,316592	0,964	0,722	0,000416	10	KIFC3
KHDRBS1	2,30E-08	0,320407	1	0,959	0,00044	10	KHDRBS1
RIF1	2,32E-08	0,255009	0,891	0,573	0,000444	10	RIF1
FBL1	2,43E-08	0,331799	0,964	0,93	0,000464	10	FBL
UQCC3	2,50E-08	0,323085	0,927	0,842	0,000478	10	UQCC3
PLEKHJ1	2,58E-08	0,264176	0,982	0,835	0,000493	10	PLEKHJ1
AUP1	2,59E-08	0,270992	0,982	0,892	0,000495	10	AUP1
SPHK1	2,64E-08	0,298968	0,709	0,4	0,000504	10	SPHK1
MED10	2,65E-08	0,317796	0,945	0,815	0,000507	10	MED10
COMMD7	2,68E-08	0,303097	0,982	0,937	0,000512	10	COMMD7
PMVK	2,74E-08	0,270161	0,964	0,827	0,000523	10	PMVK
TXNDC12	2,92E-08	0,290161	0,945	0,744	0,000558	10	TXNDC12
SZRD1	2,93E-08	0,262493	0,964	0,835	0,000559	10	SZRD1
MZT2B1	3,15E-08	0,291143	1	0,996	0,000602	10	MZT2B

PRMT12	3,18E-08	0,426534	0,982	0,887	0,000607	10	PRMT1
CCT22	3,47E-08	0,47248	1	0,903	0,000663	10	CCT2
MRPL37	3,48E-08	0,305868	0,945	0,876	0,000665	10	MRPL37
PAK2	3,60E-08	0,291298	0,945	0,862	0,000688	10	PAK2
MRPL41	3,61E-08	0,302256	1	0,991	0,00069	10	MRPL41
MAP2K31	3,74E-08	0,289954	0,873	0,641	0,000715	10	MAP2K3
SMARCB1	3,75E-08	0,292977	0,945	0,731	0,000716	10	SMARCB1
HSP90B12	3,76E-08	0,500792	1	0,966	0,000719	10	HSP90B1
YWHAZ	3,80E-08	0,274222	1	0,991	0,000727	10	YWHAZ
PXMP2	3,94E-08	0,255894	0,891	0,656	0,000753	10	PXMP2
NOC2L	4,06E-08	0,298348	0,927	0,696	0,000777	10	NOC2L
PRRC2C	4,15E-08	0,317183	1	0,971	0,000793	10	PRRC2C
FKBP1A2	4,68E-08	0,288529	1	0,996	0,000894	10	FKBP1A
TXNL4A1	4,78E-08	0,316152	0,982	0,95	0,000914	10	TXNL4A
VMA21	4,84E-08	0,256932	0,964	0,792	0,000926	10	VMA21
NOP10	4,92E-08	0,278629	1	0,995	0,00094	10	NOP10
UBE2V2	5,17E-08	0,33755	0,982	0,925	0,000988	10	UBE2V2
SF3B4	5,29E-08	0,295042	0,909	0,659	0,001011	10	SF3B4
LRRFIP2	5,31E-08	0,354313	0,945	0,843	0,001015	10	LRRFIP2
PDLIM5	5,45E-08	0,273603	0,891	0,68	0,001042	10	PDLIM5
ZCCHC17	5,45E-08	0,270511	0,982	0,88	0,001042	10	ZCCHC17
PDXK	5,56E-08	0,298203	0,945	0,892	0,001064	10	PDXK
POP5	5,69E-08	0,31632	0,818	0,604	0,001088	10	POP5
NES	5,77E-08	0,367217	0,745	0,473	0,001104	10	NES
MRPL361	5,86E-08	0,280921	1	0,95	0,00112	10	MRPL36
NUDCD2	6,23E-08	0,286815	0,927	0,733	0,001191	10	NUDCD2
HNRNPK2	6,24E-08	0,409414	1	0,995	0,001194	10	HNRNPK
RANGAP1	6,59E-08	0,344294	0,818	0,633	0,00126	10	RANGAP1
SSNA1	6,76E-08	0,325242	0,964	0,961	0,001293	10	SSNA1
HNRNPC1	6,87E-08	0,360715	1	0,985	0,001314	10	HNRNPC
MAGOH	7,26E-08	0,315958	0,909	0,696	0,001388	10	MAGOH
HNRNPF1	7,66E-08	0,380571	1	0,955	0,001465	10	HNRNPF
PNN	8,07E-08	0,344061	0,909	0,776	0,001543	10	PNN
PPP4C1	8,08E-08	0,31912	1	0,968	0,001546	10	PPP4C
CD591	8,21E-08	0,320002	1	0,997	0,00157	10	CD59
PTRF1	8,47E-08	0,278406	1	1	0,00162	10	PTRF
S100A113	8,69E-08	0,299803	1	1	0,001661	10	S100A11
TM4SF11	8,74E-08	0,680054	0,673	0,41	0,001672	10	TM4SF1
NDUFAB11	9,00E-08	0,300768	0,964	0,935	0,001721	10	NDUFAB1
PPP1CC	9,05E-08	0,256798	0,982	0,926	0,001731	10	PPP1CC
BDKRB11	9,32E-08	0,336892	0,873	0,666	0,001782	10	BDKRB1
MRPS161	9,76E-08	0,317806	1	0,957	0,001867	10	MRPS16
PTMA3	1,09E-07	0,384627	1	1	0,002091	10	PTMA
PTX3	1,11E-07	0,499515	0,564	0,258	0,002113	10	PTX3
MCFD2	1,17E-07	0,302024	1	0,927	0,00224	10	MCFD2
RBPJ	1,18E-07	0,289213	1	0,921	0,002257	10	RBPJ
VIMP1	1,18E-07	0,32716	1	0,943	0,002259	10	VIMP
FAM107B	1,21E-07	0,297429	0,873	0,642	0,002317	10	FAM107B
CDC42EP1:	1,23E-07	0,3225	0,945	0,854	0,00236	10	CDC42EP1
LINC00116	1,24E-07	0,391636	0,855	0,724	0,002368	10	LINC00116

MRPL221	1,24E-07	0,298869	0,982	0,875	0,002374	10	MRPL22
EMC8	1,32E-07	0,27035	0,873	0,743	0,002523	10	EMC8
RFC1	1,34E-07	0,329203	0,891	0,786	0,002563	10	RFC1
RAC12	1,34E-07	0,286934	1	0,999	0,002565	10	RAC1
KATNBL1	1,36E-07	0,271724	0,873	0,718	0,002592	10	KATNBL1
XRCC62	1,36E-07	0,391248	1	0,932	0,002609	10	XRCC6
HGS	1,39E-07	0,307576	0,927	0,663	0,002652	10	HGS
ABCF1	1,47E-07	0,273514	0,964	0,848	0,002815	10	ABCF1
LIMA1	1,48E-07	0,338304	1	0,988	0,002822	10	LIMA1
EIF4H	1,53E-07	0,265256	1	0,957	0,002919	10	EIF4H
KMT5A	1,55E-07	0,268381	0,782	0,502	0,002966	10	KMT5A
ACTR31	1,61E-07	0,333404	0,982	0,982	0,003074	10	ACTR3
GSTO12	1,61E-07	0,386829	1	0,994	0,003076	10	GSTO1
AK2	1,61E-07	0,251768	0,982	0,813	0,003079	10	AK2
TOR1A	1,61E-07	0,277338	0,927	0,64	0,003083	10	TOR1A
SUMO2	1,71E-07	0,282964	1	0,998	0,003276	10	SUMO2
MRPS71	1,84E-07	0,305368	0,964	0,873	0,003525	10	MRPS7
PSMB32	1,94E-07	0,372519	1	0,977	0,003711	10	PSMB3
RUVBL11	1,95E-07	0,255982	0,782	0,51	0,003727	10	RUVBL1
TRAPPC41	1,99E-07	0,329458	0,982	0,815	0,003799	10	TRAPPC4
KHSRP	2,05E-07	0,269407	0,891	0,658	0,003914	10	KHSRP
BZW12	2,09E-07	0,393934	0,945	0,966	0,004001	10	BZW1
RABL6	2,13E-07	0,253136	1	0,786	0,00407	10	RABL6
PSMD71	2,14E-07	0,302849	0,982	0,929	0,0041	10	PSMD7
PARK72	2,21E-07	0,324535	1	0,999	0,004223	10	PARK7
GTPBP4	2,25E-07	0,266237	0,855	0,593	0,004308	10	GTPBP4
NDUFB31	2,32E-07	0,304767	0,982	0,976	0,004432	10	NDUFB3
YDJC	2,37E-07	0,265345	0,927	0,641	0,004528	10	YDJC
TOMM5	2,40E-07	0,298389	0,927	0,772	0,004596	10	TOMM5
ATP1B31	2,68E-07	0,319797	0,982	0,956	0,005128	10	ATP1B3
DNAJC81	2,86E-07	0,270562	1	0,957	0,005466	10	DNAJC8
PSMA73	2,89E-07	0,373014	1	0,999	0,005521	10	PSMA7
EIF4G2	2,99E-07	0,284888	1	0,998	0,005721	10	EIF4G2
UBE2D2	3,30E-07	0,261368	0,982	0,966	0,006319	10	UBE2D2
SMIM15	3,65E-07	0,26001	0,964	0,828	0,006982	10	SMIM15
SRP19	3,66E-07	0,281712	0,964	0,853	0,007003	10	SRP19
PSMD15	3,70E-07	0,293659	1	0,843	0,007066	10	PSMD1
WDR11	3,92E-07	0,29737	1	0,989	0,007501	10	WDR1
HIST1H1C	3,97E-07	0,297216	0,727	0,456	0,007584	10	HIST1H1C
PUF601	4,02E-07	0,306707	0,945	0,808	0,007688	10	PUF60
LMNA3	4,09E-07	0,502954	1	0,998	0,007828	10	LMNA
ARPC4	4,13E-07	0,266643	1	0,95	0,007905	10	ARPC4
DAD11	4,14E-07	0,314029	0,982	0,992	0,007911	10	DAD1
BDP1	4,33E-07	0,269793	0,927	0,731	0,008275	10	BDP1
ZNF511	4,34E-07	0,27161	0,909	0,771	0,008293	10	ZNF511
PNRC2	4,34E-07	0,290402	0,927	0,756	0,008308	10	PNRC2
TMEM167A	5,16E-07	0,292357	1	0,961	0,00987	10	TMEM167A
WBSCR222	5,26E-07	0,278017	0,945	0,817	0,010063	10	WBSCR22
F32	5,55E-07	0,609939	0,945	0,767	0,010609	10	F3
POLR2J	5,85E-07	0,271452	0,982	0,981	0,011184	10	POLR2J

CAPN22	5,92E-07	0,298504	1	0,984	0,011329	10	CAPN2
ELAVL1	5,95E-07	0,253118	0,927	0,778	0,011369	10	ELAVL1
SPCS21	5,95E-07	0,324097	0,982	0,954	0,011375	10	SPCS2
EIF4G1	6,41E-07	0,300922	0,982	0,882	0,012258	10	EIF4G1
GPRC5A2	6,89E-07	0,442479	0,982	0,86	0,013169	10	GPRC5A
SEC61G2	6,98E-07	0,265102	1	0,998	0,013343	10	SEC61G
C17orf892	6,99E-07	0,322017	1	0,986	0,013363	10	C17orf89
ATG3	7,12E-07	0,261856	0,964	0,846	0,013614	10	ATG3
MAT2A	7,32E-07	0,332951	0,982	0,902	0,01399	10	MAT2A
UBE2J21	7,68E-07	0,295162	0,909	0,833	0,01468	10	UBE2J2
TMEM237	7,89E-07	0,252281	0,782	0,641	0,015087	10	TMEM237
LSM8	8,17E-07	0,316826	0,982	0,964	0,015627	10	LSM8
BAZ1B	8,51E-07	0,267208	0,964	0,835	0,016266	10	BAZ1B
CAPZA1	9,03E-07	0,295164	1	0,959	0,017265	10	CAPZA1
PSMB22	9,53E-07	0,329141	1	0,974	0,018232	10	PSMB2
ATP6V0B	9,73E-07	0,253998	0,982	0,968	0,018605	10	ATP6V0B
ARL16	9,98E-07	0,250178	0,982	0,858	0,019089	10	ARL16
UBE2K	1,02E-06	0,285674	0,891	0,88	0,01944	10	UBE2K
KXD1	1,05E-06	0,313249	0,982	0,92	0,020095	10	KXD1
TOP12	1,06E-06	0,379991	0,964	0,821	0,020199	10	TOP1
VAPA	1,08E-06	0,278484	1	0,98	0,020689	10	VAPA
EMC41	1,18E-06	0,334019	0,964	0,934	0,022495	10	EMC4
MRPL33	1,19E-06	0,284902	1	0,977	0,022668	10	MRPL33
PLIN32	1,20E-06	0,364352	0,982	0,928	0,022856	10	PLIN3
POLR2L1	1,23E-06	0,268818	1	0,999	0,023517	10	POLR2L
ANXA14	1,28E-06	0,466584	1	0,992	0,024422	10	ANXA1
GNG5	1,28E-06	0,276257	1	0,996	0,02447	10	GNG5
POLE41	1,32E-06	0,306138	1	0,975	0,025316	10	POLE4
GSPT12	1,33E-06	0,313332	0,927	0,863	0,025432	10	GSPT1
ASNSD1	1,37E-06	0,264058	0,909	0,715	0,026179	10	ASNSD1
TSPAN5	1,49E-06	0,304385	0,964	0,826	0,028439	10	TSPAN5
ARGLU1	1,49E-06	0,291796	0,964	0,919	0,028542	10	ARGLU1
MEST2	1,64E-06	0,49957	0,945	0,83	0,031375	10	MEST
PPP1R15A	1,76E-06	0,284817	0,982	0,856	0,033634	10	PPP1R15A
PSMC32	1,84E-06	0,361646	0,982	0,924	0,035258	10	PSMC3
MRPS26	2,10E-06	0,271327	0,909	0,791	0,040201	10	MRPS26
MSN	2,38E-06	0,259127	1	0,912	0,045444	10	MSN
TNFRSF11E	2,44E-06	0,552753	0,982	0,901	0,046603	10	TNFRSF11B
SURF41	2,56E-06	0,259174	0,982	0,93	0,048922	10	SURF4
GADD45A1	2,65E-06	0,40695	0,964	0,815	0,050615	10	GADD45A
SFRP12	2,68E-06	0,52567	0,782	0,665	0,051321	10	SFRP1
NOL72	2,73E-06	0,273895	0,982	0,906	0,052193	10	NOL7
UBE2L32	2,79E-06	0,316738	1	0,989	0,053347	10	UBE2L3
MT-ND51	2,96E-06	0,27752	1	0,998	0,056555	10	MT-ND5
EPS8	2,98E-06	0,258279	0,964	0,835	0,057002	10	EPS8
NDUFC22	3,12E-06	0,294217	1	0,985	0,059609	10	NDUFC2
SSSCA1	3,13E-06	0,257305	0,927	0,827	0,059824	10	SSSCA1
EIF4EBP11	3,23E-06	0,436216	1	0,972	0,061783	10	EIF4EBP1
NFIC1	3,30E-06	0,289447	0,982	0,976	0,063026	10	NFIC
TXNDC171	3,65E-06	0,279871	1	0,968	0,06976	10	TXNDC17

CNIH41	3,72E-06	0,263601	0,945	0,917	0,071147	10	CNIH4
THOC7	3,77E-06	0,251926	1	0,932	0,072162	10	THOC7
ERGIC21	3,93E-06	0,273457	0,945	0,91	0,075231	10	ERGIC2
RUVBL21	4,03E-06	0,281018	0,836	0,671	0,076982	10	RUVBL2
NDUFC11	4,10E-06	0,259787	0,964	0,966	0,07837	10	NDUFC1
RRBP11	4,47E-06	0,307695	1	0,989	0,085441	10	RRBP1
YWHAQ2	4,79E-06	0,319194	1	0,993	0,091653	10	YWHAQ
RNF5	5,18E-06	0,277748	0,818	0,646	0,09911	10	RNF5
ZDHHC121	5,48E-06	0,335237	0,982	0,94	0,104734	10	ZDHHC12
HNRNPA0	5,58E-06	0,27003	0,982	0,98	0,106711	10	HNRNPA0
KDELR22	5,60E-06	0,255332	1	0,995	0,107073	10	KDELR2
IER3IP1	6,04E-06	0,254875	0,982	0,927	0,115405	10	IER3IP1
MRPL31	6,25E-06	0,275587	0,945	0,851	0,119453	10	MRPL3
TIMM8B1	6,37E-06	0,27011	0,982	0,977	0,121842	10	TIMM8B
PSMB63	6,63E-06	0,28508	1	0,982	0,126737	10	PSMB6
C7orf50	6,91E-06	0,262437	1	0,955	0,132221	10	C7orf50
COPRS	7,05E-06	0,287025	0,982	0,945	0,134731	10	COPRS
CNN31	7,29E-06	0,284664	1	0,994	0,139338	10	CNN3
MRPL401	8,49E-06	0,270679	0,982	0,897	0,162389	10	MRPL40
TMSB4X1	8,76E-06	0,276317	1	1	0,167486	10	TMSB4X
MRPS10	8,94E-06	0,268735	0,964	0,844	0,170856	10	MRPS10
PSMD141	9,15E-06	0,268176	0,945	0,843	0,174914	10	PSMD14
ISOC21	9,96E-06	0,269199	0,964	0,924	0,190516	10	ISOC2
EIF4A31	9,99E-06	0,266247	0,836	0,674	0,191055	10	EIF4A3
PEA15	1,03E-05	0,318986	0,964	0,905	0,196378	10	PEA15
TACC1	1,10E-05	0,26188	0,927	0,846	0,211031	10	TACC1
TRIOBP	1,15E-05	0,269053	1	0,95	0,220627	10	TRIOBP
NANS3	1,17E-05	0,309874	0,927	0,849	0,223185	10	NANS
CAP11	1,27E-05	0,287068	1	0,992	0,242532	10	CAP1
PLAT2	1,33E-05	0,578568	0,964	0,834	0,25399	10	PLAT
SSBP12	1,40E-05	0,254482	0,982	0,985	0,266935	10	SSBP1
ANAPC151	1,45E-05	0,255278	0,909	0,893	0,276536	10	ANAPC15
KRT184	1,79E-05	0,64102	0,855	0,778	0,341894	10	KRT18
AAK1	1,80E-05	0,251535	0,964	0,878	0,344808	10	AAK1
CDC25B	1,89E-05	0,31435	0,855	0,689	0,360628	10	CDC25B
NDUFB61	1,96E-05	0,252	0,982	0,928	0,375681	10	NDUFB6
PSMD3	2,00E-05	0,251503	0,873	0,793	0,381964	10	PSMD3
MRPS18C1	2,21E-05	0,251104	0,964	0,883	0,423309	10	MRPS18C
PCMT11	2,23E-05	0,275554	0,945	0,839	0,427086	10	PCMT1
PTS1	2,26E-05	0,278687	0,964	0,903	0,431676	10	PTS
SSR31	2,35E-05	0,273914	1	0,993	0,449427	10	SSR3
LMO73	2,50E-05	0,289366	1	0,948	0,477922	10	LMO7
EIF4A12	2,51E-05	0,488298	0,982	0,975	0,479758	10	EIF4A1
PFKP1	2,78E-05	0,269487	0,964	0,789	0,530685	10	PFKP
NDUFA122	2,84E-05	0,271538	1	0,926	0,542471	10	NDUFA12
GLRX31	2,86E-05	0,260615	0,945	0,9	0,546363	10	GLRX3
CCL22	2,92E-05	0,673977	0,927	0,777	0,559021	10	CCL2
MT-ND21	2,96E-05	0,29183	1	1	0,566447	10	MT-ND2
SNRPD3	3,11E-05	0,280978	0,964	0,937	0,594678	10	SNRPD3
THRAP32	3,16E-05	0,28245	0,982	0,915	0,603478	10	THRAP3

DNAJB111	4,09E-05	0,264006	0,873	0,765	0,782353	10	DNAJB11
DHCR24	4,16E-05	0,260502	0,927	0,797	0,795814	10	DHCR24
SNRPC1	4,19E-05	0,281848	0,982	0,937	0,800959	10	SNRPC
ABL2	4,67E-05	0,25051	0,855	0,661	0,893626	10	ABL2
MIR4435-2	5,07E-05	0,275408	1	0,954	0,969973	10	MIR4435-2HG
TCP12	5,23E-05	0,324826	0,982	0,935	1	10	TCP1
MPC22	5,27E-05	0,303673	0,982	0,988	1	10	MPC2
CNN1	5,92E-05	0,269431	0,909	0,699	1	10	CNN1
SLC25A51	5,97E-05	0,328585	1	0,988	1	10	SLC25A5
MGAT2	6,11E-05	0,286301	0,873	0,712	1	10	MGAT2
NIFK	6,15E-05	0,265501	0,855	0,731	1	10	NIFK
TSR31	7,70E-05	0,263945	0,927	0,878	1	10	TSR3
HDAC21	7,87E-05	0,283887	0,982	0,842	1	10	HDAC2
CDC421	7,94E-05	0,270095	0,982	0,986	1	10	CDC42
SMURF22	8,14E-05	0,294326	0,945	0,837	1	10	SMURF2
HES4	8,58E-05	0,354477	0,855	0,695	1	10	HES4
BCLAF11	0,000104	0,26099	0,982	0,873	1	10	BCLAF1
PRSS232	0,000108	0,257528	1	0,873	1	10	PRSS23
BAX1	0,000112	0,251118	1	0,988	1	10	BAX
MT1X1	0,000115	0,313303	0,927	0,789	1	10	MT1X
CCT72	0,00012	0,275011	0,982	0,919	1	10	CCT7
ID31	0,000122	0,300856	1	0,934	1	10	ID3
RGCC1	0,000141	0,267862	0,527	0,32	1	10	RGCC
AC013461.	0,000151	0,250791	1	0,922	1	10	AC013461.1
PSMD83	0,000158	0,294913	1	0,994	1	10	PSMD8
MESDC22	0,000163	0,251853	0,945	0,837	1	10	MESDC2
CD472	0,00018	0,282119	0,982	0,931	1	10	CD47
PIF1	0,000186	0,311597	0,436	0,257	1	10	PIF1
S100A103	0,000191	0,252541	1	0,997	1	10	S100A10
HSPA91	0,000227	0,271302	0,964	0,885	1	10	HSPA9
ACTN11	0,000544	0,27462	1	0,997	1	10	ACTN1
LINC00152	0,000582	0,267949	0,982	0,987	1	10	LINC00152
CLEC11A1	0,000619	0,250139	0,927	0,802	1	10	CLEC11A
CTSC2	0,000653	0,29332	1	0,999	1	10	CTSC
PRDX13	0,00098	0,293188	1	1	1	10	PRDX1
CYR612	0,002273	0,513538	1	0,966	1	10	CYR61
CCDC85B1	0,003243	0,263243	1	0,995	1	10	CCDC85B
HSPA53	0,004081	0,283797	0,982	0,976	1	10	HSPA5
S100A114	1,41E-25	1,596777	1	1	2,69E-21	11	S100A11
SKP13	6,92E-25	1,282359	1	0,999	1,32E-20	11	SKP1
LGALS11	3,51E-24	0,820806	1	1	6,71E-20	11	LGALS1
PPIA1	7,85E-23	0,981356	1	1	1,50E-18	11	PPIA
ATP5J21	1,18E-21	1,009818	1	0,996	2,27E-17	11	ATP5J2
ACTB2	2,18E-21	1,544836	1	1	4,16E-17	11	ACTB
TPM21	3,19E-21	1,269675	1	1	6,11E-17	11	TPM2
TMSB101	3,15E-20	0,699232	1	1	6,03E-16	11	TMSB10
SNRPD2	3,71E-20	0,98159	1	0,998	7,10E-16	11	SNRPD2
GSTP12	4,34E-20	1,0306	1	0,999	8,31E-16	11	GSTP1
UBB2	8,24E-20	1,21409	1	0,999	1,57E-15	11	UBB
USMG52	9,35E-20	0,987897	1	0,997	1,79E-15	11	USMG5

NDUFS5	1,04E-19	0,864108	1	0,999	1,98E-15	11 NDUFS5
SEC61G3	2,39E-19	0,917632	1	0,998	4,57E-15	11 SEC61G
MYL12A1	2,81E-19	1,340161	1	1	5,36E-15	11 MYL12A
ATP5H1	6,43E-19	0,977898	1	0,995	1,23E-14	11 ATP5H
PRDX14	8,33E-19	1,134002	1	1	1,59E-14	11 PRDX1
C12orf753	1,06E-18	1,563934	1	0,996	2,02E-14	11 C12orf75
PFN13	1,09E-18	0,892276	1	1	2,08E-14	11 PFN1
GSTO13	1,21E-18	1,318839	1	0,994	2,32E-14	11 GSTO1
S100A6	1,52E-18	0,865662	1	1	2,91E-14	11 S100A6
COTL13	1,68E-18	1,31454	1	0,979	3,21E-14	11 COTL1
MINOS11	1,69E-18	0,935128	1	0,991	3,23E-14	11 MINOS1
NEDD83	2,71E-18	0,721395	1	0,999	5,18E-14	11 NEDD8
PBK1	3,15E-18	0,493727	0,333	0,044	6,03E-14	11 PBK
POLR2L2	3,37E-18	0,843353	1	0,999	6,44E-14	11 POLR2L
UQCRQ	3,98E-18	0,74328	1	1	7,61E-14	11 UQCRQ
VDAC12	5,66E-18	1,059232	1	0,988	1,08E-13	11 VDAC1
ANXA24	6,07E-18	1,422214	1	1	1,16E-13	11 ANXA2
COX7A22	8,43E-18	0,781075	1	0,999	1,61E-13	11 COX7A2
NME14	8,93E-18	1,228426	0,974	0,939	1,71E-13	11 NME1
TPI14	1,76E-17	1,157177	1	0,996	3,37E-13	11 TPI1
ANXA51	1,87E-17	1,000462	1	1	3,58E-13	11 ANXA5
SH3BGRL3:	2,05E-17	1,180993	1	0,997	3,92E-13	11 SH3BGRL3
ALDOA3	2,35E-17	0,889218	1	1	4,50E-13	11 ALDOA
COX8A2	2,43E-17	0,85533	1	0,997	4,65E-13	11 COX8A
NDUFA1	2,82E-17	0,765652	0,974	0,992	5,39E-13	11 NDUFA1
FKBP1A3	3,04E-17	1,131267	1	0,996	5,81E-13	11 FKBP1A
TMSB4X2	3,45E-17	0,794122	1	1	6,60E-13	11 TMSB4X
COX7B	3,99E-17	0,805994	1	0,996	7,63E-13	11 COX7B
S100A104	4,03E-17	0,983914	1	0,997	7,70E-13	11 S100A10
RHOC1	7,43E-17	0,991226	1	0,996	1,42E-12	11 RHOC
COX6A1	8,05E-17	0,882355	1	0,999	1,54E-12	11 COX6A1
MT2A3	8,55E-17	2,505345	1	0,999	1,64E-12	11 MT2A
MYL12B1	2,22E-16	0,944833	1	0,999	4,24E-12	11 MYL12B
RPL35	2,26E-16	0,526436	1	1	4,32E-12	11 RPL35
HINT1	2,59E-16	0,604565	1	1	4,95E-12	11 HINT1
TBCA	3,37E-16	0,633786	1	0,996	6,45E-12	11 TBCA
CFL13	3,91E-16	0,847204	1	1	7,47E-12	11 CFL1
ARF41	1,07E-15	1,019409	1	0,993	2,04E-11	11 ARF4
GAPDH2	1,10E-15	0,594587	1	1	2,11E-11	11 GAPDH
TXN1	1,64E-15	0,826072	1	1	3,14E-11	11 TXN
CALM13	2,18E-15	0,953177	1	0,991	4,17E-11	11 CALM1
PGAM13	2,57E-15	1,020101	0,974	0,995	4,91E-11	11 PGAM1
TPM32	3,28E-15	1,130062	1	0,982	6,27E-11	11 TPM3
ENO14	6,11E-15	1,146368	1	0,998	1,17E-10	11 ENO1
MYL92	8,36E-15	1,120882	1	1	1,60E-10	11 MYL9
YWHAQ3	1,19E-14	0,787998	0,974	0,993	2,28E-10	11 YWHAQ
UBL51	1,26E-14	0,696147	1	0,999	2,41E-10	11 UBL5
YBX12	1,42E-14	0,59822	1	1	2,71E-10	11 YBX1
ANXA15	1,62E-14	1,402604	1	0,992	3,11E-10	11 ANXA1
NDUFB32	1,72E-14	0,715755	1	0,976	3,28E-10	11 NDUFB3

RANBP12	1,80E-14	1,293225	0,974	0,946	3,44E-10	11	RANBP1
ARPC41	2,05E-14	0,84643	0,949	0,951	3,91E-10	11	ARPC4
SRP92	2,19E-14	0,804169	0,949	0,978	4,18E-10	11	SRP9
MIF	2,34E-14	0,730892	1	0,995	4,47E-10	11	MIF
PFDN51	2,46E-14	0,523392	1	1	4,71E-10	11	PFDN5
ATP5G13	2,76E-14	0,872713	0,949	0,971	5,27E-10	11	ATP5G1
RAN3	2,86E-14	1,070887	1	0,996	5,47E-10	11	RAN
TSPO	3,00E-14	0,732696	1	1	5,73E-10	11	TSPO
PKM4	3,38E-14	1,040913	1	0,999	6,46E-10	11	PKM
PGK12	3,83E-14	0,799274	0,974	0,975	7,33E-10	11	PGK1
DBI2	4,11E-14	0,71709	1	0,999	7,87E-10	11	DBI
CYCS3	4,26E-14	1,130836	0,974	0,967	8,14E-10	11	CYCS
COX6B1	4,47E-14	0,564325	1	0,999	8,55E-10	11	COX6B1
HSPB12	5,05E-14	0,830671	1	1	9,66E-10	11	HSPB1
PHLDA23	5,62E-14	1,34119	0,949	0,967	1,08E-09	11	PHLDA2
TRIP131	6,19E-14	0,375861	0,308	0,05	1,18E-09	11	TRIP13
UBA52	6,32E-14	0,421304	1	1	1,21E-09	11	UBA52
PRDX23	6,97E-14	0,807973	0,923	0,986	1,33E-09	11	PRDX2
BIRC52	7,94E-14	1,331433	0,513	0,142	1,52E-09	11	BIRC5
CSRP11	9,63E-14	1,066366	1	0,985	1,84E-09	11	CSRP1
DYNLRB1	1,12E-13	0,697869	1	0,993	2,14E-09	11	DYNLRB1
SOD11	1,35E-13	0,88609	1	0,998	2,58E-09	11	SOD1
PARK73	1,52E-13	0,72286	1	0,999	2,91E-09	11	PARK7
PHPT1	2,10E-13	0,66333	1	0,997	4,01E-09	11	PHPT1
LSM42	2,10E-13	0,88913	0,974	0,965	4,03E-09	11	LSM4
HSBP1	2,24E-13	0,760445	1	0,992	4,29E-09	11	HSBP1
ATPIF12	2,74E-13	0,867172	0,949	0,991	5,25E-09	11	ATPIF1
CAPZB1	2,89E-13	0,766639	1	0,993	5,53E-09	11	CAPZB
RPS27L1	3,05E-13	0,617519	1	1	5,83E-09	11	RPS27L
BANF12	3,22E-13	0,727985	0,974	0,966	6,16E-09	11	BANF1
RRM22	4,08E-13	0,832843	0,41	0,093	7,81E-09	11	RRM2
ACTG1	4,58E-13	0,694617	1	1	8,76E-09	11	ACTG1
CMPK1	5,45E-13	0,711124	0,974	0,942	1,04E-08	11	CMPK1
UCHL12	5,50E-13	0,977161	1	0,999	1,05E-08	11	UCHL1
CLTB3	6,42E-13	0,727436	1	0,982	1,23E-08	11	CLTB
TAGLN3	6,94E-13	1,419523	1	1	1,33E-08	11	TAGLN
ATOX11	8,59E-13	0,579698	1	0,996	1,64E-08	11	ATOX1
RPS262	8,64E-13	0,550677	1	1	1,65E-08	11	RPS26
RHOA	9,75E-13	0,731719	1	0,997	1,86E-08	11	RHOA
MYL62	1,24E-12	0,682508	1	1	2,38E-08	11	MYL6
PSMA74	1,30E-12	0,847158	1	0,999	2,48E-08	11	PSMA7
SNRPC2	1,30E-12	0,783202	0,923	0,938	2,49E-08	11	SNRPC
HIGD1A	1,61E-12	0,775728	0,949	0,944	3,08E-08	11	HIGD1A
POMP3	1,63E-12	0,74515	1	1	3,12E-08	11	POMP
LSM32	1,68E-12	0,76417	1	0,98	3,21E-08	11	LSM3
MAP1LC3B	1,73E-12	0,821198	1	0,992	3,31E-08	11	MAP1LC3B
TK12	1,85E-12	0,95845	0,692	0,301	3,53E-08	11	TK1
NDUFB62	2,29E-12	0,732479	0,949	0,929	4,37E-08	11	NDUFB6
NDUFA6	2,30E-12	0,692088	1	0,974	4,41E-08	11	NDUFA6
CHCHD22	2,54E-12	0,454802	1	0,999	4,86E-08	11	CHCHD2

TCEB11	2,92E-12	0,86003	0,949	0,986	5,59E-08	11	TCEB1
VBP1	3,18E-12	0,688006	0,872	0,737	6,07E-08	11	VBP1
NDUFA11	3,93E-12	0,497977	1	0,998	7,52E-08	11	NDUFA11
AP2S12	4,31E-12	0,550087	1	1	8,24E-08	11	AP2S1
FAU	4,54E-12	0,374714	1	1	8,68E-08	11	FAU
NDUFS62	4,70E-12	0,628512	1	0,997	8,99E-08	11	NDUFS6
PSMB33	4,71E-12	0,980234	0,949	0,978	9,00E-08	11	PSMB3
LDHB1	5,38E-12	0,56105	1	1	1,03E-07	11	LDHB
NDUFB2	5,89E-12	0,812355	1	0,994	1,13E-07	11	NDUFB2
ARPC23	7,37E-12	0,663336	1	0,999	1,41E-07	11	ARPC2
UQCR103	7,55E-12	0,626819	0,974	0,996	1,44E-07	11	UQCR10
EIF5A4	7,70E-12	0,961263	0,897	0,899	1,47E-07	11	EIF5A
PSMD84	9,01E-12	0,83257	1	0,994	1,72E-07	11	PSMD8
COX6C	1,14E-11	0,627274	1	0,997	2,18E-07	11	COX6C
PCBD1	1,34E-11	0,594839	0,974	0,945	2,55E-07	11	PCBD1
TAGLN22	1,39E-11	0,787771	0,974	0,989	2,65E-07	11	TAGLN2
ATP5L	1,42E-11	0,44402	1	1	2,71E-07	11	ATP5L
PSMD23	1,84E-11	0,94444	0,897	0,893	3,51E-07	11	PSMD2
NDUFA41	2,10E-11	0,737312	1	0,999	4,01E-07	11	NDUFA4
RPL31	2,10E-11	0,483691	1	1	4,02E-07	11	RPL31
SSBP13	2,15E-11	0,659181	1	0,984	4,12E-07	11	SSBP1
TXNDC172	2,41E-11	0,768127	0,949	0,969	4,60E-07	11	TXNDC17
PDAP12	2,79E-11	0,697748	0,949	0,971	5,33E-07	11	PDAP1
UQCRH	3,23E-11	0,476843	1	0,998	6,17E-07	11	UQCRH
CCND15	3,34E-11	1,46177	0,949	0,967	6,39E-07	11	CCND1
DNAJC82	3,36E-11	0,723245	0,949	0,958	6,42E-07	11	DNAJC8
RNF181	3,73E-11	0,736053	0,974	0,976	7,13E-07	11	RNF181
ECHS11	3,81E-11	0,683713	0,974	0,94	7,29E-07	11	ECHS1
C14orf166[illegible]	3,86E-11	0,551812	1	0,996	7,39E-07	11	C14orf166
PSME23	3,95E-11	0,88855	0,949	0,953	7,55E-07	11	PSME2
DRAP12	4,13E-11	0,788611	1	0,997	7,90E-07	11	DRAP1
FKBP31	4,13E-11	0,804021	0,923	0,881	7,90E-07	11	FKBP3
ESD1	4,63E-11	0,658872	0,974	0,996	8,86E-07	11	ESD
GNG111	5,41E-11	0,815325	1	0,998	1,03E-06	11	GNG11
UBE2L33	5,59E-11	0,612332	1	0,989	1,07E-06	11	UBE2L3
ATP5B2	5,68E-11	0,750128	1	0,994	1,09E-06	11	ATP5B
AP2M13	6,08E-11	0,632921	1	0,998	1,16E-06	11	AP2M1
PFN21	6,64E-11	0,75849	0,974	0,974	1,27E-06	11	PFN2
CAPZA21	7,86E-11	0,668171	0,974	0,969	1,50E-06	11	CAPZA2
RPL41	8,95E-11	0,356842	1	1	1,71E-06	11	RPL41
NPM12	1,11E-10	0,717554	1	1	2,12E-06	11	NPM1
ROMO1	1,20E-10	0,710546	0,974	0,992	2,30E-06	11	ROMO1
ATP5E1	1,43E-10	0,493145	1	1	2,74E-06	11	ATP5E
RBX11	1,57E-10	0,634341	1	0,991	3,00E-06	11	RBX1
TRAPPC1	1,96E-10	0,68206	0,974	0,995	3,74E-06	11	TRAPPC1
UQCR11	2,51E-10	0,639322	1	0,998	4,80E-06	11	UQCR11
NDUFB82	2,67E-10	0,64856	0,974	0,987	5,11E-06	11	NDUFB8
RPS10	2,71E-10	0,395085	1	1	5,18E-06	11	RPS10
RPL24	2,83E-10	0,317307	1	1	5,41E-06	11	RPL24
PSMB64	4,46E-10	0,67192	1	0,982	8,52E-06	11	PSMB6

MOCS2	5,10E-10	0,671697	0,872	0,869	9,75E-06	11	MOCS2
STMN12	5,20E-10	1,285898	0,897	0,804	9,94E-06	11	STMN1
S100A13	5,57E-10	0,692085	1	0,996	1,07E-05	11	S100A13
GUK11	5,98E-10	0,506789	1	0,998	1,14E-05	11	GUK1
MRPL331	6,94E-10	0,640578	0,974	0,978	1,33E-05	11	MRPL33
HNRNPC2	9,98E-10	0,836591	0,974	0,985	1,91E-05	11	HNRNPC
EID12	1,08E-09	0,512831	1	0,999	2,06E-05	11	EID1
COX5B	1,15E-09	0,450441	1	0,999	2,20E-05	11	COX5B
CALM32	1,30E-09	0,68715	0,923	0,972	2,49E-05	11	CALM3
VAMP3	1,32E-09	0,66095	0,897	0,957	2,53E-05	11	VAMP3
RPA32	1,35E-09	0,641732	0,795	0,634	2,57E-05	11	RPA3
NAP1L11	1,45E-09	0,575738	1	0,999	2,78E-05	11	NAP1L1
SMS2	1,46E-09	0,96905	1	0,942	2,80E-05	11	SMS
COPS63	1,75E-09	0,713588	1	0,974	3,34E-05	11	COPS6
GLO1	1,90E-09	0,576231	0,923	0,915	3,62E-05	11	GLO1
ADIRF3	1,96E-09	0,884652	0,949	0,922	3,75E-05	11	ADIRF
HMGA14	2,05E-09	1,360556	0,949	0,969	3,91E-05	11	HMGA1
DCTN31	2,17E-09	0,778679	0,974	0,978	4,16E-05	11	DCTN3
MGST32	2,20E-09	0,582853	1	0,996	4,20E-05	11	MGST3
NDUFC12	2,35E-09	0,589439	0,949	0,966	4,49E-05	11	NDUFC1
IFITM22	2,47E-09	0,692966	1	0,996	4,72E-05	11	IFITM2
CFL2	2,81E-09	0,774787	0,974	0,975	5,37E-05	11	CFL2
PCNP	2,97E-09	0,585287	0,974	0,954	5,68E-05	11	PCNP
HSPD13	3,08E-09	0,950886	0,897	0,926	5,89E-05	11	HSPD1
TMA71	3,14E-09	0,441144	1	0,999	6,01E-05	11	TMA7
SNRPF2	3,50E-09	0,565728	0,974	0,982	6,70E-05	11	SNRPF
ACTG22	3,65E-09	0,878938	1	1	6,98E-05	11	ACTG2
NDUFA2	4,40E-09	0,530212	1	0,979	8,42E-05	11	NDUFA2
TCEB2	4,47E-09	0,487703	1	1	8,55E-05	11	TCEB2
PRDX62	4,49E-09	0,796382	1	0,997	8,59E-05	11	PRDX6
RPL27	4,82E-09	0,286397	1	1	9,22E-05	11	RPL27
HSP90AB12	5,04E-09	1,016718	1	0,999	9,63E-05	11	HSP90AB1
GLRX32	5,53E-09	0,742323	0,872	0,901	0,000106	11	GLRX3
ATP5J	5,99E-09	0,451149	1	0,996	0,000115	11	ATP5J
MEA11	6,39E-09	0,57363	0,897	0,919	0,000122	11	MEA1
CDC422	6,45E-09	0,809531	0,949	0,986	0,000123	11	CDC42
PSMB72	6,48E-09	0,446468	0,949	0,994	0,000124	11	PSMB7
NCL3	6,71E-09	0,707693	0,949	0,989	0,000128	11	NCL
GTF2H51	7,43E-09	0,610752	0,974	0,987	0,000142	11	GTF2H5
CLIC14	7,91E-09	0,582876	0,974	0,999	0,000151	11	CLIC1
SUB1	8,02E-09	0,466841	1	0,999	0,000153	11	SUB1
PSMA11	8,37E-09	0,598683	0,974	0,989	0,00016	11	PSMA1
ACOT7	8,54E-09	0,485339	0,846	0,751	0,000163	11	ACOT7
NDUFB41	8,88E-09	0,524955	1	0,997	0,00017	11	NDUFB4
TROAP2	9,29E-09	0,28593	0,333	0,087	0,000178	11	TROAP
HMGN22	9,30E-09	0,938376	0,974	0,994	0,000178	11	HMGN2
SLIRP2	1,00E-08	0,630004	1	0,988	0,000192	11	SLIRP
TBCB1	1,02E-08	0,530017	1	0,955	0,000195	11	TBCB
sep-72	1,26E-08	0,657815	1	0,995	0,00024	11	07-sep
LARP61	1,70E-08	0,621233	0,949	0,987	0,000324	11	LARP6

FAM96B	1,83E-08	0,53046	1	0,987	0,00035	11	FAM96B
PHB2	1,90E-08	0,573914	0,923	0,967	0,000364	11	PHB
PSMB52	2,06E-08	0,435352	1	0,994	0,000394	11	PSMB5
SHFM1	2,09E-08	0,478158	1	0,997	0,000399	11	SHFM1
SRP14	2,25E-08	0,57337	1	1	0,000431	11	SRP14
MDH23	2,92E-08	0,639784	1	0,986	0,000558	11	MDH2
ANAPC111	3,20E-08	0,461824	1	0,997	0,000611	11	ANAPC11
CDC202	3,25E-08	0,503591	0,308	0,079	0,000621	11	CDC20
CUTA1	3,74E-08	0,443046	1	0,999	0,000715	11	CUTA
SCP2	4,30E-08	0,670092	0,974	0,982	0,000821	11	SCP2
TALDO12	4,45E-08	0,544358	1	0,997	0,00085	11	TALDO1
COPE2	4,50E-08	0,589888	0,974	0,996	0,000861	11	COPE
ETFA1	4,61E-08	0,557966	0,923	0,911	0,000882	11	ETFA
CCT73	5,16E-08	0,694042	0,897	0,921	0,000986	11	CCT7
KIAA01013	5,75E-08	1,18728	0,667	0,419	0,0011	11	KIAA0101
RPL22	5,83E-08	0,315848	1	1	0,001114	11	RPL22
ENSA	6,51E-08	0,52326	0,949	0,904	0,001245	11	ENSA
NDUFA13	6,85E-08	0,594907	1	0,987	0,00131	11	NDUFA13
NUTF22	7,42E-08	0,596116	0,949	0,978	0,001419	11	NUTF2
EIF3I2	7,59E-08	0,561914	0,974	0,992	0,001452	11	EIF3I
DYNLL12	7,69E-08	0,585307	1	0,998	0,001471	11	DYNLL1
PEBP1	8,18E-08	0,5125	1	0,998	0,001565	11	PEBP1
DSTN	8,21E-08	0,606432	1	1	0,00157	11	DSTN
TOMM51	9,37E-08	0,415097	0,821	0,774	0,001792	11	TOMM5
MRPL522	1,06E-07	0,541435	0,872	0,973	0,002019	11	MRPL52
UBE2N2	1,07E-07	0,566282	0,923	0,926	0,002045	11	UBE2N
SCOC	1,09E-07	0,643659	0,821	0,852	0,002092	11	SCOC
RPL21	1,16E-07	0,315021	1	1	0,002209	11	RPL21
WDR83OS	1,23E-07	0,48165	0,974	0,992	0,002358	11	WDR83OS
COX203	1,66E-07	0,657386	0,897	0,945	0,003172	11	COX20
HSPE12	1,71E-07	0,681968	0,923	0,989	0,003277	11	HSPE1
ATP5C13	2,02E-07	0,496484	0,974	0,984	0,00386	11	ATP5C1
ATP5I	2,10E-07	0,4947	0,923	0,992	0,004011	11	ATP5I
RPL38	2,28E-07	0,319484	1	1	0,004369	11	RPL38
IFI27L2	2,32E-07	0,507337	1	0,993	0,004431	11	IFI27L2
CETN2	2,39E-07	0,531871	0,897	0,891	0,004563	11	CETN2
TCEAL41	2,41E-07	0,646636	0,974	0,988	0,004608	11	TCEAL4
AKR1B12	2,58E-07	0,473241	1	0,978	0,004936	11	AKR1B1
MDH11	2,61E-07	0,50196	0,897	0,922	0,004994	11	MDH1
ATP6V1E11	2,68E-07	0,645872	0,923	0,977	0,005117	11	ATP6V1E1
RPLP0	2,71E-07	0,293625	1	1	0,005189	11	RPLP0
RPS24	2,85E-07	0,337438	1	1	0,005453	11	RPS24
NDUFC23	2,94E-07	0,536684	0,923	0,986	0,005627	11	NDUFC2
PSMA41	3,04E-07	0,697847	1	0,989	0,005808	11	PSMA4
NOP101	3,07E-07	0,490831	1	0,995	0,00587	11	NOP10
NDUFAB12	3,13E-07	0,642654	0,872	0,936	0,005989	11	NDUFAB1
TRAPPC2L1	3,16E-07	0,520254	0,974	0,973	0,006045	11	TRAPPC2L
EIF63	3,23E-07	0,434205	0,949	0,965	0,006173	11	EIF6
RPL37A	3,29E-07	0,258683	1	1	0,006294	11	RPL37A
RPL391	3,30E-07	0,302811	1	1	0,006313	11	RPL39

MRPS21	3,37E-07	0,509702	0,949	0,994	0,006436	11	MRPS21
PSMC33	3,53E-07	0,578146	0,872	0,926	0,006749	11	PSMC3
VPS291	3,68E-07	0,516191	0,949	0,96	0,007031	11	VPS29
VDAC2	4,07E-07	0,455665	0,974	0,996	0,007778	11	VDAC2
UFM1	4,08E-07	0,534757	0,872	0,918	0,007793	11	UFM1
HNRNPA12	4,36E-07	0,729866	0,974	0,999	0,00833	11	HNRNPA1
RPL23A	4,40E-07	0,272411	1	1	0,008422	11	RPL23A
STX8	5,17E-07	0,525857	0,769	0,765	0,009877	11	STX8
DCXR	5,45E-07	0,596149	0,846	0,861	0,010417	11	DCXR
RBM3	6,03E-07	0,408835	1	0,999	0,011538	11	RBM3
CCT81	6,72E-07	0,52099	0,949	0,994	0,012857	11	CCT8
TRMT1121	6,76E-07	0,476437	0,949	0,994	0,012924	11	TRMT112
COX7C	6,84E-07	0,3467	1	0,999	0,013077	11	COX7C
SEC61B	6,97E-07	0,345074	1	0,998	0,013333	11	SEC61B
UQCRB1	7,41E-07	0,467893	1	1	0,01416	11	UQCRB
NDUFB1	7,53E-07	0,486748	0,949	0,992	0,014395	11	NDUFB1
REXO22	8,03E-07	0,457596	1	0,994	0,015358	11	REXO2
AP1S11	8,59E-07	0,439765	0,923	0,956	0,016418	11	AP1S1
SNU133	8,64E-07	0,559305	1	0,995	0,016529	11	SNU13
RPL36	8,71E-07	0,282804	1	1	0,016653	11	RPL36
C19orf70	8,72E-07	0,464253	1	0,988	0,016684	11	C19orf70
XRCC53	9,04E-07	0,524336	0,949	0,938	0,017284	11	XRCC5
RPS17	9,09E-07	0,258648	1	1	0,017376	11	RPS17
SUMO1	9,42E-07	0,385182	1	0,996	0,018011	11	SUMO1
SAP181	9,52E-07	0,488142	0,974	0,994	0,018205	11	SAP18
HSP90AA1:	9,73E-07	0,825737	1	0,999	0,018608	11	HSP90AA1
PAICS2	1,06E-06	0,534861	0,718	0,64	0,02029	11	PAICS
DAP	1,07E-06	0,581067	0,897	0,936	0,020407	11	DAP
IFITM31	1,08E-06	0,395362	1	1	0,020657	11	IFITM3
NAA38	1,10E-06	0,485829	0,923	0,971	0,020949	11	NAA38
CENPH1	1,18E-06	0,434912	0,462	0,221	0,022651	11	CENPH
UFD1L2	1,26E-06	0,583712	0,821	0,858	0,024063	11	UFD1L
PA2G43	1,36E-06	0,747567	0,897	0,929	0,026095	11	PA2G4
CACYBP3	1,46E-06	0,506535	0,846	0,814	0,027995	11	CACYBP
C1QBP2	1,70E-06	0,58063	0,872	0,964	0,032448	11	C1QBP
RPL14	1,94E-06	0,308224	1	1	0,037109	11	RPL14
CHMP51	2,01E-06	0,482702	0,923	0,948	0,038342	11	CHMP5
PRDX31	2,07E-06	0,609833	0,821	0,926	0,039572	11	PRDX3
XRCC63	2,26E-06	0,509448	0,897	0,934	0,043297	11	XRCC6
SNX6	2,43E-06	0,446144	0,897	0,938	0,046388	11	SNX6
SNX3	2,50E-06	0,41656	1	0,998	0,047747	11	SNX3
ANXA61	2,99E-06	0,499679	0,923	0,98	0,057244	11	ANXA6
RSU11	3,01E-06	0,460429	0,846	0,905	0,057541	11	RSU1
PDCD53	3,44E-06	0,54711	1	0,989	0,065787	11	PDCD5
POLR2E2	3,52E-06	0,426007	0,949	0,974	0,067218	11	POLR2E
GSTM31	4,24E-06	0,560246	0,974	0,965	0,08101	11	GSTM3
RPL22L14	4,50E-06	0,922889	0,897	0,974	0,086047	11	RPL22L1
RPSA1	4,75E-06	0,302399	1	1	0,090902	11	RPSA
RTFDC1	4,76E-06	0,621859	0,872	0,942	0,090976	11	RTFDC1
ADI11	4,90E-06	0,536272	0,897	0,931	0,093631	11	ADI1

RPL17	5,16E-06	0,341625	1	0,992	0,09867	11	RPL17
SELT	5,52E-06	0,525814	0,872	0,953	0,105606	11	SELT
ATP5G34	5,62E-06	0,486446	1	0,997	0,107399	11	ATP5G3
RPS211	5,85E-06	0,353132	1	1	0,111914	11	RPS21
YWHAE2	6,22E-06	0,396928	1	0,996	0,119003	11	YWHAE
SDHC1	6,24E-06	0,373065	0,949	0,985	0,119359	11	SDHC
DAD12	6,73E-06	0,532151	1	0,991	0,128719	11	DAD1
PRMT13	7,27E-06	0,490183	0,821	0,89	0,138975	11	PRMT1
MGST12	7,44E-06	0,53438	1	1	0,142331	11	MGST1
SF3B61	7,65E-06	0,459736	0,974	0,991	0,146224	11	SF3B6
EIF1AY	7,86E-06	0,473702	0,795	0,827	0,150253	11	EIF1AY
BLOC1S1	7,88E-06	0,384589	0,974	0,989	0,150668	11	BLOC1S1
UBE2A	7,89E-06	0,45503	0,923	0,914	0,15078	11	UBE2A
TPM41	8,03E-06	0,362206	1	1	0,153531	11	TPM4
DYNLT3	8,92E-06	0,617201	0,744	0,794	0,170629	11	DYNLT3
GTF3C62	1,00E-05	0,610232	0,846	0,944	0,191556	11	GTF3C6
ATP5O1	1,00E-05	0,39801	1	0,995	0,191757	11	ATP5O
NDUFB7	1,05E-05	0,334407	0,974	0,996	0,200203	11	NDUFB7
BZW13	1,06E-05	0,552249	0,897	0,966	0,201814	11	BZW1
DPY30	1,07E-05	0,542232	0,846	0,875	0,204299	11	DPY30
PSMC42	1,11E-05	0,493715	0,795	0,773	0,212659	11	PSMC4
NHP23	1,27E-05	0,511471	0,949	0,968	0,242928	11	NHP2
COX172	1,39E-05	0,593448	0,821	0,893	0,264977	11	COX17
COX5A	1,45E-05	0,438372	0,974	0,994	0,276565	11	COX5A
RPL26	1,58E-05	0,279024	1	1	0,302636	11	RPL26
ADK	1,61E-05	0,617165	0,667	0,61	0,307036	11	ADK
ILF23	1,96E-05	0,466225	0,846	0,862	0,374767	11	ILF2
TMEM167/	2,00E-05	0,466099	0,897	0,962	0,381901	11	TMEM167A
H2AFZ2	2,01E-05	1,407186	0,974	0,998	0,383681	11	H2AFZ
APRT	2,05E-05	0,424813	1	0,997	0,391296	11	APRT
UQCC22	2,25E-05	0,426666	0,897	0,951	0,429849	11	UQCC2
SRM3	2,29E-05	0,522277	0,949	0,968	0,437464	11	SRM
COX4I11	2,39E-05	0,323846	1	1	0,456129	11	COX4I1
PIN12	2,42E-05	0,426566	0,897	0,966	0,462659	11	PIN1
SKA22	2,53E-05	0,446004	0,795	0,846	0,482915	11	SKA2
SNRPE2	2,65E-05	0,470295	0,974	0,993	0,507418	11	SNRPE
CIB1	2,97E-05	0,393329	0,974	0,972	0,567009	11	CIB1
C14orf2	2,99E-05	0,319781	1	0,999	0,57101	11	C14orf2
CDC373	3,25E-05	0,679616	0,821	0,97	0,621729	11	CDC37
PSME11	3,27E-05	0,446929	0,974	0,987	0,624396	11	PSME1
TMEM230	3,34E-05	0,434748	1	0,985	0,638526	11	TMEM230
PRR131	3,65E-05	0,458226	0,974	0,979	0,697858	11	PRR13
SPA17	3,69E-05	0,352562	0,513	0,333	0,704885	11	SPA17
CLTA1	3,83E-05	0,354235	1	1	0,732864	11	CLTA
DEF8	3,97E-05	0,31134	0,897	0,927	0,759853	11	DEF8
MTCH1	4,04E-05	0,475065	0,897	0,978	0,771826	11	MTCH1
PTTG12	4,13E-05	1,537113	0,564	0,418	0,789802	11	PTTG1
PRKCDBP2	4,40E-05	0,454297	0,974	0,995	0,842297	11	PRKCDBP
CYB5B	4,45E-05	0,538219	0,821	0,9	0,851315	11	CYB5B
AK21	4,60E-05	0,332549	0,795	0,816	0,879323	11	AK2

NDUFAF31	4,75E-05	0,506097	0,949	0,981	0,908296	11	NDUFAF3
MCTS11	4,85E-05	0,521433	0,872	0,912	0,926924	11	MCTS1
ATP6V1F	4,92E-05	0,455103	1	0,997	0,941713	11	ATP6V1F
SBDS1	5,34E-05	0,454201	0,923	0,969	1	11	SBDS
LMNA4	5,73E-05	0,576939	0,974	0,999	1	11	LMNA
PFDN1	6,03E-05	0,480355	0,769	0,827	1	11	PFDN1
CSTB	6,60E-05	0,348683	1	0,998	1	11	CSTB
ZBTB8OS	7,18E-05	0,575439	0,718	0,818	1	11	ZBTB8OS
PEA151	7,39E-05	0,516652	0,846	0,907	1	11	PEA15
PTMS2	7,56E-05	0,393748	0,974	0,998	1	11	PTMS
CEBPZOS	8,02E-05	0,388051	0,744	0,79	1	11	CEBPZOS
PPDPF1	8,44E-05	0,326153	1	0,999	1	11	PPDPF
MORF4L11	9,11E-05	0,436974	1	0,998	1	11	MORF4L1
FARSB	9,52E-05	0,314364	0,538	0,385	1	11	FARSB
ETHE1	0,000105	0,378234	0,974	0,981	1	11	ETHE1
TMEM261	0,000106	0,433871	0,923	0,927	1	11	TMEM261
CDKN32	0,000112	0,696808	0,513	0,342	1	11	CDKN3
COMMD42	0,000112	0,412158	0,846	0,917	1	11	COMMD4
RALB	0,000121	0,286647	0,667	0,613	1	11	RALB
DTD11	0,00013	0,352589	0,769	0,805	1	11	DTD1
TYMS2	0,000131	0,919245	0,487	0,326	1	11	TYMS
H2AFJ	0,000134	0,410795	0,974	0,992	1	11	H2AFJ
NDUFB91	0,000142	0,408247	1	0,991	1	11	NDUFB9
HPRT11	0,000143	0,431284	0,641	0,574	1	11	HPRT1
OAZ1	0,000144	0,261179	1	1	1	11	OAZ1
MRPL241	0,000147	0,490429	0,692	0,71	1	11	MRPL24
OST42	0,000151	0,288874	1	1	1	11	OST4
RNF7	0,000153	0,32482	0,974	0,991	1	11	RNF7
VDAC33	0,000154	0,486208	0,872	0,92	1	11	VDAC3
NUCB22	0,000158	0,371275	0,872	0,917	1	11	NUCB2
IL33	0,000159	0,656872	0,487	0,31	1	11	IL33
NDUFB11	0,000161	0,381632	0,949	0,989	1	11	NDUFB11
CAMTA1	0,000168	0,360787	0,923	0,955	1	11	CAMTA1
GDI2	0,000173	0,433843	0,846	0,979	1	11	GDI2
NSA2	0,000178	0,376719	0,923	0,982	1	11	NSA2
C17orf893	0,000194	0,311525	0,974	0,987	1	11	C17orf89
PLIN33	0,000203	0,404586	0,872	0,93	1	11	PLIN3
PSMC53	0,000203	0,388301	0,923	0,943	1	11	PSMC5
ENY21	0,000207	0,38099	0,949	0,985	1	11	ENY2
PSMA32	0,000214	0,393691	0,897	0,963	1	11	PSMA3
HMGB12	0,000227	1,011404	0,974	0,997	1	11	HMGB1
CISD11	0,000236	0,435735	0,949	0,972	1	11	CISD1
ASNA1	0,000254	0,299999	0,872	0,936	1	11	ASNA1
FKBP8	0,000258	0,289693	0,974	0,991	1	11	FKBP8
NAA10	0,000268	0,346548	0,897	0,979	1	11	NAA10
BCCIP	0,000269	0,304278	0,615	0,545	1	11	BCCIP
DECR11	0,00028	0,482972	0,872	0,925	1	11	DECR1
FAM127A	0,00028	0,384197	1	0,995	1	11	FAM127A
PSMC12	0,000294	0,577284	0,769	0,897	1	11	PSMC1
LSM52	0,000297	0,644169	0,821	0,945	1	11	LSM5

CD592	0,0003	0,596215	0,949	0,998	1	11	CD59
TFPI21	0,000317	0,834067	0,897	0,938	1	11	TFPI2
LDHA4	0,000321	0,656022	0,949	0,994	1	11	LDHA
FABP4	0,000321	0,273599	0,359	0,184	1	11	FABP4
SMDT1	0,00033	0,344616	0,923	0,954	1	11	SMDT1
DDT	0,000336	0,416843	0,949	0,994	1	11	DDT
GHITM1	0,00035	0,42027	0,872	0,982	1	11	GHITM
SUMO32	0,000354	0,437012	0,897	0,955	1	11	SUMO3
CKS1B2	0,000366	1,016676	0,59	0,534	1	11	CKS1B
PFDN22	0,000366	0,507209	0,821	0,947	1	11	PFDN2
NFU1	0,00037	0,337381	0,769	0,824	1	11	NFU1
AK1	0,000375	0,38398	0,846	0,906	1	11	AK1
POLR2F2	0,000389	0,440386	0,897	0,974	1	11	POLR2F
LAMTOR5	0,00039	0,333866	1	0,995	1	11	LAMTOR5
HSPB62	0,000418	0,841237	0,769	0,847	1	11	HSPB6
NDUFA3	0,000429	0,299896	0,897	0,956	1	11	NDUFA3
MRPS101	0,000435	0,365997	0,769	0,847	1	11	MRPS10
DDA11	0,000445	0,419717	0,872	0,9	1	11	DDA1
MRPL402	0,000505	0,391469	0,821	0,9	1	11	MRPL40
C20orf272	0,000528	0,344847	0,744	0,8	1	11	C20orf27
POLR2I	0,000532	0,275927	0,846	0,953	1	11	POLR2I
VTI1B	0,000562	0,316056	0,949	0,957	1	11	VTI1B
RPL26L12	0,000579	0,302239	0,821	0,945	1	11	RPL26L1
RAB11A1	0,000589	0,398672	0,897	0,961	1	11	RAB11A
LAMTOR2	0,000612	0,304054	0,897	0,956	1	11	LAMTOR2
ANAPC13	0,000625	0,426032	0,795	0,934	1	11	ANAPC13
NDUFS81	0,000678	0,285598	0,974	0,988	1	11	NDUFS8
FXYD51	0,000679	0,426162	0,846	0,984	1	11	FXYD5
TAX1BP3	0,000714	0,333834	0,897	0,99	1	11	TAX1BP3
CNN11	0,000769	0,69491	0,692	0,703	1	11	CNN1
ATP5D	0,000776	0,2894	1	0,998	1	11	ATP5D
NDUFA123	0,000789	0,437243	0,821	0,929	1	11	NDUFA12
ST131	0,000814	0,277113	0,974	0,999	1	11	ST13
ERH2	0,00086	0,376062	0,949	0,995	1	11	ERH
PSMB13	0,000896	0,323809	1	0,995	1	11	PSMB1
SSB1	0,000897	0,343778	0,923	0,966	1	11	SSB
PDZD11	0,000902	0,284318	0,821	0,861	1	11	PDZD11
IDH3G1	0,000977	0,348259	0,795	0,837	1	11	IDH3G
PSMD42	0,000987	0,481837	0,872	0,957	1	11	PSMD4
TCP13	0,001027	0,390227	0,821	0,938	1	11	TCP1
SH3BGRL1	0,001028	0,327481	1	0,975	1	11	SH3BGRL
UFC1	0,001089	0,339901	0,949	0,979	1	11	UFC1
CCT23	0,001154	0,467947	0,795	0,906	1	11	CCT2
NDUFA82	0,00119	0,315414	0,897	0,936	1	11	NDUFA8
PRDX51	0,001231	0,273343	1	0,999	1	11	PRDX5
SUCLG11	0,001319	0,302652	0,795	0,899	1	11	SUCLG1
CSNK2B	0,001329	0,276166	0,923	0,989	1	11	CSNK2B
CCT53	0,001332	0,473877	0,821	0,88	1	11	CCT5
GADD45GII	0,001373	0,365433	0,974	0,994	1	11	GADD45GIP1
NDUFA10	0,001382	0,285784	0,897	0,951	1	11	NDUFA10

TPMT	0,001395	0,256476	0,487	0,374	1	11	TPMT
MRPL132	0,001401	0,377855	0,769	0,885	1	11	MRPL13
HMGB32	0,00144	0,415609	0,564	0,501	1	11	HMGB3
PSIP11	0,001467	0,375981	0,692	0,746	1	11	PSIP1
EMP33	0,00147	0,395712	0,974	0,999	1	11	EMP3
ACP1	0,001473	0,274839	0,923	0,978	1	11	ACP1
HNRNPA2E	0,001513	0,539977	0,872	0,995	1	11	HNRNPA2B1
CAPG1	0,001569	0,363087	0,667	0,692	1	11	CAPG
SNRPD31	0,001586	0,331134	0,872	0,939	1	11	SNRPD3
RPL36A	0,001596	0,35881	1	0,995	1	11	RPL36A
SEC132	0,001624	0,417531	0,821	0,928	1	11	SEC13
FKBP22	0,001631	0,328811	0,923	0,987	1	11	FKBP2
RAB5C	0,001728	0,379137	0,897	0,979	1	11	RAB5C
COPS8	0,001843	0,444777	0,744	0,836	1	11	COPS8
MPC23	0,001901	0,373207	0,949	0,988	1	11	MPC2
TTC1	0,001965	0,310741	0,795	0,832	1	11	TTC1
ARPC1B2	0,001992	0,308211	0,974	0,996	1	11	ARPC1B
SDCBP2	0,002151	0,338294	0,872	0,988	1	11	SDCBP
ARF12	0,002164	0,405407	0,949	0,991	1	11	ARF1
CHCHD10	0,002246	0,42082	0,821	0,856	1	11	CHCHD10
TIMP12	0,00231	0,729799	1	1	1	11	TIMP1
ACADVL1	0,002317	0,310309	0,718	0,738	1	11	ACADVL
TPD52L2	0,002317	0,32727	0,744	0,896	1	11	TPD52L2
TUBA1B3	0,002325	1,149052	0,974	0,995	1	11	TUBA1B
ANXA71	0,00236	0,370053	0,795	0,882	1	11	ANXA7
ZNHIT1	0,002462	0,283038	0,923	0,992	1	11	ZNHIT1
NDUFS4	0,002464	0,323427	0,923	0,983	1	11	NDUFS4
C16orf131	0,002599	0,297595	0,897	0,957	1	11	C16orf13
LINC00998	0,002603	0,322666	0,897	0,946	1	11	LINC00998
TUBB3	0,002622	0,455472	0,974	0,999	1	11	TUBB
MRPL471	0,00268	0,301606	0,821	0,882	1	11	MRPL47
CDKN2AIPN	0,002708	0,424891	0,564	0,532	1	11	CDKN2AIPNL
DNAJC15	0,002749	0,377705	0,769	0,877	1	11	DNAJC15
CCT32	0,002756	0,287685	0,923	0,974	1	11	CCT3
MRPL421	0,002759	0,305331	0,718	0,802	1	11	MRPL42
CCDC1241	0,002772	0,330644	0,769	0,905	1	11	CCDC124
POLE42	0,003022	0,38319	0,923	0,976	1	11	POLE4
MAPRE11	0,003026	0,402098	0,692	0,822	1	11	MAPRE1
RNASEH2A	0,003048	0,336072	0,333	0,197	1	11	RNASEH2A
METAP2	0,003073	0,432579	0,821	0,963	1	11	METAP2
ARPC32	0,003087	0,342881	0,974	0,997	1	11	ARPC3
TMEM256	0,003115	0,30796	0,846	0,909	1	11	TMEM256
H2AFV2	0,003191	0,286381	0,872	0,936	1	11	H2AFV
ZWINT1	0,00327	0,359058	0,282	0,148	1	11	ZWINT
NANS4	0,00336	0,287797	0,821	0,851	1	11	NANS
TSR2	0,003423	0,439454	0,564	0,594	1	11	TSR2
COMMD1	0,003454	0,261164	0,769	0,848	1	11	COMMD1
CCND21	0,003493	0,510622	0,667	0,627	1	11	CCND2
RAB3B1	0,003518	0,338854	0,667	0,683	1	11	RAB3B
RAB132	0,003585	0,801042	0,974	0,996	1	11	RAB13

OSTF1	0,003695	0,369259	0,59	0,638	1	11	OSTF1
TIMM13	0,003718	0,269077	0,974	0,992	1	11	TIMM13
BOLA33	0,003781	0,413697	0,821	0,874	1	11	BOLA3
PLP22	0,004001	0,432823	0,846	0,939	1	11	PLP2
PSMB23	0,004027	0,336028	0,923	0,975	1	11	PSMB2
NCBP2	0,00408	0,282687	0,769	0,848	1	11	NCBP2
NDUFA91	0,004096	0,272779	0,744	0,789	1	11	NDUFA9
PTS2	0,00413	0,320972	0,846	0,905	1	11	PTS
OCIAD2	0,004186	0,377778	0,769	0,807	1	11	OCIAD2
CAPZA11	0,004255	0,342589	0,846	0,961	1	11	CAPZA1
PTGES32	0,004361	0,538321	0,949	0,985	1	11	PTGES3
NDUFB5	0,004562	0,283934	0,846	0,981	1	11	NDUFB5
DUT2	0,004587	0,330855	0,821	0,928	1	11	DUT
CENPW2	0,004595	0,490617	0,462	0,374	1	11	CENPW
STRAP1	0,00468	0,283279	0,923	0,982	1	11	STRAP
ANAPC152	0,004832	0,296133	0,821	0,894	1	11	ANAPC15
SAE11	0,004932	0,427969	0,641	0,734	1	11	SAE1
EEF1E12	0,005093	0,378901	0,641	0,64	1	11	EEF1E1
CCT42	0,005306	0,414576	0,821	0,948	1	11	CCT4
KXD11	0,005521	0,262374	0,795	0,923	1	11	KXD1
NUDT12	0,005548	0,426655	0,59	0,64	1	11	NUDT1
CAV13	0,005568	0,451783	1	0,999	1	11	CAV1
CNIH1	0,005694	0,290616	0,897	0,972	1	11	CNIH1
SPCS12	0,005776	0,26061	0,974	0,99	1	11	SPCS1
MVP1	0,005922	0,288239	0,872	0,95	1	11	MVP
TXN2	0,005937	0,288637	0,897	0,963	1	11	TXN2
FDPS2	0,006152	0,261058	0,949	0,977	1	11	FDPS
HSPB112	0,0063	0,410476	0,795	0,901	1	11	HSPB11
CLIC4	0,006673	0,269753	0,923	0,977	1	11	CLIC4
PABPC4	0,006945	0,302941	0,923	0,943	1	11	PABPC4
ARHGDIA2	0,006977	0,337309	0,949	0,976	1	11	ARHGDIA
PET100	0,007147	0,291928	0,692	0,844	1	11	PET100
ARL31	0,007257	0,258126	0,821	0,883	1	11	ARL3
MT1E4	0,007329	0,664723	0,872	0,964	1	11	MT1E
NUCKS11	0,007817	0,387825	0,897	0,995	1	11	NUCKS1
CINP	0,008719	0,317206	0,692	0,791	1	11	CINP
MRPS33	0,009223	0,280284	0,846	0,964	1	11	MRPS33
GLTP	0,009898	0,307195	0,641	0,713	1	11	GLTP

Table S4. Top 30 expressed genes in each CAF subtype for bulk RNA seq CAF classification in TCGA

c1-CAFm	c2-CAFir	c4-CAFa	c6-CAFE	c7-CAFP	c8-CAFap
RGS5	COL1A1	CFD	RGS5	S100B	RGS5
TAGLN	COL1A2	DCN	IGFBP7	GPM6B	ACTA2
ACTA2	COL3A1	FBLN1	SPARCL	CRYAB	NDUFA4L2
MYL9	MMP11	APOD	COL4A1	FXYD1	TAGLN
TPM2	CTHRC1	PTGDS	NDUFA4	LGI4	CALD1
NDUFA4	SFRP2	SFRP2	SPRY1	PLP1	PDGFRB
IGFBP7	SPARC	LUM	C11orf96	PMP22	MYL9
CALD1	LUM	MGP	COL4A2	NRXN1	NOTCH3
ADIRF	AEBP1	CCDC80	CALD1	CDH19	TPM2
MYH11	COL6A3	IGFBP6	SLC9A3F	TPPP3	COL4A1
PPP1R1	POSTN	C7	PLVAP	SCN7A	SPARCL1
C11orf96	FN1	SERPINF	RAMP2	ALDH1A	MAP1B
SPARCL	COL6A2	SFRP4	INSR	CD9	LHFPL6
PDGFRB	BGN	C1S	FLT1	SEMA3B	PPP1R14A
NOTCH3	COL5A2	C1R	TIMP3	TMEM17	STEAP4
SOD3	COL6A1	MFAP4	SPARC	CLU	CRISPLD2
BGN	THBS2	GSN	TCF4	MYOT	COL4A2
HIGD1B	MMP2	DPT	GNG11	SPARC	HIGD1B
IGFBP5	COL5A1	COL6A2	HSPG2	TMEM17	ADAMTS1
MYLK	RARRES	C3	STC1	MIA	BGN
FRZB	VCAN	RARRES	RGCC	PRNP	ADAMTS4
MAP1B	COL12A	CYR61	PLPP1	CNN3	COX4I2
MFGE8	COL11A	MMP2	CRIP2	TUBA1A	SYNPO2
PLN	CALD1	COL1A2	MYL9	MATN2	MYLK
CSRP2	COL10A	ADH1B	CLDN5	GPR155	PLAC9
DSTN	SFRP4	COL3A1	HIGD1B	AP1S2	FRZB
COL4A1	TAGLN	PLAC9	VWF	MPZ	GJA4
COL18A	CTSK	IGFBP5	PLPP3	NDRG2	CAVIN3
COX4I2	ASPN	PCOLCE	A2M	MAL	CD163
TPM1	CXCL14	CTGF	IGFBP3	ABCA8	MCAM
